# Machine learning models for rat multigeneration reproductive toxicity prediction

**DOI:** 10.3389/fphar.2022.1018226

**Published:** 2022-09-27

**Authors:** Jie Liu, Wenjing Guo, Fan Dong, Jason Aungst, Suzanne Fitzpatrick, Tucker A. Patterson, Huixiao Hong

**Affiliations:** ^1^ National Center for Toxicological Research, U.S. Food and Drug Administration, Jefferson, AR, United States; ^2^ Center for Food Safety and Applied Nutrition, U.S. Food and Drug Administration, College Park, MD, United States

**Keywords:** multigeneration reproductive toxicity, machine learning, molecular descriptor, consensus model, toxicity prediction

## Abstract

Reproductive toxicity is one of the prominent endpoints in the risk assessment of environmental and industrial chemicals. Due to the complexity of the reproductive system, traditional reproductive toxicity testing in animals, especially guideline multigeneration reproductive toxicity studies, take a long time and are expensive. Therefore, machine learning, as a promising alternative approach, should be considered when evaluating the reproductive toxicity of chemicals. We curated rat multigeneration reproductive toxicity testing data of 275 chemicals from ToxRefDB (Toxicity Reference Database) and developed predictive models using seven machine learning algorithms (decision tree, decision forest, random forest, k-nearest neighbors, support vector machine, linear discriminant analysis, and logistic regression). A consensus model was built based on the seven individual models. An external validation set was curated from the COSMOS database and the literature. The performances of individual and consensus models were evaluated using 500 iterations of 5-fold cross-validations and the external validation data set. The balanced accuracy of the models ranged from 58% to 65% in the 5-fold cross-validations and 45%–61% in the external validations. Prediction confidence analysis was conducted to provide additional information for more appropriate applications of the developed models. The impact of our findings is in increasing confidence in machine learning models. We demonstrate the importance of using consensus models for harnessing the benefits of multiple machine learning models (i.e., using redundant systems to check validity of outcomes). While we continue to build upon the models to better characterize weak toxicants, there is current utility in saving resources by being able to screen out strong reproductive toxicants before investing *in vivo* testing. The modeling approach (machine learning models) is offered for assessing the rat multigeneration reproductive toxicity of chemicals. Our results suggest that machine learning may be a promising alternative approach to evaluate the potential reproductive toxicity of chemicals.

## Introduction

Reproductive toxicity refers to a group of adverse effects caused by chemical substances on the reproductive systems of males and females, such as alterations in fertility, implantation, and estrous cycle. Safety evaluation of chemicals, including reproductive toxicity assessment, is required by regulatory agencies before authorization of the usage of many chemicals, and is important in the process of new product development for industries (Novic and Vracko 2010). To assess the reproductive toxicity of a chemical, various *in vivo* animal testing methods are typically used. A multigeneration reproductive toxicity study has a complex study design which contains multiple factors (OECD 2001; Piersma et al., 2011) such as chemical exposure to two or more generations, different dosing times, and dosing duration. Various reproductive toxicity endpoints are observed in both parental and offspring generations in a multigeneration reproductive toxicity study, which uses a number of animals and can take months to complete (Hofer et al., 2004). Therefore, *in vivo* animal testing for the assessment of reproductive toxicity for a chemical is time-consuming and expensive (Rorije et al., 2011; Beekhuijzen 2017). It is not practically feasible to assess reproductive toxicity using *in vivo* animal testing for all chemicals (Ashburn and Thor 2004; Paul et al., 2010; Tornqvist et al., 2014).

Another issue associated with assessing reproductive toxicity using *in vivo* animal testing is the ethical concern of animal use. The principle of 3Rs (Replacement, Reduction and Refinement) was proposed over 50 years ago to guide animal use (Tannenbaum and Bennett 2015; Brannen et al., 2016; Fischer et al., 2020). Replacement refers to substituting the use of animal models with non-animal alternative methods. Reduction refers to using the minimal number of animals required to demonstrate statistical significance. Refinement aims to minimize the potential pain and suffering of animals in the experiments. The scientific community, regulatory agencies, and industry are searching for efficient alternative approaches to animal models in reproductive toxicity assessment.

Two types of alternative methods for animal testing, *in vitro* and in silico methods, have been explored. While the multi-tissue reproductive system is probably one of the most complex systems, it is difficult to mimic the whole reproductive system using an *in vitro* model (Brannen et al., 2016; Nikolaidis 2017). Therefore, in silico methods as an alternative approach have attracted more effort and interest for reproductive toxicity prediction with their power and efficiency and have been widely used in reproductive toxicity assessment (Zhang et al., 2020). Some in silico models have been built for predicting potential reproductive toxicity (Martin et al., 2011; Basant et al., 2016; Jiang et al., 2019; Feng et al., 2021). Martin et al. built predictive models for reproductive toxicity by combining the *in vivo* multigeneration reproductive toxicity testing data and *in vitro* high-throughput screening assay data (Martin et al., 2011). Their model was evaluated only by a one-time 5-fold cross-validation, and thus the performance is not statistically robust. Another barrier for application of their model is the need for *in vitro* high-throughput screening assay data that are not available for new chemicals. Feng et al. generated ensemble models based on individual models that were constructed using three machine learning algorithms and nine sets of molecular fingerprints of 1,823 chemicals for reproductive toxicity prediction (Feng et al., 2021). This dataset was originally generated to build machine learning models for predicting reproductive toxicity (Jiang et al., 2019). Though both articles reported similar high prediction accuracies for their models, it is challenging to ensure the reliability of the results because of the quality issues of the dataset used. The reproductive toxicity data are heterogeneous and not solely from multigeneration *in vivo* animal testing results. Therefore, the reproductive toxicity data are not suitable for training a model for specifically predicting multigeneration reproductive toxicity in in vivo animal testing. For example, ethanol is defined as a reproductive toxic chemical, because reproductive toxicity was reported from a poorly designed study in the literature. In the 28-day study comparing ginger extract and ethanol on reproductive toxicity, a single high dose (4 g/kg) of ethanol, much higher than the commonly used doses for testing the lowest-observed-adverse effect level (LOAEL), was found to increase total homocysteine and malondialdehyde compared with the control (corn oil) and ginger groups for which a much lower dose (1 g/kg) was used (Akbari et al., 2017). Besides classification models, regression models were reported for reproductive toxicity. Basant et al. collected multigeneration reproductive toxicity study data from Toxicity Reference Database (ToxRefDB) and constructed in silico models to predict LOAEL of reproductive toxic chemicals (Basant et al., 2016). Computational models for predicting potential rat multigeneration reproductive toxicity likely observed in guideline studies from chemical structure have not been reported and a well curated dataset from guideline studies are not available. Therefore, machine learning models to classify chemicals as potentially toxic or not toxic in rat multigeneration reproductive animal testing solely based on chemical structures are needed and could facilitate chemical risk assessment in terms of multigeneration reproductive toxicity.

The goal of this study was to construct machine learning models using *in vivo* animal testing data for screening chemicals with potential reproductive toxicity. Accordingly, machine learning models were established and validated by cross-validations and external validations. The models showed the potential utility in screening potential toxicants of rat multigeneration reproductive toxicity and provide an additional tool in assisting the decision-making in regulatory science when experimental data are not available or limited. Our study suggests that machine learning could be a promising alternative approach in chemical risk assessment.

## Materials and methods

### Study design

**FIGURE 1:**
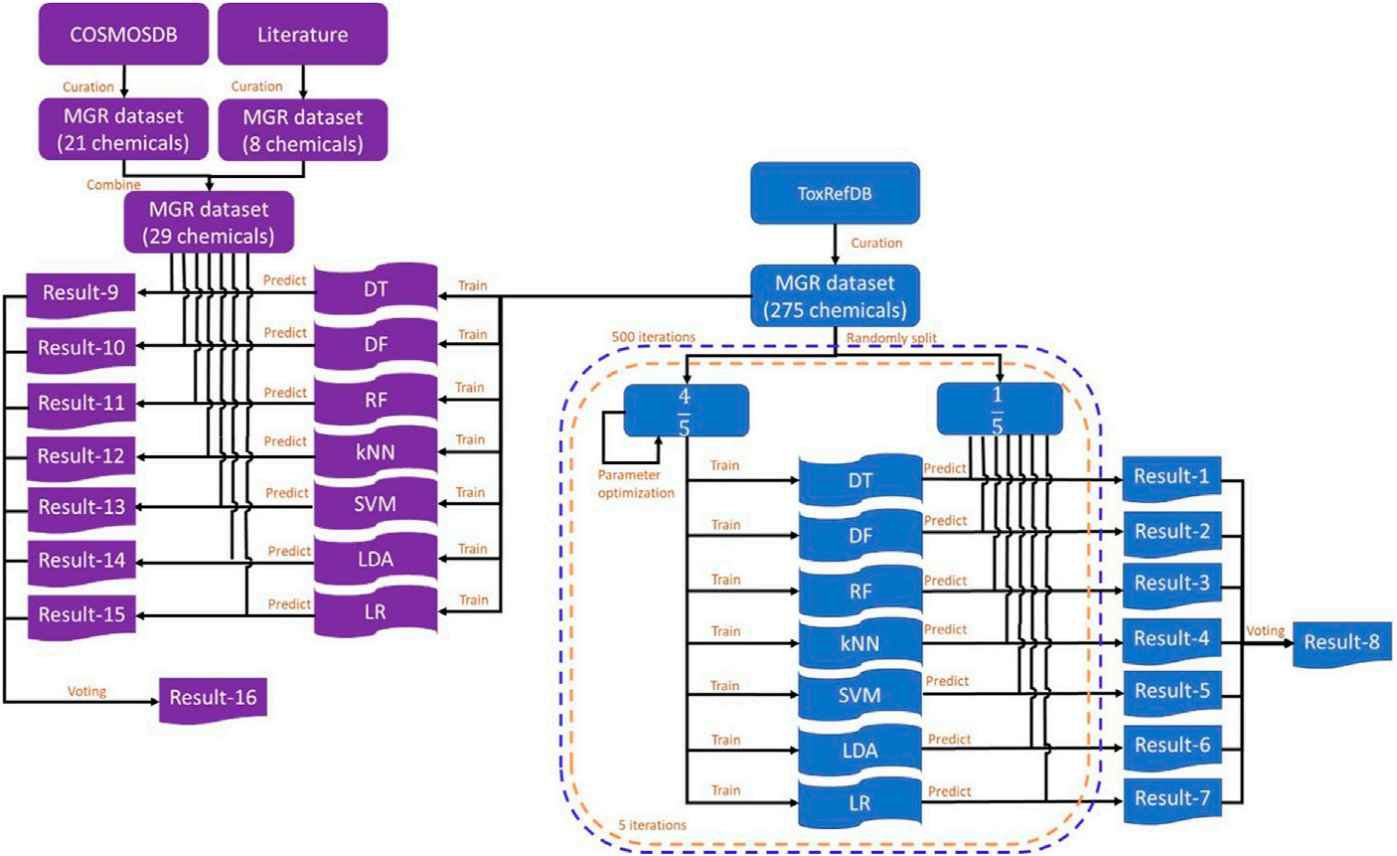
Study design. The training dataset was curated from ToxRefDB and the external validation dataset was curated from the COSMOS database and the literature. The training dataset was randomly split into five folds. Four folds were used to build models using each of the seven machine learning algorithms and the remaining fold was used to evaluate the constructed models. Predictions were recorded in Result-1 to Result-7 and the consensus predictions were generated and recorded in Result-8. This process was repeated five times until each of the five folds was used as a testing set once and only once to complete a 5-fold cross-validation. The 5-fold cross-validation was iterated 500 times. The whole training dataset was used to construct models using the same machine learning algorithms and results were recorded in Result-9 to Result-16. MGR: multigeneration reproductive; DT, decision tree; DF, decision forest; RF, random forest; kNN, k-nearest neighbors; SVM, support vector machine; LDA, linear discriminant analysis; LR, logistic regression.

The study design is illustrated in Figure 1. ToxRefDB contains high quality *in vivo* animal studies (Martin et al., 2009). The database includes the information of chemicals, study design, treatment-related effects, and effect levels. Rat multigeneration reproductive toxicity data were curated from ToxRefDB (https://www.epa.gov/chemical-research/exploring-toxcast-data-downloadable-data, version November 2014) and used as the training dataset. The external validation dataset included rat multigeneration reproductive toxicity data curated from the COSMetics to Optimise Safety (COSMOS) database (https://archive.cosmostox.org/home/welcome/, accessed on February 16, 2021) and the literature. The training dataset was used to build predictive models using seven machine learning algorithms: decision tree (DT), decision forest (DF), random forest (RF), k-nearest neighbors (kNN), support vector machine (SVM), linear discriminant analysis (LDA), and logistic regression (LR). In addition, a consensus model was generated from the seven individual models using majority voting. Five iterations of inner 5-fold cross-validations were performed for parameter tuning. Then, 500 iterations of 5-fold cross-validations were performed to evaluate performance of the machine learning models. Prediction confidence analysis was conducted on the results of 5-fold cross-validations. Machine learning models were built from the whole training dataset and evaluated by the external validation dataset.

### Datasets

Rat multigeneration reproductive toxicity studies were queried from ToxRefDB based on the following inclusion criteria: 1) multigeneration reproductive toxicity study; 2) oral dose administration in rats; 3) acceptable (data usability); 4) complete (data entry status); 5) all effects (data entry level); 6) testing and observation of reproductive effects. Multigeneration reproductive toxicity studies in ToxRefDB have three categories of treatment-related effect endpoints, “Parental,” “Offspring,” and “Reproductive.” Only the endpoints in the category of “Reproductive” were selected as the effects of reproductive toxicity. Chemicals with LOAEL data at the effect category of “Reproductive” were assigned as positives of reproductive toxicity. The chemicals without LOAEL were considered as negatives of reproductive toxicity. Mixtures of compounds were removed, and the remaining data were taken as the training dataset.

Rat multigeneration reproductive toxicity studies with the same data inclusion criteria were obtained from the COSMOS database and literature. After removing the chemicals contained in the training dataset, the remaining chemicals were used as the external validation dataset to validate the models constructed from the training dataset. The categories of the chemicals of the training and the external validation datasets are summarized in Table 1.

**TABLE 1:** Chemical category distribution of the training dataset and external validation dataset.

Data set (total chemicals)	Chemical category	Number of chemicals
Training data (275)	drug	12
	cosmetic	12
	food contact substance	8
	pesticide	243
External data (29)	drug	6
	cosmetic	15
	food additive/food contact	5
	pesticide	1
	research chemical	2

### Molecular descriptors calculation and pre-processing

Simplified molecular input line entry system (SMILES) codes of the compounds were collected from the CompTox Chemicals Dashboard (https://comptox.epa.gov/dashboard/chemical_lists/TOXREFDB2) and PubChem (https://pubchem.ncbi.nlm.nih.gov/). Two-dimensional (2D) structures were then generated using the Online SMILES Translator and Structure File Generator (https://cactus.nci.nih.gov/translate/) and output as structural description files (SDF).

Mold2 is a software that calculates molecular descriptors from 2D chemical structures (Hong et al., 2008; Hong et al., 2017). Mold2 descriptors include chemical physical property, counts for atoms, counts for bonds, counts for functional groups, structural features, 2D autocorrelation, Balaban index, connectivity index, distance (topological) index, eigen value-based descriptors, information content, Kier index, molecular walk counts, Schultz index, topological charge index, Wiener index, and Zagreb index. Therefore, it was used to calculate molecular descriptors from the SDF files for the compounds of training and external validation datasets. Mold2 calculated 777 molecular descriptors for each chemical. Some of these 777 descriptors convey little or no information for subsequent machine learning. Therefore, such low informative descriptors were removed. First, molecular descriptors with zero values for more than 90% of the compounds in the training dataset were discarded. Shannon entropy analysis (Godden et al., 2000) was then applied on the remaining descriptors to select molecular descriptors with high information. More specifically, the range of values of a molecular descriptor in the training dataset (the minimum to the maximum) was divided into 20 even bins. The chemicals were then assigned to these 20 bins based on their molecular descriptor values. Shannon entropy value for the descriptor was calculated according to the distribution of the chemicals in these 20 bins by Eq. 1.

$$H_{n}\left(p_{1},p_{2},...,p_{n}\right)=-\sum_{i=1}^{n}p_{i}\;\log_{2}p_{i}$$ (1)

Where $p_{i}$ is the ratio of chemicals in bin i to the total number of chemicals. Descriptors with Shannon entropy less than 2.5 were removed.

The descriptors with Shannon entropy greater than 2.5 were further processed using decision tree-based machine learning algorithms DT, DF, and RF. In brief, the training chemicals were randomly divided into five folds. Four folds were used to build DT, DF, and RF models based on the descriptors with Shannon entropy greater than 2.5. The descriptors used in the models were recorded. This process was repeated five times with five different combinations of four folds to build DT models. The whole process was iterated 500 times with different random divisions (using different random seeds). The frequency values of a descriptor were calculated by counting the DT, DF, and RF models that used the descriptor in the 500 cross-validations. Importance of a descriptor was calculated as the ratio of its frequency to the maximum frequency among all descriptors, separately for DT, DF, and RF. The overall importance of the descriptor was calculated as the average importance among DT, DF, and RF. Descriptors with overall importance greater than 20% were kept for subsequent model development with machine learning algorithms.

### Model development

Seven different machine learning algorithms were used for classification, including DT, DF, RF, kNN, SVM, LDA, and LR. DT is a popular approach for classification. It uses a tree-like structure to represent the predictions based on a series of features (Kingsford and Salzberg 2008; Karalis 2020). DF combines multiple decision trees and builds the ensemble model (Tong et al., 2003; Hong et al., 2004; Hong et al., 2005; Chen et al., 2013; Ng et al., 2015a; Ng et al., 2015b; Hong et al., 2017; Sakkiah et al., 2017; Hong et al., 2018; Sakkiah et al., 2020). RF is an ensemble learning algorithm (Breiman 2001). kNN generates the prediction for a sample based on the distance of *k* samples that are nearest (Baskin 2018). SVM searches for a hyperplane that can separate different groups of samples (Cortes and Vapnik 1995; Cristianini and Shawe-Taylor 2000; Christmann and Steinwart 2008). LDA is an algorithm which assumes that all data are normally distributed (Martin et al., 2011; Sipes et al., 2011). LR is a popular algorithm for supervised binary classification (Cai et al., 2019).

Tuning algorithmic parameters is a critical step in model development. When developing a model from a training dataset, a subset of 80% of the chemicals were used to tune parameters for the seven machine learning algorithms. In short, for each of the seven machine learning algorithms (DT, DF, RF, kNN, SVM, LDA, and LR), a set of algorithmic parameters was set up. This data subset was then randomly split into five folds. One fold was held out and the other four folds were used to build a model. The developed model was used to predict the held out chemicals. This process was repeated till each of the five folds was held out once and only once. Matthews correlation coefficient (MCC) was calculated from the predictions on the five folds. This inner 5-fold cross-validation was repeated five times. An average MCC value was calculated from the five MCC values. The algorithmic parameters were then changed to repeat the whole process. The parameters that resulted in the highest average MCC were selected for subsequent model development for the algorithm. With the optimized parameters, the training dataset curated from ToxRefDB was used in cross-validations and in external validation.

Based on the seven individual models constructed using DT, DF, RF, kNN, SVM, LDA, and LR, a consensus machine learning model was built by voting predictions from the seven individual models. More specifically, the consensus model predicts a chemical as positive if more than three of the seven models predict the chemical as positive, otherwise the chemical is predicted as negative (Supplementary Figure S1). The probability to predict a chemical as positive output from the consensus model is calculated using Eq. 2 when more than three individual models predict the chemical as positive or using Eq. 3 when three or less individual models predict the chemical as positive.

$$Positive-probability=1-\frac{7-N^{+}}{8}$$ (2)

$$Positive-probability=\frac{N^{+}}{6}$$ (3)

Where *N* ^*+*^ indicates the number of models that predict the chemical as positive. The consensus modeling was conducted using an in-house Matlab script.

### Cross-validations

The 5-fold cross-validation approach was used to estimate performance of machine learning models in prediction of rat multigeneration reproductive toxicity. In a 5-fold cross-validation, the dataset curated from ToxRefDB was randomly split into five folds of equal size. Four folds were used as training data, while the remaining fold was used as testing data. Predictive models were constructed using the training data with seven machine learning algorithms (DT, DF, RF, kNN, SVM, LDA, and LR) and then were tested using the testing data. In addition, a consensus model was generated based on the seven machine learning models and was tested using the testing data. This process was repeated five times till each of the five folds was used as testing data once and only once. For each of the eight models (seven individual models and one consensus model), the predictions on all five folds were used to calculate performance metrics for evaluating its performance. To reach a statistically robustness in estimation of model performance, the 5-fold cross-validation was iterated 500 times with different random seeds to randomly divide the whole dataset into five folds. The results from the 500 iterations of 5-fold cross-validations were analyzed to evaluate performance of the machine learning models for predicting rat multigeneration reproductive toxicity. The 5-fold cross validations were performed using an in-house Matlab script for DF and Python (3.8.5) Scikit-learn packages (0.23.2) for DT, RF, kNN, SVM, LDA, and LR models.

### External validation

Cross-validations are used to estimate goodness-of-fit of models constructed based on a dataset but are not suitable to generalization of models built from the dataset to the data generated in different situations such as different labs and time. Therefore, external validation is needed for estimating generalization of machine learning models yielded from the rat multigeneration reproductive toxicity dataset curated from ToxRefDB. In the external validations, DT, DF, RF, kNN, SVM, LDA, and LR models were built using the full training dataset curated from ToxRefDB. Then, each of the seven models was applied to predict the potential rat multigeneration reproductive toxicity of chemicals in the external dataset. The consensus predictions by voting of the seven models for the same chemicals were generated as the predictions of the consensus model. Performance metrics were calculated based on the prediction results to assess the external validations.

### Performance measurement

Predictive performance of the machine learning models was measured by seven metrics: accuracy, sensitivity, specificity, balanced accuracy, positive predictive rate, negative predictive rate, and Matthews correlation coefficient (MCC). These metrics for a set of predictions were calculated using Eqs 4–10.

$$Accuracy=\frac{TP+TN}{TP+TN+FP+FN}$$ (4)

$$Sensitivity=\frac{TP}{TP+FN}$$ (5)

$$Specificity=\frac{TN}{TN+FP}$$ (6)

$$Balanced\;\text{​}accuracy=\frac{Sensitivity+Specificity}{2}$$ (7)

$$Positive\;predictive\;rate=\frac{TP}{TP+FP}$$ (8)

$$Negative\;predictive\;rate=\frac{TN}{TN+FN}$$ (9)

$$MCC=\frac{TP*TN-FP*FN}{\sqrt{\left(TP+FP\right)}*\left(TP+FN\right)*\left(TP+FP\right)*\left(TP+FN\right)}$$ (10)

Where TP, TN, FP, and FN represent true positives, true negatives, false positives, and false negatives, respectively.

### Prediction confidence analysis

The output from a machine learning model not only gives a prediction (a class label in classification or a numerical value in regression), but also could provide other parameters that delineate the prediction, depending on the algorithms used. One parameter commonly provided by machine learning models for classification is the probability of a sample (chemical here) that is likely from the class predicted (such as potential rat multigeneration reproductive toxic chemicals). DT uses Eq. 11 to calculate the probability for a chemical to be classified as positive.

$$Probability_{DT}^{positive}=\frac{n^{positive}}{n^{positive}+n^{negative}}$$ (11)

Where *n* ^*positive*^ and *n* ^*negative*^ are number of positive and negative chemicals in the end node the chemical in prediction falls, respectively. Both DF and RF combine multiple trees. They use Eq. 12 to calculate probability for a chemical to be classified as positive.

$$Probability_{DF,RF}^{positive}=\frac{\sum_{i=1}^{n}Probability_{DT_{i}}^{positive}}{n}$$ (12)

kNN uses the fraction of positive chemicals (*n* ^*positive*^) among the k chemicals around the chemical in prediction as the probability for the chemical to be classified as positive which can be calculated using Eq. 13.

$$Probability_{kNN}^{positive}=\frac{n^{positive}}{k}$$ (13)

SVM in scikit-learn do not directly provide probability estimation. Probability to class (e.g., positive) is calculated using Eq. 14 based on an expensive five-fold cross-validation.

$$Probability_{SVM}^{positive}=\frac{1}{1+e^{Af+B}}$$ (14)

Where the parameters A and B are determined by maximum likelihood estimation using cross validation on the training se, and *f* is the uncalibrated value output from SVM. LDA uses Eq. 15 to calculate the probability for a chemical to be classified as positive.

$$Probability_{LDA}^{positive}=e^{a\cdot x+b}$$ (15)

Where b is the intercept and a is the coefficients vector which are determined through training; x is the descriptors vector. LR calculates the probability for a chemical to be classified as positive using Eq. 16.

$$Probability_{LR}^{positive}=\frac{1}{1+e^{-\left(a\cdot x+b\right)}}$$ (16)

Where a is the coefficients vector and b is the intercept which are determined through training; x is the descriptors vector.

In addition to the predicted class, the prediction probability is an informative measurement for assessing the goodness of a machine learning model as this probability indicates the confidence of the prediction. Therefore, prediction confidence was calculated from the probability for a prediction using Eq. 17 and was used to evaluate the confidence of the prediction.

$$prediction\;confidence=\frac{prob-0.5}{0.5}$$ (17)

Where prob is the probability of a chemical to have rat multigeneration reproductive toxic output from a machine learning model. The value of 0.5 is the minimum probability a chemical is predicted as rat multigeneration reproductive toxic. If the probability of a prediction is less than 0.5, the chemical is predicted as a negative and would not be predicted to show rat multigeneration reproductive toxicity. The prediction confidence values range between 0 and 1. A higher confidence value represents the more confidence in the prediction.

In prediction confidence analysis, prediction confidence values were first calculated using the prediction probability values from the 500 iterations of 5-fold cross-validations for each of the eight machine learning algorithms (including the consensus modeling). The prediction confidence values were partitioned into 10 groups with equal range (between 0 and 1 with an internal of 0.1). The performance metrics of predictions in each of the 10 groups were then calculated. Last, the correlation between the prediction performance and prediction confidence level was analyzed.

### Impact of lowest-observed-adverse effect level

In a mutigeneration reproductive toxicity study, different doses are used. LOAEL is the lowest dose at which a reproductive adverse effect is observed for the testing chemical. A low LOAEL indicates that a small amount of the chemical could cause reproductive adverse effects, and thus the chemical is highly toxic. In data curation, all chemicals having LOAEL values recorded in ToxRefDB were categorized as positives. To examine the influence of LOAEL on predictive performance, positive chemicals were divided into two groups, highly toxic chemicals (LOAEL ≤100 mg/kg/day) and chemicals with low toxicity (LOAEL >100 mg/kg/day). The sensitivity was then calculated separately for each of the groups and for each of the machine learning algorithms. Finally, the calculated sesitivity values for the two groups from the same machine learning algorithms were compared.

### Analysis of chemical space

The dataset from ToxRefDB has a limited chemical space (the majority are pesticide compounds) which may influence extrapolation of the machine learning models constructed based on this dataset to new chemicals. To pinpoint possible causes of model performance differences in the cross-validations and external validations, chemical spaces of the training dataset and the external validation dataset were calculated and compared. First, both datasets post pre-processing descriptors were transformed with principal component analysis (PCA). More specifically, for the training dataset represented in principal components (PCs), the centroid of the chemicals was first calculated. The Euclidean distances between the yielded centroid and chemicals in both training and external validation datasets were then computed. The distances from the two datasets were finally statistically compared.

## Results

### Datasets

**FIGURE 2:**
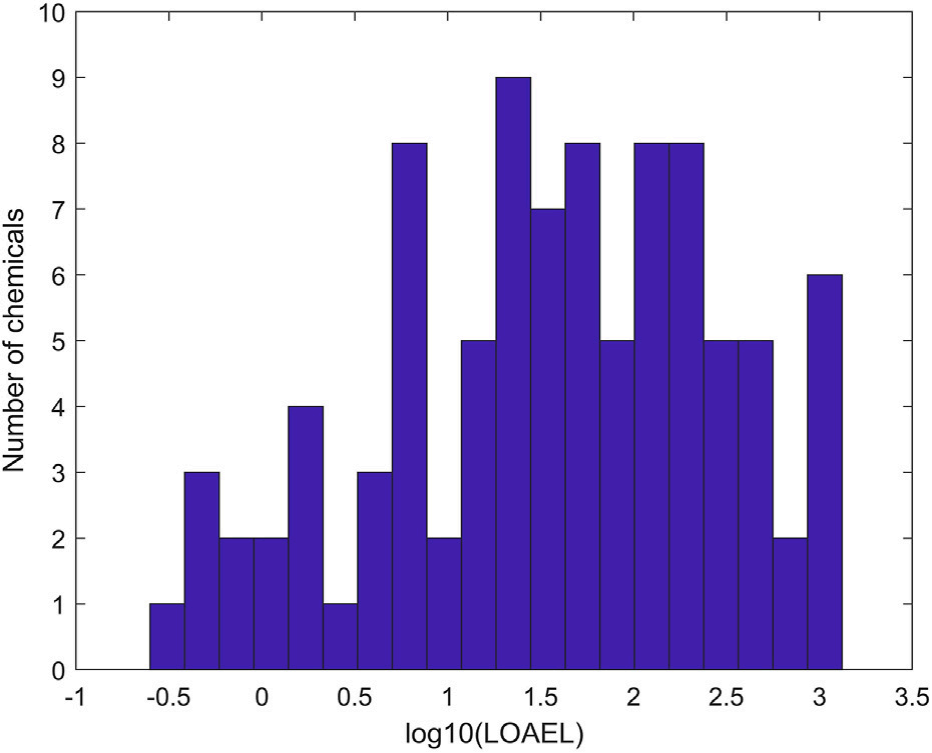
Distribution of positive chemicals in the training dataset at LOAEL (lowest observed adverse effect level) values. The *x*-axis indicates the log10 of LOAEL values. The *y*-axis gives the number of chemicals.

To ensure data quality for model development, only rat multigeneration reproductive studies with acceptable and complete data entries were selected (Pham et al., 2019). In total, 275 chemicals with data from rat multigeneration reproductive studies in ToxRefDB were used as the training chemicals for model development. Of the 275 chemicals, 94 have study LOAEL values of effect category “Reproductive” in ToxRefDB and were assigned as positive chemicals. Figure 2 shows the distribution of LOAEL values of the positive chemicals. The LOAEL values cover a wide range of four magnitudes. The remaining 181 chemicals do not have LOAEL values in ToxRefDB and were assigned as negative chemicals in rat multigeneration reproductive toxicity studies.

For external validation of our models built with training data from ToxRefDB, 29 chemicals with rat multigeneration reproductive toxicity testing (21 from COSMOS database and eight from the literature) were obtained as the external validation dataset. More specifically, of the 970 chemicals in COSMOS, 25 chemicals have rat multigeneration reproductive toxicity study data. Of the 25 chemicals, four exist in the training dataset and were excluded from external validation, and the remaining 21 chemicals were included in the external validation dataset. Of these 21 chemicals, 13 have LOAEL values and were assigned as positive chemicals, while the remaining eight do not have LOAEL and were assigned as negative chemicals. PubMed searching was conducted using combined keywords [rats (Title/Abstract)] AND [reproductive (Title/Abstract)] AND [multigenerational (Title/Abstract)] AND (2012–2021). After discarding publications of reviews, non-multigeneration reproductive toxicity studies, and studies on mixtures or metals or minerals, 10 chemicals were from the published multigeneration reproductive studies. Of the 10, one was included in the 275 training chemicals and one was contained in the 21 external validation chemicals from COSMOS. The remaining eight chemicals were added to the external validation dataset. Of the eight chemicals, five have LOAEL values and were assigned as positive chemicals, while the other three do not have LOAEL values and were assigned as negative chemicals. Finally, 29 unique chemicals (18 positive and 11 negative) were used as the external validation dataset.

The lists of the training and external validation chemicals are provided in Supplementary Tables S1, S2, respectively.

**FIGURE 3:**
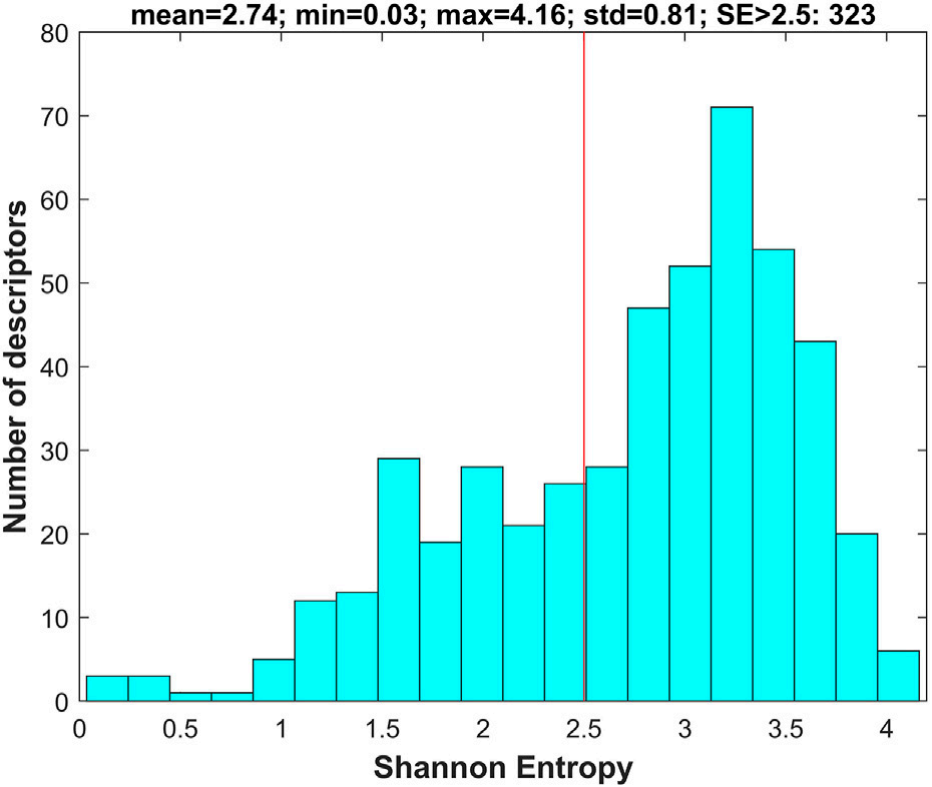
Shannon entropy of molecular descriptors. The *x*-axis indicates Shannon entropy and the *y*-axis depicts the number of descriptors. The red line marks the threshold of Shannon entropy for filtering descriptors with Shannon entropy <2.5.

For each of the chemicals in the training and external validation datasets, 777 molecular descriptors were calculated using software Mold2 (https://www.fda.gov/science-research/bioinformatics-tools/mold2). First, the descriptors with values in less than 10% of the training chemicals were removed, resulting in 505 descriptors in the datasets. Then, Shannon entropy analysis was conducted on the 505 descriptors using the training dataset. Briefly, Shannon entropy was calculated for each of the 505 descriptors. The distribution of the 505 Shannon entropy values is plotted in Figure 3.

**FIGURE 4:**
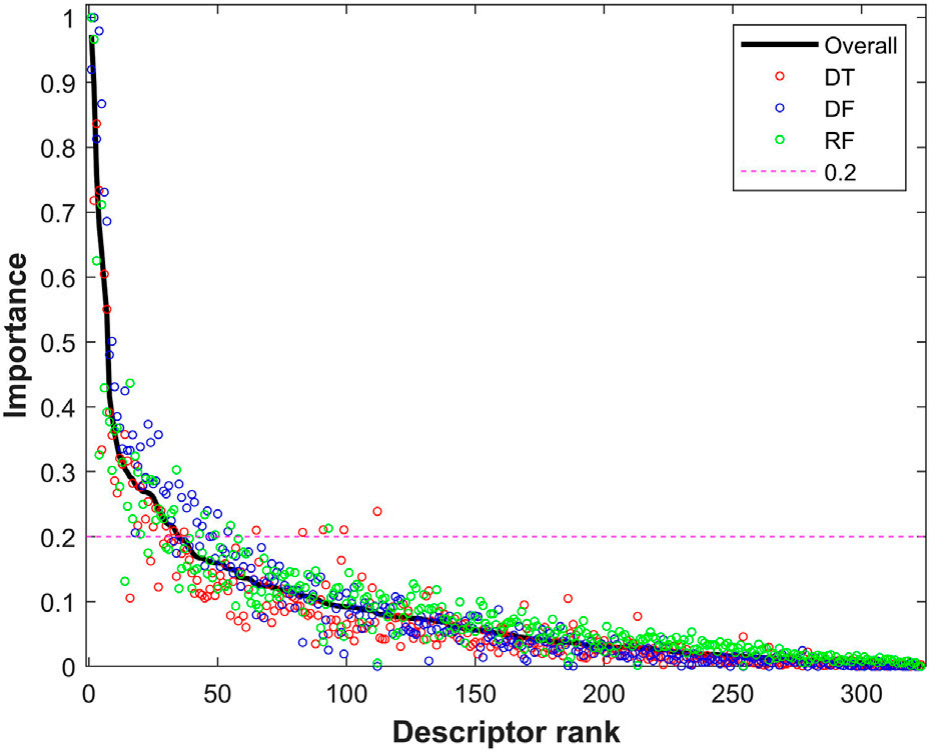
Importance of descriptors from tree-based models. The *x*-axis represents the rank of descriptors, while the *y*-axis indicates importance of descriptors. The importance values from decision tree (DT), decision forest (DF), and random forest (RF) are plotted as red, blue and green circles, respectively. The black solid line represents the combined overall importance. The red dash line marks the threshold of importance for descriptor selection.

The descriptors with Shannon entropy less than 2.5 were discarded, thus 323 descriptors remained in the datasets. Next, 500 iterations of 5-fold cross-validations on the training dataset with the 323 descriptors were conducted using the tree-based machine learning algorithms (DT, DF, and RF). The frequency for each of the 323 descriptors in the cross-validations was examined for each of the three algorithms to calculate an importance value. The calculated importance values of the 323 descriptors for DT, DF, and RF are plotted as red, blue, and green circles in Figure 4, respectively. The average importance values were calculated for the descriptors based on their three individual importance values, and the ranked average importance values are plotted as the black curve in Figure 4. Lastly, 34 descriptors with importance values >0.2 were selected for model development and validation. The selected 34 molecular descriptors are listed in Supplementary Table S3.

### Cross-validations

To estimate the goodness of machine learning models constructed on the training dataset from ToxRefDB, 500 iterations of 5-fold cross-validations were conducted on the training dataset using seven machine learning algorithms DT, DF, RF, kNN, SVM, LDA, and LR. Then, the consensus model predictions were generated based on the predictions of individual models from the cross-validations. For each of the 500 iterations of cross-validations and for each of the eight machine learning algorithms, performance metrics were calculated using Eqs 5–11 by comparing the predictions with the actual rat multigeneration reproductive toxicity assignment (positive or negative).

**FIGURE 5:**
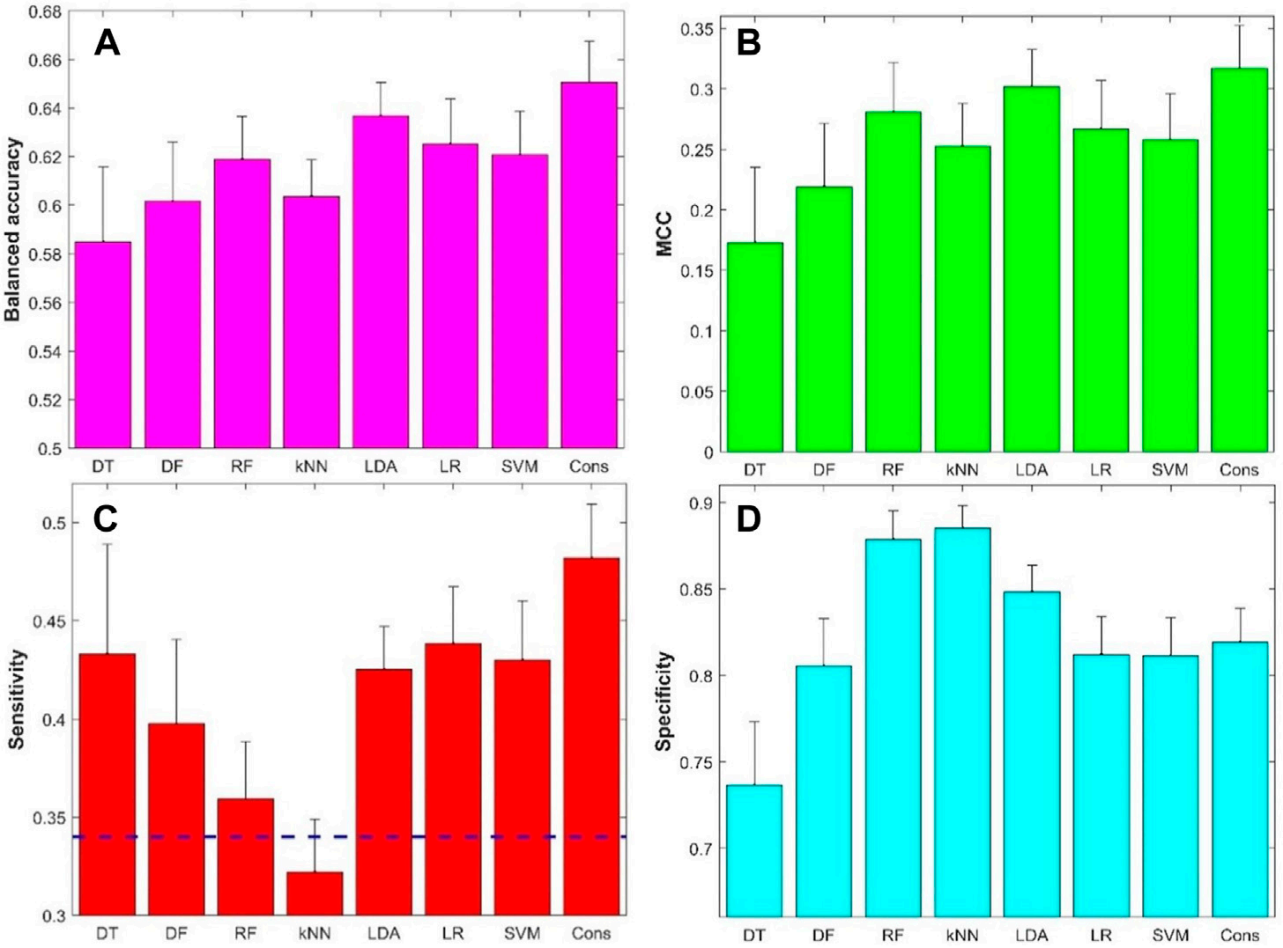
Results of 5-fold cross-validations: **(A)** Balanced accuracy, **(B)** Matthews correlation coefficient (MCC), **(C)** Sensitivity, and **(D)** Specificity. The *x*-axis indicates machine learning models and the *y*-axis represents performance metrics and starts from the performance of hypothetic random model except for sensitivity **(C)** for which the hypothetic random model metrics was marked by the dash line. The average performance metrics from the 500 iterations of 5-fold cross-validation were plotted as bars and the related standard deviations were given as sticks above the bars. The dash line in figure **(C)** represented 0.34, the ratio of positive compounds in all compounds. DT, decision tree; DF, decision forest; RF, random forest; kNN, k-nearest neighbors; LDA, linear discriminant analysis; LR, logistic regression; SVM, support vector machine; Cons, consensus model.

The performance metrics values from the 500 iterations of cross-validations were given in Supplementary Tables S4–S11. The prediction accuracy, positive predictive rate, and negative predictive rate values are summarized in Supplementary Figure S2, while the balanced accuracy, MCC, sensitivity, and specificity values are summarized in Figure 5. The average balanced accuracy (Figure 5A), MCC (Figure 5B), sensitivity (Figure 5C), and specificity (Figure 5D) from the cross-validations are plotted as magenta, green, red, and cyan bars, respectively, for the eight machine learning algorithms indicated at the *x*-axis, while the corresponding standard deviations are shown as sticks above the bars. Examination of Figure 5 found that the models constructed with the eight machine learning algorithms had some predictive power with average balance accuracy greater than 0.5 (0.58–0.65) (Figure 5A) and average MCC greater than 0 (0.17–0.32) (Figure 5B). Furthermore, the model’s performance was very stable with a small standard deviation among the 500 iterations of cross-validations. Not surprisingly, the model performance varied in machine learning algorithms. However, the variation is not large. Moreover, the consensus modeling outperformed the seven individual machine learning algorithms. Another interesting observation is that all machine learning models had lower sensitivity (average values were between 0.32 and 0.48, Figure 5C) than specificity (average values were between 0.74 and 0.89, Figure 5D). It is worth noting that kNN models had lower sensitivity than a hypothetical random model (indicated by the black dash line in Figure 5C), though the kNN models had the highest specificity (Figure 5D). Other machine learning models had a better balance between sensitivity and specificity.

### External validations

**FIGURE 6:**
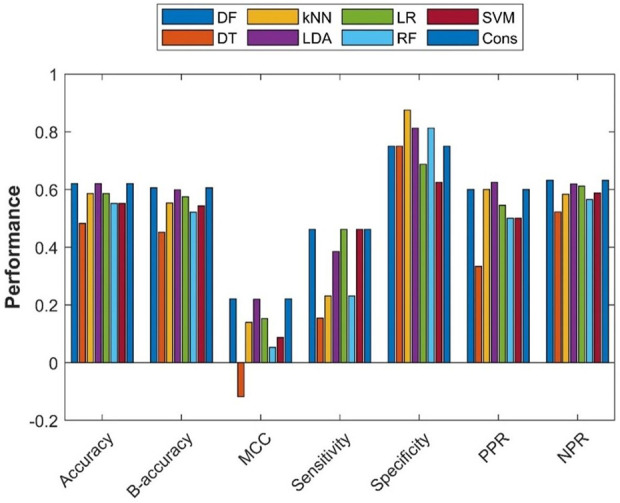
External validation results. The *x*-axis indicates performance metrics, while the *y*-axis gives values of the performance metrics. Machine learning algorithms were color coded by the color legend above the figure.

To assess the extrapolation of machine learning models constructed with the training dataset obtained from ToxRefDB to predicting the potential of rat multigeneration reproductive toxicity of new chemicals, the external validation dataset curated from COSMOS and the literature was used for validation of the machine learning models. First, all 275 chemicals in the training dataset were used to build models using the seven machine learning algorithms. Then, the seven constructed models were used to predict potential rat multigeneration reproductive toxicity of the 29 chemicals in the external validation dataset. Lastly, consensus model predictions on the 29 chemicals were made based on the predictions from the seven individual machine learning models. The performance metrics of these eight models were calculated by comparing the predictions with the actual rat multigeneration reproductive toxicity study results and were plotted as bars in Figure 6. The overall performance of the external validations showed some predictive power with metrics values greater than those of the hypothetic random model (>50% of accuracy, >50% balanced accuracy, and >0 of MCC) for seven machine learning models, but the DT model had <50% of accuracy and balanced accuracy and <0 of MCC. Though the performance variation in the seven models was not large, the DF, LDA, and consensus models outperformed the other four models. Similar to the cross-validations, all eight models had greater specificity than sensitivity, indicating all models performed better on negative chemicals than positive chemicals. However, positive and negative predictions had very similar accuracy [positive predictive rate (PPR) and negative predictive rate (NPR)] for all the models except the DT model which had remarkably lower PPR than NPR.

**FIGURE 7:**
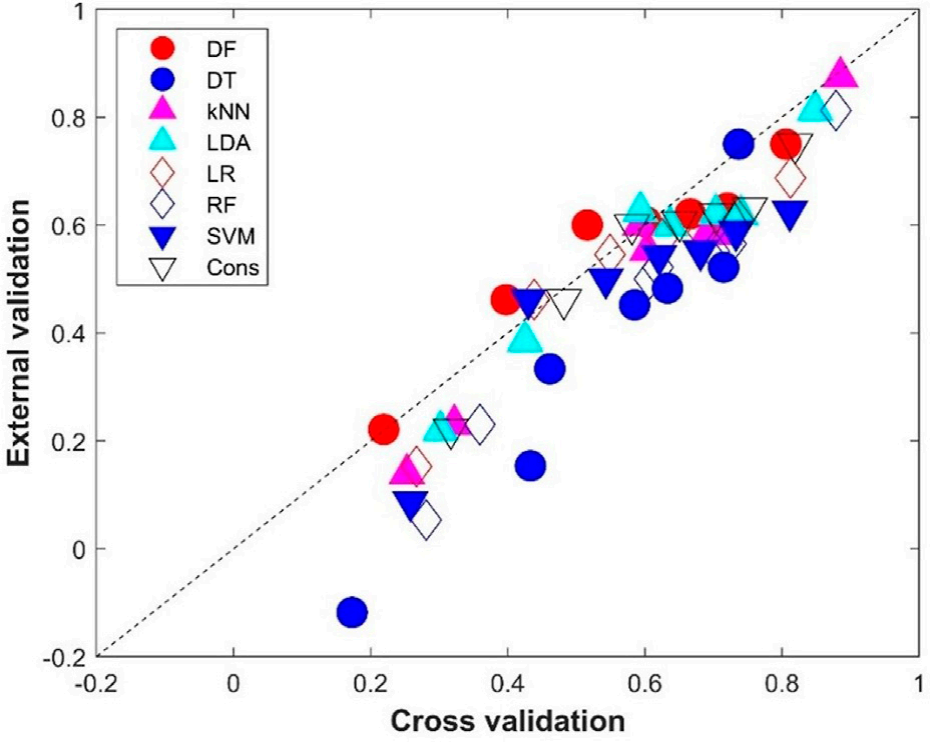
Performance metrics comparison between the cross-validations and external validations. The *x*-axis indicates the average performance metrics from the cross-validations, while the *y*-axis gives the values from the external validations. The diagonal dash line depicts where performance metrics are the same for the cross-validations and external validations. Results from the eight machine learning models are plotted in different shapes and colors given by the legend above the figure.

The comparison between the average performance metrics values from the 500 iterations of cross-validations and the performance metrics values from the external validations is given in Figure 7. Not surprisingly, the external validations showed slightly lower performance than the cross-validations for most of the eight machine learning models as most of the performance metrics are under the diagonal line, which indicates the same performance between the external validations and cross-validations. Interestingly, DF had similar performance in the external validations and in the cross-validations (the red solid circles are close to the diagonal line in Figure 7). It is worth noting that the DT model not only performed the worst in the external validation (Figure 6), but also showed the largest difference from the cross-validations (six of the seven metrics are far down to the diagonal line, the blue solid circles in Figure 7), indicating DT is prone to overfitting.

### Prediction confidence analysis

**FIGURE 8:**
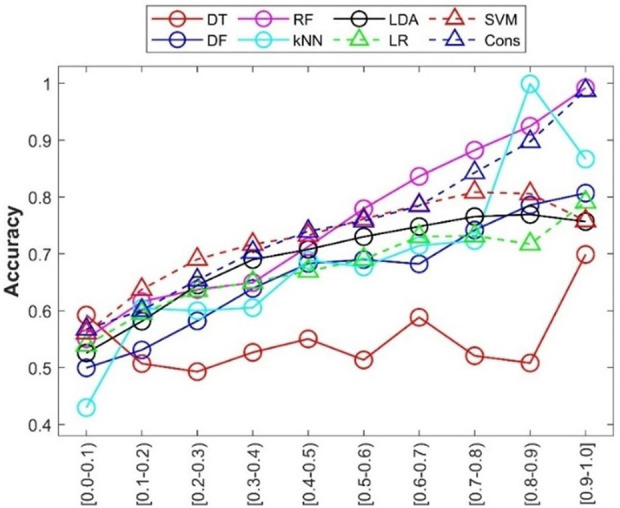
Prediction confidence analysis of the cross-validations. Prediction confidence levels are given at the *x*-axis. The accuracy of predictions at each confidence level is indicated by the *y*-axis. Performance metrics for the eight machine learning models are plotted in different shapes and colors given by the up-left corner legend.

In addition to accuracy of predictions from a machine learning model, prediction confidence from the model is a useful metric for application of the machine learning model. The prediction confidence values were calculated for the predictions from the 500 iterations of 5-fold cross-validations. The accuracies of predictions at different confidence levels were calculated and are given in Figure 8. The relationships between confidence level and other performance metrics were plotted in Supplementary Table S3. Generally, the accuracy of predictions (*y*-axis) increased with increasing prediction confidence levels (*x*-axis), especially for RF and consensus models. Interestingly, the predictions from DT (red circles in Figure 8) at the highest confidence level (0.9–1) showed higher prediction accuracy, while predictions at other confidence levels did not display a similar relationship between prediction accuracy and prediction confidence levels. Further examination of the distribution of prediction confidence values found that the majority of DT predictions had very high prediction confidence (>0.9), and the small numbers of predictions at other confidence levels hardly show a statistically meaningful relationship. The results indicated that prediction confidence provides an additional performance assessment in applications of the developed machine learning models.

### Impact of lowest-observed-adverse effect level on prediction performance

**FIGURE 9:**
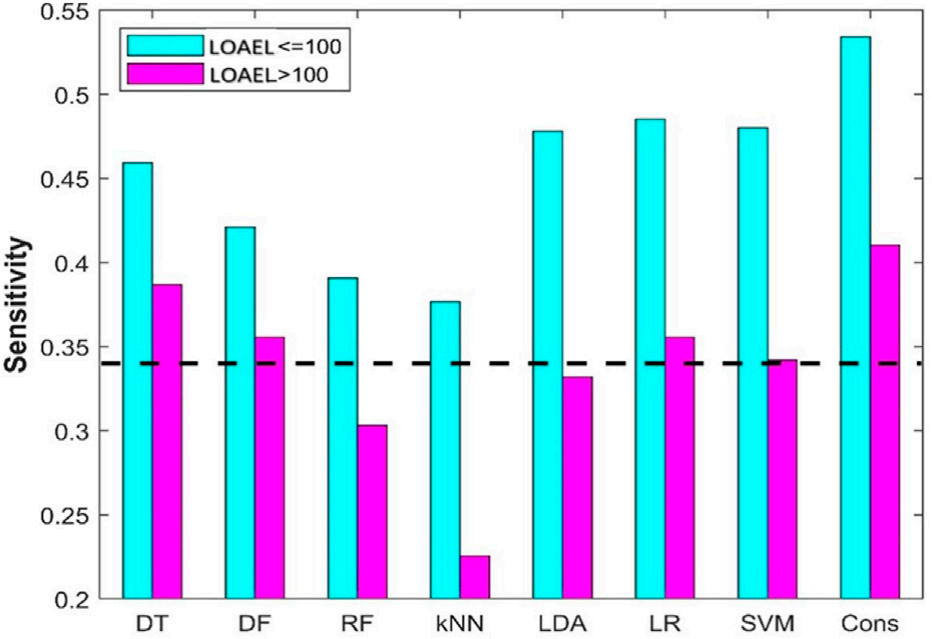
Sensitivity of positive chemicals with different LOAEL. The results from the eight machine models are indicated at the *x*-axis. The *y*-axis gives the prediction sensitivity. The sensitivities are plotted in cyan and pink bars for chemicals with LOAEL <100 mg/kg/day and >100 mg/kg/day, respectively. The black dash line represents the ratio of positive compounds in the training dataset.

In data curation from ToxRefDB, chemicals with LOAEL recorded in any reproductive toxicity endpoints in the rat multigeneration reproductive toxicity studies were assigned as positive chemicals. However, different LOAEL values indicate different activity: the lower a LOAEL value, the stronger the reproductive toxicity observed for the chemical. To examine the difference in performance of the machine learning models on chemicals with strong and weak reproductive toxicity, the 94 positive chemicals were divided into two groups based on their observed LOAEL values: strong chemicals with LOAEL ≤100 mg/kg/day and weak chemicals with LOAEL >100 mg/kg/day. Sensitivities on the strong and weak chemicals from the eight machine learning models are plotted as cyan and magenta bars in Figure 9, respectively. As expected, all eight machine learning models performed better on strong chemicals than on weak chemicals. Furthermore, it was observed that only DT and consensus models showed some predictive power on weak chemicals, and the other six machine learning models had very limited or no predictive power in predicting weak chemicals (close to or lower than the dashed line in Figure 9 for the hypothetical random model), indicating a huge challenge in development of machine learning models for predicting chemicals with weak rat multigeneration reproductive toxicity.

## Discussion

Reproductive toxicity is an important safety concern of compounds. *In vivo* multigeneration reproductive toxicity testing is complicated, time-consuming, expensive, and can require many animals. Therefore, alternative approaches are needed to assist the assessment of multigeneration reproductive toxicity. The existing *in vivo* reproductive toxicity data are invaluable for the development of alternative approaches. In the development of machine learning models as alternative approaches, data quality is crucial for objectively assessing applicability of the developed models. In this study, we focused on predicting the potential reproductive toxicity of chemicals in rat multigeneration reproductive toxicity studies. Therefore, 275 chemicals and their reproductive toxicity data in rat multigeneration reproductive toxicity studies were collected from ToxRefDB and used as the training dataset for constructing the predictive models. A dataset with 29 unique chemicals and their rat multigeneration reproductive toxicity data were curated from the COSMOS database and the literature as the external validation dataset. We constructed seven individual models using machine learning algorithms DT, DF, RF, kNN, SVM, LDA, and LR. In addition, a consensus model was derived based on the seven individual models. The performance of these eight models was evaluated through cross-validations and external validations. Our results showed that machine learning models trained on the data obtained from rat multigeneration reproductive toxicity studies had some predictive power and can be reasonably extrapolated to predict rat multigeneration reproductive toxicity of new chemicals. However, it is necessary to be cautious when applying the developed machine learning models. Model predictions are representative of activity in rat multigeneration reproductive toxicity studies and should not be interpreted as potential activity in other reproductive toxicity studies, such as one-generation, short-term reproductive toxicity testing.

Although the eight machine learning models performed differently in both cross-validations and external validations, the variations in performance of these machine learning models are not large in terms of any of the seven metrics (Figures 5, 6, Supplementary Table S2). Our results suggest that the selection of machine learning algorithms is not a huge concern. However, the consensus model outperformed the seven individual models. Moreover, the consensus models had a better balance between sensitivity and specificity compared to the seven individual models, gaining improvement in sensitivity (Figure 5C and Figure 9). As all models had lower sensitivity than specificity, and identification of potential positive chemicals in rat multigeneration reproductive toxicity studies is the major goal, the improvement in sensitivity of the consensus model is vital in application of such machine learning models. Therefore, consensus modeling based on individual machine learning algorithms is recommended as an alternative approach for predicting potential activity of chemicals in rat multigeneration reproductive toxicity studies.

In development and validation of machine learning models, selection of informative chemical descriptors is crucial to the success of the developed models for predicting new chemicals that are not included in the training. However, information leaking sometimes happens, therefore it is important to avoid information leaking during this process. The information of modeling target (rat multigeneration reproductive toxicity of chemicals in this study) should not be leaked to the selection of molecular descriptors for model building and validation. In this study, the 777 Mold2 descriptors were first filtered by removing descriptors that have the same value zero for >90% of chemicals. The left descriptors were then subjected to Shannon entropy analysis to remove descriptors with low information content (Shannon entropy less than 2.5). At last, the remaining molecular descriptors were filtered by the frequency used in the construction of tree-based models. During the whole process, the modeling target (rat multigeneration reproductive toxicity) data were not used. No information leaking happened in the selection of molecular descriptors, so the performance validation results should be fair and realistic.

One important process in machine learning model development is to determine a set of appropriate algorithmic parameters for the machine learning algorithm. Each machine learning algorithm has its own algorithmic parameters, which are not only algorithm dependent but also training data related. Thus, a universal set of parameters for an algorithm suitable for all datasets is not realistic. Appropriate algorithmic parameters need to be determined before constructing a machine learning model using the training data. However, it is critical to avoid information leaking where the whole training dataset is used for algorithmic parameters determination. Therefore, an inner 5-fold cross-validation was used to optimize the parameters for the seven machine learning algorithms. More specifically, before constructing a model on a training dataset using a machine learning algorithm, a set of algorithmic parameters were fixed. The whole training dataset was randomly divided into five folds to conduct a 5-fold cross-validation using the fixed parameters and measure the performance. This process was repeated five times with random division of the whole training dataset into five folds using different random seeds. The average performance from the five iterations of inner 5-fold cross-validations were calculated to measure the performance with the fixed set of algorithmic parameters. The parameters were then repeatedly changed to conduct the inner 5-fold cross-validations. At last, the set of parameters that resulted in the best performance were used to construct a model using the whole training dataset. As the training dataset is not balanced between positive and negative chemicals (94 positive and 181 negative), the overall prediction accuracy is not a good metric for assessing model performance, as it is biased toward the majority (negative). The commonly used balanced accuracy in statistics favors the minority (positive) too much, and thus is not a suitable metric for comparing models. Therefore, MCC was used as the performance metric to determine the parameters that resulted in the best performed model since MCC is more reasonable than accuracy and balanced accuracy in balancing positive and negative chemicals for performance measurement (Chicco et al., 2021).

**FIGURE 10:**
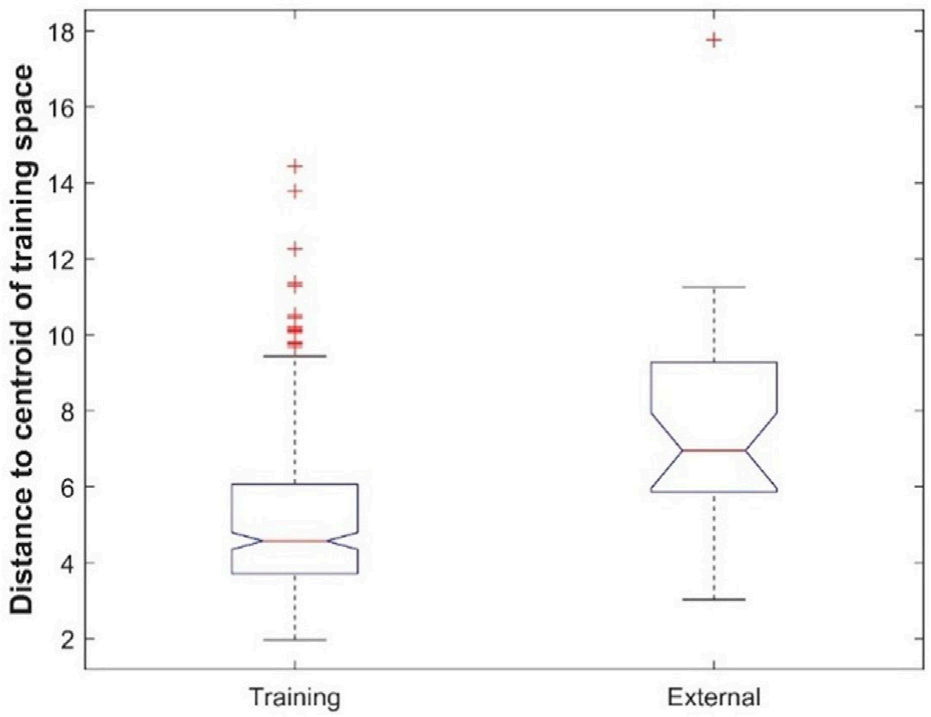
Boxplot of distances to the centroid of training chemicals for the training dataset and external validation dataset.

Comparison of prediction performance between the cross-validations and external validations showed that, except for the DF model, all machine learning models had better performance in cross-validations than in external validations (Figure 7). To ascertain the potential cause for the differences in performance, the chemical spaces of the training dataset and the external validation dataset are compared in Figure 10. The principal component (PC) analysis showed some differences between the training chemicals and the external validation chemicals (Supplementary Table S3). However, because the first two PCs only cover 30% of the variance of the chemicals, it is difficult to see the clear difference between the training chemicals and the external chemicals as well as among different categories of the external chemicals. The first three PCs cover about 38% of the variance as shown in Supplementary Figure S5 which gives the variance covered by all PCs. It is also difficult to see the clear difference in a 3-dimensional space of the first three PCs. The first 20 PCs cover 95% of the variance as shown by the red dashed line in Supplementary Figure S5. Therefore, we used the 20-dimensional space with the first 20 PCs to represent the training space and used the distance to the centroid of training chemicals in the 20-dimensional space as applicability domain measurement. The distances to the centroid of the chemicals in the training dataset are significantly larger for the external validation chemicals than for the training chemicals. Therefore, the models constructed from the training dataset lost some predictive power in predicting the external validation chemicals compared to predicting the training chemicals. The performance difference can also be indicated by the difference in chemical categories between the training dataset and the external validation dataset (Table 1). The majority of the training chemicals (243 of 275) are pesticides, while most of the external validation chemicals are cosmetics and drugs. Close look up at the external validation chemicals indicates the role of applicability domain. Because only one pesticide and two research chemicals are in the external validation set, they are not statistically representative for the categories. Therefore, we excluded them in the comparative analysis. As it can be seen form Supplementary Figure S6, 22, 15, and 11 of the 26 external chemicals have larger distances to the centroid than >50%, 80%, and 90% of the training chemicals. The 11 external chemicals far from the training space (the centroid) include one drug (4-methylimidazole), seven cosmetics [3-(dimethylamino)propylamine, 1,4-cyclohexanedimethanol, 2-butanol, butylated hydroxytoluene, 1,3-butanediol, octadecyl 2-hydroxy-1,2,3-propanetricarboxylate, benzoic acid], and three food additives (acetone peroxide, 2,2-dibromo-3-nitrilopropionamide, azodicarbonamide) which are not well represented in the training data set. For example, 4-methylimidazole has a distance to the centroid 9.751 (Supplementary Figure S6) which is larger than the distances of 95% training chemicals. It showed toxicity in the rat multigeneration reproductive toxicity study. However, among the eight machine learning models, only the decision forest correctly predicted as positive. Our results suggest that caution should be taken in applying machine learning models to chemicals that are dissimilar to training chemicals.

The results of prediction confidence analysis showed a general trend that predictions at a higher confidence level were more accurate than prediction at a lower confidence level. Therefore, prediction confidence provides an additional metric for appropriate application of machine learning models. Furthermore, distribution of predictions at confidence levels also needs to be examined when assessing a machine learning model. It is expected that a good model not only has high accuracy for high confidence predictions, but also does not predict many chemicals at low confidence. The distributions of predictions in the cross-validations at different confidence levels are plotted in Supplementary Figure S7 for the eight machine learning models. Predictions at different confidence levels are similar, indicating the previous observations in confidence analysis are meaningful and not impacted by extremely few chemicals at some confidence levels. It is worth noting that the RF model and consensus model have fewer predictions at very high confidence >0.75 than the other confidence levels, although both models had very high prediction accuracy at high confidence levels (Figure 8). Our results indicate that prediction confidence analysis should examine both accuracy and distribution of predictions at confidence levels.

The rat multigeneration reproductive toxicity studies in ToxRefDB reported diverse reproductive toxicity observations and a wide range of LOAEL values. The positive chemicals displayed quite different toxicity levels that might be caused through different mechanisms. All the machine learning models, especially the consensus model, performed differently on the chemicals with stronger reproductive toxicity (smaller LOAEL) and with weaker toxicity (larger LOAEL): predictions on the chemicals with smaller LOAEL are more accurate than on the chemicals with larger LOAEL (Figure 9). Our speculation is that chemicals with strong reproductive toxicity may have more distinct structural features and cause toxicity through more similar mechanisms. However, pinpointing the mechanisms of the diverse reproductive toxicity observations in the rat multigeneration reproductive toxicity studies and ascertaining the associated structural features are important, but very challenging with current experimental data and deserving of further investigation.

Although the models constructed showed some predictive power on potential activity of chemicals in rat multigeneration reproductive toxicity studies, the datasets used and models developed in this study have some limitations. First, due to the diverse reproductive toxicity endpoints observed in a rat multigeneration reproductive toxicity study and, thus, different mechanisms possibly associated with the observed toxicity endpoints, the number of chemicals with experimental data for model development is relatively small, resulting in obstructions in development of highly performing machine learning models. With more chemicals tested in rat multigeneration reproductive toxicity studies in the future, the performance of machine learning models could be improved. Second, although our results demonstrated that consensus modeling through combining individual machine learning models could improve overall prediction accuracy, especially sensitivity, the sensitivity from all machine learning models, including the consensus model, were much lower compared to the specificity. This disparity between sensitivity and specificity was likely caused by the disproportion between the positive and negative chemicals in the training dataset and should be improved. One solution is to test more chemicals in the experiment. Another solution is to computationally integrate strategies for balancing positive and negative chemicals in machine learning model development. Third, only rat testing data were curated for development and validation of the machine learning models. Therefore, applications of the developed machine learning models to prediction of multigeneration reproductive toxicity in other species should be cautious.

## Conclusion

In this study, machine learning models for rat multigeneration reproductive toxicity prediction were developed using seven machine learning algorithms. The developed models were evaluated by 5-fold cross-validations and external validations. These models demonstrated some predictive power for predicting potential activity of chemicals in rat multigeneration reproductive toxicity studies. Our results indicate that machine learning algorithms do not dramatically impact performance of the developed models. However, consensus modeling based on individual machine learning algorithms improved model performance, especially sensitivity, recommending consensus modeling as a good practice in applying machine learning to predict rat multigeneration reproductive toxicity. The prediction confidence derived from machine learning models correlated with prediction accuracy, providing an additional metric in applications of the developed machine learning models. Here, we demonstrate the importance of consensus models for building increased confidence in machine learning methods. Though the prediction accuracy needs improvement due to the complicated mechanisms of reproductive toxicity observed in rat multigeneration reproductive toxicity studies, our findings shed light on exploring machine learning models as alternative methods for *in vivo* rat multigeneration reproductive toxicity testing, and the developed models could be used in screening chemicals for experimental testing of reproductive toxicity. While direct application of the current model to risk assessment may be limited until further development, the model could be used in a battery of other in silico and/or *in vitro* programs for screening chemicals. As part of a new chemical development program, our model could result in cost savings by screening out strong reproductive toxicants, eliminating the need for additional *in vivo* testing.

## Data Availability

The original contributions presented in the study are included in the article/Supplementary Material, further inquiries can be directed to the corresponding author.
