# Supplementary material for: Machine learning models for rat multigeneration reproductive toxicity prediction
Source: Front Pharmacol. 2022 Sep 27;13:1018226. doi: 10.3389/fphar.2022.1018226 (PMC9552001; doi:10.3389/fphar.2022.1018226)
Supplement: Supplementary file 1 [file DataSheet1.PDF]

Machine Learning Models for Multigeneration Reproductive Toxicity Prediction

Jie Liu[1], Wenjing Guo[1], Fan Dong[1], Jason Aungst[2], Suzanne Fitzpatrick[2], Tucker A Patterson[1], Huixiao Hong[1*]

[1] National Center for Toxicological Research, U.S. Food and Drug Administration, 3900 NCTR Road, Jefferson, AR 72079, USA

[2] Center for Food Safety and Applied Nutrition, U.S. Food and Drug Administration, 5001 Campus Drive, College Park, MD 20740, USA

Correspondence: huixiao.hong@fda.hhs.gov

Disclaimer: This article reflects the views of the authors and does not necessarily reflect those of the U.S. Food and Drug Administration.

Table S1. Chemical list of training data 3
Table S2. Chemical list of external data 9
Table S3. Molecular descriptors identified for the model development 10
Table S4. Performance of 5-fold cross-validations from decision tree (DT).............................. 11
Table S5. Performance of 5-fold cross-validations from decision forest (DF)........................... 20
Table S6. Performance of 5-fold cross-validations from random forest (RF) 29
Table S7. Performance of 5-fold cross-validations from k-nearest neighbors (kNN) 38
Table S8. Performance of 5-fold cross-validations from support vector machine (SVM) 47
Table S9. Performance of 5-fold cross-validations from linear discriminant analysis (LDA) 56
Table S10. Performance of 5-fold cross-validations from logistic regression (LR) 65
Table S11. Performance of 5-fold cross-validations from consensus modeling (Cons)............. 74
Figure S1. Performance of consensus models with different model voting 83
Figure S2. Average performance of 5-fold cross-validation.. 84
Figure S3. Prediction confidence analysis 85
Figure S4. Scatter plot of chemical in principal conponent analysis…………..………………..86
Figure S5. Distribution of variance coverage of principal conponents…………..………………87
Figure S6. Applicability domain analysis…………..…………………..………..……..……….88
Figure S7. Distribution of predictions in prediction confidence analysis 89
Python codes for Decision Tree (DT)…………..……..…………………………………………..90
Python codes for Random Forest (RF)…...……..…………………………………………..95
Python codes for k-Nearest Neighbor (k-NN)…………..……..…………………………..……..99
Python codes for Support Vector Machine (SVM)…………..……..…………………………..103
Python codes for Linear Discriminant Analysis (LDA)…………..…………………………..108
Python codes for Logistic Regression (LR)…………..……..…………………………………..112
Matlab codes for Decision Forest (DF)…………..…………………………………………...116

Table S1. Chemical list of training data.

Chemical_name	CASRN	Reproductive toxicity*
Prohexadione-calcium	127277-53-6	0
Prallethrin	23031-36-9	0
Pyriproxyfen	95737-68-1	0
Spirodiclofen	148477-71-8	1
Trifloxysulfuron-sodium	199119-58-9	0
Cymoxanil	57966-95-7	0
Tebuthiuron	34014-18-1	0
Pyrimethanil	53112-28-0	0
Methoxyfenozide	161050-58-4	0
5,5-Dimethylhydantoin	77-71-4	0
Fenamiphos	22224-92-6	0
Glufosinate-ammonium	77182-82-2	0
Diphenylamine	122-39-4	1
Triflumizole	68694-11-1	1
Chlorpropham	101-21-3	1
Resmethrin	10453-86-8	1
Pirimicarb	23103-98-2	0
Bentazone	25057-89-0	0
Clodinafop-propargyl	105512-06-9	0
Mevinphos	7786-34-7	1
Thiophanate-methyl	23564-05-8	0
Cyfluthrin	68359-37-5	0
Tepraloxydim	149979-41-9	0
Clofencet	82697-71-0	0
Phosalone	2310-17-0	0
Carfentrazone-ethyl	128639-02-1	0
Cyazofamid	120116-88-3	0
Flucarbazone-sodium	181274-17-9	1
Thiobencarb	28249-77-6	0
Ethephon	16672-87-0	0
Cyhexatin	13121-70-5	1
Fenarimol	60168-88-9	1
Butyl benzyl phthalate	85-68-7	0
Bifenazate	149877-41-8	0
Sulfluramid	4151-50-2	0
Etridiazole	2593-15-9	0
Ethametsulfuron methyl	97780-06-8	0
Endosulfan	115-29-7	1
Quinoxyfen	124495-18-7	0
2-Phenylphenol	90-43-7	0
Fenthion	55-38-9	1
Chlorpyrifos-methyl	5598-13-0	0
Penoxsulam	219714-96-2	0
Orthosulfamuron	213464-77-8	0
N,N-Diethyl-3-methylbenzamide	134-62-3	0
Pyraclostrobin	175013-18-0	0
Propanil	709-98-8	1
Flumioxazin	103361-09-7	1

Acibenzolar-S-methyl	135158-54-2	0
Methamidophos	10265-92-6	0
Cyproconazole	94361-06-5	0
Thiamethoxam	153719-23-4	1
Ziram	137-30-4	0
Pyridaben	96489-71-3	0
Benzophenone	119-61-9	1
Malathion	121-75-5	0
Methylene bis(thiocyanate)	6317-18-6	0
Triadimenol	55219-65-3	1
Imazethapyr	81335-77-5	0
Diuron	330-54-1	0
Butafenacil	134605-64-4	1
Mepiquat chloride	24307-26-4	0
2-Methyl-4-chlorophenoxyacetic acid	94-74-6	1
3-Iodo-2-propynyl-N-butylcarbamate	55406-53-6	1
Maneb	12427-38-2	0
Pirimiphos-methyl	29232-93-7	0
Metsulfuron-methyl	74223-64-6	0
Iprodione	36734-19-7	0
Propargite	2312-35-8	0
Propachlor	1918-16-7	0
Propoxycarbazone-sodium	181274-15-7	1
Flumetralin	62924-70-3	0
Diethyl phthalate	84-66-2	1
Fluoxastrobin	361377-29-9	0
Diflufenzopyr	109293-97-2	1
Thiodicarb	59669-26-0	0
Clomazone	81777-89-1	0
Fenhexamid	126833-17-8	0
Cypermethrin	52315-07-8	0
Pendimethalin	40487-42-1	1
Dithiopyr	97886-45-8	0
Imazalil	35554-44-0	1
Flufenpyr-ethyl	188489-07-8	0
Fenamidone	161326-34-7	0
Azinphos-methyl	86-50-0	0
Benomyl	17804-35-2	1
Metconazole	125116-23-6	1
Rotenone	83-79-4	1
Tetraconazole	112281-77-3	1
Famoxadone	131807-57-3	0
Glyphosate	1071-83-6	0
Imazapic	104098-48-8	0
Etoxazole	153233-91-1	0
Aldicarb	116-06-3	0
Molinate	2212-67-1	1
Tetramethrin	7696-12-0	0
Lactofen	77501-63-4	1
MGK-264	113-48-4	0

Clopyralid	1702-17-6	0
Trifloxystrobin	141517-21-7	0
Oryzalin	19044-88-3	0
Chlorthal-dimethyl	1861-32-1	0
Tebufenpyrad	119168-77-3	0
Topramezone	210631-68-8	0
Prosulfuron	94125-34-5	0
Fluvalinate	69409-94-5	0
Dicloran	99-30-9	1
Myclobutanil	88671-89-0	1
Trifluralin	1582-09-8	0
Lindane	58-89-9	0
2,4-Dichlorophenoxyacetic acid	94-75-7	1
Difenoconazole	119446-68-3	0
Daminozide	1596-84-5	1
Pymetrozine	123312-89-0	0
Metam-sodium	137-42-8	0
Acifluorfen, sodium	62476-59-9	0
Metaflumizone	139968-49-3	0
Propazine	139-40-2	0
S-Bioallethrin	28434-00-6	0
Fenbuconazole	114369-43-6	1
Folpet	133-07-3	1
Diclofop-methyl	51338-27-3	1
Bispyribac-sodium	125401-92-5	0
Acequinocyl	57960-19-7	0
Ethoprop	13194-48-4	0
Aminopyralid	150114-71-9	0
Cyprodinil	121552-61-2	0
4-Nitrotoluene	99-99-0	0
Dicyclohexyl phthalate	84-61-7	1
Isoxaben	82558-50-7	1
Pyrithiobac-sodium	123343-16-8	0
Triazamate	112143-82-5	0
Boscalid	188425-85-6	0
Dichlorvos	62-73-7	1
Ethofumesate	26225-79-6	0
Desmedipham	13684-56-5	0
Butylate	2008-41-5	0
Quinclorac	84087-01-4	0
Sulfosulfuron	141776-32-1	0
Atrazine	1912-24-9	0
Sulfentrazone	122836-35-5	1
Dimethoate	60-51-5	1
Coumaphos	56-72-4	0
Thiazopyr	117718-60-2	0
Formetanate hydrochloride	23422-53-9	0
Bensulide	741-58-2	0
Chlorothalonil	1897-45-6	0
Dimethylarsinic acid	75-60-5	1

Propyzamide	23950-58-5	1
Cyanamide	420-04-2	0
Flumiclorac-pentyl	87546-18-7	0
Prometryn	7287-19-6	0
Flutolanil	66332-96-5	0
Fipronil	120068-37-3	1
Oxamyl	23135-22-0	0
Imazamox	114311-32-9	0
Paclobutrazol	76738-62-0	0
Triclopyr	55335-06-3	0
Bendiocarb	22781-23-3	0
Fluthiacet-methyl	117337-19-6	0
Fenpropathrin	39515-41-8	0
Diquat dibromide	85-00-7	0
Permethrin	52645-53-1	0
Dazomet	533-74-4	0
Chloridazon	1698-60-8	0
Nitrapyrin	1929-82-4	0
Propamocarb hydrochloride	25606-41-1	1
Tralkoxydim	87820-88-0	0
Mesosulfuron-methyl	208465-21-8	0
Metobenzuron	111578-32-6	1
Chlorfenapyr	122453-73-0	0
Metribuzin	21087-64-9	0
Dicrotophos	141-66-2	1
Cycloate	1134-23-2	1
Hexazinone	51235-04-2	0
Vinclozolin	50471-44-8	1
Acephate	30560-19-1	1
Dimethenamid	87674-68-8	0
Dicamba	1918-00-9	0
Triclosan	3380-34-5	0
Imazapyr	81334-34-1	0
Cyclanilide	113136-77-9	0
Butachlor	23184-66-9	0
Fluroxypyr	69377-81-7	0
Thidiazuron	51707-55-2	0
Iodosulfuron-methyl-sodium	144550-36-7	1
Tributyltin chloride	1461-22-9	1
Bromuconazole	116255-48-2	0
Prometon	1610-18-0	0
Simazine	122-34-9	0
Chlorethoxyfos	54593-83-8	0
Dicofol	115-32-2	1
Rimsulfuron	122931-48-0	0
S-Ethyl dipropylthiocarbamate	759-94-4	0
Bromacil	314-40-9	0
Imidacloprid	138261-41-3	0
Metolachlor	51218-45-2	0
Ametryn	834-12-8	0

Azafenidin	68049-83-2	1
Dipropyl pyridine-2,5-dicarboxylate	136-45-8	0
Isoxaflutole	141112-29-0	0
Kasugamycin	6980-18-3	1
Trichlorfon	52-68-6	1
Fluazinam	79622-59-6	1
Metasystox R	301-12-2	1
Tri-allate	2303-17-5	1
Captafol	2425-06-1	0
Linuron	330-55-2	1
Oxyfluorfen	42874-03-3	1
Naled	300-76-5	1
Metalaxyl	57837-19-1	0
Sodium pyrithione	15922-78-8	1
Triticonazole	131983-72-7	1
Fludioxonil	131341-86-1	0
Carbaryl	63-25-2	1
Buprofezin	69327-76-0	0
(+-)-Indoxacarb	144171-61-9	0
Ethalfluralin	55283-68-6	0
Tebufenozide	112410-23-8	0
Dinotefuran	165252-70-0	1
Dichlobenil	1194-65-6	0
Butylbenzene	104-51-8	1
Tribenuron-methyl	101200-48-0	0
Copper-8-hydroxyquinoline	10380-28-6	1
Novaluron	116714-46-6	1
Hydramethylnon	67485-29-4	1
Hexaconazole	79983-71-4	1
Triforine	26644-46-2	1
Primisulfuron-methyl	86209-51-0	1
Thiacloprid	111988-49-9	1
Acetamiprid	135410-20-7	1
Bifenthrin	82657-04-3	0
Methomyl	16752-77-5	0
Propiconazole	60207-90-1	1
Benfluralin	1861-40-1	1
Propetamphos	31218-83-4	1
Norflurazon	27314-13-2	1
Thiram	137-26-8	0
Halosulfuron-methyl	100784-20-1	0
Azoxystrobin	131860-33-8	0
Mesotrione	104206-82-8	1
Zoxamide	156052-68-5	0
4-(2,4-Dichlorophenoxy)butyric acid	94-82-6	1
Forchlorfenuron	68157-60-8	1
Fenpyroximate (Z,E)	111812-58-9	0
Isofenphos	25311-71-1	1
Phorate	298-02-2	0
Acetochlor	34256-82-1	0

Azamethiphos	35575-96-3	1
Diazinon	333-41-5	1
Methyl parathion	298-00-0	0
Piperonyl butoxide	51-03-6	0
Oxasulfuron	144651-06-9	1
Triadimefon	43121-43-3	1
Cyhalofop-butyl	122008-85-9	0
Fosthiazate	98886-44-3	0
Epoxiconazole	106325-08-0	1
Clothianidin	210880-92-5	1
Fenbutatin Oxide	13356-08-6	0
Amitraz	33089-61-1	1
Fluazifop-butyl	69806-50-4	1
Picloram	1918-02-1	0
Thiabendazole	148-79-8	0
Uniconazole-P	83657-17-4	0
2-(Thiocyanomethylthio)benzothiazole	21564-17-0	0
2,4-Dichlorophenol	120-83-2	1
Pentachloronitrobenzene	82-68-8	0
Triphenyltin hydroxide	76-87-9	1
Flufenacet	142459-58-3	0
Cyromazine	66215-27-8	0
Metaldehyde	108-62-3	0
Triasulfuron	82097-50-5	0
Fenoxaprop-ethyl	66441-23-4	0
1,3-Dichloro-5,5-dimethylhydantoin	118-52-5	0
7-Oxabicyclo(2.2.1)heptane-2,3-dicarboxylic acid, disodium salt	129-67-9	0

* Reproductive toxicity: 1 - positive and 0 - negative.

Table S2. Chemical list of external data.

Chemical_name	CASRN	Reproductive toxicity*
sodium molybdate dihydrate	10102-40-6	0
2,2-Dibromo-3-nitrilopropionamide	10222-01-2	1
1,4-Cyclohexanedimethanol	105-08-8	1
Caprolactam	105-60-2	0
1,3-Butanediol	107-88-0	1
3-(Dimethylamino)propylamine	109-55-7	0
2E,4E-Hexadienoic acid	110-44-1	1
Azodicarbonamide	123-77-3	0
Butylated hydroxytoluene	128-37-0	0
Acetone peroxide	1336-17-0	0
Octadecyl 2-hydroxy-1,2,3-propanetricarboxylate	1337-33-3	0
Carmine aluminum lake	1390-65-4	0
ibuprofen	15687-27-1	1
2,3,7,8-tetrachlorodibenzo-p-dioxin	1746-01-6	1
D&C Red No. 33	3567-66-6	0
betamethasone	378-44-9	1
Sodium (2-pyridylthio)-N-oxide	3811-73-2	1
Genistein	446-72-0	1
Curcumin	458-37-7	0
Benzo(a)pyrene	50-32-8	1
Bronopol	52-51-7	1
Acesulfame potassium	55589-62-3	0
Caffeine	58-08-2	0
Benzoic acid	65-85-0	0
2-Butanol	78-92-2	1
4-methylimidazole	822-36-6	1
Dibutyl phthalate	84-74-2	0
Butylparaben	94-26-8	0
Eugenol	97-53-0	0

* Reproductive toxicity: 1 - positive and 0 - negative.

Table S3. Molecular descriptors identified for the model development.

Mold2 ID	Descriptor Description
D133	mean value of atomic composition index
D247	EXP2 of Path-distance / Walk-distance over all atoms
D279	total information content order-4 index
D283	structural information content order-2 index
D448	Geary topological structure autocorrelation length-2 weighted by atomic masses
D449	Geary topological structure autocorrelation length-3 weighted by atomic masses
D450	Geary topological structure autocorrelation length-4 weighted by atomic masses
D451	Geary topological structure autocorrelation length-5 weighted by atomic masses
D452	Geary topological structure autocorrelation length-6 weighted by atomic masses
D455	Geary topological structure autocorrelation length-1 weighted by atomic van der Waals volumes
D457	Geary topological structure autocorrelation length-3 weighted by atomic van der Waals volumes
D458	Geary topological structure autocorrelation length-4 weighted by atomic van der Waals volumes
D459	Geary topological structure autocorrelation length-5 weighted by atomic van der Waals volumes
D464	Geary topological structure autocorrelation length-2 weighted by atomic Sanderson electronegativities
D465	Geary topological structure autocorrelation length-3 weighted by atomic Sanderson electronegativities
D466	Geary topological structure autocorrelation length-4 weighted by atomic Sanderson electronegativities
D467	Geary topological structure autocorrelation length-5 weighted by atomic Sanderson electronegativities
D471	Geary topological structure autocorrelation length-1 weighted by atomic polarizabilities
D486	Moran topological structure autocorrelation length-8 weighted by atomic masses
D487	Moran topological structure autocorrelation length-1 weighted by atomic van der Waals volumes
D488	Moran topological structure autocorrelation length-2 weighted by atomic van der Waals volumes
D489	Moran topological structure autocorrelation length-3 weighted by atomic van der Waals volumes
D491	Moran topological structure autocorrelation length-5 weighted by atomic van der Waals volumes
D493	Moran topological structure autocorrelation length-7 weighted by atomic van der Waals volumes
D495	Moran topological structure autocorrelation length-1 weighted by atomic Sanderson electronegativities
D497	Moran topological structure autocorrelation length-3 weighted by atomic Sanderson electronegativities
D498	Moran topological structure autocorrelation length-4 weighted by atomic Sanderson electronegativities
D499	Moran topological structure autocorrelation length-5 weighted by atomic Sanderson electronegativities
D501	Moran topological structure autocorrelation length-7 weighted by atomic Sanderson electronegativities
D504	Moran topological structure autocorrelation length-2 weighted by atomic polarizabilities
D505	Moran topological structure autocorrelation length-3 weighted by atomic polarizabilities
D506	Moran topological structure autocorrelation length-4 weighted by atomic polarizabilities
D507	Moran topological structure autocorrelation length-5 weighted by atomic polarizabilities
D510	Moran topological structure autocorrelation length-8 weighted by atomic polarizabilities

Table S4. Performance of 5-fold cross-validations from decision tree (DT). Performance of 5-fold cross-validations were evaluated by seven performance metrics. Each row shows the performance of one time 5-fold cross-validation. 5-fold cross-validation was repeated 500 times (CV-1 ~ CV-500).

Cross-validation	Accuracy	Sensitivity	Specificity	Balanced accuracy	Positive prediction rate	Negative prediction rate	Matthews correlation coefficient
CV-1	0.6655	0.5957	0.7017	0.6487	0.5091	0.7697	0.2879
CV-2	0.6000	0.3298	0.7403	0.5351	0.3974	0.6802	0.0738
CV-3	0.6836	0.5532	0.7514	0.6523	0.5361	0.7640	0.3023
CV-4	0.5927	0.4255	0.6796	0.5525	0.4082	0.6949	0.1041
CV-5	0.6218	0.4787	0.6961	0.5874	0.4500	0.7200	0.1724
CV-6	0.6582	0.5106	0.7348	0.6227	0.5000	0.7430	0.2442
CV-7	0.6036	0.3404	0.7403	0.5404	0.4051	0.6837	0.0847
CV-8	0.6218	0.4681	0.7017	0.5849	0.4490	0.7175	0.1681
CV-9	0.6327	0.5213	0.6906	0.6059	0.4667	0.7353	0.2069
CV-10	0.6145	0.3830	0.7348	0.5589	0.4286	0.6963	0.1213
CV-11	0.6327	0.3617	0.7735	0.5676	0.4533	0.7000	0.1440
CV-12	0.6582	0.4468	0.7680	0.6074	0.5000	0.7277	0.2212
CV-13	0.6545	0.4787	0.7459	0.6123	0.4945	0.7337	0.2264
CV-14	0.6436	0.3617	0.7901	0.5759	0.4722	0.7044	0.1637
CV-15	0.5673	0.3830	0.6630	0.5230	0.3711	0.6742	0.0456
CV-16	0.6000	0.3404	0.7348	0.5376	0.4000	0.6821	0.0786
CV-17	0.5891	0.3830	0.6961	0.5396	0.3956	0.6848	0.0797
CV-18	0.6364	0.3723	0.7735	0.5729	0.4605	0.7035	0.1547
CV-19	0.6400	0.4043	0.7624	0.5833	0.4691	0.7113	0.1734
CV-20	0.6727	0.4787	0.7735	0.6261	0.5233	0.7407	0.2580
CV-21	0.6582	0.5319	0.7238	0.6278	0.5000	0.7486	0.2521
CV-22	0.6073	0.4574	0.6851	0.5713	0.4300	0.7086	0.1405
CV-23	0.6000	0.4681	0.6685	0.5683	0.4231	0.7076	0.1336
CV-24	0.5709	0.3511	0.6851	0.5181	0.3667	0.6703	0.0365
CV-25	0.6618	0.4894	0.7514	0.6204	0.5055	0.7391	0.2427
CV-26	0.6473	0.4468	0.7514	0.5991	0.4828	0.7234	0.2021
CV-27	0.6182	0.4043	0.7293	0.5668	0.4368	0.7021	0.1362
CV-28	0.6218	0.4149	0.7293	0.5721	0.4432	0.7059	0.1466
CV-29	0.6291	0.4681	0.7127	0.5904	0.4583	0.7207	0.1799
CV-30	0.6327	0.4681	0.7182	0.5932	0.4632	0.7222	0.1858
CV-31	0.6473	0.4787	0.7348	0.6068	0.4839	0.7308	0.2141
CV-32	0.6145	0.4043	0.7238	0.5640	0.4318	0.7005	0.1302
CV-33	0.5673	0.3936	0.6575	0.5255	0.3737	0.6761	0.0505
CV-34	0.6255	0.4787	0.7017	0.5902	0.4545	0.7216	0.1782
CV-35	0.6182	0.4043	0.7293	0.5668	0.4368	0.7021	0.1362
CV-36	0.6509	0.4787	0.7403	0.6095	0.4891	0.7322	0.2202
CV-37	0.6509	0.4362	0.7624	0.5993	0.4881	0.7225	0.2045
CV-38	0.6327	0.4255	0.7403	0.5829	0.4598	0.7128	0.1692
CV-39	0.6218	0.4255	0.7238	0.5746	0.4444	0.7081	0.1509
CV-40	0.6764	0.5000	0.7680	0.6340	0.5281	0.7473	0.2717
CV-41	0.6182	0.3511	0.7569	0.5540	0.4286	0.6919	0.1141
CV-42	0.6255	0.4362	0.7238	0.5800	0.4505	0.7120	0.1612
CV-43	0.6909	0.5319	0.7735	0.6527	0.5495	0.7609	0.3078
CV-44	0.6727	0.5106	0.7569	0.6338	0.5217	0.7486	0.2690
CV-45	0.6873	0.5532	0.7569	0.6550	0.5417	0.7654	0.3086
CV-46	0.6545	0.4574	0.7569	0.6072	0.4943	0.7287	0.2186
CV-47	0.6764	0.4362	0.8011	0.6186	0.5325	0.7323	0.2507
CV-48	0.6364	0.3936	0.7624	0.5780	0.4625	0.7077	0.1630
CV-49	0.6327	0.3936	0.7569	0.5753	0.4568	0.7062	0.1566
CV-50	0.6509	0.5106	0.7238	0.6172	0.4898	0.7401	0.2321

CV-51	0.5964	0.3830	0.7072	0.5451	0.4045	0.6882	0.0914
CV-52	0.6291	0.4787	0.7072	0.5930	0.4592	0.7232	0.1841
CV-53	0.6509	0.5745	0.6906	0.6325	0.4909	0.7576	0.2566
CV-54	0.6655	0.5319	0.7348	0.6334	0.5102	0.7514	0.2642
CV-55	0.6364	0.4787	0.7182	0.5985	0.4688	0.7263	0.1960
CV-56	0.6145	0.3936	0.7293	0.5614	0.4302	0.6984	0.1257
CV-57	0.6255	0.4043	0.7403	0.5723	0.4471	0.7053	0.1484
CV-58	0.6655	0.4787	0.7624	0.6206	0.5114	0.7380	0.2452
CV-59	0.6145	0.4043	0.7238	0.5640	0.4318	0.7005	0.1302
CV-60	0.6473	0.5000	0.7238	0.6119	0.4845	0.7360	0.2221
CV-61	0.6618	0.4255	0.7845	0.6050	0.5063	0.7245	0.2202
CV-62	0.6945	0.5532	0.7680	0.6606	0.5532	0.7680	0.3211
CV-63	0.6218	0.3617	0.7569	0.5593	0.4359	0.6954	0.1248
CV-64	0.6400	0.4362	0.7459	0.5910	0.4713	0.7181	0.1857
CV-65	0.6800	0.4787	0.7845	0.6316	0.5357	0.7435	0.2711
CV-66	0.6291	0.3936	0.7514	0.5725	0.4512	0.7047	0.1503
CV-67	0.6473	0.4043	0.7735	0.5889	0.4810	0.7143	0.1863
CV-68	0.6400	0.4468	0.7403	0.5936	0.4719	0.7204	0.1897
CV-69	0.6509	0.4255	0.7680	0.5967	0.4878	0.7202	0.2006
CV-70	0.6182	0.3723	0.7459	0.5591	0.4321	0.6959	0.1230
CV-71	0.6618	0.4362	0.7790	0.6076	0.5062	0.7268	0.2239
CV-72	0.6945	0.5319	0.7790	0.6555	0.5556	0.7622	0.3143
CV-73	0.5964	0.4894	0.6519	0.5706	0.4220	0.7108	0.1370
CV-74	0.6145	0.4362	0.7072	0.5717	0.4362	0.7072	0.1434
CV-75	0.6364	0.3936	0.7624	0.5780	0.4625	0.7077	0.1630
CV-76	0.6255	0.3830	0.7514	0.5672	0.4444	0.7010	0.1398
CV-77	0.6218	0.4787	0.6961	0.5874	0.4500	0.7200	0.1724
CV-78	0.6364	0.4681	0.7238	0.5959	0.4681	0.7238	0.1918
CV-79	0.6436	0.4894	0.7238	0.6066	0.4792	0.7318	0.2121
CV-80	0.6545	0.4362	0.7680	0.6021	0.4940	0.7240	0.2109
CV-81	0.6655	0.3936	0.8066	0.6001	0.5139	0.7192	0.2161
CV-82	0.6364	0.3723	0.7735	0.5729	0.4605	0.7035	0.1547
CV-83	0.6400	0.4894	0.7182	0.6038	0.4742	0.7303	0.2061
CV-84	0.6255	0.4468	0.7182	0.5825	0.4516	0.7143	0.1655
CV-85	0.6036	0.4362	0.6906	0.5634	0.4227	0.7022	0.1258
CV-86	0.6691	0.4574	0.7790	0.6182	0.5181	0.7344	0.2443
CV-87	0.6145	0.3617	0.7459	0.5538	0.4250	0.6923	0.1123
CV-88	0.6509	0.4468	0.7569	0.6019	0.4884	0.7249	0.2084
CV-89	0.6000	0.4043	0.7017	0.5530	0.4130	0.6940	0.1065
CV-90	0.6182	0.3830	0.7403	0.5617	0.4337	0.6979	0.1274
CV-91	0.6691	0.4894	0.7624	0.6259	0.5169	0.7419	0.2553
CV-92	0.6327	0.4681	0.7182	0.5932	0.4632	0.7222	0.1858
CV-93	0.6509	0.4681	0.7459	0.6070	0.4889	0.7297	0.2163
CV-94	0.6218	0.4362	0.7182	0.5772	0.4457	0.7104	0.1552
CV-95	0.6582	0.5319	0.7238	0.6278	0.5000	0.7486	0.2521
CV-96	0.6218	0.5638	0.6519	0.6079	0.4569	0.7421	0.2072
CV-97	0.6400	0.4681	0.7293	0.5987	0.4731	0.7253	0.1979
CV-98	0.5855	0.4362	0.6630	0.5496	0.4020	0.6936	0.0974
CV-99	0.6109	0.4894	0.6740	0.5817	0.4381	0.7176	0.1595
CV-100	0.6291	0.4468	0.7238	0.5853	0.4565	0.7158	0.1715
CV-101	0.5745	0.3617	0.6851	0.5234	0.3736	0.6739	0.0472
CV-102	0.6036	0.3511	0.7348	0.5429	0.4074	0.6856	0.0894
CV-103	0.6145	0.4255	0.7127	0.5691	0.4348	0.7049	0.1390
CV-104	0.6400	0.4787	0.7238	0.6012	0.4737	0.7278	0.2020
CV-105	0.6327	0.4574	0.7238	0.5906	0.4624	0.7198	0.1817
CV-106	0.6036	0.4255	0.6961	0.5608	0.4211	0.7000	0.1214
CV-107	0.6364	0.4468	0.7348	0.5908	0.4667	0.7189	0.1836

CV-108	0.6436	0.4255	0.7569	0.5912	0.4762	0.7173	0.1879
CV-109	0.6327	0.3723	0.7680	0.5701	0.4545	0.7020	0.1482
CV-110	0.6400	0.3936	0.7680	0.5808	0.4684	0.7092	0.1694
CV-111	0.6327	0.4149	0.7459	0.5804	0.4588	0.7105	0.1650
CV-112	0.6909	0.4787	0.8011	0.6399	0.5556	0.7474	0.2912
CV-113	0.6909	0.5106	0.7845	0.6476	0.5517	0.7553	0.3010
CV-114	0.5964	0.3723	0.7127	0.5425	0.4023	0.6862	0.0867
CV-115	0.6073	0.3723	0.7293	0.5508	0.4167	0.6911	0.1046
CV-116	0.6509	0.4681	0.7459	0.6070	0.4889	0.7297	0.2163
CV-117	0.6655	0.4362	0.7845	0.6104	0.5125	0.7282	0.2305
CV-118	0.5855	0.4255	0.6685	0.5470	0.4000	0.6914	0.0927
CV-119	0.6509	0.5638	0.6961	0.6300	0.4907	0.7545	0.2525
CV-120	0.6327	0.4468	0.7293	0.5880	0.4615	0.7174	0.1775
CV-121	0.6655	0.4574	0.7735	0.6155	0.5119	0.7330	0.2378
CV-122	0.6255	0.4362	0.7238	0.5800	0.4505	0.7120	0.1612
CV-123	0.6145	0.3511	0.7514	0.5512	0.4231	0.6904	0.1078
CV-124	0.6473	0.4681	0.7403	0.6042	0.4835	0.7283	0.2101
CV-125	0.6145	0.5106	0.6685	0.5896	0.4444	0.7246	0.1740
CV-126	0.6836	0.4043	0.8287	0.6165	0.5507	0.7282	0.2549
CV-127	0.6400	0.4681	0.7293	0.5987	0.4731	0.7253	0.1979
CV-128	0.5891	0.4255	0.6740	0.5498	0.4040	0.6932	0.0984
CV-129	0.5927	0.3936	0.6961	0.5449	0.4022	0.6885	0.0902
CV-130	0.6509	0.4362	0.7624	0.5993	0.4881	0.7225	0.2045
CV-131	0.6218	0.4255	0.7238	0.5746	0.4444	0.7081	0.1509
CV-132	0.6000	0.4255	0.6906	0.5581	0.4167	0.6983	0.1156
CV-133	0.6255	0.4362	0.7238	0.5800	0.4505	0.7120	0.1612
CV-134	0.6691	0.4149	0.8011	0.6080	0.5200	0.7250	0.2300
CV-135	0.6691	0.3511	0.8343	0.5927	0.5238	0.7123	0.2092
CV-136	0.6945	0.5213	0.7845	0.6529	0.5568	0.7594	0.3109
CV-137	0.6327	0.4149	0.7459	0.5804	0.4588	0.7105	0.1650
CV-138	0.6473	0.5106	0.7182	0.6144	0.4848	0.7386	0.2262
CV-139	0.6364	0.4362	0.7403	0.5883	0.4659	0.7166	0.1795
CV-140	0.6327	0.3936	0.7569	0.5753	0.4568	0.7062	0.1566
CV-141	0.6582	0.5000	0.7403	0.6202	0.5000	0.7403	0.2403
CV-142	0.6400	0.5532	0.6851	0.6191	0.4771	0.7470	0.2311
CV-143	0.6436	0.4255	0.7569	0.5912	0.4762	0.7173	0.1879
CV-144	0.6327	0.4362	0.7348	0.5855	0.4607	0.7151	0.1733
CV-145	0.6655	0.4468	0.7790	0.6129	0.5122	0.7306	0.2341
CV-146	0.6364	0.3191	0.8011	0.5601	0.4545	0.6938	0.1336
CV-147	0.6000	0.3404	0.7348	0.5376	0.4000	0.6821	0.0786
CV-148	0.6255	0.3617	0.7624	0.5621	0.4416	0.6970	0.1311
CV-149	0.6255	0.3511	0.7680	0.5595	0.4400	0.6950	0.1268
CV-150	0.6145	0.2872	0.7845	0.5359	0.4091	0.6794	0.0797
CV-151	0.6545	0.4149	0.7790	0.5969	0.4937	0.7194	0.2033
CV-152	0.5855	0.4681	0.6464	0.5572	0.4074	0.7006	0.1112
CV-153	0.6291	0.3723	0.7624	0.5674	0.4487	0.7005	0.1418
CV-154	0.6400	0.3936	0.7680	0.5808	0.4684	0.7092	0.1694
CV-155	0.6400	0.4255	0.7514	0.5885	0.4706	0.7158	0.1816
CV-156	0.6436	0.4362	0.7514	0.5938	0.4767	0.7196	0.1919
CV-157	0.6291	0.4574	0.7182	0.5878	0.4574	0.7182	0.1757
CV-158	0.6182	0.4149	0.7238	0.5693	0.4382	0.7043	0.1406
CV-159	0.6255	0.4255	0.7293	0.5774	0.4494	0.7097	0.1569
CV-160	0.6364	0.4468	0.7348	0.5908	0.4667	0.7189	0.1836
CV-161	0.6764	0.5106	0.7624	0.6365	0.5275	0.7500	0.2753
CV-162	0.6618	0.4255	0.7845	0.6050	0.5063	0.7245	0.2202
CV-163	0.6545	0.4255	0.7735	0.5995	0.4938	0.7216	0.2071
CV-164	0.5818	0.4043	0.6740	0.5391	0.3918	0.6854	0.0777

CV-165	0.6145	0.4043	0.7238	0.5640	0.4318	0.7005	0.1302
CV-166	0.6400	0.4894	0.7182	0.6038	0.4742	0.7303	0.2061
CV-167	0.5891	0.3830	0.6961	0.5396	0.3956	0.6848	0.0797
CV-168	0.6400	0.4894	0.7182	0.6038	0.4742	0.7303	0.2061
CV-169	0.6291	0.3617	0.7680	0.5648	0.4474	0.6985	0.1375
CV-170	0.6073	0.4681	0.6796	0.5738	0.4314	0.7110	0.1450
CV-171	0.6291	0.4681	0.7127	0.5904	0.4583	0.7207	0.1799
CV-172	0.6182	0.5319	0.6630	0.5974	0.4505	0.7317	0.1884
CV-173	0.6473	0.4149	0.7680	0.5914	0.4815	0.7165	0.1903
CV-174	0.6545	0.4894	0.7403	0.6148	0.4946	0.7363	0.2303
CV-175	0.6109	0.3936	0.7238	0.5587	0.4253	0.6968	0.1197
CV-176	0.6873	0.5426	0.7624	0.6525	0.5426	0.7624	0.3050
CV-177	0.6582	0.4362	0.7735	0.6048	0.5000	0.7254	0.2174
CV-178	0.6327	0.3617	0.7735	0.5676	0.4533	0.7000	0.1440
CV-179	0.6473	0.3936	0.7790	0.5863	0.4805	0.7121	0.1824
CV-180	0.6764	0.4149	0.8122	0.6135	0.5342	0.7277	0.2439
CV-181	0.6218	0.4149	0.7293	0.5721	0.4432	0.7059	0.1466
CV-182	0.6655	0.4149	0.7956	0.6052	0.5132	0.7236	0.2232
CV-183	0.6291	0.4362	0.7293	0.5827	0.4556	0.7135	0.1673
CV-184	0.6109	0.4043	0.7182	0.5612	0.4270	0.6989	0.1242
CV-185	0.6509	0.4255	0.7680	0.5967	0.4878	0.7202	0.2006
CV-186	0.6145	0.3617	0.7459	0.5538	0.4250	0.6923	0.1123
CV-187	0.6473	0.4574	0.7459	0.6017	0.4831	0.7258	0.2061
CV-188	0.5891	0.4574	0.6575	0.5575	0.4095	0.7000	0.1122
CV-189	0.6218	0.4894	0.6906	0.5900	0.4510	0.7225	0.1767
CV-190	0.6909	0.5957	0.7403	0.6680	0.5437	0.7791	0.3294
CV-191	0.6218	0.5106	0.6796	0.5951	0.4528	0.7278	0.1854
CV-192	0.6036	0.3511	0.7348	0.5429	0.4074	0.6856	0.0894
CV-193	0.6291	0.4468	0.7238	0.5853	0.4565	0.7158	0.1715
CV-194	0.6364	0.4362	0.7403	0.5883	0.4659	0.7166	0.1795
CV-195	0.6291	0.3723	0.7624	0.5674	0.4487	0.7005	0.1418
CV-196	0.6327	0.4681	0.7182	0.5932	0.4632	0.7222	0.1858
CV-197	0.6073	0.4255	0.7017	0.5636	0.4255	0.7017	0.1272
CV-198	0.6509	0.5000	0.7293	0.6146	0.4896	0.7374	0.2281
CV-199	0.6618	0.5319	0.7293	0.6306	0.5051	0.7500	0.2581
CV-200	0.6400	0.4043	0.7624	0.5833	0.4691	0.7113	0.1734
CV-201	0.5855	0.4468	0.6575	0.5521	0.4038	0.6959	0.1020
CV-202	0.6582	0.4255	0.7790	0.6023	0.5000	0.7231	0.2136
CV-203	0.6291	0.4681	0.7127	0.5904	0.4583	0.7207	0.1799
CV-204	0.6218	0.4468	0.7127	0.5798	0.4468	0.7127	0.1595
CV-205	0.6836	0.5000	0.7790	0.6395	0.5402	0.7500	0.2846
CV-206	0.6291	0.4043	0.7459	0.5751	0.4524	0.7068	0.1546
CV-207	0.6509	0.4468	0.7569	0.6019	0.4884	0.7249	0.2084
CV-208	0.6036	0.3830	0.7182	0.5506	0.4138	0.6915	0.1032
CV-209	0.6655	0.4468	0.7790	0.6129	0.5122	0.7306	0.2341
CV-210	0.6145	0.3936	0.7293	0.5614	0.4302	0.6984	0.1257
CV-211	0.5636	0.4043	0.6464	0.5253	0.3725	0.6763	0.0497
CV-212	0.6436	0.3298	0.8066	0.5682	0.4697	0.6986	0.1515
CV-213	0.6000	0.4468	0.6796	0.5632	0.4200	0.7029	0.1246
CV-214	0.6364	0.3830	0.7680	0.5755	0.4615	0.7056	0.1588
CV-215	0.5927	0.3830	0.7017	0.5423	0.4000	0.6865	0.0856
CV-216	0.6582	0.4787	0.7514	0.6151	0.5000	0.7351	0.2326
CV-217	0.6182	0.3617	0.7514	0.5565	0.4304	0.6939	0.1185
CV-218	0.6145	0.4468	0.7017	0.5742	0.4375	0.7095	0.1477
CV-219	0.5891	0.4362	0.6685	0.5523	0.4059	0.6954	0.1030
CV-220	0.6036	0.3298	0.7459	0.5378	0.4026	0.6818	0.0799
CV-221	0.6255	0.3936	0.7459	0.5697	0.4458	0.7031	0.1441

CV-222	0.6327	0.4681	0.7182	0.5932	0.4632	0.7222	0.1858
CV-223	0.6109	0.3936	0.7238	0.5587	0.4253	0.6968	0.1197
CV-224	0.6509	0.3936	0.7845	0.5891	0.4868	0.7136	0.1890
CV-225	0.6109	0.3617	0.7403	0.5510	0.4198	0.6907	0.1062
CV-226	0.6218	0.4149	0.7293	0.5721	0.4432	0.7059	0.1466
CV-227	0.6364	0.4787	0.7182	0.5985	0.4688	0.7263	0.1960
CV-228	0.6364	0.4574	0.7293	0.5934	0.4674	0.7213	0.1877
CV-229	0.6218	0.4043	0.7348	0.5695	0.4419	0.7037	0.1423
CV-230	0.6182	0.3298	0.7680	0.5489	0.4247	0.6881	0.1050
CV-231	0.6327	0.4149	0.7459	0.5804	0.4588	0.7105	0.1650
CV-232	0.6182	0.4043	0.7293	0.5668	0.4368	0.7021	0.1362
CV-233	0.6364	0.4362	0.7403	0.5883	0.4659	0.7166	0.1795
CV-234	0.6400	0.4468	0.7403	0.5936	0.4719	0.7204	0.1897
CV-235	0.6218	0.3617	0.7569	0.5593	0.4359	0.6954	0.1248
CV-236	0.6436	0.4681	0.7348	0.6014	0.4783	0.7268	0.2040
CV-237	0.6291	0.4574	0.7182	0.5878	0.4574	0.7182	0.1757
CV-238	0.5964	0.3936	0.7017	0.5476	0.4066	0.6902	0.0960
CV-239	0.5673	0.3936	0.6575	0.5255	0.3737	0.6761	0.0505
CV-240	0.6182	0.4362	0.7127	0.5744	0.4409	0.7088	0.1493
CV-241	0.6255	0.3830	0.7514	0.5672	0.4444	0.7010	0.1398
CV-242	0.6073	0.4149	0.7072	0.5610	0.4239	0.6995	0.1227
CV-243	0.6800	0.4787	0.7845	0.6316	0.5357	0.7435	0.2711
CV-244	0.6182	0.4681	0.6961	0.5821	0.4444	0.7159	0.1623
CV-245	0.6145	0.3830	0.7348	0.5589	0.4286	0.6963	0.1213
CV-246	0.6545	0.4043	0.7845	0.5944	0.4935	0.7172	0.1994
CV-247	0.6618	0.3723	0.8122	0.5922	0.5072	0.7136	0.2019
CV-248	0.6291	0.4574	0.7182	0.5878	0.4574	0.7182	0.1757
CV-249	0.6218	0.4362	0.7182	0.5772	0.4457	0.7104	0.1552
CV-250	0.6545	0.4681	0.7514	0.6097	0.4944	0.7312	0.2225
CV-251	0.6255	0.4468	0.7182	0.5825	0.4516	0.7143	0.1655
CV-252	0.6291	0.4894	0.7017	0.5955	0.4600	0.7257	0.1883
CV-253	0.6509	0.4043	0.7790	0.5916	0.4872	0.7157	0.1928
CV-254	0.7236	0.5532	0.8122	0.6827	0.6047	0.7778	0.3738
CV-255	0.6473	0.4149	0.7680	0.5914	0.4815	0.7165	0.1903
CV-256	0.6764	0.5319	0.7514	0.6416	0.5263	0.7556	0.2826
CV-257	0.6000	0.3511	0.7293	0.5402	0.4024	0.6839	0.0833
CV-258	0.6691	0.4787	0.7680	0.6233	0.5172	0.7394	0.2516
CV-259	0.6255	0.3830	0.7514	0.5672	0.4444	0.7010	0.1398
CV-260	0.6473	0.3936	0.7790	0.5863	0.4805	0.7121	0.1824
CV-261	0.7018	0.5319	0.7901	0.6610	0.5682	0.7647	0.3274
CV-262	0.6145	0.4362	0.7072	0.5717	0.4362	0.7072	0.1434
CV-263	0.6509	0.4255	0.7680	0.5967	0.4878	0.7202	0.2006
CV-264	0.6873	0.4894	0.7901	0.6397	0.5476	0.7487	0.2877
CV-265	0.6655	0.4468	0.7790	0.6129	0.5122	0.7306	0.2341
CV-266	0.6145	0.5106	0.6685	0.5896	0.4444	0.7246	0.1740
CV-267	0.6000	0.3723	0.7182	0.5453	0.4070	0.6878	0.0927
CV-268	0.6073	0.4255	0.7017	0.5636	0.4255	0.7017	0.1272
CV-269	0.6145	0.4149	0.7182	0.5666	0.4333	0.7027	0.1346
CV-270	0.6473	0.4894	0.7293	0.6093	0.4842	0.7333	0.2181
CV-271	0.6364	0.3936	0.7624	0.5780	0.4625	0.7077	0.1630
CV-272	0.5891	0.3723	0.7017	0.5370	0.3933	0.6828	0.0750
CV-273	0.6327	0.4043	0.7514	0.5778	0.4578	0.7083	0.1608
CV-274	0.6364	0.4574	0.7293	0.5934	0.4674	0.7213	0.1877
CV-275	0.6509	0.4894	0.7348	0.6121	0.4894	0.7348	0.2242
CV-276	0.6727	0.4149	0.8066	0.6108	0.5270	0.7264	0.2369
CV-277	0.6000	0.3404	0.7348	0.5376	0.4000	0.6821	0.0786
CV-278	0.6364	0.4149	0.7514	0.5831	0.4643	0.7120	0.1712

CV-279	0.6255	0.4362	0.7238	0.5800	0.4505	0.7120	0.1612
CV-280	0.6436	0.5000	0.7182	0.6091	0.4796	0.7345	0.2161
CV-281	0.6255	0.3830	0.7514	0.5672	0.4444	0.7010	0.1398
CV-282	0.6327	0.3191	0.7956	0.5574	0.4478	0.6923	0.1268
CV-283	0.6364	0.3830	0.7680	0.5755	0.4615	0.7056	0.1588
CV-284	0.6509	0.4149	0.7735	0.5942	0.4875	0.7179	0.1967
CV-285	0.6109	0.3617	0.7403	0.5510	0.4198	0.6907	0.1062
CV-286	0.6291	0.5426	0.6740	0.6083	0.4636	0.7394	0.2097
CV-287	0.6436	0.4894	0.7238	0.6066	0.4792	0.7318	0.2121
CV-288	0.6218	0.3936	0.7403	0.5670	0.4405	0.7016	0.1379
CV-289	0.6109	0.4574	0.6906	0.5740	0.4343	0.7102	0.1463
CV-290	0.6436	0.4574	0.7403	0.5989	0.4778	0.7243	0.1999
CV-291	0.6618	0.5000	0.7459	0.6229	0.5054	0.7418	0.2465
CV-292	0.6691	0.5213	0.7459	0.6336	0.5158	0.7500	0.2665
CV-293	0.6836	0.4894	0.7845	0.6369	0.5412	0.7474	0.2811
CV-294	0.6436	0.4362	0.7514	0.5938	0.4767	0.7196	0.1919
CV-295	0.6691	0.4894	0.7624	0.6259	0.5169	0.7419	0.2553
CV-296	0.6327	0.4468	0.7293	0.5880	0.4615	0.7174	0.1775
CV-297	0.6218	0.4149	0.7293	0.5721	0.4432	0.7059	0.1466
CV-298	0.5927	0.4255	0.6796	0.5525	0.4082	0.6949	0.1041
CV-299	0.6727	0.4468	0.7901	0.6184	0.5250	0.7333	0.2474
CV-300	0.6545	0.5000	0.7348	0.6174	0.4947	0.7389	0.2342
CV-301	0.6327	0.4043	0.7514	0.5778	0.4578	0.7083	0.1608
CV-302	0.6618	0.5106	0.7403	0.6255	0.5053	0.7444	0.2503
CV-303	0.6182	0.4681	0.6961	0.5821	0.4444	0.7159	0.1623
CV-304	0.6109	0.4149	0.7127	0.5638	0.4286	0.7011	0.1286
CV-305	0.6836	0.4894	0.7845	0.6369	0.5412	0.7474	0.2811
CV-306	0.6618	0.4149	0.7901	0.6025	0.5065	0.7222	0.2165
CV-307	0.6291	0.4149	0.7403	0.5776	0.4535	0.7090	0.1588
CV-308	0.6545	0.5319	0.7182	0.6251	0.4950	0.7471	0.2461
CV-309	0.6545	0.4681	0.7514	0.6097	0.4944	0.7312	0.2225
CV-310	0.5927	0.3511	0.7182	0.5346	0.3929	0.6806	0.0714
CV-311	0.6327	0.4681	0.7182	0.5932	0.4632	0.7222	0.1858
CV-312	0.6582	0.4787	0.7514	0.6151	0.5000	0.7351	0.2326
CV-313	0.6545	0.5000	0.7348	0.6174	0.4947	0.7389	0.2342
CV-314	0.5818	0.3617	0.6961	0.5289	0.3820	0.6774	0.0586
CV-315	0.6982	0.4255	0.8398	0.6327	0.5797	0.7379	0.2903
CV-316	0.5927	0.4681	0.6575	0.5628	0.4151	0.7041	0.1223
CV-317	0.6291	0.4468	0.7238	0.5853	0.4565	0.7158	0.1715
CV-318	0.6218	0.4574	0.7072	0.5823	0.4479	0.7151	0.1638
CV-319	0.6655	0.4468	0.7790	0.6129	0.5122	0.7306	0.2341
CV-320	0.6509	0.3830	0.7901	0.5865	0.4865	0.7114	0.1851
CV-321	0.5745	0.3830	0.6740	0.5285	0.3789	0.6778	0.0569
CV-322	0.6327	0.5106	0.6961	0.6034	0.4660	0.7326	0.2026
CV-323	0.6764	0.4468	0.7956	0.6212	0.5316	0.7347	0.2541
CV-324	0.6218	0.4149	0.7293	0.5721	0.4432	0.7059	0.1466
CV-325	0.6509	0.4149	0.7735	0.5942	0.4875	0.7179	0.1967
CV-326	0.6582	0.4149	0.7845	0.5997	0.5000	0.7208	0.2098
CV-327	0.6618	0.5532	0.7182	0.6357	0.5049	0.7558	0.2660
CV-328	0.6218	0.4149	0.7293	0.5721	0.4432	0.7059	0.1466
CV-329	0.6764	0.5638	0.7348	0.6493	0.5248	0.7644	0.2938
CV-330	0.6364	0.4681	0.7238	0.5959	0.4681	0.7238	0.1918
CV-331	0.6364	0.4894	0.7127	0.6010	0.4694	0.7288	0.2001
CV-332	0.5964	0.3191	0.7403	0.5297	0.3896	0.6768	0.0628
CV-333	0.6364	0.3830	0.7680	0.5755	0.4615	0.7056	0.1588
CV-334	0.6400	0.3936	0.7680	0.5808	0.4684	0.7092	0.1694
CV-335	0.6691	0.4681	0.7735	0.6208	0.5176	0.7368	0.2479

CV-336	0.6764	0.5319	0.7514	0.6416	0.5263	0.7556	0.2826
CV-337	0.6036	0.4787	0.6685	0.5736	0.4286	0.7118	0.1437
CV-338	0.5964	0.3511	0.7238	0.5374	0.3976	0.6823	0.0773
CV-339	0.6218	0.4362	0.7182	0.5772	0.4457	0.7104	0.1552
CV-340	0.6400	0.4894	0.7182	0.6038	0.4742	0.7303	0.2061
CV-341	0.6582	0.4149	0.7845	0.5997	0.5000	0.7208	0.2098
CV-342	0.6400	0.4043	0.7624	0.5833	0.4691	0.7113	0.1734
CV-343	0.5964	0.3404	0.7293	0.5349	0.3951	0.6804	0.0725
CV-344	0.6582	0.4787	0.7514	0.6151	0.5000	0.7351	0.2326
CV-345	0.6218	0.4149	0.7293	0.5721	0.4432	0.7059	0.1466
CV-346	0.6509	0.4574	0.7514	0.6044	0.4886	0.7273	0.2123
CV-347	0.6473	0.4574	0.7459	0.6017	0.4831	0.7258	0.2061
CV-348	0.6509	0.4468	0.7569	0.6019	0.4884	0.7249	0.2084
CV-349	0.5600	0.3085	0.6906	0.4996	0.3412	0.6579	-0.0009
CV-350	0.6291	0.4362	0.7293	0.5827	0.4556	0.7135	0.1673
CV-351	0.6545	0.4574	0.7569	0.6072	0.4943	0.7287	0.2186
CV-352	0.6255	0.3830	0.7514	0.5672	0.4444	0.7010	0.1398
CV-353	0.6364	0.4894	0.7127	0.6010	0.4694	0.7288	0.2001
CV-354	0.6218	0.5213	0.6740	0.5977	0.4537	0.7305	0.1897
CV-355	0.6255	0.4681	0.7072	0.5876	0.4536	0.7191	0.1740
CV-356	0.6909	0.4468	0.8177	0.6322	0.5600	0.7400	0.2817
CV-357	0.6400	0.4149	0.7569	0.5859	0.4699	0.7135	0.1775
CV-358	0.6182	0.4681	0.6961	0.5821	0.4444	0.7159	0.1623
CV-359	0.6509	0.4681	0.7459	0.6070	0.4889	0.7297	0.2163
CV-360	0.6000	0.3830	0.7127	0.5478	0.4091	0.6898	0.0973
CV-361	0.6436	0.4043	0.7680	0.5861	0.4750	0.7128	0.1798
CV-362	0.6691	0.5106	0.7514	0.6310	0.5161	0.7473	0.2627
CV-363	0.6364	0.3617	0.7790	0.5704	0.4595	0.7015	0.1505
CV-364	0.6400	0.4468	0.7403	0.5936	0.4719	0.7204	0.1897
CV-365	0.6145	0.3723	0.7403	0.5563	0.4268	0.6943	0.1168
CV-366	0.6400	0.4043	0.7624	0.5833	0.4691	0.7113	0.1734
CV-367	0.6473	0.4149	0.7680	0.5914	0.4815	0.7165	0.1903
CV-368	0.6036	0.3298	0.7459	0.5378	0.4026	0.6818	0.0799
CV-369	0.5964	0.3085	0.7459	0.5272	0.3867	0.6750	0.0579
CV-370	0.6655	0.4362	0.7845	0.6104	0.5125	0.7282	0.2305
CV-371	0.6436	0.4149	0.7624	0.5887	0.4756	0.7150	0.1839
CV-372	0.6000	0.4149	0.6961	0.5555	0.4149	0.6961	0.1110
CV-373	0.5855	0.3511	0.7072	0.5291	0.3837	0.6772	0.0596
CV-374	0.6436	0.4362	0.7514	0.5938	0.4767	0.7196	0.1919
CV-375	0.5927	0.3298	0.7293	0.5295	0.3875	0.6769	0.0617
CV-376	0.6000	0.2979	0.7569	0.5274	0.3889	0.6749	0.0591
CV-377	0.6145	0.3936	0.7293	0.5614	0.4302	0.6984	0.1257
CV-378	0.6327	0.4574	0.7238	0.5906	0.4624	0.7198	0.1817
CV-379	0.5964	0.3511	0.7238	0.5374	0.3976	0.6823	0.0773
CV-380	0.5927	0.4255	0.6796	0.5525	0.4082	0.6949	0.1041
CV-381	0.6509	0.5000	0.7293	0.6146	0.4896	0.7374	0.2281
CV-382	0.6291	0.4574	0.7182	0.5878	0.4574	0.7182	0.1757
CV-383	0.6036	0.3830	0.7182	0.5506	0.4138	0.6915	0.1032
CV-384	0.6327	0.3830	0.7624	0.5727	0.4557	0.7041	0.1524
CV-385	0.5782	0.3830	0.6796	0.5313	0.3830	0.6796	0.0625
CV-386	0.5891	0.4468	0.6630	0.5549	0.4078	0.6977	0.1076
CV-387	0.5527	0.2979	0.6851	0.4915	0.3294	0.6526	-0.0175
CV-388	0.6036	0.2553	0.7845	0.5199	0.3810	0.6698	0.0450
CV-389	0.6509	0.4574	0.7514	0.6044	0.4886	0.7273	0.2123
CV-390	0.6655	0.4362	0.7845	0.6104	0.5125	0.7282	0.2305
CV-391	0.6109	0.3936	0.7238	0.5587	0.4253	0.6968	0.1197
CV-392	0.6109	0.4255	0.7072	0.5664	0.4301	0.7033	0.1331

CV-393	0.7164	0.4787	0.8398	0.6593	0.6081	0.7562	0.3406
CV-394	0.6618	0.4149	0.7901	0.6025	0.5065	0.7222	0.2165
CV-395	0.6618	0.4468	0.7735	0.6101	0.5060	0.7292	0.2276
CV-396	0.6255	0.4681	0.7072	0.5876	0.4536	0.7191	0.1740
CV-397	0.6145	0.3404	0.7569	0.5487	0.4211	0.6884	0.1032
CV-398	0.7091	0.5426	0.7956	0.6691	0.5795	0.7701	0.3438
CV-399	0.6509	0.4362	0.7624	0.5993	0.4881	0.7225	0.2045
CV-400	0.6436	0.4255	0.7569	0.5912	0.4762	0.7173	0.1879
CV-401	0.6000	0.3830	0.7127	0.5478	0.4091	0.6898	0.0973
CV-402	0.6473	0.5000	0.7238	0.6119	0.4845	0.7360	0.2221
CV-403	0.6545	0.4681	0.7514	0.6097	0.4944	0.7312	0.2225
CV-404	0.6836	0.4574	0.8011	0.6293	0.5443	0.7398	0.2710
CV-405	0.6364	0.4681	0.7238	0.5959	0.4681	0.7238	0.1918
CV-406	0.6109	0.4255	0.7072	0.5664	0.4301	0.7033	0.1331
CV-407	0.5891	0.4043	0.6851	0.5447	0.4000	0.6889	0.0891
CV-408	0.6291	0.4255	0.7348	0.5802	0.4545	0.7112	0.1630
CV-409	0.6218	0.3511	0.7624	0.5567	0.4342	0.6935	0.1204
CV-410	0.6255	0.3617	0.7624	0.5621	0.4416	0.6970	0.1311
CV-411	0.6036	0.4362	0.6906	0.5634	0.4227	0.7022	0.1258
CV-412	0.6473	0.4574	0.7459	0.6017	0.4831	0.7258	0.2061
CV-413	0.5891	0.4362	0.6685	0.5523	0.4059	0.6954	0.1030
CV-414	0.6145	0.4681	0.6906	0.5793	0.4400	0.7143	0.1565
CV-415	0.6182	0.4043	0.7293	0.5668	0.4368	0.7021	0.1362
CV-416	0.6618	0.3617	0.8177	0.5897	0.5075	0.7115	0.1982
CV-417	0.6291	0.4043	0.7459	0.5751	0.4524	0.7068	0.1546
CV-418	0.6000	0.4149	0.6961	0.5555	0.4149	0.6961	0.1110
CV-419	0.6000	0.3511	0.7293	0.5402	0.4024	0.6839	0.0833
CV-420	0.6109	0.4255	0.7072	0.5664	0.4301	0.7033	0.1331
CV-421	0.6255	0.3830	0.7514	0.5672	0.4444	0.7010	0.1398
CV-422	0.6364	0.3936	0.7624	0.5780	0.4625	0.7077	0.1630
CV-423	0.6436	0.5851	0.6740	0.6296	0.4825	0.7578	0.2495
CV-424	0.6073	0.3298	0.7514	0.5406	0.4079	0.6834	0.0861
CV-425	0.6582	0.3830	0.8011	0.5920	0.5000	0.7143	0.1986
CV-426	0.6036	0.3404	0.7403	0.5404	0.4051	0.6837	0.0847
CV-427	0.6182	0.5000	0.6796	0.5898	0.4476	0.7235	0.1753
CV-428	0.6218	0.4149	0.7293	0.5721	0.4432	0.7059	0.1466
CV-429	0.6327	0.4574	0.7238	0.5906	0.4624	0.7198	0.1817
CV-430	0.6073	0.4468	0.6906	0.5687	0.4286	0.7062	0.1361
CV-431	0.7345	0.4787	0.8674	0.6731	0.6522	0.7621	0.3787
CV-432	0.5927	0.4255	0.6796	0.5525	0.4082	0.6949	0.1041
CV-433	0.6473	0.4468	0.7514	0.5991	0.4828	0.7234	0.2021
CV-434	0.5855	0.3511	0.7072	0.5291	0.3837	0.6772	0.0596
CV-435	0.6291	0.3511	0.7735	0.5623	0.4459	0.6965	0.1332
CV-436	0.6364	0.4149	0.7514	0.5831	0.4643	0.7120	0.1712
CV-437	0.6145	0.4362	0.7072	0.5717	0.4362	0.7072	0.1434
CV-438	0.6291	0.5000	0.6961	0.5981	0.4608	0.7283	0.1926
CV-439	0.6691	0.5426	0.7348	0.6387	0.5152	0.7557	0.2741
CV-440	0.6109	0.4043	0.7182	0.5612	0.4270	0.6989	0.1242
CV-441	0.6436	0.4574	0.7403	0.5989	0.4778	0.7243	0.1999
CV-442	0.6364	0.4787	0.7182	0.5985	0.4688	0.7263	0.1960
CV-443	0.6073	0.4787	0.6740	0.5764	0.4327	0.7135	0.1494
CV-444	0.6291	0.4681	0.7127	0.5904	0.4583	0.7207	0.1799
CV-445	0.5927	0.4149	0.6851	0.5500	0.4063	0.6927	0.0995
CV-446	0.6145	0.3511	0.7514	0.5512	0.4231	0.6904	0.1078
CV-447	0.6109	0.4574	0.6906	0.5740	0.4343	0.7102	0.1463
CV-448	0.6145	0.5106	0.6685	0.5896	0.4444	0.7246	0.1740
CV-449	0.6618	0.5000	0.7459	0.6229	0.5054	0.7418	0.2465

CV-450	0.6473	0.4043	0.7735	0.5889	0.4810	0.7143	0.1863
CV-451	0.6255	0.4787	0.7017	0.5902	0.4545	0.7216	0.1782
CV-452	0.6109	0.4574	0.6906	0.5740	0.4343	0.7102	0.1463
CV-453	0.6327	0.4681	0.7182	0.5932	0.4632	0.7222	0.1858
CV-454	0.6364	0.4574	0.7293	0.5934	0.4674	0.7213	0.1877
CV-455	0.6145	0.4255	0.7127	0.5691	0.4348	0.7049	0.1390
CV-456	0.6145	0.4362	0.7072	0.5717	0.4362	0.7072	0.1434
CV-457	0.6473	0.4681	0.7403	0.6042	0.4835	0.7283	0.2101
CV-458	0.6073	0.3936	0.7182	0.5559	0.4205	0.6952	0.1137
CV-459	0.6691	0.4468	0.7845	0.6157	0.5185	0.7320	0.2407
CV-460	0.5673	0.3830	0.6630	0.5230	0.3711	0.6742	0.0456
CV-461	0.5673	0.3723	0.6685	0.5204	0.3684	0.6722	0.0407
CV-462	0.6218	0.3617	0.7569	0.5593	0.4359	0.6954	0.1248
CV-463	0.6655	0.5426	0.7293	0.6359	0.5100	0.7543	0.2680
CV-464	0.6400	0.3830	0.7735	0.5782	0.4675	0.7071	0.1653
CV-465	0.6000	0.4149	0.6961	0.5555	0.4149	0.6961	0.1110
CV-466	0.6655	0.4574	0.7735	0.6155	0.5119	0.7330	0.2378
CV-467	0.6255	0.4043	0.7403	0.5723	0.4471	0.7053	0.1484
CV-468	0.6400	0.4362	0.7459	0.5910	0.4713	0.7181	0.1857
CV-469	0.6509	0.4681	0.7459	0.6070	0.4889	0.7297	0.2163
CV-470	0.6327	0.3511	0.7790	0.5650	0.4521	0.6980	0.1397
CV-471	0.6473	0.3723	0.7901	0.5812	0.4795	0.7079	0.1744
CV-472	0.6545	0.4681	0.7514	0.6097	0.4944	0.7312	0.2225
CV-473	0.6109	0.3936	0.7238	0.5587	0.4253	0.6968	0.1197
CV-474	0.5782	0.3830	0.6796	0.5313	0.3830	0.6796	0.0625
CV-475	0.6764	0.4787	0.7790	0.6289	0.5294	0.7421	0.2645
CV-476	0.7055	0.5532	0.7845	0.6689	0.5714	0.7717	0.3404
CV-477	0.6436	0.4255	0.7569	0.5912	0.4762	0.7173	0.1879
CV-478	0.6545	0.4468	0.7624	0.6046	0.4941	0.7263	0.2148
CV-479	0.6145	0.4149	0.7182	0.5666	0.4333	0.7027	0.1346
CV-480	0.6182	0.4468	0.7072	0.5770	0.4421	0.7111	0.1536
CV-481	0.6182	0.3830	0.7403	0.5617	0.4337	0.6979	0.1274
CV-482	0.6109	0.4149	0.7127	0.5638	0.4286	0.7011	0.1286
CV-483	0.6255	0.4149	0.7348	0.5749	0.4483	0.7074	0.1527
CV-484	0.6509	0.5745	0.6906	0.6325	0.4909	0.7576	0.2566
CV-485	0.6218	0.3617	0.7569	0.5593	0.4359	0.6954	0.1248
CV-486	0.6727	0.4574	0.7845	0.6210	0.5244	0.7358	0.2509
CV-487	0.5709	0.3617	0.6796	0.5206	0.3696	0.6721	0.0415
CV-488	0.6364	0.4149	0.7514	0.5831	0.4643	0.7120	0.1712
CV-489	0.6691	0.4468	0.7845	0.6157	0.5185	0.7320	0.2407
CV-490	0.6800	0.4362	0.8066	0.6214	0.5395	0.7337	0.2575
CV-491	0.6291	0.4362	0.7293	0.5827	0.4556	0.7135	0.1673
CV-492	0.6255	0.3830	0.7514	0.5672	0.4444	0.7010	0.1398
CV-493	0.6473	0.4149	0.7680	0.5914	0.4815	0.7165	0.1903
CV-494	0.5855	0.3936	0.6851	0.5393	0.3936	0.6851	0.0787
CV-495	0.6400	0.4681	0.7293	0.5987	0.4731	0.7253	0.1979
CV-496	0.6145	0.3085	0.7735	0.5410	0.4143	0.6829	0.0893
CV-497	0.6109	0.4894	0.6740	0.5817	0.4381	0.7176	0.1595
CV-498	0.6145	0.4362	0.7072	0.5717	0.4362	0.7072	0.1434
CV-499	0.6182	0.3936	0.7348	0.5642	0.4353	0.7000	0.1318
CV-500	0.6400	0.4362	0.7459	0.5910	0.4713	0.7181	0.1857

Table S5. Performance of 5-fold cross-validations from decision forest (DF). Performance of 5-fold cross-validations were evaluated by seven performance metrics. Each row shows the performance of one time 5-fold cross-validation. 5-fold cross-validation was repeated 500 times (CV-1 ~ CV-500).

Cross-validation	Accuracy	Sensitivity	Specificity	Balanced accuracy	Positive prediction rate	Negative prediction rate	Matthews correlation coefficient
CV-1	0.6655	0.4362	0.7845	0.6104	0.5125	0.7282	0.2305
CV-2	0.6873	0.4149	0.8287	0.6218	0.5571	0.7317	0.2653
CV-3	0.6473	0.3511	0.8011	0.5761	0.4783	0.7039	0.1665
CV-4	0.6909	0.4043	0.8398	0.6220	0.5672	0.7308	0.2696
CV-5	0.6909	0.3723	0.8564	0.6143	0.5738	0.7243	0.2611
CV-6	0.6618	0.3404	0.8287	0.5846	0.5079	0.7075	0.1909
CV-7	0.6691	0.3936	0.8122	0.6029	0.5211	0.7206	0.2230
CV-8	0.7018	0.4468	0.8343	0.6405	0.5833	0.7438	0.3032
CV-9	0.6364	0.4468	0.7348	0.5908	0.4667	0.7189	0.1836
CV-10	0.6836	0.5106	0.7735	0.6421	0.5393	0.7527	0.2880
CV-11	0.6545	0.4468	0.7624	0.6046	0.4941	0.7263	0.2148
CV-12	0.6655	0.4149	0.7956	0.6052	0.5132	0.7236	0.2232
CV-13	0.6727	0.4255	0.8011	0.6133	0.5263	0.7286	0.2404
CV-14	0.7127	0.3936	0.8785	0.6360	0.6271	0.7361	0.3144
CV-15	0.6655	0.4149	0.7956	0.6052	0.5132	0.7236	0.2232
CV-16	0.7018	0.4574	0.8287	0.6431	0.5811	0.7463	0.3061
CV-17	0.6982	0.4255	0.8398	0.6327	0.5797	0.7379	0.2903
CV-18	0.6545	0.3723	0.8011	0.5867	0.4930	0.7108	0.1880
CV-19	0.6400	0.3617	0.7845	0.5731	0.4658	0.7030	0.1571
CV-20	0.7418	0.4681	0.8840	0.6760	0.6769	0.7619	0.3931
CV-21	0.6509	0.3830	0.7901	0.5865	0.4865	0.7114	0.1851
CV-22	0.6364	0.3404	0.7901	0.5652	0.4571	0.6976	0.1421
CV-23	0.6909	0.3617	0.8619	0.6118	0.5763	0.7222	0.2583
CV-24	0.6509	0.3298	0.8177	0.5737	0.4844	0.7014	0.1655
CV-25	0.6655	0.3830	0.8122	0.5976	0.5143	0.7171	0.2125
CV-26	0.6618	0.3936	0.8011	0.5974	0.5068	0.7178	0.2092
CV-27	0.6364	0.3936	0.7624	0.5780	0.4625	0.7077	0.1630
CV-28	0.6691	0.3723	0.8232	0.5978	0.5224	0.7163	0.2161
CV-29	0.6800	0.4787	0.7845	0.6316	0.5357	0.7435	0.2711
CV-30	0.7273	0.3830	0.9061	0.6445	0.6792	0.7387	0.3476
CV-31	0.6655	0.3723	0.8177	0.5950	0.5147	0.7150	0.2089
CV-32	0.6545	0.3830	0.7956	0.5893	0.4932	0.7129	0.1918
CV-33	0.6655	0.3511	0.8287	0.5899	0.5156	0.7109	0.2018
CV-34	0.6218	0.3191	0.7790	0.5491	0.4286	0.6878	0.1069
CV-35	0.6945	0.3936	0.8508	0.6222	0.5781	0.7299	0.2744
CV-36	0.6909	0.4255	0.8287	0.6271	0.5634	0.7353	0.2756
CV-37	0.6982	0.4255	0.8398	0.6327	0.5797	0.7379	0.2903
CV-38	0.6800	0.3936	0.8287	0.6112	0.5441	0.7246	0.2445
CV-39	0.6473	0.3617	0.7956	0.5786	0.4789	0.7059	0.1705
CV-40	0.6582	0.4574	0.7624	0.6099	0.5000	0.7302	0.2250
CV-41	0.6218	0.3191	0.7790	0.5491	0.4286	0.6878	0.1069
CV-42	0.6764	0.4681	0.7845	0.6263	0.5301	0.7396	0.2610
CV-43	0.6582	0.3723	0.8066	0.5895	0.5000	0.7122	0.1949
CV-44	0.6545	0.3191	0.8287	0.5739	0.4918	0.7009	0.1688
CV-45	0.6727	0.4468	0.7901	0.6184	0.5250	0.7333	0.2474
CV-46	0.6545	0.3511	0.8122	0.5816	0.4925	0.7067	0.1803
CV-47	0.6582	0.3723	0.8066	0.5895	0.5000	0.7122	0.1949
CV-48	0.6545	0.4043	0.7845	0.5944	0.4935	0.7172	0.1994
CV-49	0.6873	0.4362	0.8177	0.6269	0.5541	0.7363	0.2715
CV-50	0.6691	0.3617	0.8287	0.5952	0.5231	0.7143	0.2126

CV-51	0.7345	0.4787	0.8674	0.6731	0.6522	0.7621	0.3787
CV-52	0.6364	0.3511	0.7845	0.5678	0.4583	0.6995	0.1463
CV-53	0.6764	0.4149	0.8122	0.6135	0.5342	0.7277	0.2439
CV-54	0.6473	0.3085	0.8232	0.5659	0.4754	0.6963	0.1504
CV-55	0.6255	0.3191	0.7845	0.5518	0.4348	0.6893	0.1134
CV-56	0.6764	0.4043	0.8177	0.6110	0.5352	0.7255	0.2405
CV-57	0.7055	0.4043	0.8619	0.6331	0.6032	0.7358	0.3004
CV-58	0.6436	0.3723	0.7845	0.5784	0.4730	0.7065	0.1678
CV-59	0.6436	0.4149	0.7624	0.5887	0.4756	0.7150	0.1839
CV-60	0.6509	0.3830	0.7901	0.5865	0.4865	0.7114	0.1851
CV-61	0.6545	0.3723	0.8011	0.5867	0.4930	0.7108	0.1880
CV-62	0.6582	0.3936	0.7956	0.5946	0.5000	0.7164	0.2024
CV-63	0.6545	0.3830	0.7956	0.5893	0.4932	0.7129	0.1918
CV-64	0.6836	0.4894	0.7845	0.6369	0.5412	0.7474	0.2811
CV-65	0.6982	0.4255	0.8398	0.6327	0.5797	0.7379	0.2903
CV-66	0.6836	0.4681	0.7956	0.6318	0.5432	0.7423	0.2744
CV-67	0.6509	0.4043	0.7790	0.5916	0.4872	0.7157	0.1928
CV-68	0.6764	0.4362	0.8011	0.6186	0.5325	0.7323	0.2507
CV-69	0.6582	0.4043	0.7901	0.5972	0.5000	0.7186	0.2061
CV-70	0.6727	0.4255	0.8011	0.6133	0.5263	0.7286	0.2404
CV-71	0.7091	0.4574	0.8398	0.6486	0.5972	0.7488	0.3207
CV-72	0.6509	0.3723	0.7956	0.5840	0.4861	0.7094	0.1812
CV-73	0.6582	0.3511	0.8177	0.5844	0.5000	0.7081	0.1874
CV-74	0.6582	0.3830	0.8011	0.5920	0.5000	0.7143	0.1986
CV-75	0.6509	0.4255	0.7680	0.5967	0.4878	0.7202	0.2006
CV-76	0.6727	0.4468	0.7901	0.6184	0.5250	0.7333	0.2474
CV-77	0.6473	0.3617	0.7956	0.5786	0.4789	0.7059	0.1705
CV-78	0.6691	0.3511	0.8343	0.5927	0.5238	0.7123	0.2092
CV-79	0.6509	0.4043	0.7790	0.5916	0.4872	0.7157	0.1928
CV-80	0.6473	0.3830	0.7845	0.5838	0.4800	0.7100	0.1784
CV-81	0.6364	0.4255	0.7459	0.5857	0.4651	0.7143	0.1753
CV-82	0.6291	0.3830	0.7569	0.5699	0.4500	0.7026	0.1461
CV-83	0.6691	0.3723	0.8232	0.5978	0.5224	0.7163	0.2161
CV-84	0.6873	0.3936	0.8398	0.6167	0.5606	0.7273	0.2592
CV-85	0.7091	0.4787	0.8287	0.6537	0.5921	0.7538	0.3261
CV-86	0.6400	0.3830	0.7735	0.5782	0.4675	0.7071	0.1653
CV-87	0.6473	0.3617	0.7956	0.5786	0.4789	0.7059	0.1705
CV-88	0.6509	0.3830	0.7901	0.5865	0.4865	0.7114	0.1851
CV-89	0.6873	0.4255	0.8232	0.6244	0.5556	0.7340	0.2684
CV-90	0.6945	0.4255	0.8343	0.6299	0.5714	0.7366	0.2829
CV-91	0.6618	0.4468	0.7735	0.6101	0.5060	0.7292	0.2276
CV-92	0.6364	0.4149	0.7514	0.5831	0.4643	0.7120	0.1712
CV-93	0.6800	0.4468	0.8011	0.6240	0.5385	0.7360	0.2609
CV-94	0.6618	0.4043	0.7956	0.5999	0.5067	0.7200	0.2128
CV-95	0.6836	0.4468	0.8066	0.6267	0.5455	0.7374	0.2677
CV-96	0.6473	0.4149	0.7680	0.5914	0.4815	0.7165	0.1903
CV-97	0.6509	0.3617	0.8011	0.5814	0.4857	0.7073	0.1773
CV-98	0.6364	0.3936	0.7624	0.5780	0.4625	0.7077	0.1630
CV-99	0.6691	0.3723	0.8232	0.5978	0.5224	0.7163	0.2161
CV-100	0.7055	0.4787	0.8232	0.6510	0.5844	0.7525	0.3190
CV-101	0.6436	0.3404	0.8011	0.5708	0.4706	0.7005	0.1556
CV-102	0.6727	0.3936	0.8177	0.6056	0.5286	0.7220	0.2301
CV-103	0.6691	0.3830	0.8177	0.6003	0.5217	0.7184	0.2195
CV-104	0.6691	0.3830	0.8177	0.6003	0.5217	0.7184	0.2195
CV-105	0.6691	0.4149	0.8011	0.6080	0.5200	0.7250	0.2300
CV-106	0.6364	0.3617	0.7790	0.5704	0.4595	0.7015	0.1505
CV-107	0.6873	0.4362	0.8177	0.6269	0.5541	0.7363	0.2715

CV-108	0.6800	0.4043	0.8232	0.6137	0.5429	0.7268	0.2477
CV-109	0.6691	0.4468	0.7845	0.6157	0.5185	0.7320	0.2407
CV-110	0.6655	0.3404	0.8343	0.5873	0.5161	0.7089	0.1983
CV-111	0.7018	0.4468	0.8343	0.6405	0.5833	0.7438	0.3032
CV-112	0.6509	0.3936	0.7845	0.5891	0.4868	0.7136	0.1890
CV-113	0.6473	0.3298	0.8122	0.5710	0.4769	0.7000	0.1585
CV-114	0.6655	0.4043	0.8011	0.6027	0.5135	0.7214	0.2196
CV-115	0.6327	0.3511	0.7790	0.5650	0.4521	0.6980	0.1397
CV-116	0.6655	0.4149	0.7956	0.6052	0.5132	0.7236	0.2232
CV-117	0.7127	0.5106	0.8177	0.6642	0.5926	0.7629	0.3416
CV-118	0.6945	0.4468	0.8232	0.6350	0.5676	0.7413	0.2888
CV-119	0.6073	0.3830	0.7238	0.5534	0.4186	0.6931	0.1092
CV-120	0.6473	0.3617	0.7956	0.5786	0.4789	0.7059	0.1705
CV-121	0.6727	0.3936	0.8177	0.6056	0.5286	0.7220	0.2301
CV-122	0.6727	0.4255	0.8011	0.6133	0.5263	0.7286	0.2404
CV-123	0.6727	0.4255	0.8011	0.6133	0.5263	0.7286	0.2404
CV-124	0.6473	0.4149	0.7680	0.5914	0.4815	0.7165	0.1903
CV-125	0.6327	0.3511	0.7790	0.5650	0.4521	0.6980	0.1397
CV-126	0.6545	0.3723	0.8011	0.5867	0.4930	0.7108	0.1880
CV-127	0.6436	0.3617	0.7901	0.5759	0.4722	0.7044	0.1637
CV-128	0.6145	0.2979	0.7790	0.5384	0.4118	0.6812	0.0845
CV-129	0.6873	0.4149	0.8287	0.6218	0.5571	0.7317	0.2653
CV-130	0.7018	0.4574	0.8287	0.6431	0.5811	0.7463	0.3061
CV-131	0.6945	0.4468	0.8232	0.6350	0.5676	0.7413	0.2888
CV-132	0.6909	0.3830	0.8508	0.6169	0.5714	0.7264	0.2639
CV-133	0.6836	0.4149	0.8232	0.6190	0.5493	0.7304	0.2581
CV-134	0.6691	0.3936	0.8122	0.6029	0.5211	0.7206	0.2230
CV-135	0.6945	0.4149	0.8398	0.6273	0.5735	0.7343	0.2800
CV-136	0.6727	0.3936	0.8177	0.6056	0.5286	0.7220	0.2301
CV-137	0.6473	0.3830	0.7845	0.5838	0.4800	0.7100	0.1784
CV-138	0.6509	0.3936	0.7845	0.5891	0.4868	0.7136	0.1890
CV-139	0.6545	0.3298	0.8232	0.5765	0.4921	0.7028	0.1727
CV-140	0.6836	0.4149	0.8232	0.6190	0.5493	0.7304	0.2581
CV-141	0.6582	0.4468	0.7680	0.6074	0.5000	0.7277	0.2212
CV-142	0.7236	0.4468	0.8674	0.6571	0.6364	0.7512	0.3490
CV-143	0.6545	0.4255	0.7735	0.5995	0.4938	0.7216	0.2071
CV-144	0.6218	0.3085	0.7845	0.5465	0.4265	0.6860	0.1023
CV-145	0.6764	0.4362	0.8011	0.6186	0.5325	0.7323	0.2507
CV-146	0.6691	0.4149	0.8011	0.6080	0.5200	0.7250	0.2300
CV-147	0.6545	0.3723	0.8011	0.5867	0.4930	0.7108	0.1880
CV-148	0.6873	0.3830	0.8453	0.6141	0.5625	0.7251	0.2562
CV-149	0.6764	0.4149	0.8122	0.6135	0.5342	0.7277	0.2439
CV-150	0.6691	0.3936	0.8122	0.6029	0.5211	0.7206	0.2230
CV-151	0.6618	0.3936	0.8011	0.5974	0.5068	0.7178	0.2092
CV-152	0.6982	0.4043	0.8508	0.6275	0.5846	0.7333	0.2848
CV-153	0.6691	0.3936	0.8122	0.6029	0.5211	0.7206	0.2230
CV-154	0.6873	0.4043	0.8343	0.6193	0.5588	0.7295	0.2622
CV-155	0.6909	0.4255	0.8287	0.6271	0.5634	0.7353	0.2756
CV-156	0.6764	0.3936	0.8232	0.6084	0.5362	0.7233	0.2372
CV-157	0.6836	0.4681	0.7956	0.6318	0.5432	0.7423	0.2744
CV-158	0.6691	0.3723	0.8232	0.5978	0.5224	0.7163	0.2161
CV-159	0.6473	0.4362	0.7569	0.5965	0.4824	0.7211	0.1982
CV-160	0.6727	0.4468	0.7901	0.6184	0.5250	0.7333	0.2474
CV-161	0.6909	0.3830	0.8508	0.6169	0.5714	0.7264	0.2639
CV-162	0.6473	0.4468	0.7514	0.5991	0.4828	0.7234	0.2021
CV-163	0.6545	0.3511	0.8122	0.5816	0.4925	0.7067	0.1803
CV-164	0.6509	0.3830	0.7901	0.5865	0.4865	0.7114	0.1851

CV-165	0.6909	0.4787	0.8011	0.6399	0.5556	0.7474	0.2912
CV-166	0.6655	0.3723	0.8177	0.5950	0.5147	0.7150	0.2089
CV-167	0.6545	0.4468	0.7624	0.6046	0.4941	0.7263	0.2148
CV-168	0.6545	0.3511	0.8122	0.5816	0.4925	0.7067	0.1803
CV-169	0.6764	0.3936	0.8232	0.6084	0.5362	0.7233	0.2372
CV-170	0.6727	0.3830	0.8232	0.6031	0.5294	0.7198	0.2267
CV-171	0.6909	0.4255	0.8287	0.6271	0.5634	0.7353	0.2756
CV-172	0.6182	0.3830	0.7403	0.5617	0.4337	0.6979	0.1274
CV-173	0.6473	0.4255	0.7624	0.5940	0.4819	0.7188	0.1942
CV-174	0.6582	0.4149	0.7845	0.5997	0.5000	0.7208	0.2098
CV-175	0.6327	0.3936	0.7569	0.5753	0.4568	0.7062	0.1566
CV-176	0.6655	0.4787	0.7624	0.6206	0.5114	0.7380	0.2452
CV-177	0.6727	0.3830	0.8232	0.6031	0.5294	0.7198	0.2267
CV-178	0.6255	0.3936	0.7459	0.5697	0.4458	0.7031	0.1441
CV-179	0.6727	0.3191	0.8564	0.5878	0.5357	0.7078	0.2067
CV-180	0.6764	0.3936	0.8232	0.6084	0.5362	0.7233	0.2372
CV-181	0.6727	0.4362	0.7956	0.6159	0.5256	0.7310	0.2439
CV-182	0.6982	0.4043	0.8508	0.6275	0.5846	0.7333	0.2848
CV-183	0.6582	0.3723	0.8066	0.5895	0.5000	0.7122	0.1949
CV-184	0.6836	0.3723	0.8453	0.6088	0.5556	0.7217	0.2456
CV-185	0.6509	0.3191	0.8232	0.5712	0.4839	0.6995	0.1616
CV-186	0.6473	0.3830	0.7845	0.5838	0.4800	0.7100	0.1784
CV-187	0.6764	0.3936	0.8232	0.6084	0.5362	0.7233	0.2372
CV-188	0.6873	0.4255	0.8232	0.6244	0.5556	0.7340	0.2684
CV-189	0.6945	0.4043	0.8453	0.6248	0.5758	0.7321	0.2772
CV-190	0.7055	0.4894	0.8177	0.6535	0.5823	0.7551	0.3219
CV-191	0.6400	0.3723	0.7790	0.5757	0.4667	0.7050	0.1612
CV-192	0.6582	0.3936	0.7956	0.5946	0.5000	0.7164	0.2024
CV-193	0.6836	0.3723	0.8453	0.6088	0.5556	0.7217	0.2456
CV-194	0.6509	0.3298	0.8177	0.5737	0.4844	0.7014	0.1655
CV-195	0.6727	0.4362	0.7956	0.6159	0.5256	0.7310	0.2439
CV-196	0.6618	0.3936	0.8011	0.5974	0.5068	0.7178	0.2092
CV-197	0.6582	0.4149	0.7845	0.5997	0.5000	0.7208	0.2098
CV-198	0.6764	0.4149	0.8122	0.6135	0.5342	0.7277	0.2439
CV-199	0.6836	0.5000	0.7790	0.6395	0.5402	0.7500	0.2846
CV-200	0.6836	0.4362	0.8122	0.6242	0.5467	0.7350	0.2645
CV-201	0.6436	0.4149	0.7624	0.5887	0.4756	0.7150	0.1839
CV-202	0.6691	0.4894	0.7624	0.6259	0.5169	0.7419	0.2553
CV-203	0.6727	0.4362	0.7956	0.6159	0.5256	0.7310	0.2439
CV-204	0.6182	0.4255	0.7182	0.5719	0.4396	0.7065	0.1449
CV-205	0.6582	0.3617	0.8122	0.5869	0.5000	0.7101	0.1911
CV-206	0.6873	0.4043	0.8343	0.6193	0.5588	0.7295	0.2622
CV-207	0.6727	0.4149	0.8066	0.6108	0.5270	0.7264	0.2369
CV-208	0.6727	0.4043	0.8122	0.6082	0.5278	0.7241	0.2335
CV-209	0.6873	0.4362	0.8177	0.6269	0.5541	0.7363	0.2715
CV-210	0.6364	0.3511	0.7845	0.5678	0.4583	0.6995	0.1463
CV-211	0.6618	0.3830	0.8066	0.5948	0.5070	0.7157	0.2055
CV-212	0.6109	0.2872	0.7790	0.5331	0.4030	0.6779	0.0732
CV-213	0.6727	0.4255	0.8011	0.6133	0.5263	0.7286	0.2404
CV-214	0.6618	0.4043	0.7956	0.5999	0.5067	0.7200	0.2128
CV-215	0.6909	0.4043	0.8398	0.6220	0.5672	0.7308	0.2696
CV-216	0.6545	0.4255	0.7735	0.5995	0.4938	0.7216	0.2071
CV-217	0.6727	0.3723	0.8287	0.6005	0.5303	0.7177	0.2233
CV-218	0.6473	0.3617	0.7956	0.5786	0.4789	0.7059	0.1705
CV-219	0.6982	0.4043	0.8508	0.6275	0.5846	0.7333	0.2848
CV-220	0.6800	0.3830	0.8343	0.6086	0.5455	0.7225	0.2413
CV-221	0.6655	0.3298	0.8398	0.5848	0.5167	0.7070	0.1947

CV-222	0.6473	0.3298	0.8122	0.5710	0.4769	0.7000	0.1585
CV-223	0.6255	0.3404	0.7735	0.5570	0.4384	0.6931	0.1224
CV-224	0.6618	0.3511	0.8232	0.5871	0.5077	0.7095	0.1946
CV-225	0.6691	0.3404	0.8398	0.5901	0.5246	0.7103	0.2057
CV-226	0.6509	0.3404	0.8122	0.5763	0.4848	0.7033	0.1695
CV-227	0.6655	0.4468	0.7790	0.6129	0.5122	0.7306	0.2341
CV-228	0.6509	0.4043	0.7790	0.5916	0.4872	0.7157	0.1928
CV-229	0.6764	0.3830	0.8287	0.6059	0.5373	0.7212	0.2339
CV-230	0.6436	0.3723	0.7845	0.5784	0.4730	0.7065	0.1678
CV-231	0.6800	0.4255	0.8122	0.6188	0.5405	0.7313	0.2542
CV-232	0.6764	0.3404	0.8508	0.5956	0.5424	0.7130	0.2210
CV-233	0.6618	0.3191	0.8398	0.5795	0.5085	0.7037	0.1836
CV-234	0.6764	0.3723	0.8343	0.6033	0.5385	0.7190	0.2307
CV-235	0.6582	0.3830	0.8011	0.5920	0.5000	0.7143	0.1986
CV-236	0.6400	0.3723	0.7790	0.5757	0.4667	0.7050	0.1612
CV-237	0.6255	0.3511	0.7680	0.5595	0.4400	0.6950	0.1268
CV-238	0.6509	0.4255	0.7680	0.5967	0.4878	0.7202	0.2006
CV-239	0.6618	0.3830	0.8066	0.5948	0.5070	0.7157	0.2055
CV-240	0.6582	0.3298	0.8287	0.5793	0.5000	0.7042	0.1799
CV-241	0.6436	0.3511	0.7956	0.5733	0.4714	0.7024	0.1597
CV-242	0.6873	0.4149	0.8287	0.6218	0.5571	0.7317	0.2653
CV-243	0.6691	0.3936	0.8122	0.6029	0.5211	0.7206	0.2230
CV-244	0.6945	0.4255	0.8343	0.6299	0.5714	0.7366	0.2829
CV-245	0.6509	0.3830	0.7901	0.5865	0.4865	0.7114	0.1851
CV-246	0.6582	0.3617	0.8122	0.5869	0.5000	0.7101	0.1911
CV-247	0.6618	0.3617	0.8177	0.5897	0.5075	0.7115	0.1982
CV-248	0.7164	0.5000	0.8287	0.6644	0.6026	0.7614	0.3459
CV-249	0.6618	0.3830	0.8066	0.5948	0.5070	0.7157	0.2055
CV-250	0.6836	0.4468	0.8066	0.6267	0.5455	0.7374	0.2677
CV-251	0.6400	0.3191	0.8066	0.5629	0.4615	0.6952	0.1404
CV-252	0.7127	0.5000	0.8232	0.6616	0.5949	0.7602	0.3388
CV-253	0.6582	0.3723	0.8066	0.5895	0.5000	0.7122	0.1949
CV-254	0.6400	0.3298	0.8011	0.5654	0.4627	0.6971	0.1446
CV-255	0.6691	0.3723	0.8232	0.5978	0.5224	0.7163	0.2161
CV-256	0.6982	0.4681	0.8177	0.6429	0.5714	0.7475	0.3019
CV-257	0.6582	0.4149	0.7845	0.5997	0.5000	0.7208	0.2098
CV-258	0.6327	0.3298	0.7901	0.5599	0.4493	0.6942	0.1311
CV-259	0.6727	0.3830	0.8232	0.6031	0.5294	0.7198	0.2267
CV-260	0.6582	0.3404	0.8232	0.5818	0.5000	0.7062	0.1837
CV-261	0.6909	0.3617	0.8619	0.6118	0.5763	0.7222	0.2583
CV-262	0.6727	0.4149	0.8066	0.6108	0.5270	0.7264	0.2369
CV-263	0.7127	0.4149	0.8674	0.6411	0.6190	0.7406	0.3186
CV-264	0.6582	0.3936	0.7956	0.5946	0.5000	0.7164	0.2024
CV-265	0.6509	0.3936	0.7845	0.5891	0.4868	0.7136	0.1890
CV-266	0.6945	0.4362	0.8287	0.6324	0.5694	0.7389	0.2858
CV-267	0.6473	0.3830	0.7845	0.5838	0.4800	0.7100	0.1784
CV-268	0.6909	0.4362	0.8232	0.6297	0.5616	0.7376	0.2786
CV-269	0.6727	0.3617	0.8343	0.5980	0.5313	0.7156	0.2200
CV-270	0.6618	0.4149	0.7901	0.6025	0.5065	0.7222	0.2165
CV-271	0.6727	0.4149	0.8066	0.6108	0.5270	0.7264	0.2369
CV-272	0.6436	0.3617	0.7901	0.5759	0.4722	0.7044	0.1637
CV-273	0.6836	0.4255	0.8177	0.6216	0.5479	0.7327	0.2612
CV-274	0.6909	0.4362	0.8232	0.6297	0.5616	0.7376	0.2786
CV-275	0.6218	0.4255	0.7238	0.5746	0.4444	0.7081	0.1509
CV-276	0.6727	0.3936	0.8177	0.6056	0.5286	0.7220	0.2301
CV-277	0.6545	0.3936	0.7901	0.5918	0.4933	0.7150	0.1956
CV-278	0.6909	0.4787	0.8011	0.6399	0.5556	0.7474	0.2912

CV-279	0.6764	0.3404	0.8508	0.5956	0.5424	0.7130	0.2210
CV-280	0.6218	0.3298	0.7735	0.5516	0.4306	0.6897	0.1114
CV-281	0.6800	0.4149	0.8177	0.6163	0.5417	0.7291	0.2509
CV-282	0.6764	0.4043	0.8177	0.6110	0.5352	0.7255	0.2405
CV-283	0.6691	0.4468	0.7845	0.6157	0.5185	0.7320	0.2407
CV-284	0.6545	0.3936	0.7901	0.5918	0.4933	0.7150	0.1956
CV-285	0.6982	0.4149	0.8453	0.6301	0.5821	0.7356	0.2875
CV-286	0.6618	0.4043	0.7956	0.5999	0.5067	0.7200	0.2128
CV-287	0.6545	0.4255	0.7735	0.5995	0.4938	0.7216	0.2071
CV-288	0.6691	0.3936	0.8122	0.6029	0.5211	0.7206	0.2230
CV-289	0.6982	0.4362	0.8343	0.6352	0.5775	0.7402	0.2931
CV-290	0.7018	0.4255	0.8453	0.6354	0.5882	0.7391	0.2978
CV-291	0.6509	0.2766	0.8453	0.5609	0.4815	0.6923	0.1456
CV-292	0.6473	0.3830	0.7845	0.5838	0.4800	0.7100	0.1784
CV-293	0.6473	0.4149	0.7680	0.5914	0.4815	0.7165	0.1903
CV-294	0.6255	0.3830	0.7514	0.5672	0.4444	0.7010	0.1398
CV-295	0.6327	0.3404	0.7845	0.5625	0.4507	0.6961	0.1354
CV-296	0.6255	0.3085	0.7901	0.5493	0.4328	0.6875	0.1089
CV-297	0.6655	0.3936	0.8066	0.6001	0.5139	0.7192	0.2161
CV-298	0.6436	0.4043	0.7680	0.5861	0.4750	0.7128	0.1798
CV-299	0.6218	0.3830	0.7459	0.5644	0.4390	0.6995	0.1336
CV-300	0.6436	0.3830	0.7790	0.5810	0.4737	0.7085	0.1718
CV-301	0.6509	0.3723	0.7956	0.5840	0.4861	0.7094	0.1812
CV-302	0.6764	0.4681	0.7845	0.6263	0.5301	0.7396	0.2610
CV-303	0.6764	0.4574	0.7901	0.6238	0.5309	0.7371	0.2575
CV-304	0.6618	0.4043	0.7956	0.5999	0.5067	0.7200	0.2128
CV-305	0.6909	0.4574	0.8122	0.6348	0.5584	0.7424	0.2848
CV-306	0.6727	0.4255	0.8011	0.6133	0.5263	0.7286	0.2404
CV-307	0.6873	0.3936	0.8398	0.6167	0.5606	0.7273	0.2592
CV-308	0.6800	0.3936	0.8287	0.6112	0.5441	0.7246	0.2445
CV-309	0.6873	0.4362	0.8177	0.6269	0.5541	0.7363	0.2715
CV-310	0.6364	0.3085	0.8066	0.5576	0.4531	0.6919	0.1292
CV-311	0.6618	0.3723	0.8122	0.5922	0.5072	0.7136	0.2019
CV-312	0.6473	0.4149	0.7680	0.5914	0.4815	0.7165	0.1903
CV-313	0.6618	0.3830	0.8066	0.5948	0.5070	0.7157	0.2055
CV-314	0.6909	0.4255	0.8287	0.6271	0.5634	0.7353	0.2756
CV-315	0.6509	0.3617	0.8011	0.5814	0.4857	0.7073	0.1773
CV-316	0.6873	0.4574	0.8066	0.6320	0.5513	0.7411	0.2779
CV-317	0.6255	0.2979	0.7956	0.5467	0.4308	0.6857	0.1043
CV-318	0.6691	0.3404	0.8398	0.5901	0.5246	0.7103	0.2057
CV-319	0.6218	0.3511	0.7624	0.5567	0.4342	0.6935	0.1204
CV-320	0.6691	0.4787	0.7680	0.6233	0.5172	0.7394	0.2516
CV-321	0.6655	0.3511	0.8287	0.5899	0.5156	0.7109	0.2018
CV-322	0.6909	0.4149	0.8343	0.6246	0.5652	0.7330	0.2726
CV-323	0.6982	0.4362	0.8343	0.6352	0.5775	0.7402	0.2931
CV-324	0.6764	0.3617	0.8398	0.6007	0.5397	0.7170	0.2274
CV-325	0.6982	0.3936	0.8564	0.6250	0.5873	0.7311	0.2821
CV-326	0.6909	0.4043	0.8398	0.6220	0.5672	0.7308	0.2696
CV-327	0.6655	0.3298	0.8398	0.5848	0.5167	0.7070	0.1947
CV-328	0.6800	0.4149	0.8177	0.6163	0.5417	0.7291	0.2509
CV-329	0.6545	0.3511	0.8122	0.5816	0.4925	0.7067	0.1803
CV-330	0.6582	0.4681	0.7569	0.6125	0.5000	0.7326	0.2288
CV-331	0.6509	0.3830	0.7901	0.5865	0.4865	0.7114	0.1851
CV-332	0.6836	0.4149	0.8232	0.6190	0.5493	0.7304	0.2581
CV-333	0.6473	0.3830	0.7845	0.5838	0.4800	0.7100	0.1784
CV-334	0.6800	0.4362	0.8066	0.6214	0.5395	0.7337	0.2575
CV-335	0.6727	0.4043	0.8122	0.6082	0.5278	0.7241	0.2335

CV-336	0.6727	0.3936	0.8177	0.6056	0.5286	0.7220	0.2301
CV-337	0.6764	0.3936	0.8232	0.6084	0.5362	0.7233	0.2372
CV-338	0.6545	0.3511	0.8122	0.5816	0.4925	0.7067	0.1803
CV-339	0.6655	0.4149	0.7956	0.6052	0.5132	0.7236	0.2232
CV-340	0.6073	0.2872	0.7735	0.5304	0.3971	0.6763	0.0668
CV-341	0.6509	0.3936	0.7845	0.5891	0.4868	0.7136	0.1890
CV-342	0.6618	0.3511	0.8232	0.5871	0.5077	0.7095	0.1946
CV-343	0.6800	0.4043	0.8232	0.6137	0.5429	0.7268	0.2477
CV-344	0.6545	0.3511	0.8122	0.5816	0.4925	0.7067	0.1803
CV-345	0.6909	0.4681	0.8066	0.6374	0.5570	0.7449	0.2880
CV-346	0.6873	0.3936	0.8398	0.6167	0.5606	0.7273	0.2592
CV-347	0.6982	0.4574	0.8232	0.6403	0.5733	0.7450	0.2989
CV-348	0.6509	0.3830	0.7901	0.5865	0.4865	0.7114	0.1851
CV-349	0.6545	0.3511	0.8122	0.5816	0.4925	0.7067	0.1803
CV-350	0.6691	0.4149	0.8011	0.6080	0.5200	0.7250	0.2300
CV-351	0.6727	0.3404	0.8453	0.5929	0.5333	0.7116	0.2133
CV-352	0.6873	0.3723	0.8508	0.6116	0.5645	0.7230	0.2533
CV-353	0.6691	0.4255	0.7956	0.6106	0.5195	0.7273	0.2336
CV-354	0.6800	0.3617	0.8453	0.6035	0.5484	0.7183	0.2350
CV-355	0.6764	0.4362	0.8011	0.6186	0.5325	0.7323	0.2507
CV-356	0.6800	0.4468	0.8011	0.6240	0.5385	0.7360	0.2609
CV-357	0.6800	0.4574	0.7956	0.6265	0.5375	0.7385	0.2642
CV-358	0.6436	0.3830	0.7790	0.5810	0.4737	0.7085	0.1718
CV-359	0.6618	0.4043	0.7956	0.5999	0.5067	0.7200	0.2128
CV-360	0.7091	0.5319	0.8011	0.6665	0.5814	0.7672	0.3407
CV-361	0.6473	0.4255	0.7624	0.5940	0.4819	0.7188	0.1942
CV-362	0.6509	0.3617	0.8011	0.5814	0.4857	0.7073	0.1773
CV-363	0.6691	0.4574	0.7790	0.6182	0.5181	0.7344	0.2443
CV-364	0.6655	0.3404	0.8343	0.5873	0.5161	0.7089	0.1983
CV-365	0.6727	0.4255	0.8011	0.6133	0.5263	0.7286	0.2404
CV-366	0.6400	0.3723	0.7790	0.5757	0.4667	0.7050	0.1612
CV-367	0.6400	0.3723	0.7790	0.5757	0.4667	0.7050	0.1612
CV-368	0.6436	0.3404	0.8011	0.5708	0.4706	0.7005	0.1556
CV-369	0.7164	0.4574	0.8508	0.6541	0.6143	0.7512	0.3357
CV-370	0.6618	0.4468	0.7735	0.6101	0.5060	0.7292	0.2276
CV-371	0.6473	0.4043	0.7735	0.5889	0.4810	0.7143	0.1863
CV-372	0.6727	0.4362	0.7956	0.6159	0.5256	0.7310	0.2439
CV-373	0.6836	0.4468	0.8066	0.6267	0.5455	0.7374	0.2677
CV-374	0.6873	0.4681	0.8011	0.6346	0.5500	0.7436	0.2811
CV-375	0.6436	0.3830	0.7790	0.5810	0.4737	0.7085	0.1718
CV-376	0.6545	0.4149	0.7790	0.5969	0.4937	0.7194	0.2033
CV-377	0.6655	0.3830	0.8122	0.5976	0.5143	0.7171	0.2125
CV-378	0.6364	0.3511	0.7845	0.5678	0.4583	0.6995	0.1463
CV-379	0.6836	0.3830	0.8398	0.6114	0.5538	0.7238	0.2487
CV-380	0.7091	0.4681	0.8343	0.6512	0.5946	0.7512	0.3234
CV-381	0.6545	0.4362	0.7680	0.6021	0.4940	0.7240	0.2109
CV-382	0.6545	0.4149	0.7790	0.5969	0.4937	0.7194	0.2033
CV-383	0.6982	0.3936	0.8564	0.6250	0.5873	0.7311	0.2821
CV-384	0.6509	0.3511	0.8066	0.5788	0.4853	0.7053	0.1734
CV-385	0.6509	0.4043	0.7790	0.5916	0.4872	0.7157	0.1928
CV-386	0.6545	0.3191	0.8287	0.5739	0.4918	0.7009	0.1688
CV-387	0.6473	0.3617	0.7956	0.5786	0.4789	0.7059	0.1705
CV-388	0.6509	0.4255	0.7680	0.5967	0.4878	0.7202	0.2006
CV-389	0.6618	0.4255	0.7845	0.6050	0.5063	0.7245	0.2202
CV-390	0.6727	0.3936	0.8177	0.6056	0.5286	0.7220	0.2301
CV-391	0.6400	0.3830	0.7735	0.5782	0.4675	0.7071	0.1653
CV-392	0.6291	0.3404	0.7790	0.5597	0.4444	0.6946	0.1289

CV-393	0.6545	0.3511	0.8122	0.5816	0.4925	0.7067	0.1803
CV-394	0.6764	0.3936	0.8232	0.6084	0.5362	0.7233	0.2372
CV-395	0.6509	0.3723	0.7956	0.5840	0.4861	0.7094	0.1812
CV-396	0.6582	0.3936	0.7956	0.5946	0.5000	0.7164	0.2024
CV-397	0.6655	0.3511	0.8287	0.5899	0.5156	0.7109	0.2018
CV-398	0.6764	0.3936	0.8232	0.6084	0.5362	0.7233	0.2372
CV-399	0.6364	0.3830	0.7680	0.5755	0.4615	0.7056	0.1588
CV-400	0.6327	0.3511	0.7790	0.5650	0.4521	0.6980	0.1397
CV-401	0.6473	0.3723	0.7901	0.5812	0.4795	0.7079	0.1744
CV-402	0.6800	0.3936	0.8287	0.6112	0.5441	0.7246	0.2445
CV-403	0.6655	0.3511	0.8287	0.5899	0.5156	0.7109	0.2018
CV-404	0.6436	0.4255	0.7569	0.5912	0.4762	0.7173	0.1879
CV-405	0.7127	0.4681	0.8398	0.6539	0.6027	0.7525	0.3307
CV-406	0.6545	0.3617	0.8066	0.5842	0.4928	0.7087	0.1842
CV-407	0.6727	0.4787	0.7735	0.6261	0.5233	0.7407	0.2580
CV-408	0.6655	0.4362	0.7845	0.6104	0.5125	0.7282	0.2305
CV-409	0.6545	0.4574	0.7569	0.6072	0.4943	0.7287	0.2186
CV-410	0.6582	0.4149	0.7845	0.5997	0.5000	0.7208	0.2098
CV-411	0.6873	0.4894	0.7901	0.6397	0.5476	0.7487	0.2877
CV-412	0.6545	0.3298	0.8232	0.5765	0.4921	0.7028	0.1727
CV-413	0.6255	0.3511	0.7680	0.5595	0.4400	0.6950	0.1268
CV-414	0.6982	0.3936	0.8564	0.6250	0.5873	0.7311	0.2821
CV-415	0.6545	0.4362	0.7680	0.6021	0.4940	0.7240	0.2109
CV-416	0.6582	0.4043	0.7901	0.5972	0.5000	0.7186	0.2061
CV-417	0.6873	0.4149	0.8287	0.6218	0.5571	0.7317	0.2653
CV-418	0.6473	0.3723	0.7901	0.5812	0.4795	0.7079	0.1744
CV-419	0.6727	0.4255	0.8011	0.6133	0.5263	0.7286	0.2404
CV-420	0.6800	0.4574	0.7956	0.6265	0.5375	0.7385	0.2642
CV-421	0.6291	0.3511	0.7735	0.5623	0.4459	0.6965	0.1332
CV-422	0.6909	0.4149	0.8343	0.6246	0.5652	0.7330	0.2726
CV-423	0.6655	0.4255	0.7901	0.6078	0.5128	0.7259	0.2269
CV-424	0.6691	0.3617	0.8287	0.5952	0.5231	0.7143	0.2126
CV-425	0.6582	0.3617	0.8122	0.5869	0.5000	0.7101	0.1911
CV-426	0.6800	0.3830	0.8343	0.6086	0.5455	0.7225	0.2413
CV-427	0.6582	0.3511	0.8177	0.5844	0.5000	0.7081	0.1874
CV-428	0.6691	0.4149	0.8011	0.6080	0.5200	0.7250	0.2300
CV-429	0.6691	0.3936	0.8122	0.6029	0.5211	0.7206	0.2230
CV-430	0.6436	0.3830	0.7790	0.5810	0.4737	0.7085	0.1718
CV-431	0.6509	0.3723	0.7956	0.5840	0.4861	0.7094	0.1812
CV-432	0.6545	0.3511	0.8122	0.5816	0.4925	0.7067	0.1803
CV-433	0.7018	0.4681	0.8232	0.6456	0.5789	0.7487	0.3090
CV-434	0.6545	0.3298	0.8232	0.5765	0.4921	0.7028	0.1727
CV-435	0.6800	0.4894	0.7790	0.6342	0.5349	0.7460	0.2746
CV-436	0.6618	0.4362	0.7790	0.6076	0.5062	0.7268	0.2239
CV-437	0.6291	0.2872	0.8066	0.5469	0.4355	0.6854	0.1065
CV-438	0.6909	0.4681	0.8066	0.6374	0.5570	0.7449	0.2880
CV-439	0.6618	0.3936	0.8011	0.5974	0.5068	0.7178	0.2092
CV-440	0.6364	0.3511	0.7845	0.5678	0.4583	0.6995	0.1463
CV-441	0.6764	0.4149	0.8122	0.6135	0.5342	0.7277	0.2439
CV-442	0.6473	0.4043	0.7735	0.5889	0.4810	0.7143	0.1863
CV-443	0.6582	0.3936	0.7956	0.5946	0.5000	0.7164	0.2024
CV-444	0.6873	0.4468	0.8122	0.6295	0.5526	0.7387	0.2747
CV-445	0.6836	0.4362	0.8122	0.6242	0.5467	0.7350	0.2645
CV-446	0.6873	0.4149	0.8287	0.6218	0.5571	0.7317	0.2653
CV-447	0.6909	0.4362	0.8232	0.6297	0.5616	0.7376	0.2786
CV-448	0.6582	0.3511	0.8177	0.5844	0.5000	0.7081	0.1874
CV-449	0.6582	0.4043	0.7901	0.5972	0.5000	0.7186	0.2061

CV-450	0.6764	0.4149	0.8122	0.6135	0.5342	0.7277	0.2439
CV-451	0.7018	0.4255	0.8453	0.6354	0.5882	0.7391	0.2978
CV-452	0.6691	0.4468	0.7845	0.6157	0.5185	0.7320	0.2407
CV-453	0.6509	0.4362	0.7624	0.5993	0.4881	0.7225	0.2045
CV-454	0.6727	0.3723	0.8287	0.6005	0.5303	0.7177	0.2233
CV-455	0.6873	0.4149	0.8287	0.6218	0.5571	0.7317	0.2653
CV-456	0.6800	0.4255	0.8122	0.6188	0.5405	0.7313	0.2542
CV-457	0.6400	0.2872	0.8232	0.5552	0.4576	0.6898	0.1276
CV-458	0.7055	0.4468	0.8398	0.6433	0.5915	0.7451	0.3106
CV-459	0.6655	0.3723	0.8177	0.5950	0.5147	0.7150	0.2089
CV-460	0.6218	0.3404	0.7680	0.5542	0.4324	0.6915	0.1159
CV-461	0.6909	0.4362	0.8232	0.6297	0.5616	0.7376	0.2786
CV-462	0.6618	0.3617	0.8177	0.5897	0.5075	0.7115	0.1982
CV-463	0.6509	0.3723	0.7956	0.5840	0.4861	0.7094	0.1812
CV-464	0.6400	0.4149	0.7569	0.5859	0.4699	0.7135	0.1775
CV-465	0.6873	0.4468	0.8122	0.6295	0.5526	0.7387	0.2747
CV-466	0.6291	0.3830	0.7569	0.5699	0.4500	0.7026	0.1461
CV-467	0.6364	0.3191	0.8011	0.5601	0.4545	0.6938	0.1336
CV-468	0.6436	0.3404	0.8011	0.5708	0.4706	0.7005	0.1556
CV-469	0.6545	0.3617	0.8066	0.5842	0.4928	0.7087	0.1842
CV-470	0.6691	0.3936	0.8122	0.6029	0.5211	0.7206	0.2230
CV-471	0.6945	0.3936	0.8508	0.6222	0.5781	0.7299	0.2744
CV-472	0.7018	0.4468	0.8343	0.6405	0.5833	0.7438	0.3032
CV-473	0.6618	0.3830	0.8066	0.5948	0.5070	0.7157	0.2055
CV-474	0.6982	0.3723	0.8674	0.6199	0.5932	0.7269	0.2770
CV-475	0.6436	0.3936	0.7735	0.5835	0.4744	0.7107	0.1758
CV-476	0.6400	0.3830	0.7735	0.5782	0.4675	0.7071	0.1653
CV-477	0.7164	0.4681	0.8453	0.6567	0.6111	0.7537	0.3381
CV-478	0.6836	0.3936	0.8343	0.6139	0.5522	0.7260	0.2518
CV-479	0.6764	0.3723	0.8343	0.6033	0.5385	0.7190	0.2307
CV-480	0.7091	0.5000	0.8177	0.6588	0.5875	0.7590	0.3318
CV-481	0.6655	0.3511	0.8287	0.5899	0.5156	0.7109	0.2018
CV-482	0.6618	0.4043	0.7956	0.5999	0.5067	0.7200	0.2128
CV-483	0.6727	0.4149	0.8066	0.6108	0.5270	0.7264	0.2369
CV-484	0.7055	0.4149	0.8564	0.6356	0.6000	0.7381	0.3028
CV-485	0.6909	0.4043	0.8398	0.6220	0.5672	0.7308	0.2696
CV-486	0.6836	0.3723	0.8453	0.6088	0.5556	0.7217	0.2456
CV-487	0.6691	0.4574	0.7790	0.6182	0.5181	0.7344	0.2443
CV-488	0.6473	0.3936	0.7790	0.5863	0.4805	0.7121	0.1824
CV-489	0.6582	0.4149	0.7845	0.5997	0.5000	0.7208	0.2098
CV-490	0.6873	0.4149	0.8287	0.6218	0.5571	0.7317	0.2653
CV-491	0.6800	0.4255	0.8122	0.6188	0.5405	0.7313	0.2542
CV-492	0.6909	0.4574	0.8122	0.6348	0.5584	0.7424	0.2848
CV-493	0.6909	0.3723	0.8564	0.6143	0.5738	0.7243	0.2611
CV-494	0.6400	0.3617	0.7845	0.5731	0.4658	0.7030	0.1571
CV-495	0.6727	0.3830	0.8232	0.6031	0.5294	0.7198	0.2267
CV-496	0.6145	0.2872	0.7845	0.5359	0.4091	0.6794	0.0797
CV-497	0.6582	0.4043	0.7901	0.5972	0.5000	0.7186	0.2061
CV-498	0.6400	0.3936	0.7680	0.5808	0.4684	0.7092	0.1694
CV-499	0.6218	0.3404	0.7680	0.5542	0.4324	0.6915	0.1159
CV-500	0.6873	0.4043	0.8343	0.6193	0.5588	0.7295	0.2622

Table S6. Performance of 5-fold cross-validations from random forest (RF). Performance of 5-fold cross-validations were evaluated by seven performance metrics. Each row shows the performance of one time 5-fold cross-validation. 5-fold cross-validation was repeated 500 times (CV-1 ~ CV-500).

Cross-validation	Accuracy	Sensitivity	Specificity	Balanced accuracy	Positive prediction rate	Negative prediction rate	Matthews correlation coefficient
CV-1	0.6909	0.3617	0.8619	0.6118	0.5763	0.7222	0.2583
CV-2	0.6836	0.3085	0.8785	0.5935	0.5686	0.7098	0.2282
CV-3	0.7236	0.3936	0.8950	0.6443	0.6607	0.7397	0.3400
CV-4	0.7055	0.3723	0.8785	0.6254	0.6140	0.7294	0.2935
CV-5	0.7273	0.3830	0.9061	0.6445	0.6792	0.7387	0.3476
CV-6	0.7055	0.3723	0.8785	0.6254	0.6140	0.7294	0.2935
CV-7	0.7091	0.3830	0.8785	0.6307	0.6207	0.7327	0.3040
CV-8	0.6691	0.2979	0.8619	0.5799	0.5283	0.7027	0.1921
CV-9	0.7091	0.3511	0.8950	0.6230	0.6346	0.7265	0.2981
CV-10	0.6655	0.3085	0.8508	0.5797	0.5179	0.7032	0.1877
CV-11	0.7018	0.3830	0.8674	0.6252	0.6000	0.7302	0.2875
CV-12	0.6909	0.3404	0.8729	0.6067	0.5818	0.7182	0.2530
CV-13	0.7127	0.3723	0.8895	0.6309	0.6364	0.7318	0.3105
CV-14	0.6982	0.3723	0.8674	0.6199	0.5932	0.7269	0.2770
CV-15	0.7018	0.3404	0.8895	0.6150	0.6154	0.7220	0.2785
CV-16	0.7200	0.3511	0.9116	0.6313	0.6735	0.7301	0.3256
CV-17	0.7164	0.3511	0.9061	0.6286	0.6600	0.7289	0.3162
CV-18	0.7091	0.3298	0.9061	0.6179	0.6458	0.7225	0.2947
CV-19	0.7309	0.4043	0.9006	0.6524	0.6786	0.7443	0.3590
CV-20	0.7018	0.3830	0.8674	0.6252	0.6000	0.7302	0.2875
CV-21	0.7236	0.4149	0.8840	0.6494	0.6500	0.7442	0.3432
CV-22	0.7018	0.3617	0.8785	0.6201	0.6071	0.7260	0.2829
CV-23	0.7018	0.3830	0.8674	0.6252	0.6000	0.7302	0.2875
CV-24	0.6982	0.3298	0.8895	0.6096	0.6078	0.7188	0.2676
CV-25	0.6800	0.3191	0.8674	0.5933	0.5556	0.7104	0.2227
CV-26	0.6800	0.3191	0.8674	0.5933	0.5556	0.7104	0.2227
CV-27	0.6982	0.3298	0.8895	0.6096	0.6078	0.7188	0.2676
CV-28	0.7200	0.3723	0.9006	0.6364	0.6604	0.7342	0.3282
CV-29	0.6800	0.3723	0.8398	0.6061	0.5469	0.7204	0.2381
CV-30	0.6836	0.3191	0.8729	0.5960	0.5660	0.7117	0.2310
CV-31	0.7309	0.3830	0.9116	0.6473	0.6923	0.7399	0.3568
CV-32	0.6982	0.3404	0.8840	0.6122	0.6038	0.7207	0.2698
CV-33	0.7273	0.4149	0.8895	0.6522	0.6610	0.7454	0.3517
CV-34	0.6945	0.3404	0.8785	0.6094	0.5926	0.7195	0.2613
CV-35	0.7055	0.3723	0.8785	0.6254	0.6140	0.7294	0.2935
CV-36	0.7055	0.3723	0.8785	0.6254	0.6140	0.7294	0.2935
CV-37	0.6873	0.3298	0.8729	0.6014	0.5741	0.7149	0.2420
CV-38	0.6836	0.3298	0.8674	0.5986	0.5636	0.7136	0.2338
CV-39	0.7309	0.3936	0.9061	0.6498	0.6852	0.7421	0.3578
CV-40	0.6945	0.3085	0.8950	0.6018	0.6042	0.7137	0.2543
CV-41	0.6909	0.3617	0.8619	0.6118	0.5763	0.7222	0.2583
CV-42	0.7018	0.3191	0.9006	0.6099	0.6250	0.7181	0.2745
CV-43	0.7018	0.3191	0.9006	0.6099	0.6250	0.7181	0.2745
CV-44	0.7018	0.3723	0.8729	0.6226	0.6034	0.7281	0.2852
CV-45	0.7018	0.3298	0.8950	0.6124	0.6200	0.7200	0.2765
CV-46	0.7091	0.3723	0.8840	0.6282	0.6250	0.7306	0.3019
CV-47	0.7018	0.3617	0.8785	0.6201	0.6071	0.7260	0.2829
CV-48	0.7164	0.3830	0.8895	0.6362	0.6429	0.7352	0.3209
CV-49	0.6945	0.3723	0.8619	0.6171	0.5833	0.7256	0.2690
CV-50	0.7236	0.3723	0.9061	0.6392	0.6731	0.7354	0.3372

CV-51	0.7091	0.3617	0.8895	0.6256	0.6296	0.7285	0.2999
CV-52	0.7127	0.3723	0.8895	0.6309	0.6364	0.7318	0.3105
CV-53	0.7018	0.3723	0.8729	0.6226	0.6034	0.7281	0.2852
CV-54	0.7055	0.3830	0.8729	0.6280	0.6102	0.7315	0.2957
CV-55	0.7055	0.4043	0.8619	0.6331	0.6032	0.7358	0.3004
CV-56	0.6836	0.3298	0.8674	0.5986	0.5636	0.7136	0.2338
CV-57	0.6982	0.3298	0.8895	0.6096	0.6078	0.7188	0.2676
CV-58	0.7164	0.3830	0.8895	0.6362	0.6429	0.7352	0.3209
CV-59	0.7200	0.3617	0.9061	0.6339	0.6667	0.7321	0.3268
CV-60	0.6945	0.3511	0.8729	0.6120	0.5893	0.7215	0.2638
CV-61	0.7309	0.3830	0.9116	0.6473	0.6923	0.7399	0.3568
CV-62	0.6764	0.3511	0.8453	0.5982	0.5410	0.7150	0.2242
CV-63	0.6873	0.3191	0.8785	0.5988	0.5769	0.7130	0.2394
CV-64	0.6909	0.3191	0.8840	0.6016	0.5882	0.7143	0.2479
CV-65	0.7055	0.3617	0.8840	0.6228	0.6182	0.7273	0.2913
CV-66	0.6945	0.3511	0.8729	0.6120	0.5893	0.7215	0.2638
CV-67	0.6909	0.3404	0.8729	0.6067	0.5818	0.7182	0.2530
CV-68	0.6945	0.3298	0.8840	0.6069	0.5962	0.7175	0.2589
CV-69	0.7164	0.3511	0.9061	0.6286	0.6600	0.7289	0.3162
CV-70	0.7055	0.3723	0.8785	0.6254	0.6140	0.7294	0.2935
CV-71	0.7164	0.3617	0.9006	0.6311	0.6538	0.7309	0.3177
CV-72	0.6982	0.3830	0.8619	0.6224	0.5902	0.7290	0.2795
CV-73	0.6945	0.3511	0.8729	0.6120	0.5893	0.7215	0.2638
CV-74	0.7200	0.3830	0.8950	0.6390	0.6545	0.7364	0.3297
CV-75	0.6945	0.3617	0.8674	0.6146	0.5862	0.7235	0.2664
CV-76	0.6836	0.3404	0.8619	0.6012	0.5614	0.7156	0.2367
CV-77	0.7273	0.4255	0.8840	0.6548	0.6557	0.7477	0.3534
CV-78	0.7055	0.3298	0.9006	0.6152	0.6327	0.7212	0.2855
CV-79	0.6836	0.3298	0.8674	0.5986	0.5636	0.7136	0.2338
CV-80	0.7055	0.3191	0.9061	0.6126	0.6383	0.7193	0.2838
CV-81	0.7018	0.3723	0.8729	0.6226	0.6034	0.7281	0.2852
CV-82	0.7127	0.3617	0.8950	0.6284	0.6415	0.7297	0.3087
CV-83	0.6909	0.3404	0.8729	0.6067	0.5818	0.7182	0.2530
CV-84	0.7236	0.3617	0.9116	0.6367	0.6800	0.7333	0.3361
CV-85	0.7127	0.3723	0.8895	0.6309	0.6364	0.7318	0.3105
CV-86	0.6836	0.3085	0.8785	0.5935	0.5686	0.7098	0.2282
CV-87	0.6800	0.2766	0.8895	0.5830	0.5652	0.7031	0.2111
CV-88	0.7018	0.3617	0.8785	0.6201	0.6071	0.7260	0.2829
CV-89	0.6982	0.3830	0.8619	0.6224	0.5902	0.7290	0.2795
CV-90	0.6945	0.3511	0.8729	0.6120	0.5893	0.7215	0.2638
CV-91	0.7018	0.3511	0.8840	0.6175	0.6111	0.7240	0.2806
CV-92	0.7200	0.4043	0.8840	0.6441	0.6441	0.7407	0.3330
CV-93	0.7309	0.4255	0.8895	0.6575	0.6667	0.7488	0.3618
CV-94	0.6945	0.3511	0.8729	0.6120	0.5893	0.7215	0.2638
CV-95	0.6873	0.3511	0.8619	0.6065	0.5690	0.7189	0.2476
CV-96	0.7164	0.4043	0.8785	0.6414	0.6333	0.7395	0.3247
CV-97	0.7091	0.3511	0.8950	0.6230	0.6346	0.7265	0.2981
CV-98	0.6909	0.3191	0.8840	0.6016	0.5882	0.7143	0.2479
CV-99	0.7127	0.3617	0.8950	0.6284	0.6415	0.7297	0.3087
CV-100	0.6945	0.3511	0.8729	0.6120	0.5893	0.7215	0.2638
CV-101	0.7018	0.3617	0.8785	0.6201	0.6071	0.7260	0.2829
CV-102	0.6982	0.3617	0.8729	0.6173	0.5965	0.7248	0.2746
CV-103	0.6873	0.3298	0.8729	0.6014	0.5741	0.7149	0.2420
CV-104	0.7091	0.3404	0.9006	0.6205	0.6400	0.7244	0.2963
CV-105	0.6691	0.3191	0.8508	0.5850	0.5263	0.7064	0.1989
CV-106	0.7055	0.3936	0.8674	0.6305	0.6066	0.7336	0.2980
CV-107	0.7055	0.3617	0.8840	0.6228	0.6182	0.7273	0.2913

CV-108	0.7091	0.3723	0.8840	0.6282	0.6250	0.7306	0.3019
CV-109	0.7127	0.3723	0.8895	0.6309	0.6364	0.7318	0.3105
CV-110	0.7091	0.4255	0.8564	0.6409	0.6061	0.7416	0.3131
CV-111	0.7127	0.3830	0.8840	0.6335	0.6316	0.7339	0.3124
CV-112	0.6727	0.2872	0.8729	0.5801	0.5400	0.7022	0.1970
CV-113	0.6945	0.3723	0.8619	0.6171	0.5833	0.7256	0.2690
CV-114	0.6836	0.3191	0.8729	0.5960	0.5660	0.7117	0.2310
CV-115	0.7127	0.3936	0.8785	0.6360	0.6271	0.7361	0.3144
CV-116	0.7091	0.3617	0.8895	0.6256	0.6296	0.7285	0.2999
CV-117	0.7127	0.3298	0.9116	0.6207	0.6596	0.7237	0.3042
CV-118	0.6873	0.3723	0.8508	0.6116	0.5645	0.7230	0.2533
CV-119	0.7055	0.3936	0.8674	0.6305	0.6066	0.7336	0.2980
CV-120	0.6945	0.3511	0.8729	0.6120	0.5893	0.7215	0.2638
CV-121	0.7164	0.4043	0.8785	0.6414	0.6333	0.7395	0.3247
CV-122	0.7164	0.3511	0.9061	0.6286	0.6600	0.7289	0.3162
CV-123	0.6691	0.2872	0.8674	0.5773	0.5294	0.7009	0.1887
CV-124	0.6800	0.2979	0.8785	0.5882	0.5600	0.7067	0.2168
CV-125	0.6691	0.2872	0.8674	0.5773	0.5294	0.7009	0.1887
CV-126	0.6836	0.3617	0.8508	0.6063	0.5574	0.7196	0.2426
CV-127	0.6873	0.3723	0.8508	0.6116	0.5645	0.7230	0.2533
CV-128	0.7382	0.4149	0.9061	0.6605	0.6964	0.7489	0.3781
CV-129	0.6909	0.3617	0.8619	0.6118	0.5763	0.7222	0.2583
CV-130	0.6582	0.2872	0.8508	0.5690	0.5000	0.6968	0.1648
CV-131	0.7018	0.3830	0.8674	0.6252	0.6000	0.7302	0.2875
CV-132	0.7055	0.3617	0.8840	0.6228	0.6182	0.7273	0.2913
CV-133	0.7055	0.3617	0.8840	0.6228	0.6182	0.7273	0.2913
CV-134	0.7273	0.3936	0.9006	0.6471	0.6727	0.7409	0.3488
CV-135	0.7236	0.3191	0.9337	0.6264	0.7143	0.7253	0.3334
CV-136	0.6873	0.3404	0.8674	0.6039	0.5714	0.7169	0.2448
CV-137	0.7236	0.3936	0.8950	0.6443	0.6607	0.7397	0.3400
CV-138	0.7127	0.4255	0.8619	0.6437	0.6154	0.7429	0.3209
CV-139	0.6800	0.2872	0.8840	0.5856	0.5625	0.7048	0.2139
CV-140	0.6982	0.3404	0.8840	0.6122	0.6038	0.7207	0.2698
CV-141	0.7164	0.3617	0.9006	0.6311	0.6538	0.7309	0.3177
CV-142	0.7164	0.4043	0.8785	0.6414	0.6333	0.7395	0.3247
CV-143	0.6982	0.3617	0.8729	0.6173	0.5965	0.7248	0.2746
CV-144	0.6873	0.3298	0.8729	0.6014	0.5741	0.7149	0.2420
CV-145	0.7055	0.3723	0.8785	0.6254	0.6140	0.7294	0.2935
CV-146	0.7127	0.3085	0.9227	0.6156	0.6744	0.7198	0.3019
CV-147	0.6873	0.3085	0.8840	0.5962	0.5800	0.7111	0.2367
CV-148	0.6945	0.3191	0.8895	0.6043	0.6000	0.7156	0.2566
CV-149	0.6727	0.3191	0.8564	0.5878	0.5357	0.7078	0.2067
CV-150	0.7236	0.3617	0.9116	0.6367	0.6800	0.7333	0.3361
CV-151	0.7309	0.3830	0.9116	0.6473	0.6923	0.7399	0.3568
CV-152	0.7127	0.3936	0.8785	0.6360	0.6271	0.7361	0.3144
CV-153	0.6945	0.3617	0.8674	0.6146	0.5862	0.7235	0.2664
CV-154	0.7200	0.3936	0.8895	0.6416	0.6491	0.7385	0.3313
CV-155	0.6800	0.3617	0.8453	0.6035	0.5484	0.7183	0.2350
CV-156	0.6764	0.3404	0.8508	0.5956	0.5424	0.7130	0.2210
CV-157	0.7164	0.3936	0.8840	0.6388	0.6379	0.7373	0.3228
CV-158	0.6836	0.3723	0.8453	0.6088	0.5556	0.7217	0.2456
CV-159	0.6764	0.3617	0.8398	0.6007	0.5397	0.7170	0.2274
CV-160	0.6945	0.3511	0.8729	0.6120	0.5893	0.7215	0.2638
CV-161	0.7309	0.3830	0.9116	0.6473	0.6923	0.7399	0.3568
CV-162	0.7236	0.3936	0.8950	0.6443	0.6607	0.7397	0.3400
CV-163	0.6727	0.3191	0.8564	0.5878	0.5357	0.7078	0.2067
CV-164	0.6909	0.3511	0.8674	0.6092	0.5789	0.7202	0.2556

CV-165	0.7018	0.3511	0.8840	0.6175	0.6111	0.7240	0.2806
CV-166	0.7164	0.3936	0.8840	0.6388	0.6379	0.7373	0.3228
CV-167	0.7055	0.3723	0.8785	0.6254	0.6140	0.7294	0.2935
CV-168	0.7345	0.4043	0.9061	0.6552	0.6909	0.7455	0.3680
CV-169	0.6691	0.3191	0.8508	0.5850	0.5263	0.7064	0.1989
CV-170	0.6945	0.3723	0.8619	0.6171	0.5833	0.7256	0.2690
CV-171	0.7091	0.4149	0.8619	0.6384	0.6094	0.7393	0.3107
CV-172	0.6909	0.3617	0.8619	0.6118	0.5763	0.7222	0.2583
CV-173	0.6764	0.2660	0.8895	0.5777	0.5556	0.7000	0.1993
CV-174	0.6982	0.3617	0.8729	0.6173	0.5965	0.7248	0.2746
CV-175	0.7055	0.3511	0.8895	0.6203	0.6226	0.7252	0.2893
CV-176	0.7200	0.4149	0.8785	0.6467	0.6393	0.7430	0.3349
CV-177	0.7018	0.3298	0.8950	0.6124	0.6200	0.7200	0.2765
CV-178	0.6800	0.3617	0.8453	0.6035	0.5484	0.7183	0.2350
CV-179	0.6945	0.3511	0.8729	0.6120	0.5893	0.7215	0.2638
CV-180	0.7055	0.3723	0.8785	0.6254	0.6140	0.7294	0.2935
CV-181	0.6836	0.3404	0.8619	0.6012	0.5614	0.7156	0.2367
CV-182	0.6982	0.3191	0.8950	0.6071	0.6122	0.7168	0.2655
CV-183	0.6836	0.3191	0.8729	0.5960	0.5660	0.7117	0.2310
CV-184	0.6982	0.3511	0.8785	0.6148	0.6000	0.7227	0.2722
CV-185	0.7055	0.3617	0.8840	0.6228	0.6182	0.7273	0.2913
CV-186	0.7018	0.3298	0.8950	0.6124	0.6200	0.7200	0.2765
CV-187	0.7164	0.3830	0.8895	0.6362	0.6429	0.7352	0.3209
CV-188	0.6945	0.3298	0.8840	0.6069	0.5962	0.7175	0.2589
CV-189	0.7055	0.3723	0.8785	0.6254	0.6140	0.7294	0.2935
CV-190	0.7055	0.3617	0.8840	0.6228	0.6182	0.7273	0.2913
CV-191	0.6982	0.3617	0.8729	0.6173	0.5965	0.7248	0.2746
CV-192	0.6618	0.2872	0.8564	0.5718	0.5094	0.6982	0.1727
CV-193	0.6909	0.3617	0.8619	0.6118	0.5763	0.7222	0.2583
CV-194	0.6945	0.3404	0.8785	0.6094	0.5926	0.7195	0.2613
CV-195	0.6945	0.3723	0.8619	0.6171	0.5833	0.7256	0.2690
CV-196	0.6764	0.3404	0.8508	0.5956	0.5424	0.7130	0.2210
CV-197	0.6836	0.3298	0.8674	0.5986	0.5636	0.7136	0.2338
CV-198	0.7164	0.3617	0.9006	0.6311	0.6538	0.7309	0.3177
CV-199	0.7055	0.3723	0.8785	0.6254	0.6140	0.7294	0.2935
CV-200	0.6764	0.3298	0.8564	0.5931	0.5439	0.7110	0.2178
CV-201	0.6982	0.3404	0.8840	0.6122	0.6038	0.7207	0.2698
CV-202	0.7055	0.3617	0.8840	0.6228	0.6182	0.7273	0.2913
CV-203	0.6836	0.3511	0.8564	0.6037	0.5593	0.7176	0.2397
CV-204	0.7091	0.3723	0.8840	0.6282	0.6250	0.7306	0.3019
CV-205	0.7164	0.3723	0.8950	0.6337	0.6481	0.7330	0.3192
CV-206	0.6982	0.3511	0.8785	0.6148	0.6000	0.7227	0.2722
CV-207	0.7018	0.3617	0.8785	0.6201	0.6071	0.7260	0.2829
CV-208	0.6982	0.3511	0.8785	0.6148	0.6000	0.7227	0.2722
CV-209	0.7055	0.3830	0.8729	0.6280	0.6102	0.7315	0.2957
CV-210	0.6836	0.3298	0.8674	0.5986	0.5636	0.7136	0.2338
CV-211	0.6800	0.3298	0.8619	0.5958	0.5536	0.7123	0.2258
CV-212	0.7164	0.3830	0.8895	0.6362	0.6429	0.7352	0.3209
CV-213	0.6982	0.3617	0.8729	0.6173	0.5965	0.7248	0.2746
CV-214	0.6873	0.3511	0.8619	0.6065	0.5690	0.7189	0.2476
CV-215	0.6836	0.3404	0.8619	0.6012	0.5614	0.7156	0.2367
CV-216	0.6800	0.3298	0.8619	0.5958	0.5536	0.7123	0.2258
CV-217	0.7018	0.3723	0.8729	0.6226	0.6034	0.7281	0.2852
CV-218	0.6764	0.3298	0.8564	0.5931	0.5439	0.7110	0.2178
CV-219	0.6836	0.3404	0.8619	0.6012	0.5614	0.7156	0.2367
CV-220	0.6836	0.3191	0.8729	0.5960	0.5660	0.7117	0.2310
CV-221	0.7345	0.4149	0.9006	0.6577	0.6842	0.7477	0.3691

CV-222	0.6909	0.3298	0.8785	0.6041	0.5849	0.7162	0.2504
CV-223	0.7055	0.3298	0.9006	0.6152	0.6327	0.7212	0.2855
CV-224	0.7055	0.3617	0.8840	0.6228	0.6182	0.7273	0.2913
CV-225	0.6764	0.3404	0.8508	0.5956	0.5424	0.7130	0.2210
CV-226	0.7055	0.3936	0.8674	0.6305	0.6066	0.7336	0.2980
CV-227	0.6945	0.3617	0.8674	0.6146	0.5862	0.7235	0.2664
CV-228	0.6873	0.3404	0.8674	0.6039	0.5714	0.7169	0.2448
CV-229	0.6873	0.3191	0.8785	0.5988	0.5769	0.7130	0.2394
CV-230	0.7164	0.3617	0.9006	0.6311	0.6538	0.7309	0.3177
CV-231	0.6982	0.3404	0.8840	0.6122	0.6038	0.7207	0.2698
CV-232	0.6800	0.3404	0.8564	0.5984	0.5517	0.7143	0.2288
CV-233	0.7127	0.3723	0.8895	0.6309	0.6364	0.7318	0.3105
CV-234	0.7273	0.4255	0.8840	0.6548	0.6557	0.7477	0.3534
CV-235	0.7055	0.3723	0.8785	0.6254	0.6140	0.7294	0.2935
CV-236	0.6945	0.3617	0.8674	0.6146	0.5862	0.7235	0.2664
CV-237	0.7236	0.4043	0.8895	0.6469	0.6552	0.7419	0.3415
CV-238	0.6909	0.3191	0.8840	0.6016	0.5882	0.7143	0.2479
CV-239	0.6945	0.3723	0.8619	0.6171	0.5833	0.7256	0.2690
CV-240	0.6982	0.3830	0.8619	0.6224	0.5902	0.7290	0.2795
CV-241	0.7091	0.3723	0.8840	0.6282	0.6250	0.7306	0.3019
CV-242	0.6982	0.3723	0.8674	0.6199	0.5932	0.7269	0.2770
CV-243	0.7055	0.3936	0.8674	0.6305	0.6066	0.7336	0.2980
CV-244	0.7055	0.3830	0.8729	0.6280	0.6102	0.7315	0.2957
CV-245	0.7127	0.3723	0.8895	0.6309	0.6364	0.7318	0.3105
CV-246	0.7091	0.3617	0.8895	0.6256	0.6296	0.7285	0.2999
CV-247	0.7055	0.3617	0.8840	0.6228	0.6182	0.7273	0.2913
CV-248	0.7018	0.3617	0.8785	0.6201	0.6071	0.7260	0.2829
CV-249	0.7200	0.3617	0.9061	0.6339	0.6667	0.7321	0.3268
CV-250	0.7127	0.3511	0.9006	0.6258	0.6471	0.7277	0.3071
CV-251	0.7200	0.3830	0.8950	0.6390	0.6545	0.7364	0.3297
CV-252	0.6655	0.2979	0.8564	0.5771	0.5185	0.7014	0.1841
CV-253	0.7127	0.3511	0.9006	0.6258	0.6471	0.7277	0.3071
CV-254	0.7309	0.3936	0.9061	0.6498	0.6852	0.7421	0.3578
CV-255	0.6873	0.3404	0.8674	0.6039	0.5714	0.7169	0.2448
CV-256	0.7055	0.3830	0.8729	0.6280	0.6102	0.7315	0.2957
CV-257	0.7127	0.3617	0.8950	0.6284	0.6415	0.7297	0.3087
CV-258	0.7127	0.3830	0.8840	0.6335	0.6316	0.7339	0.3124
CV-259	0.6764	0.3298	0.8564	0.5931	0.5439	0.7110	0.2178
CV-260	0.6873	0.3404	0.8674	0.6039	0.5714	0.7169	0.2448
CV-261	0.6945	0.3298	0.8840	0.6069	0.5962	0.7175	0.2589
CV-262	0.6836	0.3191	0.8729	0.5960	0.5660	0.7117	0.2310
CV-263	0.7018	0.3617	0.8785	0.6201	0.6071	0.7260	0.2829
CV-264	0.7164	0.3723	0.8950	0.6337	0.6481	0.7330	0.3192
CV-265	0.7091	0.3723	0.8840	0.6282	0.6250	0.7306	0.3019
CV-266	0.6836	0.3723	0.8453	0.6088	0.5556	0.7217	0.2456
CV-267	0.6800	0.3511	0.8508	0.6009	0.5500	0.7163	0.2319
CV-268	0.7127	0.4043	0.8729	0.6386	0.6230	0.7383	0.3164
CV-269	0.7200	0.3830	0.8950	0.6390	0.6545	0.7364	0.3297
CV-270	0.6982	0.3404	0.8840	0.6122	0.6038	0.7207	0.2698
CV-271	0.7164	0.3936	0.8840	0.6388	0.6379	0.7373	0.3228
CV-272	0.6945	0.3617	0.8674	0.6146	0.5862	0.7235	0.2664
CV-273	0.7200	0.4043	0.8840	0.6441	0.6441	0.7407	0.3330
CV-274	0.7164	0.4149	0.8729	0.6439	0.6290	0.7418	0.3267
CV-275	0.7418	0.4362	0.9006	0.6684	0.6949	0.7546	0.3891
CV-276	0.7309	0.3723	0.9171	0.6447	0.7000	0.7378	0.3560
CV-277	0.7127	0.3830	0.8840	0.6335	0.6316	0.7339	0.3124
CV-278	0.7127	0.3936	0.8785	0.6360	0.6271	0.7361	0.3144

CV-279	0.7055	0.3617	0.8840	0.6228	0.6182	0.7273	0.2913
CV-280	0.7091	0.3617	0.8895	0.6256	0.6296	0.7285	0.2999
CV-281	0.7164	0.3298	0.9171	0.6235	0.6739	0.7249	0.3138
CV-282	0.7127	0.4043	0.8729	0.6386	0.6230	0.7383	0.3164
CV-283	0.6764	0.3298	0.8564	0.5931	0.5439	0.7110	0.2178
CV-284	0.7018	0.3404	0.8895	0.6150	0.6154	0.7220	0.2785
CV-285	0.7055	0.3511	0.8895	0.6203	0.6226	0.7252	0.2893
CV-286	0.7345	0.4362	0.8895	0.6628	0.6721	0.7523	0.3718
CV-287	0.6873	0.3617	0.8564	0.6090	0.5667	0.7209	0.2504
CV-288	0.7273	0.3830	0.9061	0.6445	0.6792	0.7387	0.3476
CV-289	0.6873	0.3191	0.8785	0.5988	0.5769	0.7130	0.2394
CV-290	0.7127	0.3936	0.8785	0.6360	0.6271	0.7361	0.3144
CV-291	0.6982	0.3723	0.8674	0.6199	0.5932	0.7269	0.2770
CV-292	0.7236	0.3936	0.8950	0.6443	0.6607	0.7397	0.3400
CV-293	0.7127	0.4043	0.8729	0.6386	0.6230	0.7383	0.3164
CV-294	0.6982	0.3404	0.8840	0.6122	0.6038	0.7207	0.2698
CV-295	0.7236	0.3830	0.9006	0.6418	0.6667	0.7376	0.3385
CV-296	0.6873	0.3085	0.8840	0.5962	0.5800	0.7111	0.2367
CV-297	0.7091	0.3723	0.8840	0.6282	0.6250	0.7306	0.3019
CV-298	0.6800	0.3085	0.8729	0.5907	0.5577	0.7085	0.2198
CV-299	0.7200	0.3617	0.9061	0.6339	0.6667	0.7321	0.3268
CV-300	0.6909	0.3511	0.8674	0.6092	0.5789	0.7202	0.2556
CV-301	0.7055	0.3723	0.8785	0.6254	0.6140	0.7294	0.2935
CV-302	0.6836	0.3298	0.8674	0.5986	0.5636	0.7136	0.2338
CV-303	0.6982	0.3511	0.8785	0.6148	0.6000	0.7227	0.2722
CV-304	0.6800	0.3617	0.8453	0.6035	0.5484	0.7183	0.2350
CV-305	0.7055	0.3617	0.8840	0.6228	0.6182	0.7273	0.2913
CV-306	0.7055	0.3617	0.8840	0.6228	0.6182	0.7273	0.2913
CV-307	0.7091	0.3404	0.9006	0.6205	0.6400	0.7244	0.2963
CV-308	0.7018	0.3298	0.8950	0.6124	0.6200	0.7200	0.2765
CV-309	0.7055	0.3723	0.8785	0.6254	0.6140	0.7294	0.2935
CV-310	0.6873	0.3617	0.8564	0.6090	0.5667	0.7209	0.2504
CV-311	0.6982	0.3723	0.8674	0.6199	0.5932	0.7269	0.2770
CV-312	0.7164	0.3511	0.9061	0.6286	0.6600	0.7289	0.3162
CV-313	0.7055	0.3830	0.8729	0.6280	0.6102	0.7315	0.2957
CV-314	0.7055	0.3723	0.8785	0.6254	0.6140	0.7294	0.2935
CV-315	0.7127	0.4043	0.8729	0.6386	0.6230	0.7383	0.3164
CV-316	0.6982	0.3298	0.8895	0.6096	0.6078	0.7188	0.2676
CV-317	0.6982	0.3830	0.8619	0.6224	0.5902	0.7290	0.2795
CV-318	0.6909	0.3298	0.8785	0.6041	0.5849	0.7162	0.2504
CV-319	0.7127	0.3511	0.9006	0.6258	0.6471	0.7277	0.3071
CV-320	0.6836	0.3191	0.8729	0.5960	0.5660	0.7117	0.2310
CV-321	0.6836	0.3723	0.8453	0.6088	0.5556	0.7217	0.2456
CV-322	0.6800	0.3617	0.8453	0.6035	0.5484	0.7183	0.2350
CV-323	0.6836	0.3191	0.8729	0.5960	0.5660	0.7117	0.2310
CV-324	0.6800	0.3191	0.8674	0.5933	0.5556	0.7104	0.2227
CV-325	0.7345	0.3830	0.9171	0.6501	0.7059	0.7411	0.3662
CV-326	0.7091	0.3830	0.8785	0.6307	0.6207	0.7327	0.3040
CV-327	0.6982	0.3511	0.8785	0.6148	0.6000	0.7227	0.2722
CV-328	0.7091	0.3723	0.8840	0.6282	0.6250	0.7306	0.3019
CV-329	0.7164	0.3830	0.8895	0.6362	0.6429	0.7352	0.3209
CV-330	0.7164	0.3617	0.9006	0.6311	0.6538	0.7309	0.3177
CV-331	0.7055	0.3511	0.8895	0.6203	0.6226	0.7252	0.2893
CV-332	0.7127	0.3936	0.8785	0.6360	0.6271	0.7361	0.3144
CV-333	0.7200	0.3617	0.9061	0.6339	0.6667	0.7321	0.3268
CV-334	0.7200	0.3723	0.9006	0.6364	0.6604	0.7342	0.3282
CV-335	0.6873	0.3404	0.8674	0.6039	0.5714	0.7169	0.2448

CV-336	0.6691	0.2979	0.8619	0.5799	0.5283	0.7027	0.1921
CV-337	0.7164	0.3617	0.9006	0.6311	0.6538	0.7309	0.3177
CV-338	0.6945	0.3936	0.8508	0.6222	0.5781	0.7299	0.2744
CV-339	0.7091	0.3830	0.8785	0.6307	0.6207	0.7327	0.3040
CV-340	0.6982	0.3723	0.8674	0.6199	0.5932	0.7269	0.2770
CV-341	0.7018	0.3723	0.8729	0.6226	0.6034	0.7281	0.2852
CV-342	0.7091	0.3511	0.8950	0.6230	0.6346	0.7265	0.2981
CV-343	0.7164	0.3936	0.8840	0.6388	0.6379	0.7373	0.3228
CV-344	0.7055	0.3723	0.8785	0.6254	0.6140	0.7294	0.2935
CV-345	0.7018	0.4043	0.8564	0.6303	0.5938	0.7346	0.2925
CV-346	0.6909	0.3511	0.8674	0.6092	0.5789	0.7202	0.2556
CV-347	0.7127	0.3511	0.9006	0.6258	0.6471	0.7277	0.3071
CV-348	0.7200	0.4043	0.8840	0.6441	0.6441	0.7407	0.3330
CV-349	0.6873	0.3404	0.8674	0.6039	0.5714	0.7169	0.2448
CV-350	0.7018	0.3511	0.8840	0.6175	0.6111	0.7240	0.2806
CV-351	0.6982	0.3617	0.8729	0.6173	0.5965	0.7248	0.2746
CV-352	0.6945	0.3191	0.8895	0.6043	0.6000	0.7156	0.2566
CV-353	0.7055	0.3511	0.8895	0.6203	0.6226	0.7252	0.2893
CV-354	0.6800	0.3298	0.8619	0.5958	0.5536	0.7123	0.2258
CV-355	0.7055	0.3723	0.8785	0.6254	0.6140	0.7294	0.2935
CV-356	0.6945	0.3404	0.8785	0.6094	0.5926	0.7195	0.2613
CV-357	0.7164	0.3617	0.9006	0.6311	0.6538	0.7309	0.3177
CV-358	0.6764	0.3617	0.8398	0.6007	0.5397	0.7170	0.2274
CV-359	0.7382	0.3936	0.9171	0.6554	0.7115	0.7444	0.3764
CV-360	0.7055	0.3511	0.8895	0.6203	0.6226	0.7252	0.2893
CV-361	0.6873	0.3191	0.8785	0.5988	0.5769	0.7130	0.2394
CV-362	0.7127	0.3617	0.8950	0.6284	0.6415	0.7297	0.3087
CV-363	0.6873	0.3511	0.8619	0.6065	0.5690	0.7189	0.2476
CV-364	0.7127	0.3511	0.9006	0.6258	0.6471	0.7277	0.3071
CV-365	0.6800	0.3298	0.8619	0.5958	0.5536	0.7123	0.2258
CV-366	0.6873	0.3617	0.8564	0.6090	0.5667	0.7209	0.2504
CV-367	0.7018	0.3191	0.9006	0.6099	0.6250	0.7181	0.2745
CV-368	0.7091	0.3830	0.8785	0.6307	0.6207	0.7327	0.3040
CV-369	0.7055	0.3511	0.8895	0.6203	0.6226	0.7252	0.2893
CV-370	0.7091	0.3723	0.8840	0.6282	0.6250	0.7306	0.3019
CV-371	0.7055	0.3404	0.8950	0.6177	0.6275	0.7232	0.2873
CV-372	0.6945	0.3511	0.8729	0.6120	0.5893	0.7215	0.2638
CV-373	0.7127	0.3298	0.9116	0.6207	0.6596	0.7237	0.3042
CV-374	0.7273	0.3936	0.9006	0.6471	0.6727	0.7409	0.3488
CV-375	0.6836	0.3298	0.8674	0.5986	0.5636	0.7136	0.2338
CV-376	0.7236	0.4149	0.8840	0.6494	0.6500	0.7442	0.3432
CV-377	0.6691	0.3085	0.8564	0.5824	0.5273	0.7045	0.1955
CV-378	0.7055	0.3298	0.9006	0.6152	0.6327	0.7212	0.2855
CV-379	0.7091	0.3617	0.8895	0.6256	0.6296	0.7285	0.2999
CV-380	0.7018	0.3723	0.8729	0.6226	0.6034	0.7281	0.2852
CV-381	0.6945	0.3617	0.8674	0.6146	0.5862	0.7235	0.2664
CV-382	0.7200	0.3723	0.9006	0.6364	0.6604	0.7342	0.3282
CV-383	0.7091	0.3617	0.8895	0.6256	0.6296	0.7285	0.2999
CV-384	0.6873	0.3191	0.8785	0.5988	0.5769	0.7130	0.2394
CV-385	0.6764	0.2979	0.8729	0.5854	0.5490	0.7054	0.2084
CV-386	0.7164	0.4043	0.8785	0.6414	0.6333	0.7395	0.3247
CV-387	0.6836	0.3404	0.8619	0.6012	0.5614	0.7156	0.2367
CV-388	0.6945	0.3404	0.8785	0.6094	0.5926	0.7195	0.2613
CV-389	0.7164	0.3830	0.8895	0.6362	0.6429	0.7352	0.3209
CV-390	0.7018	0.3617	0.8785	0.6201	0.6071	0.7260	0.2829
CV-391	0.6909	0.3723	0.8564	0.6143	0.5738	0.7243	0.2611
CV-392	0.6909	0.2766	0.9061	0.5913	0.6047	0.7069	0.2386

CV-393	0.7055	0.3298	0.9006	0.6152	0.6327	0.7212	0.2855
CV-394	0.6836	0.3511	0.8564	0.6037	0.5593	0.7176	0.2397
CV-395	0.6945	0.3723	0.8619	0.6171	0.5833	0.7256	0.2690
CV-396	0.7091	0.3723	0.8840	0.6282	0.6250	0.7306	0.3019
CV-397	0.6873	0.3723	0.8508	0.6116	0.5645	0.7230	0.2533
CV-398	0.7200	0.3617	0.9061	0.6339	0.6667	0.7321	0.3268
CV-399	0.7091	0.4043	0.8674	0.6358	0.6129	0.7371	0.3083
CV-400	0.6909	0.3298	0.8785	0.6041	0.5849	0.7162	0.2504
CV-401	0.6691	0.3085	0.8564	0.5824	0.5273	0.7045	0.1955
CV-402	0.6909	0.3723	0.8564	0.6143	0.5738	0.7243	0.2611
CV-403	0.6545	0.2766	0.8508	0.5637	0.4906	0.6937	0.1532
CV-404	0.7164	0.3830	0.8895	0.6362	0.6429	0.7352	0.3209
CV-405	0.7091	0.3936	0.8729	0.6333	0.6167	0.7349	0.3061
CV-406	0.6727	0.3191	0.8564	0.5878	0.5357	0.7078	0.2067
CV-407	0.6873	0.3511	0.8619	0.6065	0.5690	0.7189	0.2476
CV-408	0.7091	0.3723	0.8840	0.6282	0.6250	0.7306	0.3019
CV-409	0.7127	0.3617	0.8950	0.6284	0.6415	0.7297	0.3087
CV-410	0.7055	0.3723	0.8785	0.6254	0.6140	0.7294	0.2935
CV-411	0.6836	0.3617	0.8508	0.6063	0.5574	0.7196	0.2426
CV-412	0.7127	0.3723	0.8895	0.6309	0.6364	0.7318	0.3105
CV-413	0.7127	0.3936	0.8785	0.6360	0.6271	0.7361	0.3144
CV-414	0.7018	0.3617	0.8785	0.6201	0.6071	0.7260	0.2829
CV-415	0.6909	0.3511	0.8674	0.6092	0.5789	0.7202	0.2556
CV-416	0.6982	0.3404	0.8840	0.6122	0.6038	0.7207	0.2698
CV-417	0.6764	0.3298	0.8564	0.5931	0.5439	0.7110	0.2178
CV-418	0.6836	0.3298	0.8674	0.5986	0.5636	0.7136	0.2338
CV-419	0.6982	0.3830	0.8619	0.6224	0.5902	0.7290	0.2795
CV-420	0.6909	0.3511	0.8674	0.6092	0.5789	0.7202	0.2556
CV-421	0.7055	0.3617	0.8840	0.6228	0.6182	0.7273	0.2913
CV-422	0.6873	0.3617	0.8564	0.6090	0.5667	0.7209	0.2504
CV-423	0.7236	0.4149	0.8840	0.6494	0.6500	0.7442	0.3432
CV-424	0.7164	0.3723	0.8950	0.6337	0.6481	0.7330	0.3192
CV-425	0.7018	0.3617	0.8785	0.6201	0.6071	0.7260	0.2829
CV-426	0.7382	0.4043	0.9116	0.6579	0.7037	0.7466	0.3771
CV-427	0.6909	0.3511	0.8674	0.6092	0.5789	0.7202	0.2556
CV-428	0.7018	0.4043	0.8564	0.6303	0.5938	0.7346	0.2925
CV-429	0.6982	0.3404	0.8840	0.6122	0.6038	0.7207	0.2698
CV-430	0.6836	0.3404	0.8619	0.6012	0.5614	0.7156	0.2367
CV-431	0.7200	0.3830	0.8950	0.6390	0.6545	0.7364	0.3297
CV-432	0.6727	0.3298	0.8508	0.5903	0.5345	0.7097	0.2100
CV-433	0.6873	0.2979	0.8895	0.5937	0.5833	0.7093	0.2341
CV-434	0.7164	0.3723	0.8950	0.6337	0.6481	0.7330	0.3192
CV-435	0.7055	0.3723	0.8785	0.6254	0.6140	0.7294	0.2935
CV-436	0.7236	0.3936	0.8950	0.6443	0.6607	0.7397	0.3400
CV-437	0.7018	0.3723	0.8729	0.6226	0.6034	0.7281	0.2852
CV-438	0.7200	0.4362	0.8674	0.6518	0.6308	0.7476	0.3389
CV-439	0.7055	0.3511	0.8895	0.6203	0.6226	0.7252	0.2893
CV-440	0.7236	0.3936	0.8950	0.6443	0.6607	0.7397	0.3400
CV-441	0.6727	0.3404	0.8453	0.5929	0.5333	0.7116	0.2133
CV-442	0.7018	0.3617	0.8785	0.6201	0.6071	0.7260	0.2829
CV-443	0.6982	0.4043	0.8508	0.6275	0.5846	0.7333	0.2848
CV-444	0.6873	0.3298	0.8729	0.6014	0.5741	0.7149	0.2420
CV-445	0.7164	0.3830	0.8895	0.6362	0.6429	0.7352	0.3209
CV-446	0.6764	0.3404	0.8508	0.5956	0.5424	0.7130	0.2210
CV-447	0.7273	0.4043	0.8950	0.6496	0.6667	0.7431	0.3502
CV-448	0.7127	0.3936	0.8785	0.6360	0.6271	0.7361	0.3144
CV-449	0.7164	0.3830	0.8895	0.6362	0.6429	0.7352	0.3209

CV-450	0.7018	0.3298	0.8950	0.6124	0.6200	0.7200	0.2765
CV-451	0.7164	0.3936	0.8840	0.6388	0.6379	0.7373	0.3228
CV-452	0.7164	0.3830	0.8895	0.6362	0.6429	0.7352	0.3209
CV-453	0.7127	0.3511	0.9006	0.6258	0.6471	0.7277	0.3071
CV-454	0.6909	0.3617	0.8619	0.6118	0.5763	0.7222	0.2583
CV-455	0.7091	0.4149	0.8619	0.6384	0.6094	0.7393	0.3107
CV-456	0.6836	0.3404	0.8619	0.6012	0.5614	0.7156	0.2367
CV-457	0.7345	0.4043	0.9061	0.6552	0.6909	0.7455	0.3680
CV-458	0.6727	0.2979	0.8674	0.5826	0.5385	0.7040	0.2002
CV-459	0.7200	0.3830	0.8950	0.6390	0.6545	0.7364	0.3297
CV-460	0.6873	0.3511	0.8619	0.6065	0.5690	0.7189	0.2476
CV-461	0.7127	0.3936	0.8785	0.6360	0.6271	0.7361	0.3144
CV-462	0.6655	0.2979	0.8564	0.5771	0.5185	0.7014	0.1841
CV-463	0.7018	0.3830	0.8674	0.6252	0.6000	0.7302	0.2875
CV-464	0.7273	0.3936	0.9006	0.6471	0.6727	0.7409	0.3488
CV-465	0.6982	0.3617	0.8729	0.6173	0.5965	0.7248	0.2746
CV-466	0.6836	0.3617	0.8508	0.6063	0.5574	0.7196	0.2426
CV-467	0.6691	0.3085	0.8564	0.5824	0.5273	0.7045	0.1955
CV-468	0.6800	0.3617	0.8453	0.6035	0.5484	0.7183	0.2350
CV-469	0.6873	0.3830	0.8453	0.6141	0.5625	0.7251	0.2562
CV-470	0.6836	0.3191	0.8729	0.5960	0.5660	0.7117	0.2310
CV-471	0.6982	0.3830	0.8619	0.6224	0.5902	0.7290	0.2795
CV-472	0.7236	0.3723	0.9061	0.6392	0.6731	0.7354	0.3372
CV-473	0.6909	0.3404	0.8729	0.6067	0.5818	0.7182	0.2530
CV-474	0.6727	0.3617	0.8343	0.5980	0.5313	0.7156	0.2200
CV-475	0.7055	0.3723	0.8785	0.6254	0.6140	0.7294	0.2935
CV-476	0.7164	0.3617	0.9006	0.6311	0.6538	0.7309	0.3177
CV-477	0.6800	0.3298	0.8619	0.5958	0.5536	0.7123	0.2258
CV-478	0.6982	0.3617	0.8729	0.6173	0.5965	0.7248	0.2746
CV-479	0.6982	0.3404	0.8840	0.6122	0.6038	0.7207	0.2698
CV-480	0.7055	0.3830	0.8729	0.6280	0.6102	0.7315	0.2957
CV-481	0.7018	0.3830	0.8674	0.6252	0.6000	0.7302	0.2875
CV-482	0.6982	0.3404	0.8840	0.6122	0.6038	0.7207	0.2698
CV-483	0.6982	0.3723	0.8674	0.6199	0.5932	0.7269	0.2770
CV-484	0.7018	0.3617	0.8785	0.6201	0.6071	0.7260	0.2829
CV-485	0.6909	0.3511	0.8674	0.6092	0.5789	0.7202	0.2556
CV-486	0.7018	0.3617	0.8785	0.6201	0.6071	0.7260	0.2829
CV-487	0.7055	0.3830	0.8729	0.6280	0.6102	0.7315	0.2957
CV-488	0.7055	0.3511	0.8895	0.6203	0.6226	0.7252	0.2893
CV-489	0.7309	0.4255	0.8895	0.6575	0.6667	0.7488	0.3618
CV-490	0.7091	0.3830	0.8785	0.6307	0.6207	0.7327	0.3040
CV-491	0.6982	0.3511	0.8785	0.6148	0.6000	0.7227	0.2722
CV-492	0.7018	0.3723	0.8729	0.6226	0.6034	0.7281	0.2852
CV-493	0.6800	0.3511	0.8508	0.6009	0.5500	0.7163	0.2319
CV-494	0.6945	0.3830	0.8564	0.6197	0.5806	0.7277	0.2717
CV-495	0.7273	0.3936	0.9006	0.6471	0.6727	0.7409	0.3488
CV-496	0.6873	0.3511	0.8619	0.6065	0.5690	0.7189	0.2476
CV-497	0.7055	0.3511	0.8895	0.6203	0.6226	0.7252	0.2893
CV-498	0.7018	0.3404	0.8895	0.6150	0.6154	0.7220	0.2785
CV-499	0.7018	0.3404	0.8895	0.6150	0.6154	0.7220	0.2785
CV-500	0.7127	0.3723	0.8895	0.6309	0.6364	0.7318	0.3105

Table S7. Performance of 5-fold cross-validation from k-nearest neighbors (kNN). Performance of 5-fold cross-validations were evaluated by seven performance metrics. Each row shows the performance of one time 5-fold cross-validation. 5-fold cross-validation was repeated 500 times (CV-1 ~ CV-500).

Cross-validation	Accuracy	Sensitivity	Specificity	Balanced accuracy	Positive prediction rate	Negative prediction rate	Matthews correlation coefficient
CV-1	0.6836	0.2979	0.8840	0.5909	0.5714	0.7080	0.2254
CV-2	0.6800	0.3191	0.8674	0.5933	0.5556	0.7104	0.2227
CV-3	0.6982	0.2979	0.9061	0.6020	0.6222	0.7130	0.2615
CV-4	0.6764	0.3191	0.8619	0.5905	0.5455	0.7091	0.2147
CV-5	0.6873	0.3085	0.8840	0.5962	0.5800	0.7111	0.2367
CV-6	0.6982	0.3191	0.8950	0.6071	0.6122	0.7168	0.2655
CV-7	0.6836	0.2872	0.8895	0.5884	0.5745	0.7061	0.2227
CV-8	0.6836	0.3085	0.8785	0.5935	0.5686	0.7098	0.2282
CV-9	0.7164	0.3617	0.9006	0.6311	0.6538	0.7309	0.3177
CV-10	0.6836	0.2979	0.8840	0.5909	0.5714	0.7080	0.2254
CV-11	0.6909	0.3085	0.8895	0.5990	0.5918	0.7124	0.2454
CV-12	0.6764	0.2979	0.8729	0.5854	0.5490	0.7054	0.2084
CV-13	0.7055	0.3298	0.9006	0.6152	0.6327	0.7212	0.2855
CV-14	0.6873	0.3191	0.8785	0.5988	0.5769	0.7130	0.2394
CV-15	0.7091	0.3191	0.9116	0.6154	0.6522	0.7205	0.2933
CV-16	0.6945	0.3191	0.8895	0.6043	0.6000	0.7156	0.2566
CV-17	0.6982	0.3085	0.9006	0.6045	0.6170	0.7149	0.2634
CV-18	0.6909	0.2979	0.8950	0.5964	0.5957	0.7105	0.2431
CV-19	0.7127	0.3723	0.8895	0.6309	0.6364	0.7318	0.3105
CV-20	0.6909	0.3298	0.8785	0.6041	0.5849	0.7162	0.2504
CV-21	0.6836	0.3191	0.8729	0.5960	0.5660	0.7117	0.2310
CV-22	0.6836	0.2979	0.8840	0.5909	0.5714	0.7080	0.2254
CV-23	0.7127	0.3511	0.9006	0.6258	0.6471	0.7277	0.3071
CV-24	0.6982	0.3617	0.8729	0.6173	0.5965	0.7248	0.2746
CV-25	0.6727	0.2872	0.8729	0.5801	0.5400	0.7022	0.1970
CV-26	0.7091	0.3617	0.8895	0.6256	0.6296	0.7285	0.2999
CV-27	0.6873	0.3298	0.8729	0.6014	0.5741	0.7149	0.2420
CV-28	0.7200	0.3404	0.9171	0.6288	0.6809	0.7281	0.3245
CV-29	0.6909	0.3404	0.8729	0.6067	0.5818	0.7182	0.2530
CV-30	0.6873	0.3298	0.8729	0.6014	0.5741	0.7149	0.2420
CV-31	0.6909	0.3298	0.8785	0.6041	0.5849	0.7162	0.2504
CV-32	0.6909	0.3191	0.8840	0.6016	0.5882	0.7143	0.2479
CV-33	0.6982	0.3085	0.9006	0.6045	0.6170	0.7149	0.2634
CV-34	0.7091	0.3298	0.9061	0.6179	0.6458	0.7225	0.2947
CV-35	0.7018	0.3404	0.8895	0.6150	0.6154	0.7220	0.2785
CV-36	0.6764	0.2979	0.8729	0.5854	0.5490	0.7054	0.2084
CV-37	0.6691	0.2979	0.8619	0.5799	0.5283	0.7027	0.1921
CV-38	0.6836	0.2872	0.8895	0.5884	0.5745	0.7061	0.2227
CV-39	0.7018	0.3298	0.8950	0.6124	0.6200	0.7200	0.2765
CV-40	0.7091	0.3511	0.8950	0.6230	0.6346	0.7265	0.2981
CV-41	0.6982	0.3085	0.9006	0.6045	0.6170	0.7149	0.2634
CV-42	0.6982	0.3085	0.9006	0.6045	0.6170	0.7149	0.2634
CV-43	0.6800	0.3085	0.8729	0.5907	0.5577	0.7085	0.2198
CV-44	0.7018	0.3511	0.8840	0.6175	0.6111	0.7240	0.2806
CV-45	0.7018	0.3191	0.9006	0.6099	0.6250	0.7181	0.2745
CV-46	0.7127	0.3404	0.9061	0.6233	0.6531	0.7257	0.3055
CV-47	0.6764	0.2872	0.8785	0.5828	0.5510	0.7035	0.2054
CV-48	0.7018	0.3298	0.8950	0.6124	0.6200	0.7200	0.2765
CV-49	0.6873	0.3298	0.8729	0.6014	0.5741	0.7149	0.2420
CV-50	0.6982	0.3191	0.8950	0.6071	0.6122	0.7168	0.2655

CV-51	0.7018	0.2872	0.9171	0.6022	0.6429	0.7124	0.2695
CV-52	0.6945	0.2979	0.9006	0.5992	0.6087	0.7118	0.2522
CV-53	0.6691	0.2979	0.8619	0.5799	0.5283	0.7027	0.1921
CV-54	0.7127	0.3511	0.9006	0.6258	0.6471	0.7277	0.3071
CV-55	0.7018	0.3511	0.8840	0.6175	0.6111	0.7240	0.2806
CV-56	0.7055	0.3617	0.8840	0.6228	0.6182	0.7273	0.2913
CV-57	0.7091	0.3298	0.9061	0.6179	0.6458	0.7225	0.2947
CV-58	0.6836	0.3298	0.8674	0.5986	0.5636	0.7136	0.2338
CV-59	0.7055	0.3511	0.8895	0.6203	0.6226	0.7252	0.2893
CV-60	0.6764	0.3191	0.8619	0.5905	0.5455	0.7091	0.2147
CV-61	0.6873	0.2872	0.8950	0.5911	0.5870	0.7074	0.2316
CV-62	0.6945	0.3085	0.8950	0.6018	0.6042	0.7137	0.2543
CV-63	0.7127	0.3191	0.9171	0.6181	0.6667	0.7217	0.3029
CV-64	0.6836	0.3191	0.8729	0.5960	0.5660	0.7117	0.2310
CV-65	0.7018	0.3511	0.8840	0.6175	0.6111	0.7240	0.2806
CV-66	0.6909	0.2979	0.8950	0.5964	0.5957	0.7105	0.2431
CV-67	0.6691	0.2979	0.8619	0.5799	0.5283	0.7027	0.1921
CV-68	0.6873	0.3085	0.8840	0.5962	0.5800	0.7111	0.2367
CV-69	0.7055	0.3723	0.8785	0.6254	0.6140	0.7294	0.2935
CV-70	0.6945	0.3085	0.8950	0.6018	0.6042	0.7137	0.2543
CV-71	0.6909	0.2979	0.8950	0.5964	0.5957	0.7105	0.2431
CV-72	0.6800	0.3298	0.8619	0.5958	0.5536	0.7123	0.2258
CV-73	0.7127	0.3617	0.8950	0.6284	0.6415	0.7297	0.3087
CV-74	0.6873	0.3191	0.8785	0.5988	0.5769	0.7130	0.2394
CV-75	0.6873	0.3298	0.8729	0.6014	0.5741	0.7149	0.2420
CV-76	0.6836	0.2872	0.8895	0.5884	0.5745	0.7061	0.2227
CV-77	0.6764	0.2766	0.8840	0.5803	0.5532	0.7018	0.2023
CV-78	0.7055	0.3191	0.9061	0.6126	0.6383	0.7193	0.2838
CV-79	0.6909	0.2979	0.8950	0.5964	0.5957	0.7105	0.2431
CV-80	0.7091	0.3298	0.9061	0.6179	0.6458	0.7225	0.2947
CV-81	0.6945	0.3191	0.8895	0.6043	0.6000	0.7156	0.2566
CV-82	0.7018	0.3617	0.8785	0.6201	0.6071	0.7260	0.2829
CV-83	0.6982	0.3511	0.8785	0.6148	0.6000	0.7227	0.2722
CV-84	0.6945	0.2872	0.9061	0.5967	0.6136	0.7100	0.2501
CV-85	0.6982	0.3404	0.8840	0.6122	0.6038	0.7207	0.2698
CV-86	0.7055	0.3404	0.8950	0.6177	0.6275	0.7232	0.2873
CV-87	0.7018	0.3617	0.8785	0.6201	0.6071	0.7260	0.2829
CV-88	0.6873	0.3085	0.8840	0.5962	0.5800	0.7111	0.2367
CV-89	0.6909	0.3298	0.8785	0.6041	0.5849	0.7162	0.2504
CV-90	0.6945	0.3191	0.8895	0.6043	0.6000	0.7156	0.2566
CV-91	0.6982	0.3617	0.8729	0.6173	0.5965	0.7248	0.2746
CV-92	0.6727	0.2979	0.8674	0.5826	0.5385	0.7040	0.2002
CV-93	0.7091	0.3404	0.9006	0.6205	0.6400	0.7244	0.2963
CV-94	0.6945	0.3191	0.8895	0.6043	0.6000	0.7156	0.2566
CV-95	0.6836	0.2979	0.8840	0.5909	0.5714	0.7080	0.2254
CV-96	0.7055	0.3511	0.8895	0.6203	0.6226	0.7252	0.2893
CV-97	0.7091	0.3191	0.9116	0.6154	0.6522	0.7205	0.2933
CV-98	0.6873	0.3085	0.8840	0.5962	0.5800	0.7111	0.2367
CV-99	0.6873	0.3298	0.8729	0.6014	0.5741	0.7149	0.2420
CV-100	0.6909	0.3085	0.8895	0.5990	0.5918	0.7124	0.2454
CV-101	0.6836	0.2979	0.8840	0.5909	0.5714	0.7080	0.2254
CV-102	0.6836	0.3085	0.8785	0.5935	0.5686	0.7098	0.2282
CV-103	0.7018	0.3085	0.9061	0.6073	0.6304	0.7162	0.2727
CV-104	0.7055	0.3404	0.8950	0.6177	0.6275	0.7232	0.2873
CV-105	0.6764	0.2979	0.8729	0.5854	0.5490	0.7054	0.2084
CV-106	0.7018	0.3723	0.8729	0.6226	0.6034	0.7281	0.2852
CV-107	0.6945	0.2979	0.9006	0.5992	0.6087	0.7118	0.2522

CV-108	0.6945	0.3191	0.8895	0.6043	0.6000	0.7156	0.2566
CV-109	0.7127	0.3617	0.8950	0.6284	0.6415	0.7297	0.3087
CV-110	0.6873	0.3617	0.8564	0.6090	0.5667	0.7209	0.2504
CV-111	0.6909	0.3191	0.8840	0.6016	0.5882	0.7143	0.2479
CV-112	0.6764	0.2553	0.8950	0.5752	0.5581	0.6983	0.1963
CV-113	0.7018	0.3191	0.9006	0.6099	0.6250	0.7181	0.2745
CV-114	0.6945	0.3191	0.8895	0.6043	0.6000	0.7156	0.2566
CV-115	0.7200	0.3617	0.9061	0.6339	0.6667	0.7321	0.3268
CV-116	0.6982	0.3191	0.8950	0.6071	0.6122	0.7168	0.2655
CV-117	0.6873	0.3085	0.8840	0.5962	0.5800	0.7111	0.2367
CV-118	0.6909	0.3191	0.8840	0.6016	0.5882	0.7143	0.2479
CV-119	0.7091	0.3298	0.9061	0.6179	0.6458	0.7225	0.2947
CV-120	0.6873	0.3404	0.8674	0.6039	0.5714	0.7169	0.2448
CV-121	0.7200	0.3723	0.9006	0.6364	0.6604	0.7342	0.3282
CV-122	0.6909	0.3298	0.8785	0.6041	0.5849	0.7162	0.2504
CV-123	0.6800	0.3191	0.8674	0.5933	0.5556	0.7104	0.2227
CV-124	0.6909	0.2979	0.8950	0.5964	0.5957	0.7105	0.2431
CV-125	0.6945	0.3085	0.8950	0.6018	0.6042	0.7137	0.2543
CV-126	0.6982	0.3617	0.8729	0.6173	0.5965	0.7248	0.2746
CV-127	0.6800	0.3085	0.8729	0.5907	0.5577	0.7085	0.2198
CV-128	0.7018	0.3511	0.8840	0.6175	0.6111	0.7240	0.2806
CV-129	0.6873	0.3511	0.8619	0.6065	0.5690	0.7189	0.2476
CV-130	0.6873	0.2979	0.8895	0.5937	0.5833	0.7093	0.2341
CV-131	0.6909	0.3298	0.8785	0.6041	0.5849	0.7162	0.2504
CV-132	0.6945	0.3298	0.8840	0.6069	0.5962	0.7175	0.2589
CV-133	0.6873	0.2979	0.8895	0.5937	0.5833	0.7093	0.2341
CV-134	0.7018	0.3404	0.8895	0.6150	0.6154	0.7220	0.2785
CV-135	0.6873	0.2872	0.8950	0.5911	0.5870	0.7074	0.2316
CV-136	0.7018	0.3298	0.8950	0.6124	0.6200	0.7200	0.2765
CV-137	0.7055	0.3191	0.9061	0.6126	0.6383	0.7193	0.2838
CV-138	0.7055	0.3617	0.8840	0.6228	0.6182	0.7273	0.2913
CV-139	0.6945	0.3085	0.8950	0.6018	0.6042	0.7137	0.2543
CV-140	0.6873	0.2872	0.8950	0.5911	0.5870	0.7074	0.2316
CV-141	0.6800	0.2872	0.8840	0.5856	0.5625	0.7048	0.2139
CV-142	0.7018	0.3511	0.8840	0.6175	0.6111	0.7240	0.2806
CV-143	0.6727	0.2872	0.8729	0.5801	0.5400	0.7022	0.1970
CV-144	0.6727	0.3191	0.8564	0.5878	0.5357	0.7078	0.2067
CV-145	0.6982	0.3617	0.8729	0.6173	0.5965	0.7248	0.2746
CV-146	0.6873	0.2660	0.9061	0.5860	0.5952	0.7039	0.2268
CV-147	0.6945	0.3191	0.8895	0.6043	0.6000	0.7156	0.2566
CV-148	0.6873	0.2872	0.8950	0.5911	0.5870	0.7074	0.2316
CV-149	0.6800	0.3085	0.8729	0.5907	0.5577	0.7085	0.2198
CV-150	0.6909	0.2979	0.8950	0.5964	0.5957	0.7105	0.2431
CV-151	0.6982	0.3298	0.8895	0.6096	0.6078	0.7188	0.2676
CV-152	0.7091	0.3404	0.9006	0.6205	0.6400	0.7244	0.2963
CV-153	0.7018	0.3404	0.8895	0.6150	0.6154	0.7220	0.2785
CV-154	0.6909	0.3298	0.8785	0.6041	0.5849	0.7162	0.2504
CV-155	0.6909	0.3191	0.8840	0.6016	0.5882	0.7143	0.2479
CV-156	0.6945	0.3298	0.8840	0.6069	0.5962	0.7175	0.2589
CV-157	0.6982	0.3298	0.8895	0.6096	0.6078	0.7188	0.2676
CV-158	0.6982	0.3511	0.8785	0.6148	0.6000	0.7227	0.2722
CV-159	0.6764	0.2979	0.8729	0.5854	0.5490	0.7054	0.2084
CV-160	0.6691	0.2872	0.8674	0.5773	0.5294	0.7009	0.1887
CV-161	0.6836	0.3085	0.8785	0.5935	0.5686	0.7098	0.2282
CV-162	0.7018	0.3298	0.8950	0.6124	0.6200	0.7200	0.2765
CV-163	0.7018	0.3191	0.9006	0.6099	0.6250	0.7181	0.2745
CV-164	0.6873	0.3191	0.8785	0.5988	0.5769	0.7130	0.2394

CV-165	0.6836	0.3085	0.8785	0.5935	0.5686	0.7098	0.2282
CV-166	0.6982	0.3191	0.8950	0.6071	0.6122	0.7168	0.2655
CV-167	0.6836	0.3085	0.8785	0.5935	0.5686	0.7098	0.2282
CV-168	0.7018	0.3298	0.8950	0.6124	0.6200	0.7200	0.2765
CV-169	0.7018	0.3617	0.8785	0.6201	0.6071	0.7260	0.2829
CV-170	0.6982	0.2979	0.9061	0.6020	0.6222	0.7130	0.2615
CV-171	0.6764	0.2979	0.8729	0.5854	0.5490	0.7054	0.2084
CV-172	0.6909	0.3085	0.8895	0.5990	0.5918	0.7124	0.2454
CV-173	0.6618	0.2766	0.8619	0.5692	0.5098	0.6964	0.1690
CV-174	0.7164	0.3936	0.8840	0.6388	0.6379	0.7373	0.3228
CV-175	0.6873	0.2979	0.8895	0.5937	0.5833	0.7093	0.2341
CV-176	0.6945	0.3404	0.8785	0.6094	0.5926	0.7195	0.2613
CV-177	0.6982	0.3404	0.8840	0.6122	0.6038	0.7207	0.2698
CV-178	0.6945	0.3511	0.8729	0.6120	0.5893	0.7215	0.2638
CV-179	0.6945	0.2979	0.9006	0.5992	0.6087	0.7118	0.2522
CV-180	0.7055	0.3404	0.8950	0.6177	0.6275	0.7232	0.2873
CV-181	0.7164	0.3617	0.9006	0.6311	0.6538	0.7309	0.3177
CV-182	0.6764	0.2979	0.8729	0.5854	0.5490	0.7054	0.2084
CV-183	0.6691	0.2872	0.8674	0.5773	0.5294	0.7009	0.1887
CV-184	0.7055	0.3404	0.8950	0.6177	0.6275	0.7232	0.2873
CV-185	0.6945	0.2872	0.9061	0.5967	0.6136	0.7100	0.2501
CV-186	0.7055	0.3298	0.9006	0.6152	0.6327	0.7212	0.2855
CV-187	0.7091	0.3723	0.8840	0.6282	0.6250	0.7306	0.3019
CV-188	0.6800	0.2979	0.8785	0.5882	0.5600	0.7067	0.2168
CV-189	0.6982	0.3191	0.8950	0.6071	0.6122	0.7168	0.2655
CV-190	0.6800	0.2979	0.8785	0.5882	0.5600	0.7067	0.2168
CV-191	0.6655	0.3085	0.8508	0.5797	0.5179	0.7032	0.1877
CV-192	0.6727	0.2872	0.8729	0.5801	0.5400	0.7022	0.1970
CV-193	0.6691	0.2447	0.8895	0.5671	0.5349	0.6940	0.1752
CV-194	0.6945	0.3298	0.8840	0.6069	0.5962	0.7175	0.2589
CV-195	0.7018	0.3511	0.8840	0.6175	0.6111	0.7240	0.2806
CV-196	0.6873	0.2979	0.8895	0.5937	0.5833	0.7093	0.2341
CV-197	0.6982	0.2872	0.9116	0.5994	0.6279	0.7112	0.2597
CV-198	0.7055	0.3617	0.8840	0.6228	0.6182	0.7273	0.2913
CV-199	0.6836	0.2766	0.8950	0.5858	0.5778	0.7043	0.2200
CV-200	0.6691	0.2766	0.8729	0.5748	0.5306	0.6991	0.1853
CV-201	0.6836	0.3085	0.8785	0.5935	0.5686	0.7098	0.2282
CV-202	0.7091	0.3298	0.9061	0.6179	0.6458	0.7225	0.2947
CV-203	0.6873	0.3298	0.8729	0.6014	0.5741	0.7149	0.2420
CV-204	0.7018	0.3404	0.8895	0.6150	0.6154	0.7220	0.2785
CV-205	0.6982	0.3511	0.8785	0.6148	0.6000	0.7227	0.2722
CV-206	0.7018	0.3617	0.8785	0.6201	0.6071	0.7260	0.2829
CV-207	0.6873	0.3298	0.8729	0.6014	0.5741	0.7149	0.2420
CV-208	0.6836	0.3085	0.8785	0.5935	0.5686	0.7098	0.2282
CV-209	0.6909	0.3191	0.8840	0.6016	0.5882	0.7143	0.2479
CV-210	0.6909	0.3191	0.8840	0.6016	0.5882	0.7143	0.2479
CV-211	0.6982	0.3298	0.8895	0.6096	0.6078	0.7188	0.2676
CV-212	0.6909	0.3191	0.8840	0.6016	0.5882	0.7143	0.2479
CV-213	0.6873	0.3191	0.8785	0.5988	0.5769	0.7130	0.2394
CV-214	0.7127	0.3617	0.8950	0.6284	0.6415	0.7297	0.3087
CV-215	0.6982	0.3511	0.8785	0.6148	0.6000	0.7227	0.2722
CV-216	0.6909	0.3085	0.8895	0.5990	0.5918	0.7124	0.2454
CV-217	0.6800	0.2766	0.8895	0.5830	0.5652	0.7031	0.2111
CV-218	0.6873	0.3511	0.8619	0.6065	0.5690	0.7189	0.2476
CV-219	0.6945	0.3511	0.8729	0.6120	0.5893	0.7215	0.2638
CV-220	0.6982	0.3404	0.8840	0.6122	0.6038	0.7207	0.2698
CV-221	0.7164	0.3511	0.9061	0.6286	0.6600	0.7289	0.3162

CV-222	0.6691	0.2660	0.8785	0.5722	0.5319	0.6974	0.1820
CV-223	0.6764	0.2872	0.8785	0.5828	0.5510	0.7035	0.2054
CV-224	0.7055	0.3617	0.8840	0.6228	0.6182	0.7273	0.2913
CV-225	0.6764	0.3085	0.8674	0.5880	0.5472	0.7072	0.2115
CV-226	0.6873	0.2979	0.8895	0.5937	0.5833	0.7093	0.2341
CV-227	0.6909	0.3511	0.8674	0.6092	0.5789	0.7202	0.2556
CV-228	0.6945	0.2979	0.9006	0.5992	0.6087	0.7118	0.2522
CV-229	0.6945	0.3404	0.8785	0.6094	0.5926	0.7195	0.2613
CV-230	0.6691	0.2872	0.8674	0.5773	0.5294	0.7009	0.1887
CV-231	0.6945	0.3511	0.8729	0.6120	0.5893	0.7215	0.2638
CV-232	0.6909	0.3085	0.8895	0.5990	0.5918	0.7124	0.2454
CV-233	0.7018	0.3191	0.9006	0.6099	0.6250	0.7181	0.2745
CV-234	0.6800	0.2872	0.8840	0.5856	0.5625	0.7048	0.2139
CV-235	0.6764	0.3191	0.8619	0.5905	0.5455	0.7091	0.2147
CV-236	0.6945	0.3404	0.8785	0.6094	0.5926	0.7195	0.2613
CV-237	0.6982	0.3404	0.8840	0.6122	0.6038	0.7207	0.2698
CV-238	0.6764	0.3085	0.8674	0.5880	0.5472	0.7072	0.2115
CV-239	0.6800	0.3298	0.8619	0.5958	0.5536	0.7123	0.2258
CV-240	0.7127	0.3511	0.9006	0.6258	0.6471	0.7277	0.3071
CV-241	0.6909	0.2979	0.8950	0.5964	0.5957	0.7105	0.2431
CV-242	0.7018	0.3298	0.8950	0.6124	0.6200	0.7200	0.2765
CV-243	0.6873	0.3404	0.8674	0.6039	0.5714	0.7169	0.2448
CV-244	0.6800	0.2979	0.8785	0.5882	0.5600	0.7067	0.2168
CV-245	0.7091	0.3298	0.9061	0.6179	0.6458	0.7225	0.2947
CV-246	0.6909	0.3191	0.8840	0.6016	0.5882	0.7143	0.2479
CV-247	0.6800	0.2872	0.8840	0.5856	0.5625	0.7048	0.2139
CV-248	0.7091	0.3723	0.8840	0.6282	0.6250	0.7306	0.3019
CV-249	0.6618	0.2447	0.8785	0.5616	0.5111	0.6913	0.1579
CV-250	0.7127	0.3723	0.8895	0.6309	0.6364	0.7318	0.3105
CV-251	0.6982	0.3404	0.8840	0.6122	0.6038	0.7207	0.2698
CV-252	0.6800	0.3085	0.8729	0.5907	0.5577	0.7085	0.2198
CV-253	0.6982	0.3085	0.9006	0.6045	0.6170	0.7149	0.2634
CV-254	0.6800	0.3085	0.8729	0.5907	0.5577	0.7085	0.2198
CV-255	0.6982	0.3511	0.8785	0.6148	0.6000	0.7227	0.2722
CV-256	0.6800	0.3191	0.8674	0.5933	0.5556	0.7104	0.2227
CV-257	0.6909	0.3191	0.8840	0.6016	0.5882	0.7143	0.2479
CV-258	0.6909	0.3298	0.8785	0.6041	0.5849	0.7162	0.2504
CV-259	0.6836	0.3191	0.8729	0.5960	0.5660	0.7117	0.2310
CV-260	0.6655	0.2553	0.8785	0.5669	0.5217	0.6943	0.1700
CV-261	0.7018	0.2872	0.9171	0.6022	0.6429	0.7124	0.2695
CV-262	0.6655	0.2766	0.8674	0.5720	0.5200	0.6978	0.1771
CV-263	0.6982	0.2979	0.9061	0.6020	0.6222	0.7130	0.2615
CV-264	0.6909	0.3191	0.8840	0.6016	0.5882	0.7143	0.2479
CV-265	0.6764	0.2979	0.8729	0.5854	0.5490	0.7054	0.2084
CV-266	0.6945	0.3511	0.8729	0.6120	0.5893	0.7215	0.2638
CV-267	0.6945	0.3617	0.8674	0.6146	0.5862	0.7235	0.2664
CV-268	0.7018	0.3085	0.9061	0.6073	0.6304	0.7162	0.2727
CV-269	0.6800	0.2766	0.8895	0.5830	0.5652	0.7031	0.2111
CV-270	0.6873	0.2979	0.8895	0.5937	0.5833	0.7093	0.2341
CV-271	0.6945	0.3298	0.8840	0.6069	0.5962	0.7175	0.2589
CV-272	0.6764	0.3191	0.8619	0.5905	0.5455	0.7091	0.2147
CV-273	0.7018	0.3404	0.8895	0.6150	0.6154	0.7220	0.2785
CV-274	0.6873	0.3085	0.8840	0.5962	0.5800	0.7111	0.2367
CV-275	0.6945	0.3511	0.8729	0.6120	0.5893	0.7215	0.2638
CV-276	0.7055	0.3191	0.9061	0.6126	0.6383	0.7193	0.2838
CV-277	0.7091	0.3511	0.8950	0.6230	0.6346	0.7265	0.2981
CV-278	0.6836	0.3191	0.8729	0.5960	0.5660	0.7117	0.2310

CV-279	0.6691	0.2660	0.8785	0.5722	0.5319	0.6974	0.1820
CV-280	0.6873	0.3298	0.8729	0.6014	0.5741	0.7149	0.2420
CV-281	0.7091	0.3085	0.9171	0.6128	0.6591	0.7186	0.2919
CV-282	0.6836	0.3191	0.8729	0.5960	0.5660	0.7117	0.2310
CV-283	0.6836	0.3085	0.8785	0.5935	0.5686	0.7098	0.2282
CV-284	0.6982	0.3191	0.8950	0.6071	0.6122	0.7168	0.2655
CV-285	0.7018	0.3511	0.8840	0.6175	0.6111	0.7240	0.2806
CV-286	0.7091	0.3617	0.8895	0.6256	0.6296	0.7285	0.2999
CV-287	0.6764	0.3404	0.8508	0.5956	0.5424	0.7130	0.2210
CV-288	0.6982	0.3298	0.8895	0.6096	0.6078	0.7188	0.2676
CV-289	0.6945	0.3085	0.8950	0.6018	0.6042	0.7137	0.2543
CV-290	0.6982	0.3511	0.8785	0.6148	0.6000	0.7227	0.2722
CV-291	0.6982	0.3298	0.8895	0.6096	0.6078	0.7188	0.2676
CV-292	0.6800	0.3085	0.8729	0.5907	0.5577	0.7085	0.2198
CV-293	0.7127	0.3298	0.9116	0.6207	0.6596	0.7237	0.3042
CV-294	0.6836	0.2979	0.8840	0.5909	0.5714	0.7080	0.2254
CV-295	0.6982	0.3191	0.8950	0.6071	0.6122	0.7168	0.2655
CV-296	0.6909	0.3191	0.8840	0.6016	0.5882	0.7143	0.2479
CV-297	0.7055	0.3511	0.8895	0.6203	0.6226	0.7252	0.2893
CV-298	0.6727	0.2872	0.8729	0.5801	0.5400	0.7022	0.1970
CV-299	0.7164	0.3404	0.9116	0.6260	0.6667	0.7269	0.3149
CV-300	0.6945	0.3298	0.8840	0.6069	0.5962	0.7175	0.2589
CV-301	0.6836	0.3298	0.8674	0.5986	0.5636	0.7136	0.2338
CV-302	0.6873	0.2979	0.8895	0.5937	0.5833	0.7093	0.2341
CV-303	0.6909	0.3298	0.8785	0.6041	0.5849	0.7162	0.2504
CV-304	0.6800	0.2872	0.8840	0.5856	0.5625	0.7048	0.2139
CV-305	0.7018	0.3404	0.8895	0.6150	0.6154	0.7220	0.2785
CV-306	0.7164	0.3511	0.9061	0.6286	0.6600	0.7289	0.3162
CV-307	0.7127	0.3511	0.9006	0.6258	0.6471	0.7277	0.3071
CV-308	0.6764	0.3085	0.8674	0.5880	0.5472	0.7072	0.2115
CV-309	0.7055	0.3617	0.8840	0.6228	0.6182	0.7273	0.2913
CV-310	0.6982	0.3404	0.8840	0.6122	0.6038	0.7207	0.2698
CV-311	0.6836	0.3085	0.8785	0.5935	0.5686	0.7098	0.2282
CV-312	0.7055	0.2979	0.9171	0.6075	0.6512	0.7155	0.2808
CV-313	0.7200	0.3617	0.9061	0.6339	0.6667	0.7321	0.3268
CV-314	0.7018	0.3511	0.8840	0.6175	0.6111	0.7240	0.2806
CV-315	0.7055	0.3404	0.8950	0.6177	0.6275	0.7232	0.2873
CV-316	0.6909	0.3511	0.8674	0.6092	0.5789	0.7202	0.2556
CV-317	0.6909	0.3404	0.8729	0.6067	0.5818	0.7182	0.2530
CV-318	0.7200	0.3511	0.9116	0.6313	0.6735	0.7301	0.3256
CV-319	0.6764	0.2660	0.8895	0.5777	0.5556	0.7000	0.1993
CV-320	0.6982	0.3404	0.8840	0.6122	0.6038	0.7207	0.2698
CV-321	0.7127	0.3617	0.8950	0.6284	0.6415	0.7297	0.3087
CV-322	0.6945	0.3404	0.8785	0.6094	0.5926	0.7195	0.2613
CV-323	0.6764	0.2766	0.8840	0.5803	0.5532	0.7018	0.2023
CV-324	0.6873	0.3191	0.8785	0.5988	0.5769	0.7130	0.2394
CV-325	0.7018	0.3298	0.8950	0.6124	0.6200	0.7200	0.2765
CV-326	0.7055	0.3404	0.8950	0.6177	0.6275	0.7232	0.2873
CV-327	0.6945	0.3085	0.8950	0.6018	0.6042	0.7137	0.2543
CV-328	0.6655	0.2766	0.8674	0.5720	0.5200	0.6978	0.1771
CV-329	0.7127	0.3723	0.8895	0.6309	0.6364	0.7318	0.3105
CV-330	0.6945	0.3191	0.8895	0.6043	0.6000	0.7156	0.2566
CV-331	0.6945	0.3404	0.8785	0.6094	0.5926	0.7195	0.2613
CV-332	0.6727	0.3085	0.8619	0.5852	0.5370	0.7059	0.2034
CV-333	0.6982	0.2979	0.9061	0.6020	0.6222	0.7130	0.2615
CV-334	0.7018	0.3191	0.9006	0.6099	0.6250	0.7181	0.2745
CV-335	0.7164	0.3511	0.9061	0.6286	0.6600	0.7289	0.3162

CV-336	0.6982	0.3511	0.8785	0.6148	0.6000	0.7227	0.2722
CV-337	0.7055	0.3191	0.9061	0.6126	0.6383	0.7193	0.2838
CV-338	0.6727	0.3298	0.8508	0.5903	0.5345	0.7097	0.2100
CV-339	0.7055	0.3298	0.9006	0.6152	0.6327	0.7212	0.2855
CV-340	0.6945	0.3085	0.8950	0.6018	0.6042	0.7137	0.2543
CV-341	0.7018	0.3191	0.9006	0.6099	0.6250	0.7181	0.2745
CV-342	0.7091	0.3617	0.8895	0.6256	0.6296	0.7285	0.2999
CV-343	0.7055	0.3404	0.8950	0.6177	0.6275	0.7232	0.2873
CV-344	0.6982	0.3298	0.8895	0.6096	0.6078	0.7188	0.2676
CV-345	0.6691	0.2872	0.8674	0.5773	0.5294	0.7009	0.1887
CV-346	0.7091	0.3298	0.9061	0.6179	0.6458	0.7225	0.2947
CV-347	0.6945	0.3617	0.8674	0.6146	0.5862	0.7235	0.2664
CV-348	0.6655	0.2872	0.8619	0.5746	0.5192	0.6996	0.1806
CV-349	0.7200	0.3617	0.9061	0.6339	0.6667	0.7321	0.3268
CV-350	0.6873	0.2979	0.8895	0.5937	0.5833	0.7093	0.2341
CV-351	0.6945	0.3298	0.8840	0.6069	0.5962	0.7175	0.2589
CV-352	0.6800	0.3085	0.8729	0.5907	0.5577	0.7085	0.2198
CV-353	0.7018	0.3404	0.8895	0.6150	0.6154	0.7220	0.2785
CV-354	0.6836	0.2979	0.8840	0.5909	0.5714	0.7080	0.2254
CV-355	0.7018	0.3511	0.8840	0.6175	0.6111	0.7240	0.2806
CV-356	0.6909	0.3085	0.8895	0.5990	0.5918	0.7124	0.2454
CV-357	0.6909	0.2979	0.8950	0.5964	0.5957	0.7105	0.2431
CV-358	0.6909	0.2979	0.8950	0.5964	0.5957	0.7105	0.2431
CV-359	0.6764	0.2872	0.8785	0.5828	0.5510	0.7035	0.2054
CV-360	0.6909	0.3298	0.8785	0.6041	0.5849	0.7162	0.2504
CV-361	0.6618	0.2447	0.8785	0.5616	0.5111	0.6913	0.1579
CV-362	0.6836	0.3085	0.8785	0.5935	0.5686	0.7098	0.2282
CV-363	0.7018	0.3511	0.8840	0.6175	0.6111	0.7240	0.2806
CV-364	0.7018	0.3298	0.8950	0.6124	0.6200	0.7200	0.2765
CV-365	0.6691	0.3085	0.8564	0.5824	0.5273	0.7045	0.1955
CV-366	0.6909	0.3191	0.8840	0.6016	0.5882	0.7143	0.2479
CV-367	0.6836	0.3191	0.8729	0.5960	0.5660	0.7117	0.2310
CV-368	0.7127	0.3404	0.9061	0.6233	0.6531	0.7257	0.3055
CV-369	0.6764	0.3085	0.8674	0.5880	0.5472	0.7072	0.2115
CV-370	0.7164	0.3830	0.8895	0.6362	0.6429	0.7352	0.3209
CV-371	0.6764	0.2553	0.8950	0.5752	0.5581	0.6983	0.1963
CV-372	0.6691	0.2553	0.8840	0.5696	0.5333	0.6957	0.1786
CV-373	0.7018	0.3191	0.9006	0.6099	0.6250	0.7181	0.2745
CV-374	0.7091	0.3511	0.8950	0.6230	0.6346	0.7265	0.2981
CV-375	0.6873	0.2979	0.8895	0.5937	0.5833	0.7093	0.2341
CV-376	0.6873	0.3404	0.8674	0.6039	0.5714	0.7169	0.2448
CV-377	0.6473	0.2553	0.8508	0.5531	0.4706	0.6875	0.1295
CV-378	0.6800	0.2872	0.8840	0.5856	0.5625	0.7048	0.2139
CV-379	0.6909	0.3298	0.8785	0.6041	0.5849	0.7162	0.2504
CV-380	0.6982	0.3191	0.8950	0.6071	0.6122	0.7168	0.2655
CV-381	0.7018	0.3511	0.8840	0.6175	0.6111	0.7240	0.2806
CV-382	0.7018	0.3404	0.8895	0.6150	0.6154	0.7220	0.2785
CV-383	0.6764	0.3085	0.8674	0.5880	0.5472	0.7072	0.2115
CV-384	0.6945	0.3404	0.8785	0.6094	0.5926	0.7195	0.2613
CV-385	0.6909	0.3298	0.8785	0.6041	0.5849	0.7162	0.2504
CV-386	0.6800	0.3298	0.8619	0.5958	0.5536	0.7123	0.2258
CV-387	0.6764	0.2979	0.8729	0.5854	0.5490	0.7054	0.2084
CV-388	0.7018	0.3511	0.8840	0.6175	0.6111	0.7240	0.2806
CV-389	0.7164	0.3936	0.8840	0.6388	0.6379	0.7373	0.3228
CV-390	0.7018	0.3617	0.8785	0.6201	0.6071	0.7260	0.2829
CV-391	0.6945	0.3085	0.8950	0.6018	0.6042	0.7137	0.2543
CV-392	0.6836	0.2979	0.8840	0.5909	0.5714	0.7080	0.2254

CV-393	0.6909	0.3191	0.8840	0.6016	0.5882	0.7143	0.2479
CV-394	0.6836	0.3191	0.8729	0.5960	0.5660	0.7117	0.2310
CV-395	0.6945	0.3298	0.8840	0.6069	0.5962	0.7175	0.2589
CV-396	0.6873	0.2979	0.8895	0.5937	0.5833	0.7093	0.2341
CV-397	0.7018	0.3511	0.8840	0.6175	0.6111	0.7240	0.2806
CV-398	0.6945	0.3404	0.8785	0.6094	0.5926	0.7195	0.2613
CV-399	0.7127	0.3085	0.9227	0.6156	0.6744	0.7198	0.3019
CV-400	0.7055	0.3404	0.8950	0.6177	0.6275	0.7232	0.2873
CV-401	0.6945	0.3404	0.8785	0.6094	0.5926	0.7195	0.2613
CV-402	0.6945	0.3298	0.8840	0.6069	0.5962	0.7175	0.2589
CV-403	0.6655	0.2660	0.8729	0.5694	0.5208	0.6960	0.1736
CV-404	0.6945	0.3191	0.8895	0.6043	0.6000	0.7156	0.2566
CV-405	0.7127	0.3511	0.9006	0.6258	0.6471	0.7277	0.3071
CV-406	0.6800	0.2979	0.8785	0.5882	0.5600	0.7067	0.2168
CV-407	0.6800	0.2872	0.8840	0.5856	0.5625	0.7048	0.2139
CV-408	0.6945	0.3404	0.8785	0.6094	0.5926	0.7195	0.2613
CV-409	0.7055	0.3404	0.8950	0.6177	0.6275	0.7232	0.2873
CV-410	0.6836	0.3298	0.8674	0.5986	0.5636	0.7136	0.2338
CV-411	0.7055	0.3617	0.8840	0.6228	0.6182	0.7273	0.2913
CV-412	0.6982	0.3723	0.8674	0.6199	0.5932	0.7269	0.2770
CV-413	0.6691	0.3191	0.8508	0.5850	0.5263	0.7064	0.1989
CV-414	0.6982	0.3085	0.9006	0.6045	0.6170	0.7149	0.2634
CV-415	0.6727	0.2553	0.8895	0.5724	0.5455	0.6970	0.1874
CV-416	0.6800	0.2979	0.8785	0.5882	0.5600	0.7067	0.2168
CV-417	0.6836	0.2872	0.8895	0.5884	0.5745	0.7061	0.2227
CV-418	0.6836	0.2979	0.8840	0.5909	0.5714	0.7080	0.2254
CV-419	0.6909	0.3511	0.8674	0.6092	0.5789	0.7202	0.2556
CV-420	0.6909	0.3298	0.8785	0.6041	0.5849	0.7162	0.2504
CV-421	0.7127	0.3404	0.9061	0.6233	0.6531	0.7257	0.3055
CV-422	0.6800	0.3298	0.8619	0.5958	0.5536	0.7123	0.2258
CV-423	0.6945	0.3617	0.8674	0.6146	0.5862	0.7235	0.2664
CV-424	0.7164	0.3404	0.9116	0.6260	0.6667	0.7269	0.3149
CV-425	0.6764	0.2872	0.8785	0.5828	0.5510	0.7035	0.2054
CV-426	0.6982	0.3191	0.8950	0.6071	0.6122	0.7168	0.2655
CV-427	0.6800	0.3191	0.8674	0.5933	0.5556	0.7104	0.2227
CV-428	0.6945	0.3085	0.8950	0.6018	0.6042	0.7137	0.2543
CV-429	0.6764	0.2660	0.8895	0.5777	0.5556	0.7000	0.1993
CV-430	0.7018	0.3085	0.9061	0.6073	0.6304	0.7162	0.2727
CV-431	0.6727	0.3085	0.8619	0.5852	0.5370	0.7059	0.2034
CV-432	0.7018	0.3511	0.8840	0.6175	0.6111	0.7240	0.2806
CV-433	0.6836	0.2872	0.8895	0.5884	0.5745	0.7061	0.2227
CV-434	0.6945	0.3298	0.8840	0.6069	0.5962	0.7175	0.2589
CV-435	0.6945	0.3298	0.8840	0.6069	0.5962	0.7175	0.2589
CV-436	0.6982	0.3298	0.8895	0.6096	0.6078	0.7188	0.2676
CV-437	0.6909	0.3085	0.8895	0.5990	0.5918	0.7124	0.2454
CV-438	0.6945	0.3298	0.8840	0.6069	0.5962	0.7175	0.2589
CV-439	0.7091	0.3404	0.9006	0.6205	0.6400	0.7244	0.2963
CV-440	0.6873	0.3191	0.8785	0.5988	0.5769	0.7130	0.2394
CV-441	0.6982	0.3617	0.8729	0.6173	0.5965	0.7248	0.2746
CV-442	0.6945	0.3298	0.8840	0.6069	0.5962	0.7175	0.2589
CV-443	0.7091	0.3723	0.8840	0.6282	0.6250	0.7306	0.3019
CV-444	0.6764	0.2766	0.8840	0.5803	0.5532	0.7018	0.2023
CV-445	0.7055	0.3404	0.8950	0.6177	0.6275	0.7232	0.2873
CV-446	0.6618	0.2660	0.8674	0.5667	0.5102	0.6947	0.1653
CV-447	0.7055	0.3617	0.8840	0.6228	0.6182	0.7273	0.2913
CV-448	0.7127	0.3404	0.9061	0.6233	0.6531	0.7257	0.3055
CV-449	0.6909	0.3298	0.8785	0.6041	0.5849	0.7162	0.2504

CV-450	0.6873	0.2872	0.8950	0.5911	0.5870	0.7074	0.2316
CV-451	0.6873	0.3298	0.8729	0.6014	0.5741	0.7149	0.2420
CV-452	0.7127	0.3723	0.8895	0.6309	0.6364	0.7318	0.3105
CV-453	0.7091	0.3404	0.9006	0.6205	0.6400	0.7244	0.2963
CV-454	0.6691	0.2872	0.8674	0.5773	0.5294	0.7009	0.1887
CV-455	0.6836	0.3191	0.8729	0.5960	0.5660	0.7117	0.2310
CV-456	0.7091	0.3617	0.8895	0.6256	0.6296	0.7285	0.2999
CV-457	0.7091	0.3404	0.9006	0.6205	0.6400	0.7244	0.2963
CV-458	0.6727	0.2979	0.8674	0.5826	0.5385	0.7040	0.2002
CV-459	0.6909	0.3085	0.8895	0.5990	0.5918	0.7124	0.2454
CV-460	0.6909	0.3404	0.8729	0.6067	0.5818	0.7182	0.2530
CV-461	0.7018	0.3404	0.8895	0.6150	0.6154	0.7220	0.2785
CV-462	0.6836	0.3085	0.8785	0.5935	0.5686	0.7098	0.2282
CV-463	0.7127	0.3723	0.8895	0.6309	0.6364	0.7318	0.3105
CV-464	0.6982	0.3404	0.8840	0.6122	0.6038	0.7207	0.2698
CV-465	0.6909	0.3298	0.8785	0.6041	0.5849	0.7162	0.2504
CV-466	0.6836	0.3085	0.8785	0.5935	0.5686	0.7098	0.2282
CV-467	0.6836	0.2660	0.9006	0.5833	0.5814	0.7026	0.2175
CV-468	0.6945	0.3085	0.8950	0.6018	0.6042	0.7137	0.2543
CV-469	0.6764	0.3191	0.8619	0.5905	0.5455	0.7091	0.2147
CV-470	0.6909	0.2979	0.8950	0.5964	0.5957	0.7105	0.2431
CV-471	0.6691	0.2979	0.8619	0.5799	0.5283	0.7027	0.1921
CV-472	0.6982	0.3404	0.8840	0.6122	0.6038	0.7207	0.2698
CV-473	0.7055	0.3511	0.8895	0.6203	0.6226	0.7252	0.2893
CV-474	0.7055	0.3830	0.8729	0.6280	0.6102	0.7315	0.2957
CV-475	0.6836	0.3085	0.8785	0.5935	0.5686	0.7098	0.2282
CV-476	0.6945	0.3511	0.8729	0.6120	0.5893	0.7215	0.2638
CV-477	0.6800	0.3085	0.8729	0.5907	0.5577	0.7085	0.2198
CV-478	0.7018	0.3085	0.9061	0.6073	0.6304	0.7162	0.2727
CV-479	0.7018	0.3191	0.9006	0.6099	0.6250	0.7181	0.2745
CV-480	0.6873	0.3191	0.8785	0.5988	0.5769	0.7130	0.2394
CV-481	0.6873	0.3085	0.8840	0.5962	0.5800	0.7111	0.2367
CV-482	0.7091	0.3617	0.8895	0.6256	0.6296	0.7285	0.2999
CV-483	0.7055	0.3404	0.8950	0.6177	0.6275	0.7232	0.2873
CV-484	0.6618	0.2660	0.8674	0.5667	0.5102	0.6947	0.1653
CV-485	0.6800	0.2979	0.8785	0.5882	0.5600	0.7067	0.2168
CV-486	0.6873	0.3298	0.8729	0.6014	0.5741	0.7149	0.2420
CV-487	0.6836	0.3298	0.8674	0.5986	0.5636	0.7136	0.2338
CV-488	0.6945	0.3191	0.8895	0.6043	0.6000	0.7156	0.2566
CV-489	0.6945	0.3298	0.8840	0.6069	0.5962	0.7175	0.2589
CV-490	0.6764	0.3085	0.8674	0.5880	0.5472	0.7072	0.2115
CV-491	0.7018	0.3298	0.8950	0.6124	0.6200	0.7200	0.2765
CV-492	0.7091	0.3830	0.8785	0.6307	0.6207	0.7327	0.3040
CV-493	0.6764	0.2979	0.8729	0.5854	0.5490	0.7054	0.2084
CV-494	0.6873	0.3298	0.8729	0.6014	0.5741	0.7149	0.2420
CV-495	0.7055	0.3511	0.8895	0.6203	0.6226	0.7252	0.2893
CV-496	0.7164	0.3404	0.9116	0.6260	0.6667	0.7269	0.3149
CV-497	0.6764	0.3191	0.8619	0.5905	0.5455	0.7091	0.2147
CV-498	0.6909	0.3085	0.8895	0.5990	0.5918	0.7124	0.2454
CV-499	0.7018	0.3085	0.9061	0.6073	0.6304	0.7162	0.2727
CV-500	0.7055	0.3404	0.8950	0.6177	0.6275	0.7232	0.2873

Table S8. Performance of 5-fold cross-validations from support vector machine (SVM). Performance of 5-fold cross-validations were evaluated by seven performance metrics. Each row shows the performance of one time 5-fold cross-validation. 5-fold cross-validation was repeated 500 times (CV-1 ~ CV-500).

Cross-validation	Accuracy	Sensitivity	Specificity	Balanced accuracy	Positive prediction rate	Negative prediction rate	Matthews correlation coefficient
CV-1	0.7091	0.4362	0.8508	0.6435	0.6029	0.7440	0.3155
CV-2	0.6727	0.4149	0.8066	0.6108	0.5270	0.7264	0.2369
CV-3	0.7127	0.4787	0.8343	0.6565	0.6000	0.7550	0.3333
CV-4	0.6655	0.3936	0.8066	0.6001	0.5139	0.7192	0.2161
CV-5	0.6800	0.4362	0.8066	0.6214	0.5395	0.7337	0.2575
CV-6	0.6945	0.4787	0.8066	0.6427	0.5625	0.7487	0.2980
CV-7	0.6691	0.4149	0.8011	0.6080	0.5200	0.7250	0.2300
CV-8	0.6655	0.4255	0.7901	0.6078	0.5128	0.7259	0.2269
CV-9	0.6909	0.4149	0.8343	0.6246	0.5652	0.7330	0.2726
CV-10	0.6655	0.3936	0.8066	0.6001	0.5139	0.7192	0.2161
CV-11	0.6836	0.4574	0.8011	0.6293	0.5443	0.7398	0.2710
CV-12	0.6582	0.3936	0.7956	0.5946	0.5000	0.7164	0.2024
CV-13	0.6764	0.4362	0.8011	0.6186	0.5325	0.7323	0.2507
CV-14	0.6873	0.4681	0.8011	0.6346	0.5500	0.7436	0.2811
CV-15	0.6655	0.4043	0.8011	0.6027	0.5135	0.7214	0.2196
CV-16	0.6945	0.4255	0.8343	0.6299	0.5714	0.7366	0.2829
CV-17	0.7091	0.4362	0.8508	0.6435	0.6029	0.7440	0.3155
CV-18	0.6836	0.4149	0.8232	0.6190	0.5493	0.7304	0.2581
CV-19	0.6545	0.3936	0.7901	0.5918	0.4933	0.7150	0.1956
CV-20	0.6945	0.4255	0.8343	0.6299	0.5714	0.7366	0.2829
CV-21	0.6655	0.4574	0.7735	0.6155	0.5119	0.7330	0.2378
CV-22	0.6909	0.4362	0.8232	0.6297	0.5616	0.7376	0.2786
CV-23	0.6982	0.4255	0.8398	0.6327	0.5797	0.7379	0.2903
CV-24	0.6982	0.4255	0.8398	0.6327	0.5797	0.7379	0.2903
CV-25	0.6873	0.4468	0.8122	0.6295	0.5526	0.7387	0.2747
CV-26	0.6909	0.4149	0.8343	0.6246	0.5652	0.7330	0.2726
CV-27	0.6800	0.4362	0.8066	0.6214	0.5395	0.7337	0.2575
CV-28	0.6945	0.4574	0.8177	0.6376	0.5658	0.7437	0.2918
CV-29	0.6727	0.3723	0.8287	0.6005	0.5303	0.7177	0.2233
CV-30	0.6655	0.4362	0.7845	0.6104	0.5125	0.7282	0.2305
CV-31	0.6691	0.4043	0.8066	0.6054	0.5205	0.7228	0.2265
CV-32	0.6545	0.4362	0.7680	0.6021	0.4940	0.7240	0.2109
CV-33	0.6873	0.4362	0.8177	0.6269	0.5541	0.7363	0.2715
CV-34	0.6545	0.4043	0.7845	0.5944	0.4935	0.7172	0.1994
CV-35	0.6436	0.3936	0.7735	0.5835	0.4744	0.7107	0.1758
CV-36	0.7018	0.4681	0.8232	0.6456	0.5789	0.7487	0.3090
CV-37	0.6800	0.4149	0.8177	0.6163	0.5417	0.7291	0.2509
CV-38	0.6618	0.4149	0.7901	0.6025	0.5065	0.7222	0.2165
CV-39	0.6545	0.3830	0.7956	0.5893	0.4932	0.7129	0.1918
CV-40	0.6509	0.4043	0.7790	0.5916	0.4872	0.7157	0.1928
CV-41	0.6873	0.3830	0.8453	0.6141	0.5625	0.7251	0.2562
CV-42	0.6945	0.5106	0.7901	0.6503	0.5581	0.7566	0.3076
CV-43	0.6764	0.4468	0.7956	0.6212	0.5316	0.7347	0.2541
CV-44	0.7091	0.4574	0.8398	0.6486	0.5972	0.7488	0.3207
CV-45	0.6582	0.3936	0.7956	0.5946	0.5000	0.7164	0.2024
CV-46	0.6764	0.4468	0.7956	0.6212	0.5316	0.7347	0.2541
CV-47	0.6655	0.4255	0.7901	0.6078	0.5128	0.7259	0.2269
CV-48	0.6836	0.4362	0.8122	0.6242	0.5467	0.7350	0.2645
CV-49	0.6800	0.4362	0.8066	0.6214	0.5395	0.7337	0.2575
CV-50	0.7164	0.4468	0.8564	0.6516	0.6176	0.7488	0.3333

CV-51	0.7018	0.4574	0.8287	0.6431	0.5811	0.7463	0.3061
CV-52	0.6873	0.4149	0.8287	0.6218	0.5571	0.7317	0.2653
CV-53	0.7236	0.5106	0.8343	0.6724	0.6154	0.7665	0.3629
CV-54	0.6764	0.4681	0.7845	0.6263	0.5301	0.7396	0.2610
CV-55	0.6691	0.4362	0.7901	0.6131	0.5190	0.7296	0.2371
CV-56	0.6836	0.4149	0.8232	0.6190	0.5493	0.7304	0.2581
CV-57	0.6873	0.4149	0.8287	0.6218	0.5571	0.7317	0.2653
CV-58	0.6945	0.4149	0.8398	0.6273	0.5735	0.7343	0.2800
CV-59	0.6873	0.4894	0.7901	0.6397	0.5476	0.7487	0.2877
CV-60	0.6836	0.4149	0.8232	0.6190	0.5493	0.7304	0.2581
CV-61	0.6982	0.4255	0.8398	0.6327	0.5797	0.7379	0.2903
CV-62	0.6836	0.4043	0.8287	0.6165	0.5507	0.7282	0.2549
CV-63	0.7018	0.4894	0.8122	0.6508	0.5750	0.7538	0.3149
CV-64	0.6764	0.4574	0.7901	0.6238	0.5309	0.7371	0.2575
CV-65	0.6909	0.4043	0.8398	0.6220	0.5672	0.7308	0.2696
CV-66	0.6655	0.3404	0.8343	0.5873	0.5161	0.7089	0.1983
CV-67	0.6873	0.4149	0.8287	0.6218	0.5571	0.7317	0.2653
CV-68	0.6764	0.4255	0.8066	0.6161	0.5333	0.7300	0.2473
CV-69	0.6582	0.4043	0.7901	0.5972	0.5000	0.7186	0.2061
CV-70	0.6836	0.4043	0.8287	0.6165	0.5507	0.7282	0.2549
CV-71	0.6909	0.4255	0.8287	0.6271	0.5634	0.7353	0.2756
CV-72	0.6509	0.4149	0.7735	0.5942	0.4875	0.7179	0.1967
CV-73	0.6618	0.3617	0.8177	0.5897	0.5075	0.7115	0.1982
CV-74	0.6945	0.4362	0.8287	0.6324	0.5694	0.7389	0.2858
CV-75	0.7091	0.4574	0.8398	0.6486	0.5972	0.7488	0.3207
CV-76	0.6836	0.4043	0.8287	0.6165	0.5507	0.7282	0.2549
CV-77	0.6727	0.4468	0.7901	0.6184	0.5250	0.7333	0.2474
CV-78	0.6691	0.4255	0.7956	0.6106	0.5195	0.7273	0.2336
CV-79	0.7018	0.4681	0.8232	0.6456	0.5789	0.7487	0.3090
CV-80	0.6727	0.3936	0.8177	0.6056	0.5286	0.7220	0.2301
CV-81	0.6836	0.4362	0.8122	0.6242	0.5467	0.7350	0.2645
CV-82	0.6691	0.4255	0.7956	0.6106	0.5195	0.7273	0.2336
CV-83	0.6691	0.4149	0.8011	0.6080	0.5200	0.7250	0.2300
CV-84	0.6800	0.3936	0.8287	0.6112	0.5441	0.7246	0.2445
CV-85	0.6873	0.4362	0.8177	0.6269	0.5541	0.7363	0.2715
CV-86	0.6836	0.4255	0.8177	0.6216	0.5479	0.7327	0.2612
CV-87	0.6909	0.4362	0.8232	0.6297	0.5616	0.7376	0.2786
CV-88	0.6764	0.4255	0.8066	0.6161	0.5333	0.7300	0.2473
CV-89	0.6691	0.4362	0.7901	0.6131	0.5190	0.7296	0.2371
CV-90	0.6764	0.4255	0.8066	0.6161	0.5333	0.7300	0.2473
CV-91	0.6764	0.4149	0.8122	0.6135	0.5342	0.7277	0.2439
CV-92	0.6800	0.3830	0.8343	0.6086	0.5455	0.7225	0.2413
CV-93	0.6945	0.4787	0.8066	0.6427	0.5625	0.7487	0.2980
CV-94	0.6873	0.4255	0.8232	0.6244	0.5556	0.7340	0.2684
CV-95	0.7127	0.5000	0.8232	0.6616	0.5949	0.7602	0.3388
CV-96	0.7018	0.4787	0.8177	0.6482	0.5769	0.7513	0.3119
CV-97	0.6836	0.3936	0.8343	0.6139	0.5522	0.7260	0.2518
CV-98	0.6691	0.3511	0.8343	0.5927	0.5238	0.7123	0.2092
CV-99	0.6909	0.4255	0.8287	0.6271	0.5634	0.7353	0.2756
CV-100	0.6436	0.4043	0.7680	0.5861	0.4750	0.7128	0.1798
CV-101	0.6873	0.4681	0.8011	0.6346	0.5500	0.7436	0.2811
CV-102	0.7164	0.4362	0.8619	0.6490	0.6212	0.7464	0.3310
CV-103	0.7091	0.4681	0.8343	0.6512	0.5946	0.7512	0.3234
CV-104	0.6473	0.3936	0.7790	0.5863	0.4805	0.7121	0.1824
CV-105	0.6691	0.4362	0.7901	0.6131	0.5190	0.7296	0.2371
CV-106	0.6982	0.4255	0.8398	0.6327	0.5797	0.7379	0.2903
CV-107	0.6836	0.4255	0.8177	0.6216	0.5479	0.7327	0.2612

CV-108	0.6800	0.4043	0.8232	0.6137	0.5429	0.7268	0.2477
CV-109	0.6764	0.3936	0.8232	0.6084	0.5362	0.7233	0.2372
CV-110	0.7091	0.4468	0.8453	0.6461	0.6000	0.7463	0.3181
CV-111	0.6800	0.4149	0.8177	0.6163	0.5417	0.7291	0.2509
CV-112	0.6618	0.3936	0.8011	0.5974	0.5068	0.7178	0.2092
CV-113	0.7127	0.4894	0.8287	0.6590	0.5974	0.7576	0.3360
CV-114	0.6691	0.3830	0.8177	0.6003	0.5217	0.7184	0.2195
CV-115	0.6873	0.4574	0.8066	0.6320	0.5513	0.7411	0.2779
CV-116	0.6582	0.3936	0.7956	0.5946	0.5000	0.7164	0.2024
CV-117	0.6945	0.4574	0.8177	0.6376	0.5658	0.7437	0.2918
CV-118	0.6764	0.4043	0.8177	0.6110	0.5352	0.7255	0.2405
CV-119	0.6836	0.5000	0.7790	0.6395	0.5402	0.7500	0.2846
CV-120	0.6582	0.4149	0.7845	0.5997	0.5000	0.7208	0.2098
CV-121	0.6982	0.4681	0.8177	0.6429	0.5714	0.7475	0.3019
CV-122	0.6691	0.4043	0.8066	0.6054	0.5205	0.7228	0.2265
CV-123	0.6400	0.3617	0.7845	0.5731	0.4658	0.7030	0.1571
CV-124	0.6764	0.4255	0.8066	0.6161	0.5333	0.7300	0.2473
CV-125	0.7127	0.4681	0.8398	0.6539	0.6027	0.7525	0.3307
CV-126	0.6509	0.4149	0.7735	0.5942	0.4875	0.7179	0.1967
CV-127	0.6836	0.4149	0.8232	0.6190	0.5493	0.7304	0.2581
CV-128	0.6909	0.4149	0.8343	0.6246	0.5652	0.7330	0.2726
CV-129	0.6873	0.4787	0.7956	0.6372	0.5488	0.7461	0.2844
CV-130	0.6655	0.4149	0.7956	0.6052	0.5132	0.7236	0.2232
CV-131	0.6800	0.4362	0.8066	0.6214	0.5395	0.7337	0.2575
CV-132	0.7018	0.4362	0.8398	0.6380	0.5857	0.7415	0.3005
CV-133	0.6836	0.4681	0.7956	0.6318	0.5432	0.7423	0.2744
CV-134	0.6800	0.4255	0.8122	0.6188	0.5405	0.7313	0.2542
CV-135	0.6945	0.4149	0.8398	0.6273	0.5735	0.7343	0.2800
CV-136	0.6873	0.4043	0.8343	0.6193	0.5588	0.7295	0.2622
CV-137	0.6909	0.4255	0.8287	0.6271	0.5634	0.7353	0.2756
CV-138	0.6727	0.4681	0.7790	0.6235	0.5238	0.7382	0.2545
CV-139	0.6618	0.3723	0.8122	0.5922	0.5072	0.7136	0.2019
CV-140	0.7018	0.4681	0.8232	0.6456	0.5789	0.7487	0.3090
CV-141	0.7055	0.4468	0.8398	0.6433	0.5915	0.7451	0.3106
CV-142	0.6655	0.4468	0.7790	0.6129	0.5122	0.7306	0.2341
CV-143	0.6836	0.4468	0.8066	0.6267	0.5455	0.7374	0.2677
CV-144	0.6727	0.4043	0.8122	0.6082	0.5278	0.7241	0.2335
CV-145	0.6764	0.4043	0.8177	0.6110	0.5352	0.7255	0.2405
CV-146	0.6764	0.3936	0.8232	0.6084	0.5362	0.7233	0.2372
CV-147	0.6509	0.3511	0.8066	0.5788	0.4853	0.7053	0.1734
CV-148	0.6800	0.4255	0.8122	0.6188	0.5405	0.7313	0.2542
CV-149	0.6836	0.4255	0.8177	0.6216	0.5479	0.7327	0.2612
CV-150	0.6909	0.4468	0.8177	0.6322	0.5600	0.7400	0.2817
CV-151	0.6764	0.4043	0.8177	0.6110	0.5352	0.7255	0.2405
CV-152	0.6836	0.4787	0.7901	0.6344	0.5422	0.7448	0.2777
CV-153	0.6618	0.4149	0.7901	0.6025	0.5065	0.7222	0.2165
CV-154	0.7055	0.4681	0.8287	0.6484	0.5867	0.7500	0.3161
CV-155	0.6836	0.4043	0.8287	0.6165	0.5507	0.7282	0.2549
CV-156	0.6727	0.4681	0.7790	0.6235	0.5238	0.7382	0.2545
CV-157	0.7091	0.5000	0.8177	0.6588	0.5875	0.7590	0.3318
CV-158	0.6545	0.4149	0.7790	0.5969	0.4937	0.7194	0.2033
CV-159	0.6436	0.4043	0.7680	0.5861	0.4750	0.7128	0.1798
CV-160	0.7018	0.4255	0.8453	0.6354	0.5882	0.7391	0.2978
CV-161	0.6618	0.4149	0.7901	0.6025	0.5065	0.7222	0.2165
CV-162	0.6727	0.4574	0.7845	0.6210	0.5244	0.7358	0.2509
CV-163	0.6545	0.3936	0.7901	0.5918	0.4933	0.7150	0.1956
CV-164	0.6764	0.4255	0.8066	0.6161	0.5333	0.7300	0.2473

CV-165	0.6400	0.4149	0.7569	0.5859	0.4699	0.7135	0.1775
CV-166	0.6764	0.4894	0.7735	0.6314	0.5287	0.7447	0.2681
CV-167	0.6509	0.3830	0.7901	0.5865	0.4865	0.7114	0.1851
CV-168	0.7055	0.4362	0.8453	0.6407	0.5942	0.7427	0.3080
CV-169	0.6909	0.4468	0.8177	0.6322	0.5600	0.7400	0.2817
CV-170	0.6909	0.4468	0.8177	0.6322	0.5600	0.7400	0.2817
CV-171	0.6873	0.4362	0.8177	0.6269	0.5541	0.7363	0.2715
CV-172	0.6800	0.4149	0.8177	0.6163	0.5417	0.7291	0.2509
CV-173	0.6836	0.4043	0.8287	0.6165	0.5507	0.7282	0.2549
CV-174	0.6764	0.4468	0.7956	0.6212	0.5316	0.7347	0.2541
CV-175	0.6691	0.4149	0.8011	0.6080	0.5200	0.7250	0.2300
CV-176	0.7164	0.4574	0.8508	0.6541	0.6143	0.7512	0.3357
CV-177	0.6873	0.4681	0.8011	0.6346	0.5500	0.7436	0.2811
CV-178	0.7018	0.4574	0.8287	0.6431	0.5811	0.7463	0.3061
CV-179	0.6836	0.4681	0.7956	0.6318	0.5432	0.7423	0.2744
CV-180	0.6727	0.4149	0.8066	0.6108	0.5270	0.7264	0.2369
CV-181	0.6691	0.4149	0.8011	0.6080	0.5200	0.7250	0.2300
CV-182	0.6945	0.4468	0.8232	0.6350	0.5676	0.7413	0.2888
CV-183	0.6764	0.4149	0.8122	0.6135	0.5342	0.7277	0.2439
CV-184	0.6800	0.4468	0.8011	0.6240	0.5385	0.7360	0.2609
CV-185	0.6582	0.4255	0.7790	0.6023	0.5000	0.7231	0.2136
CV-186	0.6764	0.3936	0.8232	0.6084	0.5362	0.7233	0.2372
CV-187	0.6618	0.4362	0.7790	0.6076	0.5062	0.7268	0.2239
CV-188	0.6982	0.4681	0.8177	0.6429	0.5714	0.7475	0.3019
CV-189	0.6764	0.3936	0.8232	0.6084	0.5362	0.7233	0.2372
CV-190	0.6436	0.4362	0.7514	0.5938	0.4767	0.7196	0.1919
CV-191	0.6691	0.4043	0.8066	0.6054	0.5205	0.7228	0.2265
CV-192	0.6800	0.4255	0.8122	0.6188	0.5405	0.7313	0.2542
CV-193	0.7055	0.4787	0.8232	0.6510	0.5844	0.7525	0.3190
CV-194	0.6836	0.4681	0.7956	0.6318	0.5432	0.7423	0.2744
CV-195	0.6691	0.4149	0.8011	0.6080	0.5200	0.7250	0.2300
CV-196	0.7164	0.4787	0.8398	0.6593	0.6081	0.7562	0.3406
CV-197	0.6691	0.4255	0.7956	0.6106	0.5195	0.7273	0.2336
CV-198	0.7055	0.4574	0.8343	0.6459	0.5890	0.7475	0.3133
CV-199	0.6764	0.4255	0.8066	0.6161	0.5333	0.7300	0.2473
CV-200	0.6764	0.4468	0.7956	0.6212	0.5316	0.7347	0.2541
CV-201	0.7018	0.4468	0.8343	0.6405	0.5833	0.7438	0.3032
CV-202	0.6909	0.4468	0.8177	0.6322	0.5600	0.7400	0.2817
CV-203	0.6545	0.3830	0.7956	0.5893	0.4932	0.7129	0.1918
CV-204	0.6764	0.4894	0.7735	0.6314	0.5287	0.7447	0.2681
CV-205	0.6618	0.4468	0.7735	0.6101	0.5060	0.7292	0.2276
CV-206	0.7164	0.4574	0.8508	0.6541	0.6143	0.7512	0.3357
CV-207	0.6836	0.4255	0.8177	0.6216	0.5479	0.7327	0.2612
CV-208	0.6655	0.4043	0.8011	0.6027	0.5135	0.7214	0.2196
CV-209	0.6727	0.4043	0.8122	0.6082	0.5278	0.7241	0.2335
CV-210	0.6727	0.4574	0.7845	0.6210	0.5244	0.7358	0.2509
CV-211	0.6618	0.3936	0.8011	0.5974	0.5068	0.7178	0.2092
CV-212	0.7018	0.4362	0.8398	0.6380	0.5857	0.7415	0.3005
CV-213	0.6873	0.4255	0.8232	0.6244	0.5556	0.7340	0.2684
CV-214	0.6945	0.4787	0.8066	0.6427	0.5625	0.7487	0.2980
CV-215	0.6873	0.4681	0.8011	0.6346	0.5500	0.7436	0.2811
CV-216	0.6727	0.4255	0.8011	0.6133	0.5263	0.7286	0.2404
CV-217	0.6764	0.4362	0.8011	0.6186	0.5325	0.7323	0.2507
CV-218	0.6727	0.4362	0.7956	0.6159	0.5256	0.7310	0.2439
CV-219	0.6909	0.4255	0.8287	0.6271	0.5634	0.7353	0.2756
CV-220	0.6655	0.4043	0.8011	0.6027	0.5135	0.7214	0.2196
CV-221	0.6800	0.4681	0.7901	0.6291	0.5366	0.7409	0.2677

CV-222	0.6945	0.4681	0.8122	0.6401	0.5641	0.7462	0.2949
CV-223	0.6873	0.4043	0.8343	0.6193	0.5588	0.7295	0.2622
CV-224	0.7018	0.4468	0.8343	0.6405	0.5833	0.7438	0.3032
CV-225	0.6582	0.4574	0.7624	0.6099	0.5000	0.7302	0.2250
CV-226	0.6909	0.4362	0.8232	0.6297	0.5616	0.7376	0.2786
CV-227	0.6655	0.4255	0.7901	0.6078	0.5128	0.7259	0.2269
CV-228	0.6509	0.3936	0.7845	0.5891	0.4868	0.7136	0.1890
CV-229	0.6873	0.4574	0.8066	0.6320	0.5513	0.7411	0.2779
CV-230	0.6800	0.4149	0.8177	0.6163	0.5417	0.7291	0.2509
CV-231	0.6836	0.4255	0.8177	0.6216	0.5479	0.7327	0.2612
CV-232	0.6764	0.4574	0.7901	0.6238	0.5309	0.7371	0.2575
CV-233	0.6982	0.4255	0.8398	0.6327	0.5797	0.7379	0.2903
CV-234	0.6945	0.4468	0.8232	0.6350	0.5676	0.7413	0.2888
CV-235	0.6982	0.4574	0.8232	0.6403	0.5733	0.7450	0.2989
CV-236	0.6764	0.4043	0.8177	0.6110	0.5352	0.7255	0.2405
CV-237	0.6655	0.4149	0.7956	0.6052	0.5132	0.7236	0.2232
CV-238	0.6691	0.4255	0.7956	0.6106	0.5195	0.7273	0.2336
CV-239	0.6909	0.4787	0.8011	0.6399	0.5556	0.7474	0.2912
CV-240	0.6691	0.4255	0.7956	0.6106	0.5195	0.7273	0.2336
CV-241	0.6691	0.4255	0.7956	0.6106	0.5195	0.7273	0.2336
CV-242	0.6836	0.4362	0.8122	0.6242	0.5467	0.7350	0.2645
CV-243	0.6618	0.4043	0.7956	0.5999	0.5067	0.7200	0.2128
CV-244	0.6764	0.4362	0.8011	0.6186	0.5325	0.7323	0.2507
CV-245	0.6800	0.4149	0.8177	0.6163	0.5417	0.7291	0.2509
CV-246	0.6836	0.4787	0.7901	0.6344	0.5422	0.7448	0.2777
CV-247	0.6836	0.4255	0.8177	0.6216	0.5479	0.7327	0.2612
CV-248	0.6836	0.4362	0.8122	0.6242	0.5467	0.7350	0.2645
CV-249	0.7018	0.4574	0.8287	0.6431	0.5811	0.7463	0.3061
CV-250	0.6836	0.4468	0.8066	0.6267	0.5455	0.7374	0.2677
CV-251	0.6800	0.4255	0.8122	0.6188	0.5405	0.7313	0.2542
CV-252	0.6473	0.3830	0.7845	0.5838	0.4800	0.7100	0.1784
CV-253	0.6727	0.4149	0.8066	0.6108	0.5270	0.7264	0.2369
CV-254	0.7055	0.3936	0.8674	0.6305	0.6066	0.7336	0.2980
CV-255	0.7055	0.4468	0.8398	0.6433	0.5915	0.7451	0.3106
CV-256	0.6836	0.4362	0.8122	0.6242	0.5467	0.7350	0.2645
CV-257	0.6909	0.4043	0.8398	0.6220	0.5672	0.7308	0.2696
CV-258	0.6764	0.3936	0.8232	0.6084	0.5362	0.7233	0.2372
CV-259	0.6873	0.4468	0.8122	0.6295	0.5526	0.7387	0.2747
CV-260	0.6727	0.3830	0.8232	0.6031	0.5294	0.7198	0.2267
CV-261	0.6800	0.3723	0.8398	0.6061	0.5469	0.7204	0.2381
CV-262	0.6836	0.4149	0.8232	0.6190	0.5493	0.7304	0.2581
CV-263	0.6509	0.3936	0.7845	0.5891	0.4868	0.7136	0.1890
CV-264	0.6909	0.4468	0.8177	0.6322	0.5600	0.7400	0.2817
CV-265	0.6473	0.3723	0.7901	0.5812	0.4795	0.7079	0.1744
CV-266	0.6836	0.4255	0.8177	0.6216	0.5479	0.7327	0.2612
CV-267	0.6909	0.4255	0.8287	0.6271	0.5634	0.7353	0.2756
CV-268	0.6945	0.4574	0.8177	0.6376	0.5658	0.7437	0.2918
CV-269	0.6800	0.4255	0.8122	0.6188	0.5405	0.7313	0.2542
CV-270	0.6836	0.4362	0.8122	0.6242	0.5467	0.7350	0.2645
CV-271	0.6655	0.3191	0.8453	0.5822	0.5172	0.7051	0.1912
CV-272	0.6800	0.4468	0.8011	0.6240	0.5385	0.7360	0.2609
CV-273	0.6873	0.4255	0.8232	0.6244	0.5556	0.7340	0.2684
CV-274	0.6873	0.4787	0.7956	0.6372	0.5488	0.7461	0.2844
CV-275	0.7091	0.4681	0.8343	0.6512	0.5946	0.7512	0.3234
CV-276	0.6727	0.4043	0.8122	0.6082	0.5278	0.7241	0.2335
CV-277	0.6909	0.4255	0.8287	0.6271	0.5634	0.7353	0.2756
CV-278	0.6800	0.4255	0.8122	0.6188	0.5405	0.7313	0.2542

CV-279	0.6473	0.4255	0.7624	0.5940	0.4819	0.7188	0.1942
CV-280	0.6873	0.4468	0.8122	0.6295	0.5526	0.7387	0.2747
CV-281	0.6800	0.4255	0.8122	0.6188	0.5405	0.7313	0.2542
CV-282	0.6836	0.4468	0.8066	0.6267	0.5455	0.7374	0.2677
CV-283	0.6800	0.4149	0.8177	0.6163	0.5417	0.7291	0.2509
CV-284	0.6727	0.3936	0.8177	0.6056	0.5286	0.7220	0.2301
CV-285	0.6545	0.4149	0.7790	0.5969	0.4937	0.7194	0.2033
CV-286	0.6982	0.4468	0.8287	0.6378	0.5753	0.7426	0.2960
CV-287	0.6800	0.3617	0.8453	0.6035	0.5484	0.7183	0.2350
CV-288	0.6836	0.4468	0.8066	0.6267	0.5455	0.7374	0.2677
CV-289	0.6800	0.4574	0.7956	0.6265	0.5375	0.7385	0.2642
CV-290	0.6982	0.4468	0.8287	0.6378	0.5753	0.7426	0.2960
CV-291	0.6873	0.4574	0.8066	0.6320	0.5513	0.7411	0.2779
CV-292	0.6873	0.4574	0.8066	0.6320	0.5513	0.7411	0.2779
CV-293	0.6982	0.4362	0.8343	0.6352	0.5775	0.7402	0.2931
CV-294	0.6909	0.4468	0.8177	0.6322	0.5600	0.7400	0.2817
CV-295	0.6691	0.4362	0.7901	0.6131	0.5190	0.7296	0.2371
CV-296	0.6800	0.4149	0.8177	0.6163	0.5417	0.7291	0.2509
CV-297	0.6873	0.4574	0.8066	0.6320	0.5513	0.7411	0.2779
CV-298	0.6873	0.4468	0.8122	0.6295	0.5526	0.7387	0.2747
CV-299	0.6764	0.4043	0.8177	0.6110	0.5352	0.7255	0.2405
CV-300	0.6873	0.4255	0.8232	0.6244	0.5556	0.7340	0.2684
CV-301	0.6836	0.4255	0.8177	0.6216	0.5479	0.7327	0.2612
CV-302	0.7055	0.4574	0.8343	0.6459	0.5890	0.7475	0.3133
CV-303	0.6691	0.4043	0.8066	0.6054	0.5205	0.7228	0.2265
CV-304	0.6836	0.4255	0.8177	0.6216	0.5479	0.7327	0.2612
CV-305	0.6836	0.4362	0.8122	0.6242	0.5467	0.7350	0.2645
CV-306	0.6764	0.4255	0.8066	0.6161	0.5333	0.7300	0.2473
CV-307	0.6836	0.4574	0.8011	0.6293	0.5443	0.7398	0.2710
CV-308	0.6836	0.4255	0.8177	0.6216	0.5479	0.7327	0.2612
CV-309	0.6764	0.4574	0.7901	0.6238	0.5309	0.7371	0.2575
CV-310	0.6764	0.3936	0.8232	0.6084	0.5362	0.7233	0.2372
CV-311	0.6836	0.4362	0.8122	0.6242	0.5467	0.7350	0.2645
CV-312	0.6909	0.4681	0.8066	0.6374	0.5570	0.7449	0.2880
CV-313	0.6873	0.4362	0.8177	0.6269	0.5541	0.7363	0.2715
CV-314	0.6618	0.4149	0.7901	0.6025	0.5065	0.7222	0.2165
CV-315	0.6764	0.4681	0.7845	0.6263	0.5301	0.7396	0.2610
CV-316	0.6836	0.4149	0.8232	0.6190	0.5493	0.7304	0.2581
CV-317	0.7236	0.4681	0.8564	0.6622	0.6286	0.7561	0.3533
CV-318	0.6982	0.4362	0.8343	0.6352	0.5775	0.7402	0.2931
CV-319	0.6582	0.3723	0.8066	0.5895	0.5000	0.7122	0.1949
CV-320	0.6873	0.4149	0.8287	0.6218	0.5571	0.7317	0.2653
CV-321	0.6836	0.4681	0.7956	0.6318	0.5432	0.7423	0.2744
CV-322	0.6945	0.4574	0.8177	0.6376	0.5658	0.7437	0.2918
CV-323	0.6982	0.4149	0.8453	0.6301	0.5821	0.7356	0.2875
CV-324	0.7018	0.4574	0.8287	0.6431	0.5811	0.7463	0.3061
CV-325	0.6764	0.4574	0.7901	0.6238	0.5309	0.7371	0.2575
CV-326	0.7091	0.4681	0.8343	0.6512	0.5946	0.7512	0.3234
CV-327	0.6618	0.4468	0.7735	0.6101	0.5060	0.7292	0.2276
CV-328	0.6836	0.3830	0.8398	0.6114	0.5538	0.7238	0.2487
CV-329	0.6982	0.4255	0.8398	0.6327	0.5797	0.7379	0.2903
CV-330	0.6691	0.4468	0.7845	0.6157	0.5185	0.7320	0.2407
CV-331	0.6873	0.3936	0.8398	0.6167	0.5606	0.7273	0.2592
CV-332	0.7345	0.4894	0.8619	0.6756	0.6479	0.7647	0.3807
CV-333	0.7127	0.4362	0.8564	0.6463	0.6119	0.7452	0.3232
CV-334	0.6691	0.4362	0.7901	0.6131	0.5190	0.7296	0.2371
CV-335	0.6800	0.3617	0.8453	0.6035	0.5484	0.7183	0.2350

CV-336	0.6982	0.4468	0.8287	0.6378	0.5753	0.7426	0.2960
CV-337	0.6364	0.4149	0.7514	0.5831	0.4643	0.7120	0.1712
CV-338	0.6873	0.4362	0.8177	0.6269	0.5541	0.7363	0.2715
CV-339	0.7055	0.4574	0.8343	0.6459	0.5890	0.7475	0.3133
CV-340	0.6764	0.4468	0.7956	0.6212	0.5316	0.7347	0.2541
CV-341	0.6618	0.4043	0.7956	0.5999	0.5067	0.7200	0.2128
CV-342	0.6691	0.4681	0.7735	0.6208	0.5176	0.7368	0.2479
CV-343	0.6691	0.4149	0.8011	0.6080	0.5200	0.7250	0.2300
CV-344	0.6727	0.4468	0.7901	0.6184	0.5250	0.7333	0.2474
CV-345	0.6945	0.4468	0.8232	0.6350	0.5676	0.7413	0.2888
CV-346	0.7236	0.4894	0.8453	0.6673	0.6216	0.7612	0.3579
CV-347	0.6909	0.4787	0.8011	0.6399	0.5556	0.7474	0.2912
CV-348	0.6945	0.4362	0.8287	0.6324	0.5694	0.7389	0.2858
CV-349	0.6727	0.4362	0.7956	0.6159	0.5256	0.7310	0.2439
CV-350	0.6727	0.4255	0.8011	0.6133	0.5263	0.7286	0.2404
CV-351	0.6618	0.4043	0.7956	0.5999	0.5067	0.7200	0.2128
CV-352	0.6800	0.3723	0.8398	0.6061	0.5469	0.7204	0.2381
CV-353	0.6945	0.4894	0.8011	0.6452	0.5610	0.7513	0.3012
CV-354	0.6836	0.4574	0.8011	0.6293	0.5443	0.7398	0.2710
CV-355	0.6909	0.4255	0.8287	0.6271	0.5634	0.7353	0.2756
CV-356	0.6909	0.4468	0.8177	0.6322	0.5600	0.7400	0.2817
CV-357	0.6800	0.3723	0.8398	0.6061	0.5469	0.7204	0.2381
CV-358	0.6800	0.4574	0.7956	0.6265	0.5375	0.7385	0.2642
CV-359	0.6691	0.4362	0.7901	0.6131	0.5190	0.7296	0.2371
CV-360	0.6873	0.4255	0.8232	0.6244	0.5556	0.7340	0.2684
CV-361	0.6945	0.3936	0.8508	0.6222	0.5781	0.7299	0.2744
CV-362	0.6764	0.4362	0.8011	0.6186	0.5325	0.7323	0.2507
CV-363	0.6836	0.4468	0.8066	0.6267	0.5455	0.7374	0.2677
CV-364	0.6545	0.4043	0.7845	0.5944	0.4935	0.7172	0.1994
CV-365	0.6691	0.3830	0.8177	0.6003	0.5217	0.7184	0.2195
CV-366	0.7091	0.4468	0.8453	0.6461	0.6000	0.7463	0.3181
CV-367	0.6509	0.3936	0.7845	0.5891	0.4868	0.7136	0.1890
CV-368	0.7091	0.4681	0.8343	0.6512	0.5946	0.7512	0.3234
CV-369	0.6545	0.4468	0.7624	0.6046	0.4941	0.7263	0.2148
CV-370	0.6691	0.4362	0.7901	0.6131	0.5190	0.7296	0.2371
CV-371	0.7127	0.4574	0.8453	0.6514	0.6056	0.7500	0.3281
CV-372	0.7018	0.4787	0.8177	0.6482	0.5769	0.7513	0.3119
CV-373	0.6618	0.3830	0.8066	0.5948	0.5070	0.7157	0.2055
CV-374	0.6873	0.4255	0.8232	0.6244	0.5556	0.7340	0.2684
CV-375	0.6509	0.3830	0.7901	0.5865	0.4865	0.7114	0.1851
CV-376	0.6655	0.4362	0.7845	0.6104	0.5125	0.7282	0.2305
CV-377	0.7055	0.4468	0.8398	0.6433	0.5915	0.7451	0.3106
CV-378	0.6873	0.4681	0.8011	0.6346	0.5500	0.7436	0.2811
CV-379	0.6909	0.4043	0.8398	0.6220	0.5672	0.7308	0.2696
CV-380	0.6800	0.4149	0.8177	0.6163	0.5417	0.7291	0.2509
CV-381	0.6655	0.4149	0.7956	0.6052	0.5132	0.7236	0.2232
CV-382	0.6945	0.4574	0.8177	0.6376	0.5658	0.7437	0.2918
CV-383	0.6800	0.4255	0.8122	0.6188	0.5405	0.7313	0.2542
CV-384	0.6764	0.4149	0.8122	0.6135	0.5342	0.7277	0.2439
CV-385	0.6800	0.4362	0.8066	0.6214	0.5395	0.7337	0.2575
CV-386	0.6909	0.4574	0.8122	0.6348	0.5584	0.7424	0.2848
CV-387	0.6509	0.4255	0.7680	0.5967	0.4878	0.7202	0.2006
CV-388	0.7091	0.4468	0.8453	0.6461	0.6000	0.7463	0.3181
CV-389	0.7164	0.4787	0.8398	0.6593	0.6081	0.7562	0.3406
CV-390	0.7055	0.4468	0.8398	0.6433	0.5915	0.7451	0.3106
CV-391	0.6909	0.4043	0.8398	0.6220	0.5672	0.7308	0.2696
CV-392	0.6727	0.4149	0.8066	0.6108	0.5270	0.7264	0.2369

CV-393	0.6727	0.4468	0.7901	0.6184	0.5250	0.7333	0.2474
CV-394	0.6836	0.4255	0.8177	0.6216	0.5479	0.7327	0.2612
CV-395	0.6691	0.4681	0.7735	0.6208	0.5176	0.7368	0.2479
CV-396	0.6618	0.4255	0.7845	0.6050	0.5063	0.7245	0.2202
CV-397	0.6618	0.4043	0.7956	0.5999	0.5067	0.7200	0.2128
CV-398	0.6945	0.4894	0.8011	0.6452	0.5610	0.7513	0.3012
CV-399	0.7200	0.4468	0.8619	0.6543	0.6269	0.7500	0.3411
CV-400	0.6982	0.4468	0.8287	0.6378	0.5753	0.7426	0.2960
CV-401	0.6909	0.4362	0.8232	0.6297	0.5616	0.7376	0.2786
CV-402	0.6836	0.4574	0.8011	0.6293	0.5443	0.7398	0.2710
CV-403	0.6873	0.4149	0.8287	0.6218	0.5571	0.7317	0.2653
CV-404	0.6800	0.4149	0.8177	0.6163	0.5417	0.7291	0.2509
CV-405	0.6800	0.4468	0.8011	0.6240	0.5385	0.7360	0.2609
CV-406	0.6909	0.4468	0.8177	0.6322	0.5600	0.7400	0.2817
CV-407	0.6727	0.3830	0.8232	0.6031	0.5294	0.7198	0.2267
CV-408	0.6473	0.3617	0.7956	0.5786	0.4789	0.7059	0.1705
CV-409	0.7018	0.4787	0.8177	0.6482	0.5769	0.7513	0.3119
CV-410	0.7091	0.4574	0.8398	0.6486	0.5972	0.7488	0.3207
CV-411	0.6655	0.4362	0.7845	0.6104	0.5125	0.7282	0.2305
CV-412	0.6364	0.4468	0.7348	0.5908	0.4667	0.7189	0.1836
CV-413	0.6764	0.4255	0.8066	0.6161	0.5333	0.7300	0.2473
CV-414	0.6727	0.3830	0.8232	0.6031	0.5294	0.7198	0.2267
CV-415	0.6727	0.4362	0.7956	0.6159	0.5256	0.7310	0.2439
CV-416	0.6909	0.4255	0.8287	0.6271	0.5634	0.7353	0.2756
CV-417	0.6618	0.4149	0.7901	0.6025	0.5065	0.7222	0.2165
CV-418	0.6836	0.4255	0.8177	0.6216	0.5479	0.7327	0.2612
CV-419	0.6691	0.4043	0.8066	0.6054	0.5205	0.7228	0.2265
CV-420	0.6800	0.4149	0.8177	0.6163	0.5417	0.7291	0.2509
CV-421	0.6509	0.4043	0.7790	0.5916	0.4872	0.7157	0.1928
CV-422	0.6691	0.4255	0.7956	0.6106	0.5195	0.7273	0.2336
CV-423	0.6836	0.3830	0.8398	0.6114	0.5538	0.7238	0.2487
CV-424	0.6727	0.3830	0.8232	0.6031	0.5294	0.7198	0.2267
CV-425	0.7055	0.4362	0.8453	0.6407	0.5942	0.7427	0.3080
CV-426	0.6945	0.4043	0.8453	0.6248	0.5758	0.7321	0.2772
CV-427	0.6909	0.4043	0.8398	0.6220	0.5672	0.7308	0.2696
CV-428	0.6509	0.4255	0.7680	0.5967	0.4878	0.7202	0.2006
CV-429	0.7091	0.4681	0.8343	0.6512	0.5946	0.7512	0.3234
CV-430	0.6764	0.3511	0.8453	0.5982	0.5410	0.7150	0.2242
CV-431	0.6691	0.4574	0.7790	0.6182	0.5181	0.7344	0.2443
CV-432	0.6545	0.4149	0.7790	0.5969	0.4937	0.7194	0.2033
CV-433	0.6909	0.4149	0.8343	0.6246	0.5652	0.7330	0.2726
CV-434	0.6764	0.4574	0.7901	0.6238	0.5309	0.7371	0.2575
CV-435	0.6945	0.4043	0.8453	0.6248	0.5758	0.7321	0.2772
CV-436	0.7127	0.4362	0.8564	0.6463	0.6119	0.7452	0.3232
CV-437	0.6836	0.5000	0.7790	0.6395	0.5402	0.7500	0.2846
CV-438	0.6691	0.4362	0.7901	0.6131	0.5190	0.7296	0.2371
CV-439	0.6873	0.4255	0.8232	0.6244	0.5556	0.7340	0.2684
CV-440	0.6873	0.4255	0.8232	0.6244	0.5556	0.7340	0.2684
CV-441	0.6618	0.4681	0.7624	0.6153	0.5057	0.7340	0.2351
CV-442	0.7091	0.4574	0.8398	0.6486	0.5972	0.7488	0.3207
CV-443	0.6655	0.3936	0.8066	0.6001	0.5139	0.7192	0.2161
CV-444	0.6655	0.4255	0.7901	0.6078	0.5128	0.7259	0.2269
CV-445	0.6909	0.4787	0.8011	0.6399	0.5556	0.7474	0.2912
CV-446	0.6873	0.4362	0.8177	0.6269	0.5541	0.7363	0.2715
CV-447	0.6800	0.4681	0.7901	0.6291	0.5366	0.7409	0.2677
CV-448	0.7018	0.4255	0.8453	0.6354	0.5882	0.7391	0.2978
CV-449	0.6727	0.4043	0.8122	0.6082	0.5278	0.7241	0.2335

CV-450	0.6436	0.3723	0.7845	0.5784	0.4730	0.7065	0.1678
CV-451	0.6836	0.4255	0.8177	0.6216	0.5479	0.7327	0.2612
CV-452	0.6473	0.4255	0.7624	0.5940	0.4819	0.7188	0.1942
CV-453	0.6509	0.3936	0.7845	0.5891	0.4868	0.7136	0.1890
CV-454	0.6545	0.3830	0.7956	0.5893	0.4932	0.7129	0.1918
CV-455	0.6691	0.3830	0.8177	0.6003	0.5217	0.7184	0.2195
CV-456	0.6945	0.4362	0.8287	0.6324	0.5694	0.7389	0.2858
CV-457	0.7018	0.4149	0.8508	0.6329	0.5909	0.7368	0.2951
CV-458	0.6764	0.3723	0.8343	0.6033	0.5385	0.7190	0.2307
CV-459	0.6909	0.4362	0.8232	0.6297	0.5616	0.7376	0.2786
CV-460	0.6618	0.3936	0.8011	0.5974	0.5068	0.7178	0.2092
CV-461	0.6982	0.4574	0.8232	0.6403	0.5733	0.7450	0.2989
CV-462	0.6655	0.3617	0.8232	0.5925	0.5152	0.7129	0.2054
CV-463	0.6655	0.4574	0.7735	0.6155	0.5119	0.7330	0.2378
CV-464	0.6582	0.4362	0.7735	0.6048	0.5000	0.7254	0.2174
CV-465	0.7091	0.4681	0.8343	0.6512	0.5946	0.7512	0.3234
CV-466	0.6509	0.4043	0.7790	0.5916	0.4872	0.7157	0.1928
CV-467	0.6545	0.4043	0.7845	0.5944	0.4935	0.7172	0.1994
CV-468	0.6691	0.4362	0.7901	0.6131	0.5190	0.7296	0.2371
CV-469	0.6691	0.3936	0.8122	0.6029	0.5211	0.7206	0.2230
CV-470	0.6836	0.4362	0.8122	0.6242	0.5467	0.7350	0.2645
CV-471	0.6800	0.4574	0.7956	0.6265	0.5375	0.7385	0.2642
CV-472	0.6800	0.4255	0.8122	0.6188	0.5405	0.7313	0.2542
CV-473	0.6800	0.4149	0.8177	0.6163	0.5417	0.7291	0.2509
CV-474	0.6655	0.4574	0.7735	0.6155	0.5119	0.7330	0.2378
CV-475	0.6800	0.4362	0.8066	0.6214	0.5395	0.7337	0.2575
CV-476	0.6836	0.4362	0.8122	0.6242	0.5467	0.7350	0.2645
CV-477	0.6800	0.4362	0.8066	0.6214	0.5395	0.7337	0.2575
CV-478	0.6909	0.4574	0.8122	0.6348	0.5584	0.7424	0.2848
CV-479	0.7091	0.4255	0.8564	0.6409	0.6061	0.7416	0.3131
CV-480	0.7018	0.4255	0.8453	0.6354	0.5882	0.7391	0.2978
CV-481	0.7055	0.4787	0.8232	0.6510	0.5844	0.7525	0.3190
CV-482	0.6400	0.4255	0.7514	0.5885	0.4706	0.7158	0.1816
CV-483	0.6655	0.4574	0.7735	0.6155	0.5119	0.7330	0.2378
CV-484	0.6691	0.4255	0.7956	0.6106	0.5195	0.7273	0.2336
CV-485	0.6618	0.3830	0.8066	0.5948	0.5070	0.7157	0.2055
CV-486	0.6509	0.3617	0.8011	0.5814	0.4857	0.7073	0.1773
CV-487	0.6727	0.4149	0.8066	0.6108	0.5270	0.7264	0.2369
CV-488	0.6800	0.4362	0.8066	0.6214	0.5395	0.7337	0.2575
CV-489	0.6873	0.4681	0.8011	0.6346	0.5500	0.7436	0.2811
CV-490	0.6655	0.4043	0.8011	0.6027	0.5135	0.7214	0.2196
CV-491	0.7055	0.4894	0.8177	0.6535	0.5823	0.7551	0.3219
CV-492	0.7055	0.4362	0.8453	0.6407	0.5942	0.7427	0.3080
CV-493	0.6800	0.4468	0.8011	0.6240	0.5385	0.7360	0.2609
CV-494	0.6655	0.4574	0.7735	0.6155	0.5119	0.7330	0.2378
CV-495	0.7018	0.4468	0.8343	0.6405	0.5833	0.7438	0.3032
CV-496	0.6764	0.4468	0.7956	0.6212	0.5316	0.7347	0.2541
CV-497	0.6909	0.4362	0.8232	0.6297	0.5616	0.7376	0.2786
CV-498	0.6945	0.3830	0.8564	0.6197	0.5806	0.7277	0.2717
CV-499	0.6764	0.4362	0.8011	0.6186	0.5325	0.7323	0.2507
CV-500	0.6982	0.4787	0.8122	0.6454	0.5696	0.7500	0.3049

Table S9. Performance of 5-fold cross-validations from linear discriminant analysis (LDA). Performance of 5-fold cross-validations were evaluated by seven performance metrics. Each row shows the performance of one time 5-fold cross-validation. 5-fold cross-validation was repeated 500 times (CV-1 ~ CV-500).

Cross-validation	Accuracy	Sensitivity	Specificity	Balanced accuracy	Positive prediction rate	Negative prediction rate	Matthews correlation coefficient
CV-1	0.7309	0.4468	0.8785	0.6626	0.6563	0.7536	0.3651
CV-2	0.7127	0.4362	0.8564	0.6463	0.6119	0.7452	0.3232
CV-3	0.7455	0.5106	0.8674	0.6890	0.6667	0.7734	0.4079
CV-4	0.6909	0.4255	0.8287	0.6271	0.5634	0.7353	0.2756
CV-5	0.6982	0.4149	0.8453	0.6301	0.5821	0.7356	0.2875
CV-6	0.7055	0.4255	0.8508	0.6382	0.5970	0.7404	0.3054
CV-7	0.6800	0.3830	0.8343	0.6086	0.5455	0.7225	0.2413
CV-8	0.6873	0.3936	0.8398	0.6167	0.5606	0.7273	0.2592
CV-9	0.7164	0.4362	0.8619	0.6490	0.6212	0.7464	0.3310
CV-10	0.7091	0.4043	0.8674	0.6358	0.6129	0.7371	0.3083
CV-11	0.6945	0.4362	0.8287	0.6324	0.5694	0.7389	0.2858
CV-12	0.6945	0.4043	0.8453	0.6248	0.5758	0.7321	0.2772
CV-13	0.7236	0.4787	0.8508	0.6648	0.6250	0.7586	0.3556
CV-14	0.7091	0.4255	0.8564	0.6409	0.6061	0.7416	0.3131
CV-15	0.7091	0.4468	0.8453	0.6461	0.6000	0.7463	0.3181
CV-16	0.6945	0.4362	0.8287	0.6324	0.5694	0.7389	0.2858
CV-17	0.7200	0.4362	0.8674	0.6518	0.6308	0.7476	0.3389
CV-18	0.6873	0.3830	0.8453	0.6141	0.5625	0.7251	0.2562
CV-19	0.7127	0.4149	0.8674	0.6411	0.6190	0.7406	0.3186
CV-20	0.6909	0.3830	0.8508	0.6169	0.5714	0.7264	0.2639
CV-21	0.6982	0.3936	0.8564	0.6250	0.5873	0.7311	0.2821
CV-22	0.7200	0.4255	0.8729	0.6492	0.6349	0.7453	0.3369
CV-23	0.7018	0.4255	0.8453	0.6354	0.5882	0.7391	0.2978
CV-24	0.7200	0.4468	0.8619	0.6543	0.6269	0.7500	0.3411
CV-25	0.6945	0.4149	0.8398	0.6273	0.5735	0.7343	0.2800
CV-26	0.6982	0.4149	0.8453	0.6301	0.5821	0.7356	0.2875
CV-27	0.7091	0.4362	0.8508	0.6435	0.6029	0.7440	0.3155
CV-28	0.7236	0.4255	0.8785	0.6520	0.6452	0.7465	0.3450
CV-29	0.6982	0.4149	0.8453	0.6301	0.5821	0.7356	0.2875
CV-30	0.7164	0.4468	0.8564	0.6516	0.6176	0.7488	0.3333
CV-31	0.7164	0.4362	0.8619	0.6490	0.6212	0.7464	0.3310
CV-32	0.6909	0.4574	0.8122	0.6348	0.5584	0.7424	0.2848
CV-33	0.7055	0.4255	0.8508	0.6382	0.5970	0.7404	0.3054
CV-34	0.7055	0.4149	0.8564	0.6356	0.6000	0.7381	0.3028
CV-35	0.7127	0.4468	0.8508	0.6488	0.6087	0.7476	0.3256
CV-36	0.7018	0.4255	0.8453	0.6354	0.5882	0.7391	0.2978
CV-37	0.7164	0.4468	0.8564	0.6516	0.6176	0.7488	0.3333
CV-38	0.7055	0.4574	0.8343	0.6459	0.5890	0.7475	0.3133
CV-39	0.6982	0.4149	0.8453	0.6301	0.5821	0.7356	0.2875
CV-40	0.7164	0.4468	0.8564	0.6516	0.6176	0.7488	0.3333
CV-41	0.7091	0.4149	0.8619	0.6384	0.6094	0.7393	0.3107
CV-42	0.6982	0.4255	0.8398	0.6327	0.5797	0.7379	0.2903
CV-43	0.7200	0.4574	0.8564	0.6569	0.6232	0.7524	0.3433
CV-44	0.7018	0.4149	0.8508	0.6329	0.5909	0.7368	0.2951
CV-45	0.6982	0.4043	0.8508	0.6275	0.5846	0.7333	0.2848
CV-46	0.7127	0.4468	0.8508	0.6488	0.6087	0.7476	0.3256
CV-47	0.7200	0.4468	0.8619	0.6543	0.6269	0.7500	0.3411
CV-48	0.6982	0.4149	0.8453	0.6301	0.5821	0.7356	0.2875
CV-49	0.6945	0.4149	0.8398	0.6273	0.5735	0.7343	0.2800
CV-50	0.7200	0.4468	0.8619	0.6543	0.6269	0.7500	0.3411

CV-51	0.7164	0.4681	0.8453	0.6567	0.6111	0.7537	0.3381
CV-52	0.6909	0.3936	0.8453	0.6195	0.5692	0.7286	0.2667
CV-53	0.7273	0.4574	0.8674	0.6624	0.6418	0.7548	0.3589
CV-54	0.7018	0.4468	0.8343	0.6405	0.5833	0.7438	0.3032
CV-55	0.7091	0.4149	0.8619	0.6384	0.6094	0.7393	0.3107
CV-56	0.7055	0.4468	0.8398	0.6433	0.5915	0.7451	0.3106
CV-57	0.7200	0.4362	0.8674	0.6518	0.6308	0.7476	0.3389
CV-58	0.7127	0.4574	0.8453	0.6514	0.6056	0.7500	0.3281
CV-59	0.7236	0.4574	0.8619	0.6597	0.6324	0.7536	0.3511
CV-60	0.6982	0.4255	0.8398	0.6327	0.5797	0.7379	0.2903
CV-61	0.6982	0.4043	0.8508	0.6275	0.5846	0.7333	0.2848
CV-62	0.6982	0.4043	0.8508	0.6275	0.5846	0.7333	0.2848
CV-63	0.6982	0.4468	0.8287	0.6378	0.5753	0.7426	0.2960
CV-64	0.7273	0.4574	0.8674	0.6624	0.6418	0.7548	0.3589
CV-65	0.6945	0.4043	0.8453	0.6248	0.5758	0.7321	0.2772
CV-66	0.6982	0.3936	0.8564	0.6250	0.5873	0.7311	0.2821
CV-67	0.7055	0.4043	0.8619	0.6331	0.6032	0.7358	0.3004
CV-68	0.7200	0.4468	0.8619	0.6543	0.6269	0.7500	0.3411
CV-69	0.6945	0.4149	0.8398	0.6273	0.5735	0.7343	0.2800
CV-70	0.7018	0.3936	0.8619	0.6277	0.5968	0.7324	0.2900
CV-71	0.7018	0.4043	0.8564	0.6303	0.5938	0.7346	0.2925
CV-72	0.7055	0.4043	0.8619	0.6331	0.6032	0.7358	0.3004
CV-73	0.6873	0.4149	0.8287	0.6218	0.5571	0.7317	0.2653
CV-74	0.6945	0.4149	0.8398	0.6273	0.5735	0.7343	0.2800
CV-75	0.7018	0.4255	0.8453	0.6354	0.5882	0.7391	0.2978
CV-76	0.7018	0.4255	0.8453	0.6354	0.5882	0.7391	0.2978
CV-77	0.7018	0.4043	0.8564	0.6303	0.5938	0.7346	0.2925
CV-78	0.7127	0.4468	0.8508	0.6488	0.6087	0.7476	0.3256
CV-79	0.7018	0.4255	0.8453	0.6354	0.5882	0.7391	0.2978
CV-80	0.6873	0.3936	0.8398	0.6167	0.5606	0.7273	0.2592
CV-81	0.7164	0.4574	0.8508	0.6541	0.6143	0.7512	0.3357
CV-82	0.7018	0.4043	0.8564	0.6303	0.5938	0.7346	0.2925
CV-83	0.7018	0.4255	0.8453	0.6354	0.5882	0.7391	0.2978
CV-84	0.6800	0.3723	0.8398	0.6061	0.5469	0.7204	0.2381
CV-85	0.7018	0.4149	0.8508	0.6329	0.5909	0.7368	0.2951
CV-86	0.6945	0.4255	0.8343	0.6299	0.5714	0.7366	0.2829
CV-87	0.7018	0.4149	0.8508	0.6329	0.5909	0.7368	0.2951
CV-88	0.6982	0.4255	0.8398	0.6327	0.5797	0.7379	0.2903
CV-89	0.6509	0.4043	0.7790	0.5916	0.4872	0.7157	0.1928
CV-90	0.6982	0.4362	0.8343	0.6352	0.5775	0.7402	0.2931
CV-91	0.7055	0.4149	0.8564	0.6356	0.6000	0.7381	0.3028
CV-92	0.6982	0.4255	0.8398	0.6327	0.5797	0.7379	0.2903
CV-93	0.6909	0.4255	0.8287	0.6271	0.5634	0.7353	0.2756
CV-94	0.7164	0.4043	0.8785	0.6414	0.6333	0.7395	0.3247
CV-95	0.7091	0.4149	0.8619	0.6384	0.6094	0.7393	0.3107
CV-96	0.7055	0.4362	0.8453	0.6407	0.5942	0.7427	0.3080
CV-97	0.6945	0.4149	0.8398	0.6273	0.5735	0.7343	0.2800
CV-98	0.7091	0.4362	0.8508	0.6435	0.6029	0.7440	0.3155
CV-99	0.7018	0.4255	0.8453	0.6354	0.5882	0.7391	0.2978
CV-100	0.6945	0.4255	0.8343	0.6299	0.5714	0.7366	0.2829
CV-101	0.6873	0.4255	0.8232	0.6244	0.5556	0.7340	0.2684
CV-102	0.7091	0.4574	0.8398	0.6486	0.5972	0.7488	0.3207
CV-103	0.7164	0.4362	0.8619	0.6490	0.6212	0.7464	0.3310
CV-104	0.6982	0.3830	0.8619	0.6224	0.5902	0.7290	0.2795
CV-105	0.6873	0.4574	0.8066	0.6320	0.5513	0.7411	0.2779
CV-106	0.7236	0.4362	0.8729	0.6545	0.6406	0.7488	0.3470
CV-107	0.7091	0.4255	0.8564	0.6409	0.6061	0.7416	0.3131

CV-108	0.7127	0.4255	0.8619	0.6437	0.6154	0.7429	0.3209
CV-109	0.6909	0.4043	0.8398	0.6220	0.5672	0.7308	0.2696
CV-110	0.7091	0.4362	0.8508	0.6435	0.6029	0.7440	0.3155
CV-111	0.6945	0.4255	0.8343	0.6299	0.5714	0.7366	0.2829
CV-112	0.7055	0.4043	0.8619	0.6331	0.6032	0.7358	0.3004
CV-113	0.7091	0.4468	0.8453	0.6461	0.6000	0.7463	0.3181
CV-114	0.7091	0.4362	0.8508	0.6435	0.6029	0.7440	0.3155
CV-115	0.6945	0.4255	0.8343	0.6299	0.5714	0.7366	0.2829
CV-116	0.6982	0.4149	0.8453	0.6301	0.5821	0.7356	0.2875
CV-117	0.7091	0.4362	0.8508	0.6435	0.6029	0.7440	0.3155
CV-118	0.6800	0.3936	0.8287	0.6112	0.5441	0.7246	0.2445
CV-119	0.6800	0.4255	0.8122	0.6188	0.5405	0.7313	0.2542
CV-120	0.7055	0.4362	0.8453	0.6407	0.5942	0.7427	0.3080
CV-121	0.7091	0.4362	0.8508	0.6435	0.6029	0.7440	0.3155
CV-122	0.6945	0.4362	0.8287	0.6324	0.5694	0.7389	0.2858
CV-123	0.7236	0.4574	0.8619	0.6597	0.6324	0.7536	0.3511
CV-124	0.7018	0.4255	0.8453	0.6354	0.5882	0.7391	0.2978
CV-125	0.7127	0.4468	0.8508	0.6488	0.6087	0.7476	0.3256
CV-126	0.6873	0.4149	0.8287	0.6218	0.5571	0.7317	0.2653
CV-127	0.7055	0.4255	0.8508	0.6382	0.5970	0.7404	0.3054
CV-128	0.7273	0.4574	0.8674	0.6624	0.6418	0.7548	0.3589
CV-129	0.6764	0.4043	0.8177	0.6110	0.5352	0.7255	0.2405
CV-130	0.6909	0.4255	0.8287	0.6271	0.5634	0.7353	0.2756
CV-131	0.7091	0.4468	0.8453	0.6461	0.6000	0.7463	0.3181
CV-132	0.7127	0.4149	0.8674	0.6411	0.6190	0.7406	0.3186
CV-133	0.6982	0.3936	0.8564	0.6250	0.5873	0.7311	0.2821
CV-134	0.7091	0.4255	0.8564	0.6409	0.6061	0.7416	0.3131
CV-135	0.7236	0.4468	0.8674	0.6571	0.6364	0.7512	0.3490
CV-136	0.7091	0.4468	0.8453	0.6461	0.6000	0.7463	0.3181
CV-137	0.7018	0.3936	0.8619	0.6277	0.5968	0.7324	0.2900
CV-138	0.7018	0.4149	0.8508	0.6329	0.5909	0.7368	0.2951
CV-139	0.6945	0.3830	0.8564	0.6197	0.5806	0.7277	0.2717
CV-140	0.7055	0.3936	0.8674	0.6305	0.6066	0.7336	0.2980
CV-141	0.7127	0.4149	0.8674	0.6411	0.6190	0.7406	0.3186
CV-142	0.6982	0.4149	0.8453	0.6301	0.5821	0.7356	0.2875
CV-143	0.6982	0.3936	0.8564	0.6250	0.5873	0.7311	0.2821
CV-144	0.6945	0.4255	0.8343	0.6299	0.5714	0.7366	0.2829
CV-145	0.7018	0.3936	0.8619	0.6277	0.5968	0.7324	0.2900
CV-146	0.6982	0.3936	0.8564	0.6250	0.5873	0.7311	0.2821
CV-147	0.7018	0.4149	0.8508	0.6329	0.5909	0.7368	0.2951
CV-148	0.6945	0.3936	0.8508	0.6222	0.5781	0.7299	0.2744
CV-149	0.6836	0.3936	0.8343	0.6139	0.5522	0.7260	0.2518
CV-150	0.6873	0.4149	0.8287	0.6218	0.5571	0.7317	0.2653
CV-151	0.7164	0.4468	0.8564	0.6516	0.6176	0.7488	0.3333
CV-152	0.7055	0.4468	0.8398	0.6433	0.5915	0.7451	0.3106
CV-153	0.7236	0.4362	0.8729	0.6545	0.6406	0.7488	0.3470
CV-154	0.6909	0.4362	0.8232	0.6297	0.5616	0.7376	0.2786
CV-155	0.7055	0.4149	0.8564	0.6356	0.6000	0.7381	0.3028
CV-156	0.6836	0.4362	0.8122	0.6242	0.5467	0.7350	0.2645
CV-157	0.7091	0.4468	0.8453	0.6461	0.6000	0.7463	0.3181
CV-158	0.7236	0.4468	0.8674	0.6571	0.6364	0.7512	0.3490
CV-159	0.6909	0.3936	0.8453	0.6195	0.5692	0.7286	0.2667
CV-160	0.7091	0.4149	0.8619	0.6384	0.6094	0.7393	0.3107
CV-161	0.7091	0.4362	0.8508	0.6435	0.6029	0.7440	0.3155
CV-162	0.7055	0.4043	0.8619	0.6331	0.6032	0.7358	0.3004
CV-163	0.6909	0.3830	0.8508	0.6169	0.5714	0.7264	0.2639
CV-164	0.7018	0.4149	0.8508	0.6329	0.5909	0.7368	0.2951

CV-165	0.6836	0.4043	0.8287	0.6165	0.5507	0.7282	0.2549
CV-166	0.7127	0.4681	0.8398	0.6539	0.6027	0.7525	0.3307
CV-167	0.6727	0.3936	0.8177	0.6056	0.5286	0.7220	0.2301
CV-168	0.7091	0.4149	0.8619	0.6384	0.6094	0.7393	0.3107
CV-169	0.6909	0.4043	0.8398	0.6220	0.5672	0.7308	0.2696
CV-170	0.6800	0.3936	0.8287	0.6112	0.5441	0.7246	0.2445
CV-171	0.6982	0.4255	0.8398	0.6327	0.5797	0.7379	0.2903
CV-172	0.6909	0.4362	0.8232	0.6297	0.5616	0.7376	0.2786
CV-173	0.7164	0.4681	0.8453	0.6567	0.6111	0.7537	0.3381
CV-174	0.7309	0.4681	0.8674	0.6677	0.6471	0.7585	0.3688
CV-175	0.7127	0.4255	0.8619	0.6437	0.6154	0.7429	0.3209
CV-176	0.7018	0.4362	0.8398	0.6380	0.5857	0.7415	0.3005
CV-177	0.7018	0.4362	0.8398	0.6380	0.5857	0.7415	0.3005
CV-178	0.7055	0.4149	0.8564	0.6356	0.6000	0.7381	0.3028
CV-179	0.7091	0.4255	0.8564	0.6409	0.6061	0.7416	0.3131
CV-180	0.6873	0.4149	0.8287	0.6218	0.5571	0.7317	0.2653
CV-181	0.6727	0.4149	0.8066	0.6108	0.5270	0.7264	0.2369
CV-182	0.7127	0.4574	0.8453	0.6514	0.6056	0.7500	0.3281
CV-183	0.7018	0.4255	0.8453	0.6354	0.5882	0.7391	0.2978
CV-184	0.7127	0.4255	0.8619	0.6437	0.6154	0.7429	0.3209
CV-185	0.7018	0.4043	0.8564	0.6303	0.5938	0.7346	0.2925
CV-186	0.6945	0.4149	0.8398	0.6273	0.5735	0.7343	0.2800
CV-187	0.7164	0.4255	0.8674	0.6465	0.6250	0.7441	0.3288
CV-188	0.6945	0.3936	0.8508	0.6222	0.5781	0.7299	0.2744
CV-189	0.6982	0.3936	0.8564	0.6250	0.5873	0.7311	0.2821
CV-190	0.7200	0.4255	0.8729	0.6492	0.6349	0.7453	0.3369
CV-191	0.7018	0.3936	0.8619	0.6277	0.5968	0.7324	0.2900
CV-192	0.6727	0.3723	0.8287	0.6005	0.5303	0.7177	0.2233
CV-193	0.6873	0.4043	0.8343	0.6193	0.5588	0.7295	0.2622
CV-194	0.6945	0.4043	0.8453	0.6248	0.5758	0.7321	0.2772
CV-195	0.6873	0.4149	0.8287	0.6218	0.5571	0.7317	0.2653
CV-196	0.6909	0.4149	0.8343	0.6246	0.5652	0.7330	0.2726
CV-197	0.7200	0.4574	0.8564	0.6569	0.6232	0.7524	0.3433
CV-198	0.7018	0.4255	0.8453	0.6354	0.5882	0.7391	0.2978
CV-199	0.6909	0.3936	0.8453	0.6195	0.5692	0.7286	0.2667
CV-200	0.6836	0.4255	0.8177	0.6216	0.5479	0.7327	0.2612
CV-201	0.7055	0.4043	0.8619	0.6331	0.6032	0.7358	0.3004
CV-202	0.6873	0.4043	0.8343	0.6193	0.5588	0.7295	0.2622
CV-203	0.7018	0.4149	0.8508	0.6329	0.5909	0.7368	0.2951
CV-204	0.6982	0.4362	0.8343	0.6352	0.5775	0.7402	0.2931
CV-205	0.7127	0.4255	0.8619	0.6437	0.6154	0.7429	0.3209
CV-206	0.7127	0.4468	0.8508	0.6488	0.6087	0.7476	0.3256
CV-207	0.6945	0.4255	0.8343	0.6299	0.5714	0.7366	0.2829
CV-208	0.7055	0.4255	0.8508	0.6382	0.5970	0.7404	0.3054
CV-209	0.6982	0.4362	0.8343	0.6352	0.5775	0.7402	0.2931
CV-210	0.7055	0.4574	0.8343	0.6459	0.5890	0.7475	0.3133
CV-211	0.7018	0.4149	0.8508	0.6329	0.5909	0.7368	0.2951
CV-212	0.7055	0.4255	0.8508	0.6382	0.5970	0.7404	0.3054
CV-213	0.7055	0.4149	0.8564	0.6356	0.6000	0.7381	0.3028
CV-214	0.7309	0.4255	0.8895	0.6575	0.6667	0.7488	0.3618
CV-215	0.6836	0.4149	0.8232	0.6190	0.5493	0.7304	0.2581
CV-216	0.6982	0.4255	0.8398	0.6327	0.5797	0.7379	0.2903
CV-217	0.6945	0.4362	0.8287	0.6324	0.5694	0.7389	0.2858
CV-218	0.7164	0.4574	0.8508	0.6541	0.6143	0.7512	0.3357
CV-219	0.6945	0.4255	0.8343	0.6299	0.5714	0.7366	0.2829
CV-220	0.7091	0.4574	0.8398	0.6486	0.5972	0.7488	0.3207
CV-221	0.7309	0.4574	0.8729	0.6652	0.6515	0.7560	0.3669

CV-222	0.6945	0.4255	0.8343	0.6299	0.5714	0.7366	0.2829
CV-223	0.7018	0.4149	0.8508	0.6329	0.5909	0.7368	0.2951
CV-224	0.7200	0.4468	0.8619	0.6543	0.6269	0.7500	0.3411
CV-225	0.6618	0.4149	0.7901	0.6025	0.5065	0.7222	0.2165
CV-226	0.7055	0.4362	0.8453	0.6407	0.5942	0.7427	0.3080
CV-227	0.6945	0.4043	0.8453	0.6248	0.5758	0.7321	0.2772
CV-228	0.6945	0.4468	0.8232	0.6350	0.5676	0.7413	0.2888
CV-229	0.7273	0.4574	0.8674	0.6624	0.6418	0.7548	0.3589
CV-230	0.7091	0.4255	0.8564	0.6409	0.6061	0.7416	0.3131
CV-231	0.7018	0.4255	0.8453	0.6354	0.5882	0.7391	0.2978
CV-232	0.6800	0.4149	0.8177	0.6163	0.5417	0.7291	0.2509
CV-233	0.7055	0.4362	0.8453	0.6407	0.5942	0.7427	0.3080
CV-234	0.7164	0.4574	0.8508	0.6541	0.6143	0.7512	0.3357
CV-235	0.7309	0.4787	0.8619	0.6703	0.6429	0.7610	0.3709
CV-236	0.7273	0.4574	0.8674	0.6624	0.6418	0.7548	0.3589
CV-237	0.6873	0.4043	0.8343	0.6193	0.5588	0.7295	0.2622
CV-238	0.7164	0.4362	0.8619	0.6490	0.6212	0.7464	0.3310
CV-239	0.7055	0.4149	0.8564	0.6356	0.6000	0.7381	0.3028
CV-240	0.6873	0.4043	0.8343	0.6193	0.5588	0.7295	0.2622
CV-241	0.7091	0.4255	0.8564	0.6409	0.6061	0.7416	0.3131
CV-242	0.6982	0.3936	0.8564	0.6250	0.5873	0.7311	0.2821
CV-243	0.6945	0.4362	0.8287	0.6324	0.5694	0.7389	0.2858
CV-244	0.6873	0.4149	0.8287	0.6218	0.5571	0.7317	0.2653
CV-245	0.7018	0.4255	0.8453	0.6354	0.5882	0.7391	0.2978
CV-246	0.6945	0.4681	0.8122	0.6401	0.5641	0.7462	0.2949
CV-247	0.7055	0.4149	0.8564	0.6356	0.6000	0.7381	0.3028
CV-248	0.7127	0.4574	0.8453	0.6514	0.6056	0.7500	0.3281
CV-249	0.7055	0.4255	0.8508	0.6382	0.5970	0.7404	0.3054
CV-250	0.7091	0.4362	0.8508	0.6435	0.6029	0.7440	0.3155
CV-251	0.7018	0.4149	0.8508	0.6329	0.5909	0.7368	0.2951
CV-252	0.6945	0.4149	0.8398	0.6273	0.5735	0.7343	0.2800
CV-253	0.7127	0.4255	0.8619	0.6437	0.6154	0.7429	0.3209
CV-254	0.7164	0.4255	0.8674	0.6465	0.6250	0.7441	0.3288
CV-255	0.7127	0.4043	0.8729	0.6386	0.6230	0.7383	0.3164
CV-256	0.7055	0.4362	0.8453	0.6407	0.5942	0.7427	0.3080
CV-257	0.7055	0.4468	0.8398	0.6433	0.5915	0.7451	0.3106
CV-258	0.7018	0.4149	0.8508	0.6329	0.5909	0.7368	0.2951
CV-259	0.6836	0.4043	0.8287	0.6165	0.5507	0.7282	0.2549
CV-260	0.7018	0.4362	0.8398	0.6380	0.5857	0.7415	0.3005
CV-261	0.7091	0.4149	0.8619	0.6384	0.6094	0.7393	0.3107
CV-262	0.7164	0.4468	0.8564	0.6516	0.6176	0.7488	0.3333
CV-263	0.7018	0.3936	0.8619	0.6277	0.5968	0.7324	0.2900
CV-264	0.7236	0.4574	0.8619	0.6597	0.6324	0.7536	0.3511
CV-265	0.7091	0.4043	0.8674	0.6358	0.6129	0.7371	0.3083
CV-266	0.6800	0.3936	0.8287	0.6112	0.5441	0.7246	0.2445
CV-267	0.7164	0.4468	0.8564	0.6516	0.6176	0.7488	0.3333
CV-268	0.7127	0.4787	0.8343	0.6565	0.6000	0.7550	0.3333
CV-269	0.6945	0.4149	0.8398	0.6273	0.5735	0.7343	0.2800
CV-270	0.7018	0.4362	0.8398	0.6380	0.5857	0.7415	0.3005
CV-271	0.7127	0.4362	0.8564	0.6463	0.6119	0.7452	0.3232
CV-272	0.7164	0.4468	0.8564	0.6516	0.6176	0.7488	0.3333
CV-273	0.7236	0.4468	0.8674	0.6571	0.6364	0.7512	0.3490
CV-274	0.7236	0.5000	0.8398	0.6699	0.6184	0.7638	0.3604
CV-275	0.7200	0.4362	0.8674	0.6518	0.6308	0.7476	0.3389
CV-276	0.7091	0.4149	0.8619	0.6384	0.6094	0.7393	0.3107
CV-277	0.7164	0.4574	0.8508	0.6541	0.6143	0.7512	0.3357
CV-278	0.7091	0.4362	0.8508	0.6435	0.6029	0.7440	0.3155

CV-279	0.7091	0.4255	0.8564	0.6409	0.6061	0.7416	0.3131
CV-280	0.6945	0.3936	0.8508	0.6222	0.5781	0.7299	0.2744
CV-281	0.7018	0.4255	0.8453	0.6354	0.5882	0.7391	0.2978
CV-282	0.7018	0.4468	0.8343	0.6405	0.5833	0.7438	0.3032
CV-283	0.6945	0.3936	0.8508	0.6222	0.5781	0.7299	0.2744
CV-284	0.7055	0.4362	0.8453	0.6407	0.5942	0.7427	0.3080
CV-285	0.7127	0.4574	0.8453	0.6514	0.6056	0.7500	0.3281
CV-286	0.7127	0.4362	0.8564	0.6463	0.6119	0.7452	0.3232
CV-287	0.7091	0.4362	0.8508	0.6435	0.6029	0.7440	0.3155
CV-288	0.7055	0.3936	0.8674	0.6305	0.6066	0.7336	0.2980
CV-289	0.6982	0.3936	0.8564	0.6250	0.5873	0.7311	0.2821
CV-290	0.7164	0.4681	0.8453	0.6567	0.6111	0.7537	0.3381
CV-291	0.7273	0.4362	0.8785	0.6573	0.6508	0.7500	0.3551
CV-292	0.7127	0.4681	0.8398	0.6539	0.6027	0.7525	0.3307
CV-293	0.7055	0.4149	0.8564	0.6356	0.6000	0.7381	0.3028
CV-294	0.6982	0.4255	0.8398	0.6327	0.5797	0.7379	0.2903
CV-295	0.7164	0.4574	0.8508	0.6541	0.6143	0.7512	0.3357
CV-296	0.6836	0.3936	0.8343	0.6139	0.5522	0.7260	0.2518
CV-297	0.6982	0.4255	0.8398	0.6327	0.5797	0.7379	0.2903
CV-298	0.7091	0.4468	0.8453	0.6461	0.6000	0.7463	0.3181
CV-299	0.6982	0.4362	0.8343	0.6352	0.5775	0.7402	0.2931
CV-300	0.7018	0.4255	0.8453	0.6354	0.5882	0.7391	0.2978
CV-301	0.7127	0.4362	0.8564	0.6463	0.6119	0.7452	0.3232
CV-302	0.7309	0.4468	0.8785	0.6626	0.6563	0.7536	0.3651
CV-303	0.6982	0.4043	0.8508	0.6275	0.5846	0.7333	0.2848
CV-304	0.6982	0.4255	0.8398	0.6327	0.5797	0.7379	0.2903
CV-305	0.7164	0.4362	0.8619	0.6490	0.6212	0.7464	0.3310
CV-306	0.7091	0.4362	0.8508	0.6435	0.6029	0.7440	0.3155
CV-307	0.7127	0.4149	0.8674	0.6411	0.6190	0.7406	0.3186
CV-308	0.7164	0.4255	0.8674	0.6465	0.6250	0.7441	0.3288
CV-309	0.7055	0.4574	0.8343	0.6459	0.5890	0.7475	0.3133
CV-310	0.7018	0.4043	0.8564	0.6303	0.5938	0.7346	0.2925
CV-311	0.7164	0.4681	0.8453	0.6567	0.6111	0.7537	0.3381
CV-312	0.7018	0.4255	0.8453	0.6354	0.5882	0.7391	0.2978
CV-313	0.7164	0.4574	0.8508	0.6541	0.6143	0.7512	0.3357
CV-314	0.7055	0.4362	0.8453	0.6407	0.5942	0.7427	0.3080
CV-315	0.7200	0.4362	0.8674	0.6518	0.6308	0.7476	0.3389
CV-316	0.7164	0.4468	0.8564	0.6516	0.6176	0.7488	0.3333
CV-317	0.7055	0.4149	0.8564	0.6356	0.6000	0.7381	0.3028
CV-318	0.7091	0.4255	0.8564	0.6409	0.6061	0.7416	0.3131
CV-319	0.6982	0.4362	0.8343	0.6352	0.5775	0.7402	0.2931
CV-320	0.7018	0.4255	0.8453	0.6354	0.5882	0.7391	0.2978
CV-321	0.7164	0.4681	0.8453	0.6567	0.6111	0.7537	0.3381
CV-322	0.6909	0.4043	0.8398	0.6220	0.5672	0.7308	0.2696
CV-323	0.6945	0.4043	0.8453	0.6248	0.5758	0.7321	0.2772
CV-324	0.7055	0.4255	0.8508	0.6382	0.5970	0.7404	0.3054
CV-325	0.7164	0.4255	0.8674	0.6465	0.6250	0.7441	0.3288
CV-326	0.7164	0.4362	0.8619	0.6490	0.6212	0.7464	0.3310
CV-327	0.6836	0.4043	0.8287	0.6165	0.5507	0.7282	0.2549
CV-328	0.7164	0.4362	0.8619	0.6490	0.6212	0.7464	0.3310
CV-329	0.7200	0.4255	0.8729	0.6492	0.6349	0.7453	0.3369
CV-330	0.7236	0.4574	0.8619	0.6597	0.6324	0.7536	0.3511
CV-331	0.6982	0.4255	0.8398	0.6327	0.5797	0.7379	0.2903
CV-332	0.7164	0.4574	0.8508	0.6541	0.6143	0.7512	0.3357
CV-333	0.7273	0.4362	0.8785	0.6573	0.6508	0.7500	0.3551
CV-334	0.7091	0.4043	0.8674	0.6358	0.6129	0.7371	0.3083
CV-335	0.6982	0.4149	0.8453	0.6301	0.5821	0.7356	0.2875

CV-336	0.6909	0.3936	0.8453	0.6195	0.5692	0.7286	0.2667
CV-337	0.6982	0.4362	0.8343	0.6352	0.5775	0.7402	0.2931
CV-338	0.6800	0.4468	0.8011	0.6240	0.5385	0.7360	0.2609
CV-339	0.7164	0.4362	0.8619	0.6490	0.6212	0.7464	0.3310
CV-340	0.6800	0.4149	0.8177	0.6163	0.5417	0.7291	0.2509
CV-341	0.7055	0.4255	0.8508	0.6382	0.5970	0.7404	0.3054
CV-342	0.6909	0.4149	0.8343	0.6246	0.5652	0.7330	0.2726
CV-343	0.6836	0.3936	0.8343	0.6139	0.5522	0.7260	0.2518
CV-344	0.7018	0.4468	0.8343	0.6405	0.5833	0.7438	0.3032
CV-345	0.6982	0.4149	0.8453	0.6301	0.5821	0.7356	0.2875
CV-346	0.7236	0.4255	0.8785	0.6520	0.6452	0.7465	0.3450
CV-347	0.7309	0.4574	0.8729	0.6652	0.6515	0.7560	0.3669
CV-348	0.7018	0.4255	0.8453	0.6354	0.5882	0.7391	0.2978
CV-349	0.6764	0.4043	0.8177	0.6110	0.5352	0.7255	0.2405
CV-350	0.6800	0.3936	0.8287	0.6112	0.5441	0.7246	0.2445
CV-351	0.7236	0.4255	0.8785	0.6520	0.6452	0.7465	0.3450
CV-352	0.6764	0.3723	0.8343	0.6033	0.5385	0.7190	0.2307
CV-353	0.7273	0.4468	0.8729	0.6599	0.6462	0.7524	0.3570
CV-354	0.6945	0.4255	0.8343	0.6299	0.5714	0.7366	0.2829
CV-355	0.6945	0.4149	0.8398	0.6273	0.5735	0.7343	0.2800
CV-356	0.7200	0.4468	0.8619	0.6543	0.6269	0.7500	0.3411
CV-357	0.7273	0.4574	0.8674	0.6624	0.6418	0.7548	0.3589
CV-358	0.7055	0.4468	0.8398	0.6433	0.5915	0.7451	0.3106
CV-359	0.7164	0.4149	0.8729	0.6439	0.6290	0.7418	0.3267
CV-360	0.7091	0.4362	0.8508	0.6435	0.6029	0.7440	0.3155
CV-361	0.7091	0.4149	0.8619	0.6384	0.6094	0.7393	0.3107
CV-362	0.6909	0.4043	0.8398	0.6220	0.5672	0.7308	0.2696
CV-363	0.6945	0.4043	0.8453	0.6248	0.5758	0.7321	0.2772
CV-364	0.6873	0.3936	0.8398	0.6167	0.5606	0.7273	0.2592
CV-365	0.6945	0.3936	0.8508	0.6222	0.5781	0.7299	0.2744
CV-366	0.7018	0.4149	0.8508	0.6329	0.5909	0.7368	0.2951
CV-367	0.7164	0.4362	0.8619	0.6490	0.6212	0.7464	0.3310
CV-368	0.6982	0.4043	0.8508	0.6275	0.5846	0.7333	0.2848
CV-369	0.7091	0.4362	0.8508	0.6435	0.6029	0.7440	0.3155
CV-370	0.6982	0.4149	0.8453	0.6301	0.5821	0.7356	0.2875
CV-371	0.7164	0.4149	0.8729	0.6439	0.6290	0.7418	0.3267
CV-372	0.7055	0.4255	0.8508	0.6382	0.5970	0.7404	0.3054
CV-373	0.7018	0.3830	0.8674	0.6252	0.6000	0.7302	0.2875
CV-374	0.7091	0.4255	0.8564	0.6409	0.6061	0.7416	0.3131
CV-375	0.7055	0.4255	0.8508	0.6382	0.5970	0.7404	0.3054
CV-376	0.7018	0.4362	0.8398	0.6380	0.5857	0.7415	0.3005
CV-377	0.6909	0.3830	0.8508	0.6169	0.5714	0.7264	0.2639
CV-378	0.7018	0.4255	0.8453	0.6354	0.5882	0.7391	0.2978
CV-379	0.6909	0.4362	0.8232	0.6297	0.5616	0.7376	0.2786
CV-380	0.7091	0.4362	0.8508	0.6435	0.6029	0.7440	0.3155
CV-381	0.7055	0.4255	0.8508	0.6382	0.5970	0.7404	0.3054
CV-382	0.7091	0.4255	0.8564	0.6409	0.6061	0.7416	0.3131
CV-383	0.6873	0.4362	0.8177	0.6269	0.5541	0.7363	0.2715
CV-384	0.7018	0.4149	0.8508	0.6329	0.5909	0.7368	0.2951
CV-385	0.6873	0.4255	0.8232	0.6244	0.5556	0.7340	0.2684
CV-386	0.7018	0.4255	0.8453	0.6354	0.5882	0.7391	0.2978
CV-387	0.6873	0.4149	0.8287	0.6218	0.5571	0.7317	0.2653
CV-388	0.6909	0.3936	0.8453	0.6195	0.5692	0.7286	0.2667
CV-389	0.7164	0.4149	0.8729	0.6439	0.6290	0.7418	0.3267
CV-390	0.6836	0.4149	0.8232	0.6190	0.5493	0.7304	0.2581
CV-391	0.6945	0.4043	0.8453	0.6248	0.5758	0.7321	0.2772
CV-392	0.6982	0.3936	0.8564	0.6250	0.5873	0.7311	0.2821

CV-393	0.6873	0.3936	0.8398	0.6167	0.5606	0.7273	0.2592
CV-394	0.6873	0.4043	0.8343	0.6193	0.5588	0.7295	0.2622
CV-395	0.7164	0.4255	0.8674	0.6465	0.6250	0.7441	0.3288
CV-396	0.6945	0.4255	0.8343	0.6299	0.5714	0.7366	0.2829
CV-397	0.7018	0.4362	0.8398	0.6380	0.5857	0.7415	0.3005
CV-398	0.7164	0.4362	0.8619	0.6490	0.6212	0.7464	0.3310
CV-399	0.7127	0.4362	0.8564	0.6463	0.6119	0.7452	0.3232
CV-400	0.6836	0.3723	0.8453	0.6088	0.5556	0.7217	0.2456
CV-401	0.7127	0.4149	0.8674	0.6411	0.6190	0.7406	0.3186
CV-402	0.7127	0.4362	0.8564	0.6463	0.6119	0.7452	0.3232
CV-403	0.7091	0.4043	0.8674	0.6358	0.6129	0.7371	0.3083
CV-404	0.7200	0.4574	0.8564	0.6569	0.6232	0.7524	0.3433
CV-405	0.6764	0.3723	0.8343	0.6033	0.5385	0.7190	0.2307
CV-406	0.7200	0.4362	0.8674	0.6518	0.6308	0.7476	0.3389
CV-407	0.6982	0.4043	0.8508	0.6275	0.5846	0.7333	0.2848
CV-408	0.7273	0.4362	0.8785	0.6573	0.6508	0.7500	0.3551
CV-409	0.7200	0.4468	0.8619	0.6543	0.6269	0.7500	0.3411
CV-410	0.7236	0.4255	0.8785	0.6520	0.6452	0.7465	0.3450
CV-411	0.6982	0.4149	0.8453	0.6301	0.5821	0.7356	0.2875
CV-412	0.7200	0.4574	0.8564	0.6569	0.6232	0.7524	0.3433
CV-413	0.6945	0.4574	0.8177	0.6376	0.5658	0.7437	0.2918
CV-414	0.7127	0.4362	0.8564	0.6463	0.6119	0.7452	0.3232
CV-415	0.7127	0.4362	0.8564	0.6463	0.6119	0.7452	0.3232
CV-416	0.7127	0.4468	0.8508	0.6488	0.6087	0.7476	0.3256
CV-417	0.6836	0.4362	0.8122	0.6242	0.5467	0.7350	0.2645
CV-418	0.7091	0.3936	0.8729	0.6333	0.6167	0.7349	0.3061
CV-419	0.6982	0.4362	0.8343	0.6352	0.5775	0.7402	0.2931
CV-420	0.7018	0.4255	0.8453	0.6354	0.5882	0.7391	0.2978
CV-421	0.7055	0.4149	0.8564	0.6356	0.6000	0.7381	0.3028
CV-422	0.6873	0.4043	0.8343	0.6193	0.5588	0.7295	0.2622
CV-423	0.6727	0.3830	0.8232	0.6031	0.5294	0.7198	0.2267
CV-424	0.7018	0.4149	0.8508	0.6329	0.5909	0.7368	0.2951
CV-425	0.7055	0.4149	0.8564	0.6356	0.6000	0.7381	0.3028
CV-426	0.7018	0.3936	0.8619	0.6277	0.5968	0.7324	0.2900
CV-427	0.7018	0.4468	0.8343	0.6405	0.5833	0.7438	0.3032
CV-428	0.6691	0.4043	0.8066	0.6054	0.5205	0.7228	0.2265
CV-429	0.7127	0.4043	0.8729	0.6386	0.6230	0.7383	0.3164
CV-430	0.7091	0.4574	0.8398	0.6486	0.5972	0.7488	0.3207
CV-431	0.7236	0.4362	0.8729	0.6545	0.6406	0.7488	0.3470
CV-432	0.7200	0.4574	0.8564	0.6569	0.6232	0.7524	0.3433
CV-433	0.6873	0.4255	0.8232	0.6244	0.5556	0.7340	0.2684
CV-434	0.7091	0.4362	0.8508	0.6435	0.6029	0.7440	0.3155
CV-435	0.7127	0.4468	0.8508	0.6488	0.6087	0.7476	0.3256
CV-436	0.7091	0.4043	0.8674	0.6358	0.6129	0.7371	0.3083
CV-437	0.7091	0.4468	0.8453	0.6461	0.6000	0.7463	0.3181
CV-438	0.6945	0.4255	0.8343	0.6299	0.5714	0.7366	0.2829
CV-439	0.6945	0.4255	0.8343	0.6299	0.5714	0.7366	0.2829
CV-440	0.7127	0.4149	0.8674	0.6411	0.6190	0.7406	0.3186
CV-441	0.6873	0.3723	0.8508	0.6116	0.5645	0.7230	0.2533
CV-442	0.6945	0.4149	0.8398	0.6273	0.5735	0.7343	0.2800
CV-443	0.6909	0.4255	0.8287	0.6271	0.5634	0.7353	0.2756
CV-444	0.7018	0.4255	0.8453	0.6354	0.5882	0.7391	0.2978
CV-445	0.6873	0.4149	0.8287	0.6218	0.5571	0.7317	0.2653
CV-446	0.6982	0.4043	0.8508	0.6275	0.5846	0.7333	0.2848
CV-447	0.7018	0.4574	0.8287	0.6431	0.5811	0.7463	0.3061
CV-448	0.7164	0.4255	0.8674	0.6465	0.6250	0.7441	0.3288
CV-449	0.7127	0.4574	0.8453	0.6514	0.6056	0.7500	0.3281

CV-450	0.7164	0.4149	0.8729	0.6439	0.6290	0.7418	0.3267
CV-451	0.6982	0.4043	0.8508	0.6275	0.5846	0.7333	0.2848
CV-452	0.7200	0.4362	0.8674	0.6518	0.6308	0.7476	0.3389
CV-453	0.6800	0.3830	0.8343	0.6086	0.5455	0.7225	0.2413
CV-454	0.7055	0.4255	0.8508	0.6382	0.5970	0.7404	0.3054
CV-455	0.6909	0.4362	0.8232	0.6297	0.5616	0.7376	0.2786
CV-456	0.7164	0.4468	0.8564	0.6516	0.6176	0.7488	0.3333
CV-457	0.7127	0.4149	0.8674	0.6411	0.6190	0.7406	0.3186
CV-458	0.6873	0.4255	0.8232	0.6244	0.5556	0.7340	0.2684
CV-459	0.7055	0.4149	0.8564	0.6356	0.6000	0.7381	0.3028
CV-460	0.7236	0.4468	0.8674	0.6571	0.6364	0.7512	0.3490
CV-461	0.7236	0.4362	0.8729	0.6545	0.6406	0.7488	0.3470
CV-462	0.6873	0.3723	0.8508	0.6116	0.5645	0.7230	0.2533
CV-463	0.7018	0.4468	0.8343	0.6405	0.5833	0.7438	0.3032
CV-464	0.7236	0.4574	0.8619	0.6597	0.6324	0.7536	0.3511
CV-465	0.7091	0.4362	0.8508	0.6435	0.6029	0.7440	0.3155
CV-466	0.6909	0.4149	0.8343	0.6246	0.5652	0.7330	0.2726
CV-467	0.6909	0.3830	0.8508	0.6169	0.5714	0.7264	0.2639
CV-468	0.7127	0.4043	0.8729	0.6386	0.6230	0.7383	0.3164
CV-469	0.7127	0.4362	0.8564	0.6463	0.6119	0.7452	0.3232
CV-470	0.6836	0.4149	0.8232	0.6190	0.5493	0.7304	0.2581
CV-471	0.7091	0.4681	0.8343	0.6512	0.5946	0.7512	0.3234
CV-472	0.7236	0.4468	0.8674	0.6571	0.6364	0.7512	0.3490
CV-473	0.7164	0.4149	0.8729	0.6439	0.6290	0.7418	0.3267
CV-474	0.6909	0.4574	0.8122	0.6348	0.5584	0.7424	0.2848
CV-475	0.6945	0.4043	0.8453	0.6248	0.5758	0.7321	0.2772
CV-476	0.7200	0.4681	0.8508	0.6595	0.6197	0.7549	0.3456
CV-477	0.6982	0.4255	0.8398	0.6327	0.5797	0.7379	0.2903
CV-478	0.7091	0.4043	0.8674	0.6358	0.6129	0.7371	0.3083
CV-479	0.7273	0.4574	0.8674	0.6624	0.6418	0.7548	0.3589
CV-480	0.6836	0.3936	0.8343	0.6139	0.5522	0.7260	0.2518
CV-481	0.7127	0.4255	0.8619	0.6437	0.6154	0.7429	0.3209
CV-482	0.7091	0.4362	0.8508	0.6435	0.6029	0.7440	0.3155
CV-483	0.6836	0.4255	0.8177	0.6216	0.5479	0.7327	0.2612
CV-484	0.6945	0.4255	0.8343	0.6299	0.5714	0.7366	0.2829
CV-485	0.6909	0.4043	0.8398	0.6220	0.5672	0.7308	0.2696
CV-486	0.7018	0.3936	0.8619	0.6277	0.5968	0.7324	0.2900
CV-487	0.6836	0.3830	0.8398	0.6114	0.5538	0.7238	0.2487
CV-488	0.7091	0.4255	0.8564	0.6409	0.6061	0.7416	0.3131
CV-489	0.6909	0.4574	0.8122	0.6348	0.5584	0.7424	0.2848
CV-490	0.6909	0.4043	0.8398	0.6220	0.5672	0.7308	0.2696
CV-491	0.7345	0.4787	0.8674	0.6731	0.6522	0.7621	0.3787
CV-492	0.7236	0.4468	0.8674	0.6571	0.6364	0.7512	0.3490
CV-493	0.6982	0.4574	0.8232	0.6403	0.5733	0.7450	0.2989
CV-494	0.6945	0.4362	0.8287	0.6324	0.5694	0.7389	0.2858
CV-495	0.7236	0.4468	0.8674	0.6571	0.6364	0.7512	0.3490
CV-496	0.7018	0.4149	0.8508	0.6329	0.5909	0.7368	0.2951
CV-497	0.6982	0.3936	0.8564	0.6250	0.5873	0.7311	0.2821
CV-498	0.6909	0.4043	0.8398	0.6220	0.5672	0.7308	0.2696
CV-499	0.6982	0.4255	0.8398	0.6327	0.5797	0.7379	0.2903
CV-500	0.7164	0.4255	0.8674	0.6465	0.6250	0.7441	0.3288

Table S10. Performance of 5-fold cross-validations from logistic regression (LR). Performance of 5-fold cross-validations were evaluated by seven performance metrics. Each row shows the performance of one time 5-fold cross-validation. 5-fold cross-validation was repeated 500 times (CV-1 ~ CV-500).

Cross-validation	Accuracy	Sensitivity	Specificity	Balanced accuracy	Positive prediction rate	Negative prediction rate	Matthews correlation coefficient
CV-1	0.7345	0.5213	0.8453	0.6833	0.6364	0.7727	0.3873
CV-2	0.6655	0.4149	0.7956	0.6052	0.5132	0.7236	0.2232
CV-3	0.7055	0.4787	0.8232	0.6510	0.5844	0.7525	0.3190
CV-4	0.6509	0.4362	0.7624	0.5993	0.4881	0.7225	0.2045
CV-5	0.6582	0.4255	0.7790	0.6023	0.5000	0.7231	0.2136
CV-6	0.7091	0.5000	0.8177	0.6588	0.5875	0.7590	0.3318
CV-7	0.6764	0.4362	0.8011	0.6186	0.5325	0.7323	0.2507
CV-8	0.6836	0.4574	0.8011	0.6293	0.5443	0.7398	0.2710
CV-9	0.6691	0.4043	0.8066	0.6054	0.5205	0.7228	0.2265
CV-10	0.6691	0.3936	0.8122	0.6029	0.5211	0.7206	0.2230
CV-11	0.6873	0.4894	0.7901	0.6397	0.5476	0.7487	0.2877
CV-12	0.6655	0.4468	0.7790	0.6129	0.5122	0.7306	0.2341
CV-13	0.6691	0.4149	0.8011	0.6080	0.5200	0.7250	0.2300
CV-14	0.6764	0.4468	0.7956	0.6212	0.5316	0.7347	0.2541
CV-15	0.6982	0.4468	0.8287	0.6378	0.5753	0.7426	0.2960
CV-16	0.7164	0.4574	0.8508	0.6541	0.6143	0.7512	0.3357
CV-17	0.7127	0.4787	0.8343	0.6565	0.6000	0.7550	0.3333
CV-18	0.7018	0.4468	0.8343	0.6405	0.5833	0.7438	0.3032
CV-19	0.6800	0.4255	0.8122	0.6188	0.5405	0.7313	0.2542
CV-20	0.6691	0.4043	0.8066	0.6054	0.5205	0.7228	0.2265
CV-21	0.6582	0.4149	0.7845	0.5997	0.5000	0.7208	0.2098
CV-22	0.6691	0.4149	0.8011	0.6080	0.5200	0.7250	0.2300
CV-23	0.7018	0.4362	0.8398	0.6380	0.5857	0.7415	0.3005
CV-24	0.6945	0.4681	0.8122	0.6401	0.5641	0.7462	0.2949
CV-25	0.7018	0.4787	0.8177	0.6482	0.5769	0.7513	0.3119
CV-26	0.6691	0.4255	0.7956	0.6106	0.5195	0.7273	0.2336
CV-27	0.6836	0.4362	0.8122	0.6242	0.5467	0.7350	0.2645
CV-28	0.7018	0.4574	0.8287	0.6431	0.5811	0.7463	0.3061
CV-29	0.6982	0.4149	0.8453	0.6301	0.5821	0.7356	0.2875
CV-30	0.6764	0.4468	0.7956	0.6212	0.5316	0.7347	0.2541
CV-31	0.7164	0.4787	0.8398	0.6593	0.6081	0.7562	0.3406
CV-32	0.6800	0.4255	0.8122	0.6188	0.5405	0.7313	0.2542
CV-33	0.6800	0.4681	0.7901	0.6291	0.5366	0.7409	0.2677
CV-34	0.6764	0.4149	0.8122	0.6135	0.5342	0.7277	0.2439
CV-35	0.6982	0.4787	0.8122	0.6454	0.5696	0.7500	0.3049
CV-36	0.6982	0.4468	0.8287	0.6378	0.5753	0.7426	0.2960
CV-37	0.6727	0.4149	0.8066	0.6108	0.5270	0.7264	0.2369
CV-38	0.6618	0.4043	0.7956	0.5999	0.5067	0.7200	0.2128
CV-39	0.6727	0.4149	0.8066	0.6108	0.5270	0.7264	0.2369
CV-40	0.6473	0.4149	0.7680	0.5914	0.4815	0.7165	0.1903
CV-41	0.6836	0.3936	0.8343	0.6139	0.5522	0.7260	0.2518
CV-42	0.7164	0.4787	0.8398	0.6593	0.6081	0.7562	0.3406
CV-43	0.6873	0.4255	0.8232	0.6244	0.5556	0.7340	0.2684
CV-44	0.6836	0.4362	0.8122	0.6242	0.5467	0.7350	0.2645
CV-45	0.6909	0.4362	0.8232	0.6297	0.5616	0.7376	0.2786
CV-46	0.6691	0.4255	0.7956	0.6106	0.5195	0.7273	0.2336
CV-47	0.6436	0.4255	0.7569	0.5912	0.4762	0.7173	0.1879
CV-48	0.6582	0.4362	0.7735	0.6048	0.5000	0.7254	0.2174
CV-49	0.6800	0.4362	0.8066	0.6214	0.5395	0.7337	0.2575
CV-50	0.6982	0.4362	0.8343	0.6352	0.5775	0.7402	0.2931

CV-51	0.6873	0.4362	0.8177	0.6269	0.5541	0.7363	0.2715
CV-52	0.6909	0.4362	0.8232	0.6297	0.5616	0.7376	0.2786
CV-53	0.7236	0.4894	0.8453	0.6673	0.6216	0.7612	0.3579
CV-54	0.6800	0.4894	0.7790	0.6342	0.5349	0.7460	0.2746
CV-55	0.6836	0.4681	0.7956	0.6318	0.5432	0.7423	0.2744
CV-56	0.6655	0.4043	0.8011	0.6027	0.5135	0.7214	0.2196
CV-57	0.7091	0.4362	0.8508	0.6435	0.6029	0.7440	0.3155
CV-58	0.6945	0.4468	0.8232	0.6350	0.5676	0.7413	0.2888
CV-59	0.7127	0.4894	0.8287	0.6590	0.5974	0.7576	0.3360
CV-60	0.6909	0.4255	0.8287	0.6271	0.5634	0.7353	0.2756
CV-61	0.6836	0.3936	0.8343	0.6139	0.5522	0.7260	0.2518
CV-62	0.6727	0.4468	0.7901	0.6184	0.5250	0.7333	0.2474
CV-63	0.6764	0.4468	0.7956	0.6212	0.5316	0.7347	0.2541
CV-64	0.6982	0.4894	0.8066	0.6480	0.5679	0.7526	0.3080
CV-65	0.6800	0.4362	0.8066	0.6214	0.5395	0.7337	0.2575
CV-66	0.6800	0.4043	0.8232	0.6137	0.5429	0.7268	0.2477
CV-67	0.6691	0.4362	0.7901	0.6131	0.5190	0.7296	0.2371
CV-68	0.6836	0.4468	0.8066	0.6267	0.5455	0.7374	0.2677
CV-69	0.6691	0.4149	0.8011	0.6080	0.5200	0.7250	0.2300
CV-70	0.6800	0.3936	0.8287	0.6112	0.5441	0.7246	0.2445
CV-71	0.6873	0.4574	0.8066	0.6320	0.5513	0.7411	0.2779
CV-72	0.6618	0.4468	0.7735	0.6101	0.5060	0.7292	0.2276
CV-73	0.6618	0.4255	0.7845	0.6050	0.5063	0.7245	0.2202
CV-74	0.7018	0.4681	0.8232	0.6456	0.5789	0.7487	0.3090
CV-75	0.6836	0.4574	0.8011	0.6293	0.5443	0.7398	0.2710
CV-76	0.6945	0.4574	0.8177	0.6376	0.5658	0.7437	0.2918
CV-77	0.6764	0.4255	0.8066	0.6161	0.5333	0.7300	0.2473
CV-78	0.6909	0.4362	0.8232	0.6297	0.5616	0.7376	0.2786
CV-79	0.7055	0.4468	0.8398	0.6433	0.5915	0.7451	0.3106
CV-80	0.6909	0.4149	0.8343	0.6246	0.5652	0.7330	0.2726
CV-81	0.6764	0.4362	0.8011	0.6186	0.5325	0.7323	0.2507
CV-82	0.6691	0.4255	0.7956	0.6106	0.5195	0.7273	0.2336
CV-83	0.6945	0.4574	0.8177	0.6376	0.5658	0.7437	0.2918
CV-84	0.6764	0.4043	0.8177	0.6110	0.5352	0.7255	0.2405
CV-85	0.6582	0.4255	0.7790	0.6023	0.5000	0.7231	0.2136
CV-86	0.6727	0.3936	0.8177	0.6056	0.5286	0.7220	0.2301
CV-87	0.6836	0.4149	0.8232	0.6190	0.5493	0.7304	0.2581
CV-88	0.7091	0.4574	0.8398	0.6486	0.5972	0.7488	0.3207
CV-89	0.6727	0.4574	0.7845	0.6210	0.5244	0.7358	0.2509
CV-90	0.6909	0.4574	0.8122	0.6348	0.5584	0.7424	0.2848
CV-91	0.6836	0.4255	0.8177	0.6216	0.5479	0.7327	0.2612
CV-92	0.7018	0.4468	0.8343	0.6405	0.5833	0.7438	0.3032
CV-93	0.6945	0.4681	0.8122	0.6401	0.5641	0.7462	0.2949
CV-94	0.7127	0.4681	0.8398	0.6539	0.6027	0.7525	0.3307
CV-95	0.7055	0.4681	0.8287	0.6484	0.5867	0.7500	0.3161
CV-96	0.6727	0.4362	0.7956	0.6159	0.5256	0.7310	0.2439
CV-97	0.6655	0.3936	0.8066	0.6001	0.5139	0.7192	0.2161
CV-98	0.6909	0.4043	0.8398	0.6220	0.5672	0.7308	0.2696
CV-99	0.7055	0.4468	0.8398	0.6433	0.5915	0.7451	0.3106
CV-100	0.6618	0.4255	0.7845	0.6050	0.5063	0.7245	0.2202
CV-101	0.6945	0.4574	0.8177	0.6376	0.5658	0.7437	0.2918
CV-102	0.6691	0.3723	0.8232	0.5978	0.5224	0.7163	0.2161
CV-103	0.7164	0.4468	0.8564	0.6516	0.6176	0.7488	0.3333
CV-104	0.6618	0.4149	0.7901	0.6025	0.5065	0.7222	0.2165
CV-105	0.6400	0.4149	0.7569	0.5859	0.4699	0.7135	0.1775
CV-106	0.6909	0.4149	0.8343	0.6246	0.5652	0.7330	0.2726
CV-107	0.6945	0.4574	0.8177	0.6376	0.5658	0.7437	0.2918

CV-108	0.6945	0.4362	0.8287	0.6324	0.5694	0.7389	0.2858
CV-109	0.6800	0.4149	0.8177	0.6163	0.5417	0.7291	0.2509
CV-110	0.6873	0.4468	0.8122	0.6295	0.5526	0.7387	0.2747
CV-111	0.6836	0.4681	0.7956	0.6318	0.5432	0.7423	0.2744
CV-112	0.6691	0.4043	0.8066	0.6054	0.5205	0.7228	0.2265
CV-113	0.6945	0.4574	0.8177	0.6376	0.5658	0.7437	0.2918
CV-114	0.6873	0.3936	0.8398	0.6167	0.5606	0.7273	0.2592
CV-115	0.6582	0.4362	0.7735	0.6048	0.5000	0.7254	0.2174
CV-116	0.6545	0.3830	0.7956	0.5893	0.4932	0.7129	0.1918
CV-117	0.7055	0.4787	0.8232	0.6510	0.5844	0.7525	0.3190
CV-118	0.6582	0.3830	0.8011	0.5920	0.5000	0.7143	0.1986
CV-119	0.6836	0.4894	0.7845	0.6369	0.5412	0.7474	0.2811
CV-120	0.7055	0.4468	0.8398	0.6433	0.5915	0.7451	0.3106
CV-121	0.7055	0.4362	0.8453	0.6407	0.5942	0.7427	0.3080
CV-122	0.6473	0.4043	0.7735	0.5889	0.4810	0.7143	0.1863
CV-123	0.6800	0.4362	0.8066	0.6214	0.5395	0.7337	0.2575
CV-124	0.6727	0.4149	0.8066	0.6108	0.5270	0.7264	0.2369
CV-125	0.6945	0.4149	0.8398	0.6273	0.5735	0.7343	0.2800
CV-126	0.6582	0.3936	0.7956	0.5946	0.5000	0.7164	0.2024
CV-127	0.6800	0.4468	0.8011	0.6240	0.5385	0.7360	0.2609
CV-128	0.7127	0.4362	0.8564	0.6463	0.6119	0.7452	0.3232
CV-129	0.6582	0.4574	0.7624	0.6099	0.5000	0.7302	0.2250
CV-130	0.6509	0.3830	0.7901	0.5865	0.4865	0.7114	0.1851
CV-131	0.7127	0.5106	0.8177	0.6642	0.5926	0.7629	0.3416
CV-132	0.7127	0.4681	0.8398	0.6539	0.6027	0.7525	0.3307
CV-133	0.6800	0.4043	0.8232	0.6137	0.5429	0.7268	0.2477
CV-134	0.6691	0.3830	0.8177	0.6003	0.5217	0.7184	0.2195
CV-135	0.6873	0.3936	0.8398	0.6167	0.5606	0.7273	0.2592
CV-136	0.6982	0.4043	0.8508	0.6275	0.5846	0.7333	0.2848
CV-137	0.6800	0.4043	0.8232	0.6137	0.5429	0.7268	0.2477
CV-138	0.6836	0.4574	0.8011	0.6293	0.5443	0.7398	0.2710
CV-139	0.6436	0.3617	0.7901	0.5759	0.4722	0.7044	0.1637
CV-140	0.6982	0.4255	0.8398	0.6327	0.5797	0.7379	0.2903
CV-141	0.7200	0.4681	0.8508	0.6595	0.6197	0.7549	0.3456
CV-142	0.6655	0.4362	0.7845	0.6104	0.5125	0.7282	0.2305
CV-143	0.6800	0.4043	0.8232	0.6137	0.5429	0.7268	0.2477
CV-144	0.6509	0.3936	0.7845	0.5891	0.4868	0.7136	0.1890
CV-145	0.6800	0.4149	0.8177	0.6163	0.5417	0.7291	0.2509
CV-146	0.6655	0.4149	0.7956	0.6052	0.5132	0.7236	0.2232
CV-147	0.6545	0.4149	0.7790	0.5969	0.4937	0.7194	0.2033
CV-148	0.6800	0.4043	0.8232	0.6137	0.5429	0.7268	0.2477
CV-149	0.6655	0.4149	0.7956	0.6052	0.5132	0.7236	0.2232
CV-150	0.6909	0.4362	0.8232	0.6297	0.5616	0.7376	0.2786
CV-151	0.6982	0.4681	0.8177	0.6429	0.5714	0.7475	0.3019
CV-152	0.6909	0.4681	0.8066	0.6374	0.5570	0.7449	0.2880
CV-153	0.6655	0.4149	0.7956	0.6052	0.5132	0.7236	0.2232
CV-154	0.6982	0.4468	0.8287	0.6378	0.5753	0.7426	0.2960
CV-155	0.6800	0.4149	0.8177	0.6163	0.5417	0.7291	0.2509
CV-156	0.6727	0.4681	0.7790	0.6235	0.5238	0.7382	0.2545
CV-157	0.7018	0.4787	0.8177	0.6482	0.5769	0.7513	0.3119
CV-158	0.6618	0.4681	0.7624	0.6153	0.5057	0.7340	0.2351
CV-159	0.6473	0.4149	0.7680	0.5914	0.4815	0.7165	0.1903
CV-160	0.6873	0.4149	0.8287	0.6218	0.5571	0.7317	0.2653
CV-161	0.6545	0.4255	0.7735	0.5995	0.4938	0.7216	0.2071
CV-162	0.6945	0.4468	0.8232	0.6350	0.5676	0.7413	0.2888
CV-163	0.6727	0.4043	0.8122	0.6082	0.5278	0.7241	0.2335
CV-164	0.6691	0.4468	0.7845	0.6157	0.5185	0.7320	0.2407

CV-165	0.6545	0.4255	0.7735	0.5995	0.4938	0.7216	0.2071
CV-166	0.6836	0.4681	0.7956	0.6318	0.5432	0.7423	0.2744
CV-167	0.6582	0.4149	0.7845	0.5997	0.5000	0.7208	0.2098
CV-168	0.7200	0.4894	0.8398	0.6646	0.6133	0.7600	0.3505
CV-169	0.6764	0.4468	0.7956	0.6212	0.5316	0.7347	0.2541
CV-170	0.6982	0.4468	0.8287	0.6378	0.5753	0.7426	0.2960
CV-171	0.6982	0.4468	0.8287	0.6378	0.5753	0.7426	0.2960
CV-172	0.6873	0.4894	0.7901	0.6397	0.5476	0.7487	0.2877
CV-173	0.6764	0.3723	0.8343	0.6033	0.5385	0.7190	0.2307
CV-174	0.6945	0.4681	0.8122	0.6401	0.5641	0.7462	0.2949
CV-175	0.6873	0.4468	0.8122	0.6295	0.5526	0.7387	0.2747
CV-176	0.6873	0.4149	0.8287	0.6218	0.5571	0.7317	0.2653
CV-177	0.6909	0.4681	0.8066	0.6374	0.5570	0.7449	0.2880
CV-178	0.6764	0.4255	0.8066	0.6161	0.5333	0.7300	0.2473
CV-179	0.6909	0.4149	0.8343	0.6246	0.5652	0.7330	0.2726
CV-180	0.6727	0.4255	0.8011	0.6133	0.5263	0.7286	0.2404
CV-181	0.6545	0.3830	0.7956	0.5893	0.4932	0.7129	0.1918
CV-182	0.6909	0.4362	0.8232	0.6297	0.5616	0.7376	0.2786
CV-183	0.6764	0.4255	0.8066	0.6161	0.5333	0.7300	0.2473
CV-184	0.6691	0.4468	0.7845	0.6157	0.5185	0.7320	0.2407
CV-185	0.6691	0.4149	0.8011	0.6080	0.5200	0.7250	0.2300
CV-186	0.7018	0.4149	0.8508	0.6329	0.5909	0.7368	0.2951
CV-187	0.6836	0.4043	0.8287	0.6165	0.5507	0.7282	0.2549
CV-188	0.6982	0.4255	0.8398	0.6327	0.5797	0.7379	0.2903
CV-189	0.6618	0.3936	0.8011	0.5974	0.5068	0.7178	0.2092
CV-190	0.6545	0.4362	0.7680	0.6021	0.4940	0.7240	0.2109
CV-191	0.6691	0.4043	0.8066	0.6054	0.5205	0.7228	0.2265
CV-192	0.6836	0.4043	0.8287	0.6165	0.5507	0.7282	0.2549
CV-193	0.6764	0.4468	0.7956	0.6212	0.5316	0.7347	0.2541
CV-194	0.6909	0.4255	0.8287	0.6271	0.5634	0.7353	0.2756
CV-195	0.6909	0.4362	0.8232	0.6297	0.5616	0.7376	0.2786
CV-196	0.7018	0.4787	0.8177	0.6482	0.5769	0.7513	0.3119
CV-197	0.6509	0.4149	0.7735	0.5942	0.4875	0.7179	0.1967
CV-198	0.6873	0.4681	0.8011	0.6346	0.5500	0.7436	0.2811
CV-199	0.6873	0.4255	0.8232	0.6244	0.5556	0.7340	0.2684
CV-200	0.6836	0.4574	0.8011	0.6293	0.5443	0.7398	0.2710
CV-201	0.6873	0.4362	0.8177	0.6269	0.5541	0.7363	0.2715
CV-202	0.7018	0.4681	0.8232	0.6456	0.5789	0.7487	0.3090
CV-203	0.6618	0.4149	0.7901	0.6025	0.5065	0.7222	0.2165
CV-204	0.6836	0.4894	0.7845	0.6369	0.5412	0.7474	0.2811
CV-205	0.6655	0.4681	0.7680	0.6180	0.5116	0.7354	0.2415
CV-206	0.7055	0.4574	0.8343	0.6459	0.5890	0.7475	0.3133
CV-207	0.6691	0.4468	0.7845	0.6157	0.5185	0.7320	0.2407
CV-208	0.6764	0.4574	0.7901	0.6238	0.5309	0.7371	0.2575
CV-209	0.6909	0.4362	0.8232	0.6297	0.5616	0.7376	0.2786
CV-210	0.6727	0.4255	0.8011	0.6133	0.5263	0.7286	0.2404
CV-211	0.6691	0.4362	0.7901	0.6131	0.5190	0.7296	0.2371
CV-212	0.7127	0.4787	0.8343	0.6565	0.6000	0.7550	0.3333
CV-213	0.6873	0.4149	0.8287	0.6218	0.5571	0.7317	0.2653
CV-214	0.6873	0.4681	0.8011	0.6346	0.5500	0.7436	0.2811
CV-215	0.6764	0.4362	0.8011	0.6186	0.5325	0.7323	0.2507
CV-216	0.6655	0.4149	0.7956	0.6052	0.5132	0.7236	0.2232
CV-217	0.6691	0.4574	0.7790	0.6182	0.5181	0.7344	0.2443
CV-218	0.6909	0.4574	0.8122	0.6348	0.5584	0.7424	0.2848
CV-219	0.7164	0.4468	0.8564	0.6516	0.6176	0.7488	0.3333
CV-220	0.6764	0.4468	0.7956	0.6212	0.5316	0.7347	0.2541
CV-221	0.6909	0.4574	0.8122	0.6348	0.5584	0.7424	0.2848

CV-222	0.6800	0.4574	0.7956	0.6265	0.5375	0.7385	0.2642
CV-223	0.6691	0.3830	0.8177	0.6003	0.5217	0.7184	0.2195
CV-224	0.6909	0.4255	0.8287	0.6271	0.5634	0.7353	0.2756
CV-225	0.6436	0.4255	0.7569	0.5912	0.4762	0.7173	0.1879
CV-226	0.6691	0.4681	0.7735	0.6208	0.5176	0.7368	0.2479
CV-227	0.6582	0.4043	0.7901	0.5972	0.5000	0.7186	0.2061
CV-228	0.6582	0.3830	0.8011	0.5920	0.5000	0.7143	0.1986
CV-229	0.6945	0.4362	0.8287	0.6324	0.5694	0.7389	0.2858
CV-230	0.6655	0.3936	0.8066	0.6001	0.5139	0.7192	0.2161
CV-231	0.7055	0.4468	0.8398	0.6433	0.5915	0.7451	0.3106
CV-232	0.6618	0.4574	0.7680	0.6127	0.5059	0.7316	0.2314
CV-233	0.7200	0.4468	0.8619	0.6543	0.6269	0.7500	0.3411
CV-234	0.6909	0.4362	0.8232	0.6297	0.5616	0.7376	0.2786
CV-235	0.7091	0.5000	0.8177	0.6588	0.5875	0.7590	0.3318
CV-236	0.7127	0.4787	0.8343	0.6565	0.6000	0.7550	0.3333
CV-237	0.6618	0.4681	0.7624	0.6153	0.5057	0.7340	0.2351
CV-238	0.6836	0.4255	0.8177	0.6216	0.5479	0.7327	0.2612
CV-239	0.6836	0.4787	0.7901	0.6344	0.5422	0.7448	0.2777
CV-240	0.6873	0.4468	0.8122	0.6295	0.5526	0.7387	0.2747
CV-241	0.6800	0.4362	0.8066	0.6214	0.5395	0.7337	0.2575
CV-242	0.6873	0.4574	0.8066	0.6320	0.5513	0.7411	0.2779
CV-243	0.6618	0.4149	0.7901	0.6025	0.5065	0.7222	0.2165
CV-244	0.6764	0.4468	0.7956	0.6212	0.5316	0.7347	0.2541
CV-245	0.6982	0.4468	0.8287	0.6378	0.5753	0.7426	0.2960
CV-246	0.6800	0.4574	0.7956	0.6265	0.5375	0.7385	0.2642
CV-247	0.6800	0.4468	0.8011	0.6240	0.5385	0.7360	0.2609
CV-248	0.6982	0.4681	0.8177	0.6429	0.5714	0.7475	0.3019
CV-249	0.7091	0.4362	0.8508	0.6435	0.6029	0.7440	0.3155
CV-250	0.7091	0.4468	0.8453	0.6461	0.6000	0.7463	0.3181
CV-251	0.6836	0.4043	0.8287	0.6165	0.5507	0.7282	0.2549
CV-252	0.6691	0.4043	0.8066	0.6054	0.5205	0.7228	0.2265
CV-253	0.7055	0.4574	0.8343	0.6459	0.5890	0.7475	0.3133
CV-254	0.7091	0.3936	0.8729	0.6333	0.6167	0.7349	0.3061
CV-255	0.6945	0.4574	0.8177	0.6376	0.5658	0.7437	0.2918
CV-256	0.7018	0.4574	0.8287	0.6431	0.5811	0.7463	0.3061
CV-257	0.6909	0.3936	0.8453	0.6195	0.5692	0.7286	0.2667
CV-258	0.6945	0.4255	0.8343	0.6299	0.5714	0.7366	0.2829
CV-259	0.6836	0.4468	0.8066	0.6267	0.5455	0.7374	0.2677
CV-260	0.6873	0.4362	0.8177	0.6269	0.5541	0.7363	0.2715
CV-261	0.6945	0.4468	0.8232	0.6350	0.5676	0.7413	0.2888
CV-262	0.6764	0.4043	0.8177	0.6110	0.5352	0.7255	0.2405
CV-263	0.6727	0.4255	0.8011	0.6133	0.5263	0.7286	0.2404
CV-264	0.7018	0.4468	0.8343	0.6405	0.5833	0.7438	0.3032
CV-265	0.6727	0.3830	0.8232	0.6031	0.5294	0.7198	0.2267
CV-266	0.7236	0.4894	0.8453	0.6673	0.6216	0.7612	0.3579
CV-267	0.6909	0.4468	0.8177	0.6322	0.5600	0.7400	0.2817
CV-268	0.7018	0.4574	0.8287	0.6431	0.5811	0.7463	0.3061
CV-269	0.6800	0.4468	0.8011	0.6240	0.5385	0.7360	0.2609
CV-270	0.6764	0.4149	0.8122	0.6135	0.5342	0.7277	0.2439
CV-271	0.6945	0.4149	0.8398	0.6273	0.5735	0.7343	0.2800
CV-272	0.7018	0.4894	0.8122	0.6508	0.5750	0.7538	0.3149
CV-273	0.6945	0.4149	0.8398	0.6273	0.5735	0.7343	0.2800
CV-274	0.6836	0.4787	0.7901	0.6344	0.5422	0.7448	0.2777
CV-275	0.6982	0.4681	0.8177	0.6429	0.5714	0.7475	0.3019
CV-276	0.6982	0.4574	0.8232	0.6403	0.5733	0.7450	0.2989
CV-277	0.7127	0.4681	0.8398	0.6539	0.6027	0.7525	0.3307
CV-278	0.6836	0.4149	0.8232	0.6190	0.5493	0.7304	0.2581

CV-279	0.6509	0.4043	0.7790	0.5916	0.4872	0.7157	0.1928
CV-280	0.6873	0.4574	0.8066	0.6320	0.5513	0.7411	0.2779
CV-281	0.7018	0.4468	0.8343	0.6405	0.5833	0.7438	0.3032
CV-282	0.6909	0.4787	0.8011	0.6399	0.5556	0.7474	0.2912
CV-283	0.6909	0.4468	0.8177	0.6322	0.5600	0.7400	0.2817
CV-284	0.7018	0.4255	0.8453	0.6354	0.5882	0.7391	0.2978
CV-285	0.6691	0.4043	0.8066	0.6054	0.5205	0.7228	0.2265
CV-286	0.6836	0.4362	0.8122	0.6242	0.5467	0.7350	0.2645
CV-287	0.6945	0.4362	0.8287	0.6324	0.5694	0.7389	0.2858
CV-288	0.6982	0.4787	0.8122	0.6454	0.5696	0.7500	0.3049
CV-289	0.6727	0.4681	0.7790	0.6235	0.5238	0.7382	0.2545
CV-290	0.7091	0.4894	0.8232	0.6563	0.5897	0.7563	0.3289
CV-291	0.6873	0.4574	0.8066	0.6320	0.5513	0.7411	0.2779
CV-292	0.6727	0.4362	0.7956	0.6159	0.5256	0.7310	0.2439
CV-293	0.6800	0.4574	0.7956	0.6265	0.5375	0.7385	0.2642
CV-294	0.7200	0.4787	0.8453	0.6620	0.6164	0.7574	0.3481
CV-295	0.6800	0.4468	0.8011	0.6240	0.5385	0.7360	0.2609
CV-296	0.6727	0.4043	0.8122	0.6082	0.5278	0.7241	0.2335
CV-297	0.7164	0.5106	0.8232	0.6669	0.6000	0.7641	0.3486
CV-298	0.6618	0.4362	0.7790	0.6076	0.5062	0.7268	0.2239
CV-299	0.6727	0.4255	0.8011	0.6133	0.5263	0.7286	0.2404
CV-300	0.6727	0.4149	0.8066	0.6108	0.5270	0.7264	0.2369
CV-301	0.6945	0.4574	0.8177	0.6376	0.5658	0.7437	0.2918
CV-302	0.7055	0.4787	0.8232	0.6510	0.5844	0.7525	0.3190
CV-303	0.6800	0.4149	0.8177	0.6163	0.5417	0.7291	0.2509
CV-304	0.6800	0.4362	0.8066	0.6214	0.5395	0.7337	0.2575
CV-305	0.6982	0.4255	0.8398	0.6327	0.5797	0.7379	0.2903
CV-306	0.6655	0.4043	0.8011	0.6027	0.5135	0.7214	0.2196
CV-307	0.6764	0.4255	0.8066	0.6161	0.5333	0.7300	0.2473
CV-308	0.7091	0.4681	0.8343	0.6512	0.5946	0.7512	0.3234
CV-309	0.6909	0.4681	0.8066	0.6374	0.5570	0.7449	0.2880
CV-310	0.6909	0.4149	0.8343	0.6246	0.5652	0.7330	0.2726
CV-311	0.6545	0.3936	0.7901	0.5918	0.4933	0.7150	0.1956
CV-312	0.6945	0.4787	0.8066	0.6427	0.5625	0.7487	0.2980
CV-313	0.7018	0.4468	0.8343	0.6405	0.5833	0.7438	0.3032
CV-314	0.6691	0.4043	0.8066	0.6054	0.5205	0.7228	0.2265
CV-315	0.6982	0.4681	0.8177	0.6429	0.5714	0.7475	0.3019
CV-316	0.6945	0.4681	0.8122	0.6401	0.5641	0.7462	0.2949
CV-317	0.7091	0.4362	0.8508	0.6435	0.6029	0.7440	0.3155
CV-318	0.7055	0.4468	0.8398	0.6433	0.5915	0.7451	0.3106
CV-319	0.6509	0.3830	0.7901	0.5865	0.4865	0.7114	0.1851
CV-320	0.6945	0.4255	0.8343	0.6299	0.5714	0.7366	0.2829
CV-321	0.6873	0.4894	0.7901	0.6397	0.5476	0.7487	0.2877
CV-322	0.7127	0.4787	0.8343	0.6565	0.6000	0.7550	0.3333
CV-323	0.7018	0.4574	0.8287	0.6431	0.5811	0.7463	0.3061
CV-324	0.6873	0.4255	0.8232	0.6244	0.5556	0.7340	0.2684
CV-325	0.6727	0.3936	0.8177	0.6056	0.5286	0.7220	0.2301
CV-326	0.6909	0.4468	0.8177	0.6322	0.5600	0.7400	0.2817
CV-327	0.6655	0.4574	0.7735	0.6155	0.5119	0.7330	0.2378
CV-328	0.6873	0.3723	0.8508	0.6116	0.5645	0.7230	0.2533
CV-329	0.6800	0.4468	0.8011	0.6240	0.5385	0.7360	0.2609
CV-330	0.6982	0.4574	0.8232	0.6403	0.5733	0.7450	0.2989
CV-331	0.6909	0.4468	0.8177	0.6322	0.5600	0.7400	0.2817
CV-332	0.7345	0.5213	0.8453	0.6833	0.6364	0.7727	0.3873
CV-333	0.7200	0.4468	0.8619	0.6543	0.6269	0.7500	0.3411
CV-334	0.6764	0.4468	0.7956	0.6212	0.5316	0.7347	0.2541
CV-335	0.7018	0.4149	0.8508	0.6329	0.5909	0.7368	0.2951

CV-336	0.6727	0.4362	0.7956	0.6159	0.5256	0.7310	0.2439
CV-337	0.6655	0.4255	0.7901	0.6078	0.5128	0.7259	0.2269
CV-338	0.6800	0.4043	0.8232	0.6137	0.5429	0.7268	0.2477
CV-339	0.6909	0.4574	0.8122	0.6348	0.5584	0.7424	0.2848
CV-340	0.6873	0.4787	0.7956	0.6372	0.5488	0.7461	0.2844
CV-341	0.6691	0.4043	0.8066	0.6054	0.5205	0.7228	0.2265
CV-342	0.6909	0.4681	0.8066	0.6374	0.5570	0.7449	0.2880
CV-343	0.6836	0.4362	0.8122	0.6242	0.5467	0.7350	0.2645
CV-344	0.6691	0.4468	0.7845	0.6157	0.5185	0.7320	0.2407
CV-345	0.6800	0.4362	0.8066	0.6214	0.5395	0.7337	0.2575
CV-346	0.7309	0.4894	0.8564	0.6729	0.6389	0.7635	0.3730
CV-347	0.7055	0.5000	0.8122	0.6561	0.5802	0.7577	0.3248
CV-348	0.7091	0.4468	0.8453	0.6461	0.6000	0.7463	0.3181
CV-349	0.6436	0.3830	0.7790	0.5810	0.4737	0.7085	0.1718
CV-350	0.6400	0.3830	0.7735	0.5782	0.4675	0.7071	0.1653
CV-351	0.6909	0.4255	0.8287	0.6271	0.5634	0.7353	0.2756
CV-352	0.6764	0.4149	0.8122	0.6135	0.5342	0.7277	0.2439
CV-353	0.6945	0.4362	0.8287	0.6324	0.5694	0.7389	0.2858
CV-354	0.6982	0.4787	0.8122	0.6454	0.5696	0.7500	0.3049
CV-355	0.6945	0.4255	0.8343	0.6299	0.5714	0.7366	0.2829
CV-356	0.6909	0.4149	0.8343	0.6246	0.5652	0.7330	0.2726
CV-357	0.6764	0.3723	0.8343	0.6033	0.5385	0.7190	0.2307
CV-358	0.6727	0.4362	0.7956	0.6159	0.5256	0.7310	0.2439
CV-359	0.6982	0.4787	0.8122	0.6454	0.5696	0.7500	0.3049
CV-360	0.6727	0.4255	0.8011	0.6133	0.5263	0.7286	0.2404
CV-361	0.7127	0.4468	0.8508	0.6488	0.6087	0.7476	0.3256
CV-362	0.6836	0.4255	0.8177	0.6216	0.5479	0.7327	0.2612
CV-363	0.6945	0.4362	0.8287	0.6324	0.5694	0.7389	0.2858
CV-364	0.6691	0.4468	0.7845	0.6157	0.5185	0.7320	0.2407
CV-365	0.6800	0.4255	0.8122	0.6188	0.5405	0.7313	0.2542
CV-366	0.6982	0.4362	0.8343	0.6352	0.5775	0.7402	0.2931
CV-367	0.6691	0.4149	0.8011	0.6080	0.5200	0.7250	0.2300
CV-368	0.7127	0.4574	0.8453	0.6514	0.6056	0.7500	0.3281
CV-369	0.6764	0.4787	0.7790	0.6289	0.5294	0.7421	0.2645
CV-370	0.7018	0.4681	0.8232	0.6456	0.5789	0.7487	0.3090
CV-371	0.6982	0.4255	0.8398	0.6327	0.5797	0.7379	0.2903
CV-372	0.6909	0.4574	0.8122	0.6348	0.5584	0.7424	0.2848
CV-373	0.6800	0.3723	0.8398	0.6061	0.5469	0.7204	0.2381
CV-374	0.6800	0.4468	0.8011	0.6240	0.5385	0.7360	0.2609
CV-375	0.6800	0.4043	0.8232	0.6137	0.5429	0.7268	0.2477
CV-376	0.6836	0.4149	0.8232	0.6190	0.5493	0.7304	0.2581
CV-377	0.7018	0.4362	0.8398	0.6380	0.5857	0.7415	0.3005
CV-378	0.6982	0.4574	0.8232	0.6403	0.5733	0.7450	0.2989
CV-379	0.6873	0.4149	0.8287	0.6218	0.5571	0.7317	0.2653
CV-380	0.7127	0.4681	0.8398	0.6539	0.6027	0.7525	0.3307
CV-381	0.7018	0.4362	0.8398	0.6380	0.5857	0.7415	0.3005
CV-382	0.6982	0.4787	0.8122	0.6454	0.5696	0.7500	0.3049
CV-383	0.7055	0.4681	0.8287	0.6484	0.5867	0.7500	0.3161
CV-384	0.6873	0.4255	0.8232	0.6244	0.5556	0.7340	0.2684
CV-385	0.6800	0.4468	0.8011	0.6240	0.5385	0.7360	0.2609
CV-386	0.6982	0.4681	0.8177	0.6429	0.5714	0.7475	0.3019
CV-387	0.6545	0.4574	0.7569	0.6072	0.4943	0.7287	0.2186
CV-388	0.6836	0.4362	0.8122	0.6242	0.5467	0.7350	0.2645
CV-389	0.7273	0.4894	0.8508	0.6701	0.6301	0.7624	0.3654
CV-390	0.7091	0.4574	0.8398	0.6486	0.5972	0.7488	0.3207
CV-391	0.6873	0.4255	0.8232	0.6244	0.5556	0.7340	0.2684
CV-392	0.6655	0.3830	0.8122	0.5976	0.5143	0.7171	0.2125

CV-393	0.6691	0.3936	0.8122	0.6029	0.5211	0.7206	0.2230
CV-394	0.7091	0.4894	0.8232	0.6563	0.5897	0.7563	0.3289
CV-395	0.6982	0.4574	0.8232	0.6403	0.5733	0.7450	0.2989
CV-396	0.6727	0.3936	0.8177	0.6056	0.5286	0.7220	0.2301
CV-397	0.6727	0.4255	0.8011	0.6133	0.5263	0.7286	0.2404
CV-398	0.6764	0.4681	0.7845	0.6263	0.5301	0.7396	0.2610
CV-399	0.7345	0.4574	0.8785	0.6679	0.6615	0.7571	0.3750
CV-400	0.6982	0.4149	0.8453	0.6301	0.5821	0.7356	0.2875
CV-401	0.6982	0.4681	0.8177	0.6429	0.5714	0.7475	0.3019
CV-402	0.6800	0.4362	0.8066	0.6214	0.5395	0.7337	0.2575
CV-403	0.6800	0.4255	0.8122	0.6188	0.5405	0.7313	0.2542
CV-404	0.6945	0.4681	0.8122	0.6401	0.5641	0.7462	0.2949
CV-405	0.6764	0.4362	0.8011	0.6186	0.5325	0.7323	0.2507
CV-406	0.6982	0.4574	0.8232	0.6403	0.5733	0.7450	0.2989
CV-407	0.6909	0.4043	0.8398	0.6220	0.5672	0.7308	0.2696
CV-408	0.6727	0.3723	0.8287	0.6005	0.5303	0.7177	0.2233
CV-409	0.6800	0.4255	0.8122	0.6188	0.5405	0.7313	0.2542
CV-410	0.7018	0.4574	0.8287	0.6431	0.5811	0.7463	0.3061
CV-411	0.6691	0.4362	0.7901	0.6131	0.5190	0.7296	0.2371
CV-412	0.6727	0.4787	0.7735	0.6261	0.5233	0.7407	0.2580
CV-413	0.6764	0.4362	0.8011	0.6186	0.5325	0.7323	0.2507
CV-414	0.7018	0.4468	0.8343	0.6405	0.5833	0.7438	0.3032
CV-415	0.6582	0.4362	0.7735	0.6048	0.5000	0.7254	0.2174
CV-416	0.6764	0.4255	0.8066	0.6161	0.5333	0.7300	0.2473
CV-417	0.6545	0.4255	0.7735	0.5995	0.4938	0.7216	0.2071
CV-418	0.6945	0.4468	0.8232	0.6350	0.5676	0.7413	0.2888
CV-419	0.7055	0.4894	0.8177	0.6535	0.5823	0.7551	0.3219
CV-420	0.6800	0.3936	0.8287	0.6112	0.5441	0.7246	0.2445
CV-421	0.6727	0.4362	0.7956	0.6159	0.5256	0.7310	0.2439
CV-422	0.6618	0.4255	0.7845	0.6050	0.5063	0.7245	0.2202
CV-423	0.6800	0.4362	0.8066	0.6214	0.5395	0.7337	0.2575
CV-424	0.6582	0.4043	0.7901	0.5972	0.5000	0.7186	0.2061
CV-425	0.6873	0.4255	0.8232	0.6244	0.5556	0.7340	0.2684
CV-426	0.6873	0.4255	0.8232	0.6244	0.5556	0.7340	0.2684
CV-427	0.6873	0.4362	0.8177	0.6269	0.5541	0.7363	0.2715
CV-428	0.6618	0.4362	0.7790	0.6076	0.5062	0.7268	0.2239
CV-429	0.7018	0.4787	0.8177	0.6482	0.5769	0.7513	0.3119
CV-430	0.6873	0.3936	0.8398	0.6167	0.5606	0.7273	0.2592
CV-431	0.6800	0.4681	0.7901	0.6291	0.5366	0.7409	0.2677
CV-432	0.6691	0.4149	0.8011	0.6080	0.5200	0.7250	0.2300
CV-433	0.6909	0.4574	0.8122	0.6348	0.5584	0.7424	0.2848
CV-434	0.6727	0.4787	0.7735	0.6261	0.5233	0.7407	0.2580
CV-435	0.6836	0.4574	0.8011	0.6293	0.5443	0.7398	0.2710
CV-436	0.6800	0.4149	0.8177	0.6163	0.5417	0.7291	0.2509
CV-437	0.6691	0.4362	0.7901	0.6131	0.5190	0.7296	0.2371
CV-438	0.6800	0.4362	0.8066	0.6214	0.5395	0.7337	0.2575
CV-439	0.6982	0.4681	0.8177	0.6429	0.5714	0.7475	0.3019
CV-440	0.7018	0.4787	0.8177	0.6482	0.5769	0.7513	0.3119
CV-441	0.6764	0.4574	0.7901	0.6238	0.5309	0.7371	0.2575
CV-442	0.6982	0.4362	0.8343	0.6352	0.5775	0.7402	0.2931
CV-443	0.6545	0.3936	0.7901	0.5918	0.4933	0.7150	0.1956
CV-444	0.6618	0.4255	0.7845	0.6050	0.5063	0.7245	0.2202
CV-445	0.6727	0.4681	0.7790	0.6235	0.5238	0.7382	0.2545
CV-446	0.6909	0.4574	0.8122	0.6348	0.5584	0.7424	0.2848
CV-447	0.6655	0.4468	0.7790	0.6129	0.5122	0.7306	0.2341
CV-448	0.7091	0.4681	0.8343	0.6512	0.5946	0.7512	0.3234
CV-449	0.6582	0.4255	0.7790	0.6023	0.5000	0.7231	0.2136

CV-450	0.6836	0.4362	0.8122	0.6242	0.5467	0.7350	0.2645
CV-451	0.7055	0.4468	0.8398	0.6433	0.5915	0.7451	0.3106
CV-452	0.6727	0.4149	0.8066	0.6108	0.5270	0.7264	0.2369
CV-453	0.6873	0.4574	0.8066	0.6320	0.5513	0.7411	0.2779
CV-454	0.6545	0.4149	0.7790	0.5969	0.4937	0.7194	0.2033
CV-455	0.6727	0.4255	0.8011	0.6133	0.5263	0.7286	0.2404
CV-456	0.6764	0.4681	0.7845	0.6263	0.5301	0.7396	0.2610
CV-457	0.6945	0.4149	0.8398	0.6273	0.5735	0.7343	0.2800
CV-458	0.6655	0.3723	0.8177	0.5950	0.5147	0.7150	0.2089
CV-459	0.6945	0.4255	0.8343	0.6299	0.5714	0.7366	0.2829
CV-460	0.6727	0.4149	0.8066	0.6108	0.5270	0.7264	0.2369
CV-461	0.7018	0.4681	0.8232	0.6456	0.5789	0.7487	0.3090
CV-462	0.6691	0.4255	0.7956	0.6106	0.5195	0.7273	0.2336
CV-463	0.6691	0.4787	0.7680	0.6233	0.5172	0.7394	0.2516
CV-464	0.6800	0.4255	0.8122	0.6188	0.5405	0.7313	0.2542
CV-465	0.7055	0.4681	0.8287	0.6484	0.5867	0.7500	0.3161
CV-466	0.6509	0.4255	0.7680	0.5967	0.4878	0.7202	0.2006
CV-467	0.6691	0.4043	0.8066	0.6054	0.5205	0.7228	0.2265
CV-468	0.6909	0.4574	0.8122	0.6348	0.5584	0.7424	0.2848
CV-469	0.6764	0.4255	0.8066	0.6161	0.5333	0.7300	0.2473
CV-470	0.6873	0.4681	0.8011	0.6346	0.5500	0.7436	0.2811
CV-471	0.6727	0.4574	0.7845	0.6210	0.5244	0.7358	0.2509
CV-472	0.6873	0.4149	0.8287	0.6218	0.5571	0.7317	0.2653
CV-473	0.6800	0.4362	0.8066	0.6214	0.5395	0.7337	0.2575
CV-474	0.6764	0.4468	0.7956	0.6212	0.5316	0.7347	0.2541
CV-475	0.6909	0.4681	0.8066	0.6374	0.5570	0.7449	0.2880
CV-476	0.6800	0.4468	0.8011	0.6240	0.5385	0.7360	0.2609
CV-477	0.6800	0.4468	0.8011	0.6240	0.5385	0.7360	0.2609
CV-478	0.6873	0.4255	0.8232	0.6244	0.5556	0.7340	0.2684
CV-479	0.7055	0.4681	0.8287	0.6484	0.5867	0.7500	0.3161
CV-480	0.6945	0.4255	0.8343	0.6299	0.5714	0.7366	0.2829
CV-481	0.7164	0.4894	0.8343	0.6618	0.6053	0.7588	0.3432
CV-482	0.6436	0.4043	0.7680	0.5861	0.4750	0.7128	0.1798
CV-483	0.6291	0.3830	0.7569	0.5699	0.4500	0.7026	0.1461
CV-484	0.6982	0.4574	0.8232	0.6403	0.5733	0.7450	0.2989
CV-485	0.6655	0.4043	0.8011	0.6027	0.5135	0.7214	0.2196
CV-486	0.6655	0.3936	0.8066	0.6001	0.5139	0.7192	0.2161
CV-487	0.7018	0.4574	0.8287	0.6431	0.5811	0.7463	0.3061
CV-488	0.6691	0.4255	0.7956	0.6106	0.5195	0.7273	0.2336
CV-489	0.6909	0.4681	0.8066	0.6374	0.5570	0.7449	0.2880
CV-490	0.6655	0.4149	0.7956	0.6052	0.5132	0.7236	0.2232
CV-491	0.7164	0.4681	0.8453	0.6567	0.6111	0.7537	0.3381
CV-492	0.7273	0.4894	0.8508	0.6701	0.6301	0.7624	0.3654
CV-493	0.6909	0.4468	0.8177	0.6322	0.5600	0.7400	0.2817
CV-494	0.6873	0.4787	0.7956	0.6372	0.5488	0.7461	0.2844
CV-495	0.7091	0.4468	0.8453	0.6461	0.6000	0.7463	0.3181
CV-496	0.6800	0.4468	0.8011	0.6240	0.5385	0.7360	0.2609
CV-497	0.6909	0.4362	0.8232	0.6297	0.5616	0.7376	0.2786
CV-498	0.6691	0.3617	0.8287	0.5952	0.5231	0.7143	0.2126
CV-499	0.6691	0.4149	0.8011	0.6080	0.5200	0.7250	0.2300
CV-500	0.6909	0.4681	0.8066	0.6374	0.5570	0.7449	0.2880

Table S11. Performance of 5-fold cross-validations from consensus modeling (Cons). Performance of 5-fold cross-validations were evaluated by seven performance metrics. Each row shows the performance of one time 5-fold cross-validation. 5-fold cross-validation was repeated 500 times (CV-1 ~ CV-500).

Cross-validation	Accuracy	Sensitivity	Specificity	Balanced accuracy	Positive prediction rate	Negative prediction rate	Matthews correlation coefficient
CV-1	0.7345	0.5426	0.8343	0.6884	0.6296	0.7784	0.3921
CV-2	0.6909	0.4574	0.8122	0.6348	0.5584	0.7424	0.2848
CV-3	0.7345	0.5426	0.8343	0.6884	0.6296	0.7784	0.3921
CV-4	0.6982	0.5106	0.7956	0.6531	0.5647	0.7579	0.3143
CV-5	0.7055	0.4787	0.8232	0.6510	0.5844	0.7525	0.3190
CV-6	0.6982	0.5106	0.7956	0.6531	0.5647	0.7579	0.3143
CV-7	0.6909	0.4787	0.8011	0.6399	0.5556	0.7474	0.2912
CV-8	0.6764	0.4468	0.7956	0.6212	0.5316	0.7347	0.2541
CV-9	0.7018	0.4574	0.8287	0.6431	0.5811	0.7463	0.3061
CV-10	0.7018	0.4574	0.8287	0.6431	0.5811	0.7463	0.3061
CV-11	0.7164	0.5000	0.8287	0.6644	0.6026	0.7614	0.3459
CV-12	0.6691	0.4362	0.7901	0.6131	0.5190	0.7296	0.2371
CV-13	0.6800	0.4681	0.7901	0.6291	0.5366	0.7409	0.2677
CV-14	0.7164	0.4787	0.8398	0.6593	0.6081	0.7562	0.3406
CV-15	0.6836	0.4468	0.8066	0.6267	0.5455	0.7374	0.2677
CV-16	0.7127	0.4787	0.8343	0.6565	0.6000	0.7550	0.3333
CV-17	0.7309	0.5000	0.8508	0.6754	0.6351	0.7662	0.3752
CV-18	0.6945	0.4149	0.8398	0.6273	0.5735	0.7343	0.2800
CV-19	0.7127	0.4894	0.8287	0.6590	0.5974	0.7576	0.3360
CV-20	0.7236	0.4894	0.8453	0.6673	0.6216	0.7612	0.3579
CV-21	0.7055	0.4894	0.8177	0.6535	0.5823	0.7551	0.3219
CV-22	0.7018	0.4787	0.8177	0.6482	0.5769	0.7513	0.3119
CV-23	0.7127	0.4787	0.8343	0.6565	0.6000	0.7550	0.3333
CV-24	0.7200	0.4681	0.8508	0.6595	0.6197	0.7549	0.3456
CV-25	0.6909	0.4894	0.7956	0.6425	0.5542	0.7500	0.2944
CV-26	0.7055	0.4894	0.8177	0.6535	0.5823	0.7551	0.3219
CV-27	0.7018	0.4681	0.8232	0.6456	0.5789	0.7487	0.3090
CV-28	0.7127	0.4681	0.8398	0.6539	0.6027	0.7525	0.3307
CV-29	0.7091	0.4787	0.8287	0.6537	0.5921	0.7538	0.3261
CV-30	0.7091	0.5213	0.8066	0.6640	0.5833	0.7644	0.3377
CV-31	0.7055	0.4787	0.8232	0.6510	0.5844	0.7525	0.3190
CV-32	0.7091	0.4894	0.8232	0.6563	0.5897	0.7563	0.3289
CV-33	0.7127	0.5106	0.8177	0.6642	0.5926	0.7629	0.3416
CV-34	0.6873	0.4787	0.7956	0.6372	0.5488	0.7461	0.2844
CV-35	0.7164	0.5000	0.8287	0.6644	0.6026	0.7614	0.3459
CV-36	0.7091	0.4681	0.8343	0.6512	0.5946	0.7512	0.3234
CV-37	0.7091	0.5000	0.8177	0.6588	0.5875	0.7590	0.3318
CV-38	0.7236	0.5319	0.8232	0.6776	0.6098	0.7720	0.3682
CV-39	0.6982	0.4787	0.8122	0.6454	0.5696	0.7500	0.3049
CV-40	0.6982	0.5106	0.7956	0.6531	0.5647	0.7579	0.3143
CV-41	0.6982	0.4468	0.8287	0.6378	0.5753	0.7426	0.2960
CV-42	0.7055	0.4787	0.8232	0.6510	0.5844	0.7525	0.3190
CV-43	0.7455	0.5426	0.8508	0.6967	0.6538	0.7817	0.4139
CV-44	0.7164	0.5000	0.8287	0.6644	0.6026	0.7614	0.3459
CV-45	0.6982	0.4468	0.8287	0.6378	0.5753	0.7426	0.2960
CV-46	0.7091	0.4787	0.8287	0.6537	0.5921	0.7538	0.3261
CV-47	0.6982	0.5106	0.7956	0.6531	0.5647	0.7579	0.3143
CV-48	0.6945	0.4681	0.8122	0.6401	0.5641	0.7462	0.2949
CV-49	0.6764	0.4468	0.7956	0.6212	0.5316	0.7347	0.2541
CV-50	0.7091	0.5000	0.8177	0.6588	0.5875	0.7590	0.3318

CV-51	0.7200	0.4681	0.8508	0.6595	0.6197	0.7549	0.3456
CV-52	0.6945	0.4894	0.8011	0.6452	0.5610	0.7513	0.3012
CV-53	0.7309	0.5319	0.8343	0.6831	0.6250	0.7744	0.3824
CV-54	0.6873	0.5000	0.7845	0.6423	0.5465	0.7513	0.2911
CV-55	0.6836	0.4787	0.7901	0.6344	0.5422	0.7448	0.2777
CV-56	0.6982	0.4894	0.8066	0.6480	0.5679	0.7526	0.3080
CV-57	0.7200	0.4787	0.8453	0.6620	0.6164	0.7574	0.3481
CV-58	0.7055	0.4681	0.8287	0.6484	0.5867	0.7500	0.3161
CV-59	0.7273	0.5106	0.8398	0.6752	0.6234	0.7677	0.3702
CV-60	0.7018	0.5000	0.8066	0.6533	0.5732	0.7565	0.3179
CV-61	0.7091	0.4681	0.8343	0.6512	0.5946	0.7512	0.3234
CV-62	0.6873	0.4681	0.8011	0.6346	0.5500	0.7436	0.2811
CV-63	0.6909	0.4894	0.7956	0.6425	0.5542	0.7500	0.2944
CV-64	0.7055	0.5000	0.8122	0.6561	0.5802	0.7577	0.3248
CV-65	0.7055	0.4787	0.8232	0.6510	0.5844	0.7525	0.3190
CV-66	0.7091	0.4574	0.8398	0.6486	0.5972	0.7488	0.3207
CV-67	0.6982	0.4362	0.8343	0.6352	0.5775	0.7402	0.2931
CV-68	0.7164	0.5000	0.8287	0.6644	0.6026	0.7614	0.3459
CV-69	0.7055	0.4681	0.8287	0.6484	0.5867	0.7500	0.3161
CV-70	0.7164	0.4681	0.8453	0.6567	0.6111	0.7537	0.3381
CV-71	0.7345	0.5000	0.8564	0.6782	0.6438	0.7673	0.3828
CV-72	0.6873	0.4787	0.7956	0.6372	0.5488	0.7461	0.2844
CV-73	0.6764	0.4574	0.7901	0.6238	0.5309	0.7371	0.2575
CV-74	0.7273	0.5426	0.8232	0.6829	0.6145	0.7760	0.3779
CV-75	0.7091	0.5000	0.8177	0.6588	0.5875	0.7590	0.3318
CV-76	0.7055	0.4894	0.8177	0.6535	0.5823	0.7551	0.3219
CV-77	0.7055	0.5106	0.8066	0.6586	0.5783	0.7604	0.3278
CV-78	0.7018	0.4787	0.8177	0.6482	0.5769	0.7513	0.3119
CV-79	0.7164	0.5106	0.8232	0.6669	0.6000	0.7641	0.3486
CV-80	0.7273	0.4787	0.8564	0.6675	0.6338	0.7598	0.3632
CV-81	0.7091	0.5000	0.8177	0.6588	0.5875	0.7590	0.3318
CV-82	0.6800	0.4362	0.8066	0.6214	0.5395	0.7337	0.2575
CV-83	0.6800	0.4574	0.7956	0.6265	0.5375	0.7385	0.2642
CV-84	0.7127	0.4787	0.8343	0.6565	0.6000	0.7550	0.3333
CV-85	0.7091	0.4894	0.8232	0.6563	0.5897	0.7563	0.3289
CV-86	0.6764	0.4149	0.8122	0.6135	0.5342	0.7277	0.2439
CV-87	0.7127	0.5000	0.8232	0.6616	0.5949	0.7602	0.3388
CV-88	0.7127	0.4681	0.8398	0.6539	0.6027	0.7525	0.3307
CV-89	0.6655	0.4468	0.7790	0.6129	0.5122	0.7306	0.2341
CV-90	0.6800	0.4362	0.8066	0.6214	0.5395	0.7337	0.2575
CV-91	0.7091	0.4681	0.8343	0.6512	0.5946	0.7512	0.3234
CV-92	0.7018	0.4787	0.8177	0.6482	0.5769	0.7513	0.3119
CV-93	0.7236	0.5426	0.8177	0.6801	0.6071	0.7749	0.3710
CV-94	0.7236	0.4894	0.8453	0.6673	0.6216	0.7612	0.3579
CV-95	0.7091	0.5106	0.8122	0.6614	0.5854	0.7617	0.3347
CV-96	0.7127	0.5213	0.8122	0.6667	0.5904	0.7656	0.3445
CV-97	0.7127	0.4574	0.8453	0.6514	0.6056	0.7500	0.3281
CV-98	0.7055	0.4681	0.8287	0.6484	0.5867	0.7500	0.3161
CV-99	0.6982	0.4574	0.8232	0.6403	0.5733	0.7450	0.2989
CV-100	0.6873	0.4681	0.8011	0.6346	0.5500	0.7436	0.2811
CV-101	0.6909	0.5000	0.7901	0.6450	0.5529	0.7526	0.2977
CV-102	0.7309	0.5213	0.8398	0.6805	0.6282	0.7716	0.3799
CV-103	0.7273	0.4574	0.8674	0.6624	0.6418	0.7548	0.3589
CV-104	0.6764	0.4574	0.7901	0.6238	0.5309	0.7371	0.2575
CV-105	0.6691	0.4787	0.7680	0.6233	0.5172	0.7394	0.2516
CV-106	0.7055	0.4787	0.8232	0.6510	0.5844	0.7525	0.3190
CV-107	0.7091	0.5106	0.8122	0.6614	0.5854	0.7617	0.3347

CV-108	0.7164	0.4681	0.8453	0.6567	0.6111	0.7537	0.3381
CV-109	0.7055	0.4787	0.8232	0.6510	0.5844	0.7525	0.3190
CV-110	0.7164	0.4681	0.8453	0.6567	0.6111	0.7537	0.3381
CV-111	0.7018	0.4787	0.8177	0.6482	0.5769	0.7513	0.3119
CV-112	0.6836	0.4468	0.8066	0.6267	0.5455	0.7374	0.2677
CV-113	0.7382	0.5426	0.8398	0.6912	0.6375	0.7795	0.3993
CV-114	0.6982	0.4574	0.8232	0.6403	0.5733	0.7450	0.2989
CV-115	0.6873	0.5000	0.7845	0.6423	0.5465	0.7513	0.2911
CV-116	0.6945	0.4894	0.8011	0.6452	0.5610	0.7513	0.3012
CV-117	0.7200	0.5106	0.8287	0.6697	0.6076	0.7653	0.3557
CV-118	0.6909	0.4681	0.8066	0.6374	0.5570	0.7449	0.2880
CV-119	0.6909	0.5106	0.7845	0.6476	0.5517	0.7553	0.3010
CV-120	0.6945	0.4787	0.8066	0.6427	0.5625	0.7487	0.2980
CV-121	0.7018	0.4681	0.8232	0.6456	0.5789	0.7487	0.3090
CV-122	0.6909	0.4681	0.8066	0.6374	0.5570	0.7449	0.2880
CV-123	0.6727	0.4255	0.8011	0.6133	0.5263	0.7286	0.2404
CV-124	0.6873	0.4468	0.8122	0.6295	0.5526	0.7387	0.2747
CV-125	0.6945	0.4681	0.8122	0.6401	0.5641	0.7462	0.2949
CV-126	0.6836	0.4681	0.7956	0.6318	0.5432	0.7423	0.2744
CV-127	0.6836	0.4681	0.7956	0.6318	0.5432	0.7423	0.2744
CV-128	0.7164	0.5000	0.8287	0.6644	0.6026	0.7614	0.3459
CV-129	0.6873	0.5000	0.7845	0.6423	0.5465	0.7513	0.2911
CV-130	0.6945	0.4681	0.8122	0.6401	0.5641	0.7462	0.2949
CV-131	0.7382	0.5638	0.8287	0.6963	0.6310	0.7853	0.4043
CV-132	0.7164	0.4681	0.8453	0.6567	0.6111	0.7537	0.3381
CV-133	0.7055	0.4681	0.8287	0.6484	0.5867	0.7500	0.3161
CV-134	0.7055	0.4787	0.8232	0.6510	0.5844	0.7525	0.3190
CV-135	0.7309	0.4362	0.8840	0.6601	0.6613	0.7512	0.3634
CV-136	0.7309	0.5106	0.8453	0.6780	0.6316	0.7688	0.3775
CV-137	0.6982	0.4574	0.8232	0.6403	0.5733	0.7450	0.2989
CV-138	0.6727	0.4681	0.7790	0.6235	0.5238	0.7382	0.2545
CV-139	0.6836	0.4468	0.8066	0.6267	0.5455	0.7374	0.2677
CV-140	0.7055	0.4574	0.8343	0.6459	0.5890	0.7475	0.3133
CV-141	0.7273	0.5213	0.8343	0.6778	0.6203	0.7704	0.3727
CV-142	0.7127	0.5213	0.8122	0.6667	0.5904	0.7656	0.3445
CV-143	0.7127	0.4681	0.8398	0.6539	0.6027	0.7525	0.3307
CV-144	0.6800	0.4362	0.8066	0.6214	0.5395	0.7337	0.2575
CV-145	0.6945	0.4468	0.8232	0.6350	0.5676	0.7413	0.2888
CV-146	0.7127	0.4574	0.8453	0.6514	0.6056	0.7500	0.3281
CV-147	0.6800	0.4149	0.8177	0.6163	0.5417	0.7291	0.2509
CV-148	0.6982	0.4362	0.8343	0.6352	0.5775	0.7402	0.2931
CV-149	0.7018	0.4787	0.8177	0.6482	0.5769	0.7513	0.3119
CV-150	0.7091	0.4787	0.8287	0.6537	0.5921	0.7538	0.3261
CV-151	0.7164	0.4894	0.8343	0.6618	0.6053	0.7588	0.3432
CV-152	0.7091	0.5000	0.8177	0.6588	0.5875	0.7590	0.3318
CV-153	0.7127	0.4894	0.8287	0.6590	0.5974	0.7576	0.3360
CV-154	0.7127	0.4681	0.8398	0.6539	0.6027	0.7525	0.3307
CV-155	0.6945	0.4574	0.8177	0.6376	0.5658	0.7437	0.2918
CV-156	0.6945	0.5213	0.7845	0.6529	0.5568	0.7594	0.3109
CV-157	0.7200	0.5319	0.8177	0.6748	0.6024	0.7708	0.3612
CV-158	0.7055	0.5106	0.8066	0.6586	0.5783	0.7604	0.3278
CV-159	0.6909	0.5106	0.7845	0.6476	0.5517	0.7553	0.3010
CV-160	0.6909	0.4468	0.8177	0.6322	0.5600	0.7400	0.2817
CV-161	0.6909	0.4468	0.8177	0.6322	0.5600	0.7400	0.2817
CV-162	0.7200	0.5000	0.8343	0.6671	0.6104	0.7626	0.3531
CV-163	0.6800	0.4149	0.8177	0.6163	0.5417	0.7291	0.2509
CV-164	0.6945	0.4787	0.8066	0.6427	0.5625	0.7487	0.2980

CV-165	0.6909	0.4787	0.8011	0.6399	0.5556	0.7474	0.2912
CV-166	0.6945	0.5000	0.7956	0.6478	0.5595	0.7539	0.3044
CV-167	0.6836	0.4787	0.7901	0.6344	0.5422	0.7448	0.2777
CV-168	0.7164	0.4894	0.8343	0.6618	0.6053	0.7588	0.3432
CV-169	0.6909	0.4681	0.8066	0.6374	0.5570	0.7449	0.2880
CV-170	0.7018	0.4574	0.8287	0.6431	0.5811	0.7463	0.3061
CV-171	0.7164	0.5426	0.8066	0.6746	0.5930	0.7725	0.3573
CV-172	0.7018	0.4894	0.8122	0.6508	0.5750	0.7538	0.3149
CV-173	0.7018	0.4468	0.8343	0.6405	0.5833	0.7438	0.3032
CV-174	0.7127	0.5319	0.8066	0.6693	0.5882	0.7684	0.3475
CV-175	0.6800	0.4468	0.8011	0.6240	0.5385	0.7360	0.2609
CV-176	0.7418	0.5213	0.8564	0.6888	0.6533	0.7750	0.4022
CV-177	0.7018	0.4468	0.8343	0.6405	0.5833	0.7438	0.3032
CV-178	0.7091	0.5000	0.8177	0.6588	0.5875	0.7590	0.3318
CV-179	0.7382	0.4894	0.8674	0.6784	0.6571	0.7659	0.3885
CV-180	0.6873	0.4681	0.8011	0.6346	0.5500	0.7436	0.2811
CV-181	0.6727	0.4574	0.7845	0.6210	0.5244	0.7358	0.2509
CV-182	0.7127	0.4894	0.8287	0.6590	0.5974	0.7576	0.3360
CV-183	0.6909	0.4574	0.8122	0.6348	0.5584	0.7424	0.2848
CV-184	0.6800	0.4574	0.7956	0.6265	0.5375	0.7385	0.2642
CV-185	0.6836	0.4362	0.8122	0.6242	0.5467	0.7350	0.2645
CV-186	0.7127	0.5000	0.8232	0.6616	0.5949	0.7602	0.3388
CV-187	0.7091	0.5000	0.8177	0.6588	0.5875	0.7590	0.3318
CV-188	0.7018	0.4787	0.8177	0.6482	0.5769	0.7513	0.3119
CV-189	0.6982	0.4787	0.8122	0.6454	0.5696	0.7500	0.3049
CV-190	0.7164	0.5532	0.8011	0.6771	0.5909	0.7754	0.3603
CV-191	0.6945	0.4681	0.8122	0.6401	0.5641	0.7462	0.2949
CV-192	0.6800	0.4255	0.8122	0.6188	0.5405	0.7313	0.2542
CV-193	0.6982	0.4787	0.8122	0.6454	0.5696	0.7500	0.3049
CV-194	0.6982	0.4681	0.8177	0.6429	0.5714	0.7475	0.3019
CV-195	0.6909	0.4574	0.8122	0.6348	0.5584	0.7424	0.2848
CV-196	0.7091	0.5106	0.8122	0.6614	0.5854	0.7617	0.3347
CV-197	0.6945	0.4681	0.8122	0.6401	0.5641	0.7462	0.2949
CV-198	0.7200	0.5213	0.8232	0.6722	0.6049	0.7680	0.3584
CV-199	0.6909	0.4787	0.8011	0.6399	0.5556	0.7474	0.2912
CV-200	0.6945	0.4894	0.8011	0.6452	0.5610	0.7513	0.3012
CV-201	0.6945	0.4787	0.8066	0.6427	0.5625	0.7487	0.2980
CV-202	0.7091	0.5106	0.8122	0.6614	0.5854	0.7617	0.3347
CV-203	0.6909	0.4681	0.8066	0.6374	0.5570	0.7449	0.2880
CV-204	0.6836	0.4894	0.7845	0.6369	0.5412	0.7474	0.2811
CV-205	0.6909	0.4787	0.8011	0.6399	0.5556	0.7474	0.2912
CV-206	0.7236	0.5106	0.8343	0.6724	0.6154	0.7665	0.3629
CV-207	0.7018	0.4468	0.8343	0.6405	0.5833	0.7438	0.3032
CV-208	0.6945	0.5106	0.7901	0.6503	0.5581	0.7566	0.3076
CV-209	0.7055	0.4468	0.8398	0.6433	0.5915	0.7451	0.3106
CV-210	0.6873	0.4574	0.8066	0.6320	0.5513	0.7411	0.2779
CV-211	0.6836	0.4574	0.8011	0.6293	0.5443	0.7398	0.2710
CV-212	0.7091	0.4681	0.8343	0.6512	0.5946	0.7512	0.3234
CV-213	0.6945	0.4787	0.8066	0.6427	0.5625	0.7487	0.2980
CV-214	0.7164	0.5000	0.8287	0.6644	0.6026	0.7614	0.3459
CV-215	0.6727	0.4468	0.7901	0.6184	0.5250	0.7333	0.2474
CV-216	0.6945	0.4787	0.8066	0.6427	0.5625	0.7487	0.2980
CV-217	0.6873	0.4574	0.8066	0.6320	0.5513	0.7411	0.2779
CV-218	0.6909	0.5000	0.7901	0.6450	0.5529	0.7526	0.2977
CV-219	0.7236	0.5000	0.8398	0.6699	0.6184	0.7638	0.3604
CV-220	0.6764	0.4362	0.8011	0.6186	0.5325	0.7323	0.2507
CV-221	0.7345	0.5213	0.8453	0.6833	0.6364	0.7727	0.3873

CV-222	0.7091	0.5000	0.8177	0.6588	0.5875	0.7590	0.3318
CV-223	0.7091	0.4574	0.8398	0.6486	0.5972	0.7488	0.3207
CV-224	0.7164	0.4894	0.8343	0.6618	0.6053	0.7588	0.3432
CV-225	0.6727	0.4787	0.7735	0.6261	0.5233	0.7407	0.2580
CV-226	0.6945	0.4787	0.8066	0.6427	0.5625	0.7487	0.2980
CV-227	0.6836	0.4681	0.7956	0.6318	0.5432	0.7423	0.2744
CV-228	0.6836	0.4681	0.7956	0.6318	0.5432	0.7423	0.2744
CV-229	0.6873	0.4681	0.8011	0.6346	0.5500	0.7436	0.2811
CV-230	0.6800	0.4149	0.8177	0.6163	0.5417	0.7291	0.2509
CV-231	0.7345	0.5213	0.8453	0.6833	0.6364	0.7727	0.3873
CV-232	0.7055	0.4894	0.8177	0.6535	0.5823	0.7551	0.3219
CV-233	0.7273	0.4894	0.8508	0.6701	0.6301	0.7624	0.3654
CV-234	0.7309	0.5106	0.8453	0.6780	0.6316	0.7688	0.3775
CV-235	0.7055	0.4894	0.8177	0.6535	0.5823	0.7551	0.3219
CV-236	0.7091	0.4894	0.8232	0.6563	0.5897	0.7563	0.3289
CV-237	0.6727	0.4681	0.7790	0.6235	0.5238	0.7382	0.2545
CV-238	0.6873	0.4894	0.7901	0.6397	0.5476	0.7487	0.2877
CV-239	0.6727	0.4681	0.7790	0.6235	0.5238	0.7382	0.2545
CV-240	0.6727	0.4468	0.7901	0.6184	0.5250	0.7333	0.2474
CV-241	0.7200	0.4681	0.8508	0.6595	0.6197	0.7549	0.3456
CV-242	0.7091	0.4787	0.8287	0.6537	0.5921	0.7538	0.3261
CV-243	0.6909	0.4894	0.7956	0.6425	0.5542	0.7500	0.2944
CV-244	0.7091	0.5000	0.8177	0.6588	0.5875	0.7590	0.3318
CV-245	0.7164	0.4787	0.8398	0.6593	0.6081	0.7562	0.3406
CV-246	0.6982	0.5106	0.7956	0.6531	0.5647	0.7579	0.3143
CV-247	0.6909	0.4574	0.8122	0.6348	0.5584	0.7424	0.2848
CV-248	0.7127	0.5319	0.8066	0.6693	0.5882	0.7684	0.3475
CV-249	0.7200	0.4574	0.8564	0.6569	0.6232	0.7524	0.3433
CV-250	0.7127	0.4894	0.8287	0.6590	0.5974	0.7576	0.3360
CV-251	0.7091	0.4894	0.8232	0.6563	0.5897	0.7563	0.3289
CV-252	0.6945	0.4681	0.8122	0.6401	0.5641	0.7462	0.2949
CV-253	0.7273	0.4787	0.8564	0.6675	0.6338	0.7598	0.3632
CV-254	0.7273	0.4574	0.8674	0.6624	0.6418	0.7548	0.3589
CV-255	0.6945	0.4468	0.8232	0.6350	0.5676	0.7413	0.2888
CV-256	0.7236	0.5000	0.8398	0.6699	0.6184	0.7638	0.3604
CV-257	0.7055	0.4681	0.8287	0.6484	0.5867	0.7500	0.3161
CV-258	0.7091	0.4681	0.8343	0.6512	0.5946	0.7512	0.3234
CV-259	0.7018	0.4787	0.8177	0.6482	0.5769	0.7513	0.3119
CV-260	0.7127	0.4894	0.8287	0.6590	0.5974	0.7576	0.3360
CV-261	0.7273	0.4894	0.8508	0.6701	0.6301	0.7624	0.3654
CV-262	0.6909	0.4468	0.8177	0.6322	0.5600	0.7400	0.2817
CV-263	0.6945	0.4468	0.8232	0.6350	0.5676	0.7413	0.2888
CV-264	0.7236	0.5106	0.8343	0.6724	0.6154	0.7665	0.3629
CV-265	0.7018	0.4362	0.8398	0.6380	0.5857	0.7415	0.3005
CV-266	0.7055	0.5106	0.8066	0.6586	0.5783	0.7604	0.3278
CV-267	0.7055	0.4787	0.8232	0.6510	0.5844	0.7525	0.3190
CV-268	0.7273	0.5213	0.8343	0.6778	0.6203	0.7704	0.3727
CV-269	0.7018	0.4787	0.8177	0.6482	0.5769	0.7513	0.3119
CV-270	0.6873	0.4574	0.8066	0.6320	0.5513	0.7411	0.2779
CV-271	0.7273	0.5000	0.8453	0.6727	0.6267	0.7650	0.3678
CV-272	0.6982	0.5106	0.7956	0.6531	0.5647	0.7579	0.3143
CV-273	0.7345	0.5000	0.8564	0.6782	0.6438	0.7673	0.3828
CV-274	0.7164	0.5426	0.8066	0.6746	0.5930	0.7725	0.3573
CV-275	0.7345	0.5426	0.8343	0.6884	0.6296	0.7784	0.3921
CV-276	0.7382	0.5106	0.8564	0.6835	0.6486	0.7711	0.3925
CV-277	0.7200	0.5000	0.8343	0.6671	0.6104	0.7626	0.3531
CV-278	0.7200	0.5000	0.8343	0.6671	0.6104	0.7626	0.3531

CV-279	0.6800	0.4681	0.7901	0.6291	0.5366	0.7409	0.2677
CV-280	0.7055	0.4787	0.8232	0.6510	0.5844	0.7525	0.3190
CV-281	0.7309	0.4894	0.8564	0.6729	0.6389	0.7635	0.3730
CV-282	0.7164	0.5000	0.8287	0.6644	0.6026	0.7614	0.3459
CV-283	0.6655	0.4255	0.7901	0.6078	0.5128	0.7259	0.2269
CV-284	0.7200	0.4681	0.8508	0.6595	0.6197	0.7549	0.3456
CV-285	0.7018	0.4894	0.8122	0.6508	0.5750	0.7538	0.3149
CV-286	0.7164	0.4894	0.8343	0.6618	0.6053	0.7588	0.3432
CV-287	0.6873	0.4574	0.8066	0.6320	0.5513	0.7411	0.2779
CV-288	0.7164	0.4574	0.8508	0.6541	0.6143	0.7512	0.3357
CV-289	0.6982	0.4681	0.8177	0.6429	0.5714	0.7475	0.3019
CV-290	0.7382	0.5106	0.8564	0.6835	0.6486	0.7711	0.3925
CV-291	0.7200	0.5106	0.8287	0.6697	0.6076	0.7653	0.3557
CV-292	0.7055	0.5000	0.8122	0.6561	0.5802	0.7577	0.3248
CV-293	0.7236	0.5319	0.8232	0.6776	0.6098	0.7720	0.3682
CV-294	0.7018	0.4894	0.8122	0.6508	0.5750	0.7538	0.3149
CV-295	0.7127	0.5426	0.8011	0.6718	0.5862	0.7713	0.3505
CV-296	0.6618	0.4149	0.7901	0.6025	0.5065	0.7222	0.2165
CV-297	0.7091	0.5319	0.8011	0.6665	0.5814	0.7672	0.3407
CV-298	0.7018	0.5106	0.8011	0.6559	0.5714	0.7592	0.3210
CV-299	0.6873	0.4574	0.8066	0.6320	0.5513	0.7411	0.2779
CV-300	0.6982	0.5000	0.8011	0.6506	0.5663	0.7552	0.3111
CV-301	0.6982	0.4681	0.8177	0.6429	0.5714	0.7475	0.3019
CV-302	0.7273	0.5000	0.8453	0.6727	0.6267	0.7650	0.3678
CV-303	0.7018	0.5213	0.7956	0.6584	0.5698	0.7619	0.3242
CV-304	0.6691	0.4468	0.7845	0.6157	0.5185	0.7320	0.2407
CV-305	0.7091	0.4681	0.8343	0.6512	0.5946	0.7512	0.3234
CV-306	0.7164	0.4894	0.8343	0.6618	0.6053	0.7588	0.3432
CV-307	0.7164	0.4681	0.8453	0.6567	0.6111	0.7537	0.3381
CV-308	0.7164	0.5106	0.8232	0.6669	0.6000	0.7641	0.3486
CV-309	0.7091	0.5000	0.8177	0.6588	0.5875	0.7590	0.3318
CV-310	0.6873	0.4255	0.8232	0.6244	0.5556	0.7340	0.2684
CV-311	0.6873	0.4574	0.8066	0.6320	0.5513	0.7411	0.2779
CV-312	0.6945	0.4787	0.8066	0.6427	0.5625	0.7487	0.2980
CV-313	0.7236	0.5213	0.8287	0.6750	0.6125	0.7692	0.3655
CV-314	0.6909	0.5000	0.7901	0.6450	0.5529	0.7526	0.2977
CV-315	0.7309	0.5106	0.8453	0.6780	0.6316	0.7688	0.3775
CV-316	0.7236	0.5106	0.8343	0.6724	0.6154	0.7665	0.3629
CV-317	0.7055	0.4681	0.8287	0.6484	0.5867	0.7500	0.3161
CV-318	0.7236	0.4894	0.8453	0.6673	0.6216	0.7612	0.3579
CV-319	0.6945	0.4362	0.8287	0.6324	0.5694	0.7389	0.2858
CV-320	0.6982	0.4681	0.8177	0.6429	0.5714	0.7475	0.3019
CV-321	0.7164	0.5319	0.8122	0.6720	0.5952	0.7696	0.3543
CV-322	0.7164	0.5000	0.8287	0.6644	0.6026	0.7614	0.3459
CV-323	0.7127	0.4681	0.8398	0.6539	0.6027	0.7525	0.3307
CV-324	0.7018	0.4681	0.8232	0.6456	0.5789	0.7487	0.3090
CV-325	0.6982	0.4681	0.8177	0.6429	0.5714	0.7475	0.3019
CV-326	0.7236	0.4894	0.8453	0.6673	0.6216	0.7612	0.3579
CV-327	0.6982	0.4894	0.8066	0.6480	0.5679	0.7526	0.3080
CV-328	0.6909	0.4362	0.8232	0.6297	0.5616	0.7376	0.2786
CV-329	0.7164	0.5000	0.8287	0.6644	0.6026	0.7614	0.3459
CV-330	0.7164	0.4894	0.8343	0.6618	0.6053	0.7588	0.3432
CV-331	0.7091	0.4787	0.8287	0.6537	0.5921	0.7538	0.3261
CV-332	0.7382	0.5213	0.8508	0.6861	0.6447	0.7739	0.3947
CV-333	0.7236	0.4574	0.8619	0.6597	0.6324	0.7536	0.3511
CV-334	0.7273	0.4894	0.8508	0.6701	0.6301	0.7624	0.3654
CV-335	0.6982	0.4362	0.8343	0.6352	0.5775	0.7402	0.2931

CV-336	0.7164	0.5000	0.8287	0.6644	0.6026	0.7614	0.3459
CV-337	0.7055	0.4787	0.8232	0.6510	0.5844	0.7525	0.3190
CV-338	0.6764	0.4681	0.7845	0.6263	0.5301	0.7396	0.2610
CV-339	0.6873	0.4787	0.7956	0.6372	0.5488	0.7461	0.2844
CV-340	0.6836	0.4894	0.7845	0.6369	0.5412	0.7474	0.2811
CV-341	0.7018	0.4681	0.8232	0.6456	0.5789	0.7487	0.3090
CV-342	0.7018	0.4574	0.8287	0.6431	0.5811	0.7463	0.3061
CV-343	0.7018	0.4362	0.8398	0.6380	0.5857	0.7415	0.3005
CV-344	0.7127	0.5106	0.8177	0.6642	0.5926	0.7629	0.3416
CV-345	0.6909	0.4681	0.8066	0.6374	0.5570	0.7449	0.2880
CV-346	0.7200	0.4894	0.8398	0.6646	0.6133	0.7600	0.3505
CV-347	0.7200	0.5213	0.8232	0.6722	0.6049	0.7680	0.3584
CV-348	0.7018	0.4787	0.8177	0.6482	0.5769	0.7513	0.3119
CV-349	0.6945	0.4681	0.8122	0.6401	0.5641	0.7462	0.2949
CV-350	0.6691	0.4468	0.7845	0.6157	0.5185	0.7320	0.2407
CV-351	0.7055	0.4681	0.8287	0.6484	0.5867	0.7500	0.3161
CV-352	0.6836	0.4255	0.8177	0.6216	0.5479	0.7327	0.2612
CV-353	0.7164	0.5213	0.8177	0.6695	0.5976	0.7668	0.3514
CV-354	0.7018	0.5000	0.8066	0.6533	0.5732	0.7565	0.3179
CV-355	0.7164	0.5106	0.8232	0.6669	0.6000	0.7641	0.3486
CV-356	0.7345	0.5106	0.8508	0.6807	0.6400	0.7700	0.3850
CV-357	0.7127	0.4681	0.8398	0.6539	0.6027	0.7525	0.3307
CV-358	0.6982	0.4787	0.8122	0.6454	0.5696	0.7500	0.3049
CV-359	0.7127	0.5000	0.8232	0.6616	0.5949	0.7602	0.3388
CV-360	0.7091	0.5106	0.8122	0.6614	0.5854	0.7617	0.3347
CV-361	0.7091	0.4787	0.8287	0.6537	0.5921	0.7538	0.3261
CV-362	0.7055	0.4681	0.8287	0.6484	0.5867	0.7500	0.3161
CV-363	0.7200	0.5319	0.8177	0.6748	0.6024	0.7708	0.3612
CV-364	0.6945	0.4681	0.8122	0.6401	0.5641	0.7462	0.2949
CV-365	0.6945	0.4574	0.8177	0.6376	0.5658	0.7437	0.2918
CV-366	0.7091	0.4787	0.8287	0.6537	0.5921	0.7538	0.3261
CV-367	0.7018	0.4787	0.8177	0.6482	0.5769	0.7513	0.3119
CV-368	0.7236	0.4681	0.8564	0.6622	0.6286	0.7561	0.3533
CV-369	0.7018	0.5000	0.8066	0.6533	0.5732	0.7565	0.3179
CV-370	0.6982	0.4787	0.8122	0.6454	0.5696	0.7500	0.3049
CV-371	0.7200	0.4681	0.8508	0.6595	0.6197	0.7549	0.3456
CV-372	0.7236	0.5000	0.8398	0.6699	0.6184	0.7638	0.3604
CV-373	0.7018	0.4255	0.8453	0.6354	0.5882	0.7391	0.2978
CV-374	0.7091	0.5000	0.8177	0.6588	0.5875	0.7590	0.3318
CV-375	0.6909	0.4468	0.8177	0.6322	0.5600	0.7400	0.2817
CV-376	0.7055	0.4681	0.8287	0.6484	0.5867	0.7500	0.3161
CV-377	0.6909	0.4255	0.8287	0.6271	0.5634	0.7353	0.2756
CV-378	0.7164	0.4681	0.8453	0.6567	0.6111	0.7537	0.3381
CV-379	0.7091	0.4681	0.8343	0.6512	0.5946	0.7512	0.3234
CV-380	0.6982	0.4894	0.8066	0.6480	0.5679	0.7526	0.3080
CV-381	0.7018	0.4894	0.8122	0.6508	0.5750	0.7538	0.3149
CV-382	0.7345	0.5106	0.8508	0.6807	0.6400	0.7700	0.3850
CV-383	0.7164	0.4787	0.8398	0.6593	0.6081	0.7562	0.3406
CV-384	0.7018	0.4574	0.8287	0.6431	0.5811	0.7463	0.3061
CV-385	0.7055	0.4574	0.8343	0.6459	0.5890	0.7475	0.3133
CV-386	0.7273	0.5426	0.8232	0.6829	0.6145	0.7760	0.3779
CV-387	0.6473	0.4681	0.7403	0.6042	0.4835	0.7283	0.2101
CV-388	0.6982	0.4255	0.8398	0.6327	0.5797	0.7379	0.2903
CV-389	0.7055	0.5000	0.8122	0.6561	0.5802	0.7577	0.3248
CV-390	0.7091	0.4894	0.8232	0.6563	0.5897	0.7563	0.3289
CV-391	0.6945	0.4681	0.8122	0.6401	0.5641	0.7462	0.2949
CV-392	0.7091	0.4574	0.8398	0.6486	0.5972	0.7488	0.3207

CV-393	0.7018	0.4681	0.8232	0.6456	0.5789	0.7487	0.3090
CV-394	0.6873	0.4362	0.8177	0.6269	0.5541	0.7363	0.2715
CV-395	0.7018	0.5106	0.8011	0.6559	0.5714	0.7592	0.3210
CV-396	0.6873	0.4787	0.7956	0.6372	0.5488	0.7461	0.2844
CV-397	0.7055	0.4468	0.8398	0.6433	0.5915	0.7451	0.3106
CV-398	0.7091	0.5213	0.8066	0.6640	0.5833	0.7644	0.3377
CV-399	0.7236	0.4574	0.8619	0.6597	0.6324	0.7536	0.3511
CV-400	0.7055	0.4787	0.8232	0.6510	0.5844	0.7525	0.3190
CV-401	0.7127	0.5000	0.8232	0.6616	0.5949	0.7602	0.3388
CV-402	0.6800	0.4894	0.7790	0.6342	0.5349	0.7460	0.2746
CV-403	0.6945	0.4255	0.8343	0.6299	0.5714	0.7366	0.2829
CV-404	0.7273	0.5319	0.8287	0.6803	0.6173	0.7732	0.3753
CV-405	0.6873	0.4681	0.8011	0.6346	0.5500	0.7436	0.2811
CV-406	0.7018	0.5000	0.8066	0.6533	0.5732	0.7565	0.3179
CV-407	0.6655	0.4362	0.7845	0.6104	0.5125	0.7282	0.2305
CV-408	0.6945	0.4894	0.8011	0.6452	0.5610	0.7513	0.3012
CV-409	0.7018	0.4894	0.8122	0.6508	0.5750	0.7538	0.3149
CV-410	0.7055	0.4681	0.8287	0.6484	0.5867	0.7500	0.3161
CV-411	0.7018	0.5000	0.8066	0.6533	0.5732	0.7565	0.3179
CV-412	0.7091	0.5106	0.8122	0.6614	0.5854	0.7617	0.3347
CV-413	0.6873	0.5000	0.7845	0.6423	0.5465	0.7513	0.2911
CV-414	0.7055	0.4574	0.8343	0.6459	0.5890	0.7475	0.3133
CV-415	0.6836	0.5000	0.7790	0.6395	0.5402	0.7500	0.2846
CV-416	0.7164	0.5000	0.8287	0.6644	0.6026	0.7614	0.3459
CV-417	0.6873	0.4468	0.8122	0.6295	0.5526	0.7387	0.2747
CV-418	0.7091	0.4787	0.8287	0.6537	0.5921	0.7538	0.3261
CV-419	0.6909	0.4681	0.8066	0.6374	0.5570	0.7449	0.2880
CV-420	0.7164	0.4787	0.8398	0.6593	0.6081	0.7562	0.3406
CV-421	0.6909	0.4574	0.8122	0.6348	0.5584	0.7424	0.2848
CV-422	0.6800	0.4894	0.7790	0.6342	0.5349	0.7460	0.2746
CV-423	0.7018	0.4894	0.8122	0.6508	0.5750	0.7538	0.3149
CV-424	0.7164	0.4894	0.8343	0.6618	0.6053	0.7588	0.3432
CV-425	0.7164	0.4787	0.8398	0.6593	0.6081	0.7562	0.3406
CV-426	0.7018	0.4468	0.8343	0.6405	0.5833	0.7438	0.3032
CV-427	0.7091	0.5106	0.8122	0.6614	0.5854	0.7617	0.3347
CV-428	0.6982	0.5319	0.7845	0.6582	0.5618	0.7634	0.3208
CV-429	0.7127	0.4787	0.8343	0.6565	0.6000	0.7550	0.3333
CV-430	0.7200	0.4787	0.8453	0.6620	0.6164	0.7574	0.3481
CV-431	0.7127	0.4894	0.8287	0.6590	0.5974	0.7576	0.3360
CV-432	0.7018	0.4894	0.8122	0.6508	0.5750	0.7538	0.3149
CV-433	0.7055	0.4681	0.8287	0.6484	0.5867	0.7500	0.3161
CV-434	0.6836	0.4894	0.7845	0.6369	0.5412	0.7474	0.2811
CV-435	0.7055	0.4894	0.8177	0.6535	0.5823	0.7551	0.3219
CV-436	0.7055	0.4681	0.8287	0.6484	0.5867	0.7500	0.3161
CV-437	0.6909	0.5000	0.7901	0.6450	0.5529	0.7526	0.2977
CV-438	0.6945	0.5106	0.7901	0.6503	0.5581	0.7566	0.3076
CV-439	0.7164	0.5000	0.8287	0.6644	0.6026	0.7614	0.3459
CV-440	0.7273	0.5426	0.8232	0.6829	0.6145	0.7760	0.3779
CV-441	0.7127	0.5106	0.8177	0.6642	0.5926	0.7629	0.3416
CV-442	0.7055	0.5000	0.8122	0.6561	0.5802	0.7577	0.3248
CV-443	0.6982	0.5106	0.7956	0.6531	0.5647	0.7579	0.3143
CV-444	0.7164	0.5213	0.8177	0.6695	0.5976	0.7668	0.3514
CV-445	0.6945	0.4894	0.8011	0.6452	0.5610	0.7513	0.3012
CV-446	0.7091	0.4894	0.8232	0.6563	0.5897	0.7563	0.3289
CV-447	0.7273	0.5532	0.8177	0.6854	0.6118	0.7789	0.3807
CV-448	0.7164	0.4894	0.8343	0.6618	0.6053	0.7588	0.3432
CV-449	0.7164	0.5000	0.8287	0.6644	0.6026	0.7614	0.3459

CV-450	0.7055	0.4787	0.8232	0.6510	0.5844	0.7525	0.3190
CV-451	0.7127	0.4681	0.8398	0.6539	0.6027	0.7525	0.3307
CV-452	0.6982	0.5106	0.7956	0.6531	0.5647	0.7579	0.3143
CV-453	0.6982	0.4362	0.8343	0.6352	0.5775	0.7402	0.2931
CV-454	0.6800	0.4574	0.7956	0.6265	0.5375	0.7385	0.2642
CV-455	0.6909	0.4468	0.8177	0.6322	0.5600	0.7400	0.2817
CV-456	0.6982	0.4894	0.8066	0.6480	0.5679	0.7526	0.3080
CV-457	0.7200	0.4574	0.8564	0.6569	0.6232	0.7524	0.3433
CV-458	0.6945	0.4468	0.8232	0.6350	0.5676	0.7413	0.2888
CV-459	0.6909	0.4574	0.8122	0.6348	0.5584	0.7424	0.2848
CV-460	0.6764	0.4468	0.7956	0.6212	0.5316	0.7347	0.2541
CV-461	0.7382	0.5319	0.8453	0.6886	0.6410	0.7766	0.3969
CV-462	0.6691	0.4255	0.7956	0.6106	0.5195	0.7273	0.2336
CV-463	0.6945	0.5106	0.7901	0.6503	0.5581	0.7566	0.3076
CV-464	0.7200	0.5000	0.8343	0.6671	0.6104	0.7626	0.3531
CV-465	0.7200	0.5000	0.8343	0.6671	0.6104	0.7626	0.3531
CV-466	0.6909	0.4574	0.8122	0.6348	0.5584	0.7424	0.2848
CV-467	0.6764	0.4255	0.8066	0.6161	0.5333	0.7300	0.2473
CV-468	0.7055	0.5213	0.8011	0.6612	0.5765	0.7632	0.3309
CV-469	0.6945	0.4894	0.8011	0.6452	0.5610	0.7513	0.3012
CV-470	0.6945	0.4681	0.8122	0.6401	0.5641	0.7462	0.2949
CV-471	0.6982	0.5106	0.7956	0.6531	0.5647	0.7579	0.3143
CV-472	0.7309	0.5106	0.8453	0.6780	0.6316	0.7688	0.3775
CV-473	0.6982	0.4894	0.8066	0.6480	0.5679	0.7526	0.3080
CV-474	0.6691	0.4681	0.7735	0.6208	0.5176	0.7368	0.2479
CV-475	0.7164	0.5426	0.8066	0.6746	0.5930	0.7725	0.3573
CV-476	0.7200	0.5000	0.8343	0.6671	0.6104	0.7626	0.3531
CV-477	0.6909	0.4894	0.7956	0.6425	0.5542	0.7500	0.2944
CV-478	0.7091	0.4681	0.8343	0.6512	0.5946	0.7512	0.3234
CV-479	0.7164	0.4894	0.8343	0.6618	0.6053	0.7588	0.3432
CV-480	0.7200	0.4894	0.8398	0.6646	0.6133	0.7600	0.3505
CV-481	0.7273	0.5319	0.8287	0.6803	0.6173	0.7732	0.3753
CV-482	0.6800	0.4574	0.7956	0.6265	0.5375	0.7385	0.2642
CV-483	0.6727	0.4574	0.7845	0.6210	0.5244	0.7358	0.2509
CV-484	0.6945	0.4574	0.8177	0.6376	0.5658	0.7437	0.2918
CV-485	0.6909	0.4468	0.8177	0.6322	0.5600	0.7400	0.2817
CV-486	0.6982	0.4468	0.8287	0.6378	0.5753	0.7426	0.2960
CV-487	0.7055	0.5000	0.8122	0.6561	0.5802	0.7577	0.3248
CV-488	0.6836	0.4787	0.7901	0.6344	0.5422	0.7448	0.2777
CV-489	0.7164	0.5213	0.8177	0.6695	0.5976	0.7668	0.3514
CV-490	0.7055	0.4574	0.8343	0.6459	0.5890	0.7475	0.3133
CV-491	0.7309	0.5000	0.8508	0.6754	0.6351	0.7662	0.3752
CV-492	0.7309	0.5106	0.8453	0.6780	0.6316	0.7688	0.3775
CV-493	0.6945	0.5000	0.7956	0.6478	0.5595	0.7539	0.3044
CV-494	0.6945	0.4894	0.8011	0.6452	0.5610	0.7513	0.3012
CV-495	0.7164	0.5000	0.8287	0.6644	0.6026	0.7614	0.3459
CV-496	0.6909	0.4681	0.8066	0.6374	0.5570	0.7449	0.2880
CV-497	0.7091	0.5000	0.8177	0.6588	0.5875	0.7590	0.3318
CV-498	0.7055	0.4574	0.8343	0.6459	0.5890	0.7475	0.3133
CV-499	0.7055	0.4894	0.8177	0.6535	0.5823	0.7551	0.3219
CV-500	0.7127	0.4787	0.8343	0.6565	0.6000	0.7550	0.3333

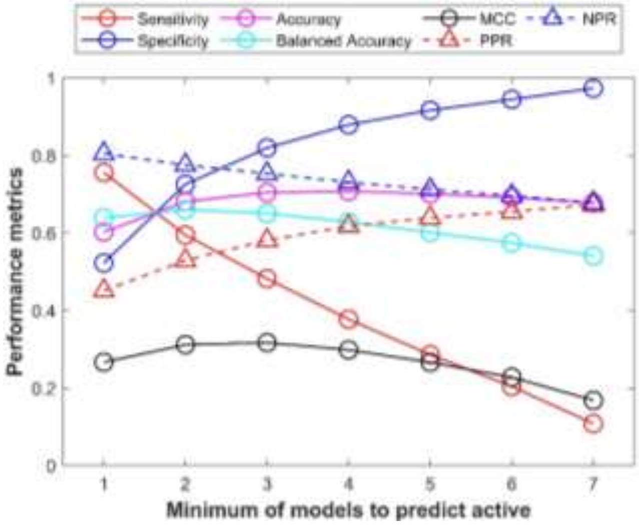

Figure S1. Performance of consensus models with different model voting. The x-axis represents the minimum of models to predict positive among the seven individual models. The y-axis indicated the prediction performance metrics. Each line represented one of the seven prediction performance metrics. MCC: Matthews correlation coefficient; PPR: positive prediction rate; NPR: negative prediction rate. The accuracy was plotted in pink line with circle, sensitivity in red line with circle, specificity in blue line with circle, balanced accuracy in cyan with circle, MCC in black line with circle, PPR in red dash line with triangle, and NPR in blue dash line with triangle.

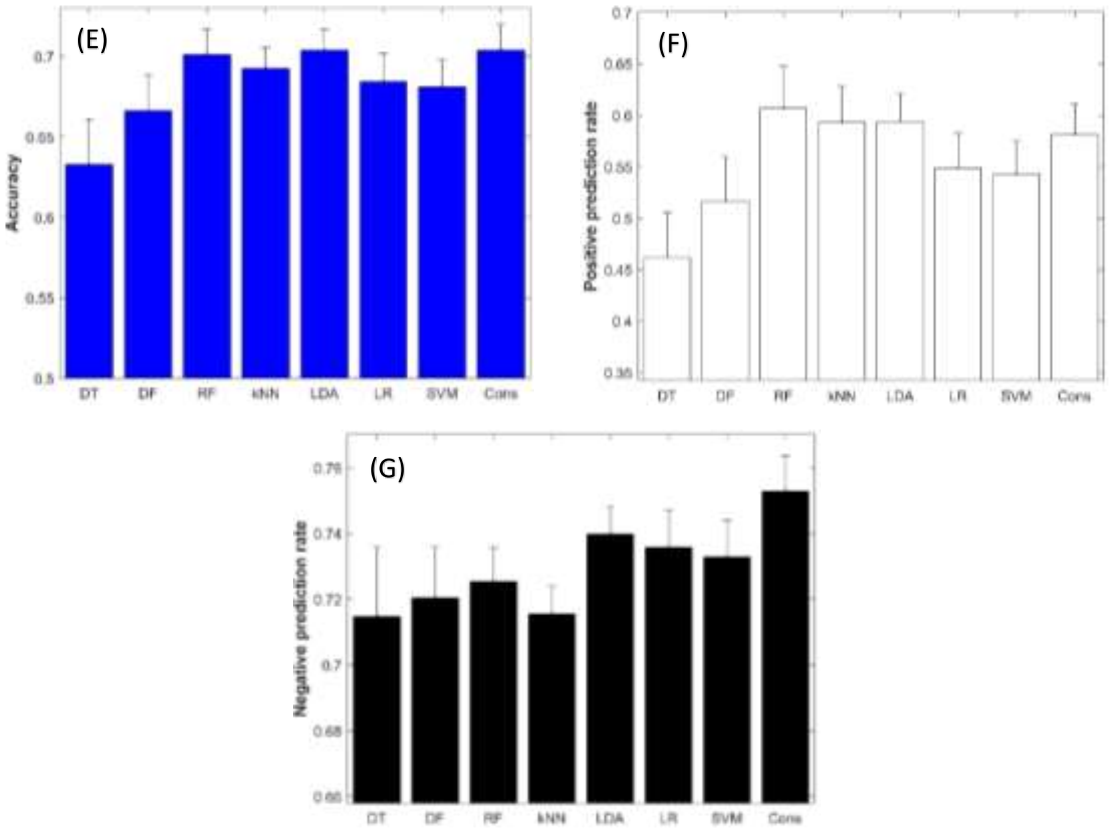

Figure S2. Average performance of 5-fold cross-validation (E) Accuracy, (F) Positive prediction rate, and (G) Negative prediction rate. X-axis indicated different models and the average performance metrics values were given as the y-axis. The standard deviation from 500 values of performance metrics was also plotted. DT: decision tree; DF: decision forest; RF: random forest; kNN: k-nearest neighbors; LDA: linear discriminant analysis; LR: logistic regression; SVM: support vector machine; Cons: consensus model.

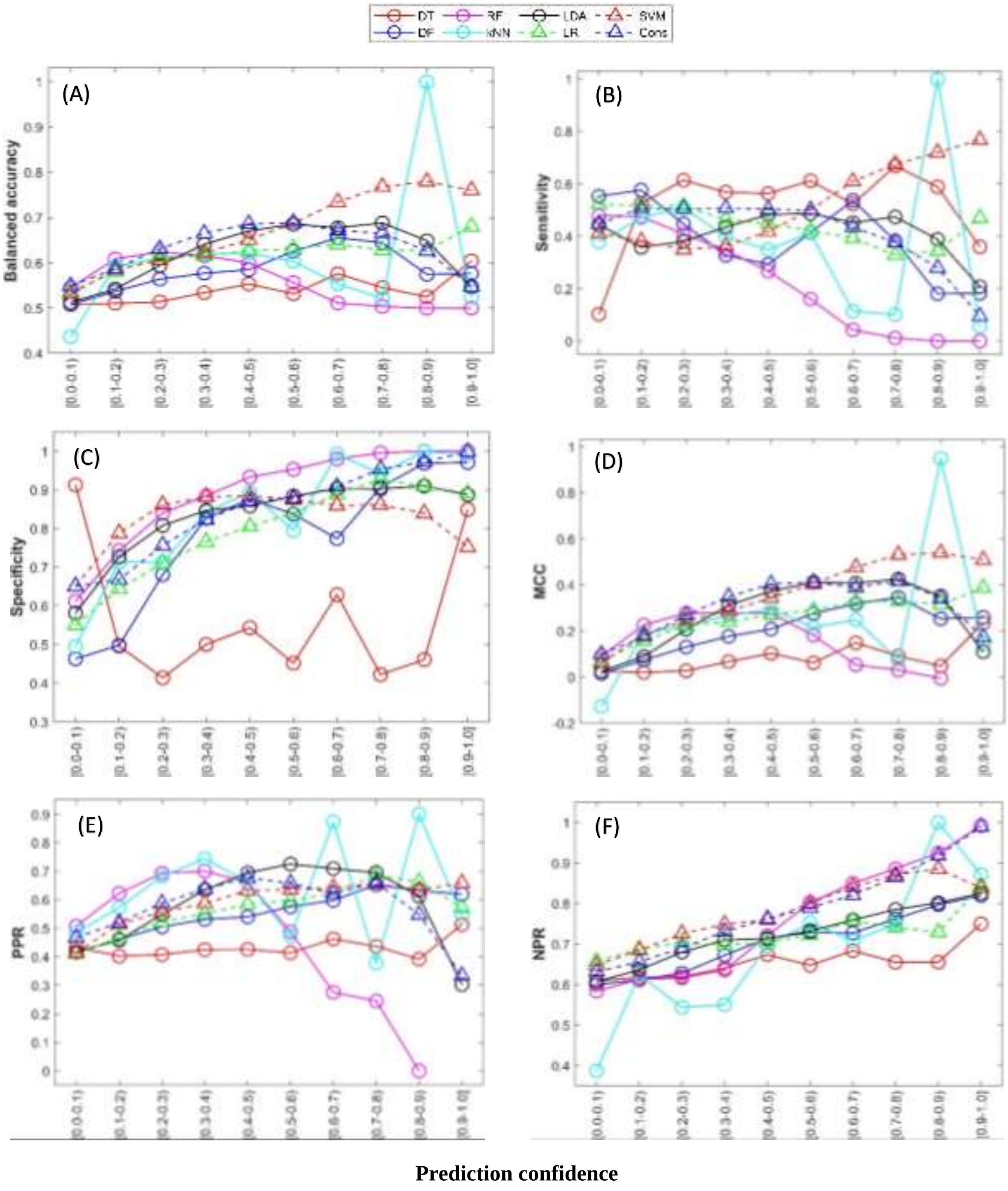

Figure S3. Prediction confidence analysis. (A) Balanced accuracy (B) Sensitivity (C) Specificity (D) MCC (Matthews correlation coefficient) (E) PPR (positive prediction rate) (F) NPR (negative prediction rate). Prediction confidence values were grouped into 10 bins with even interval of 0.1 and given at the x-axis. The prediction performance metrics were represented by y-axis. Models from DT (decision tree) were plotted in red line with circle, DF (decision forest) in blue line with circle, RF (random forest) in pink line with circle, kNN (k-nearest neighbors) in cyan line with circle, LDA (linear discriminant analysis) in black line with circle, LR (logistic regression) in green dash line with triangle, SVM (support vector machine) in red dash line with triangle, and Cons (consensus model) in blue dash line with triangle.

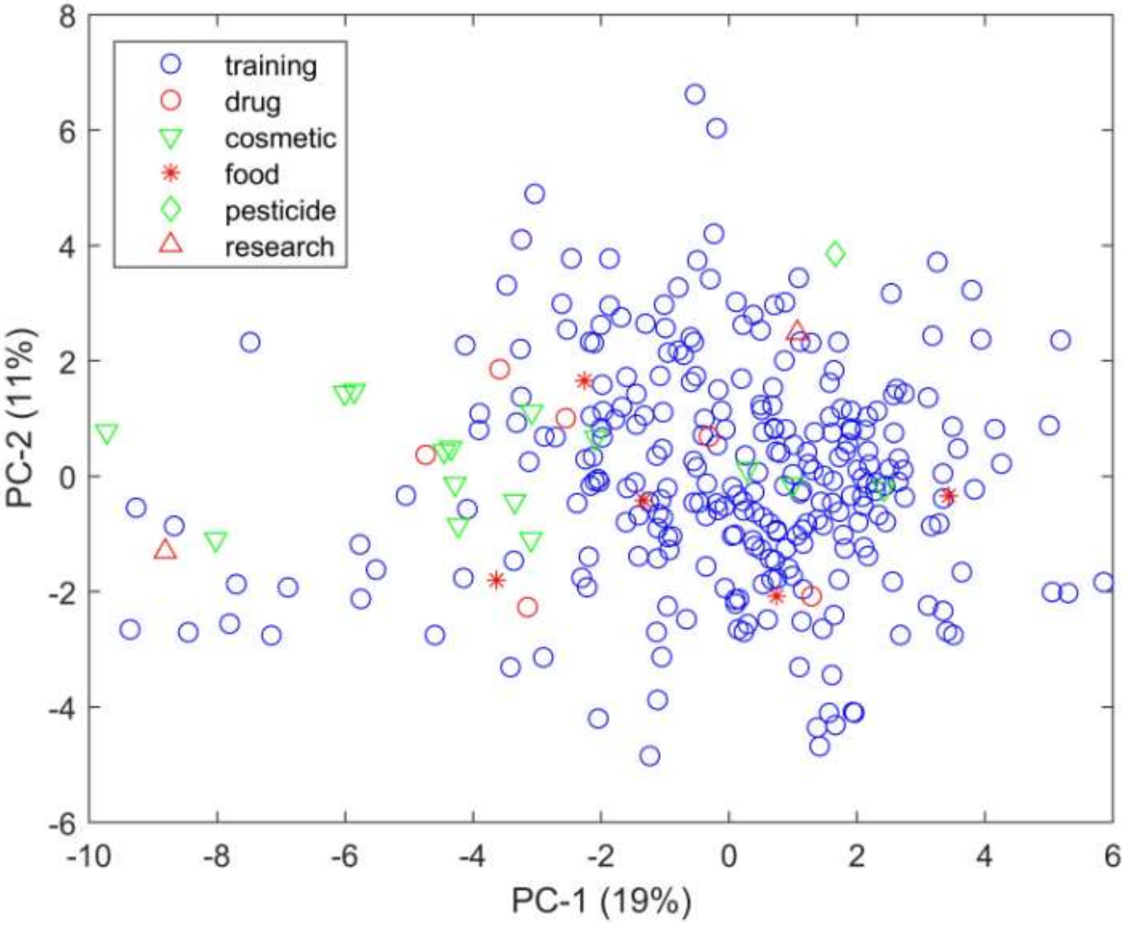

Figure S4. Scatter plot of chemicals in principal component analysis.

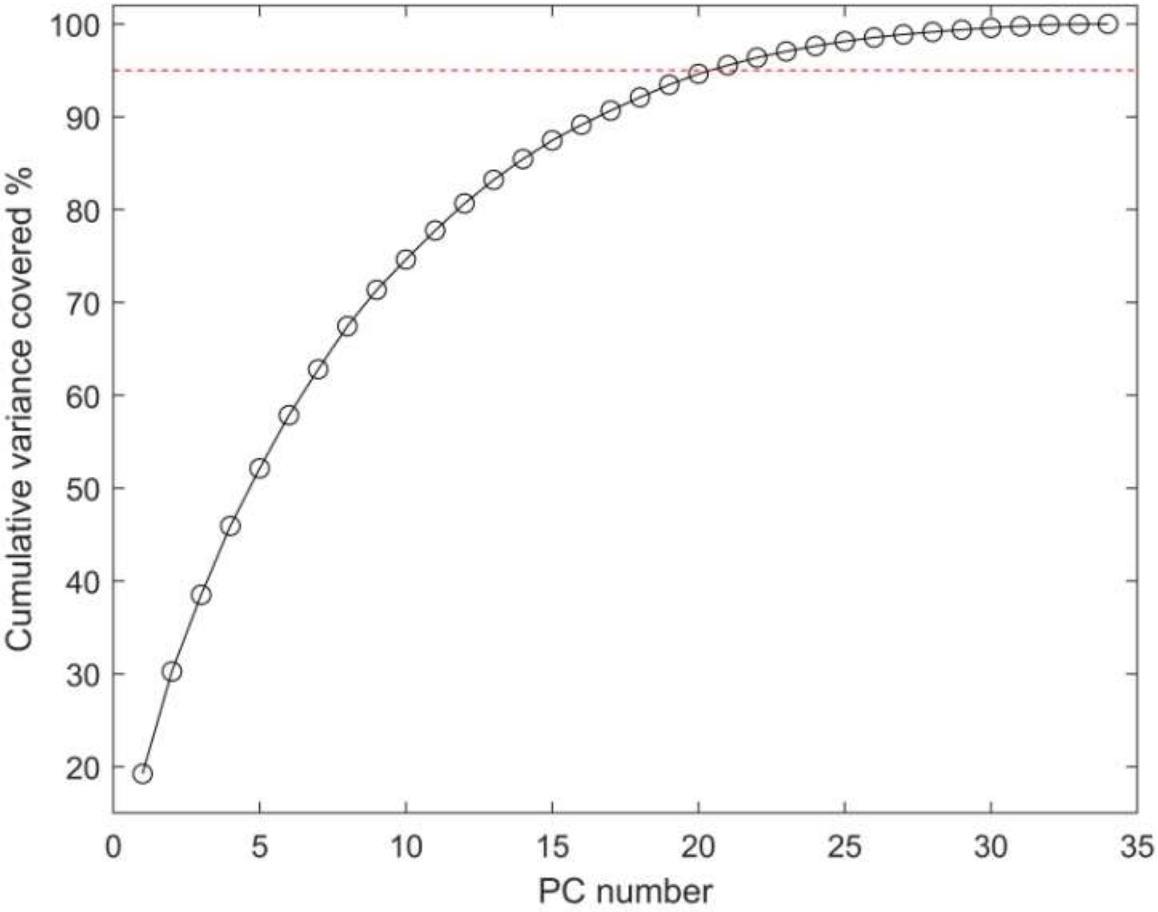

Figure S5. Distribution of variance coverage of principal components.

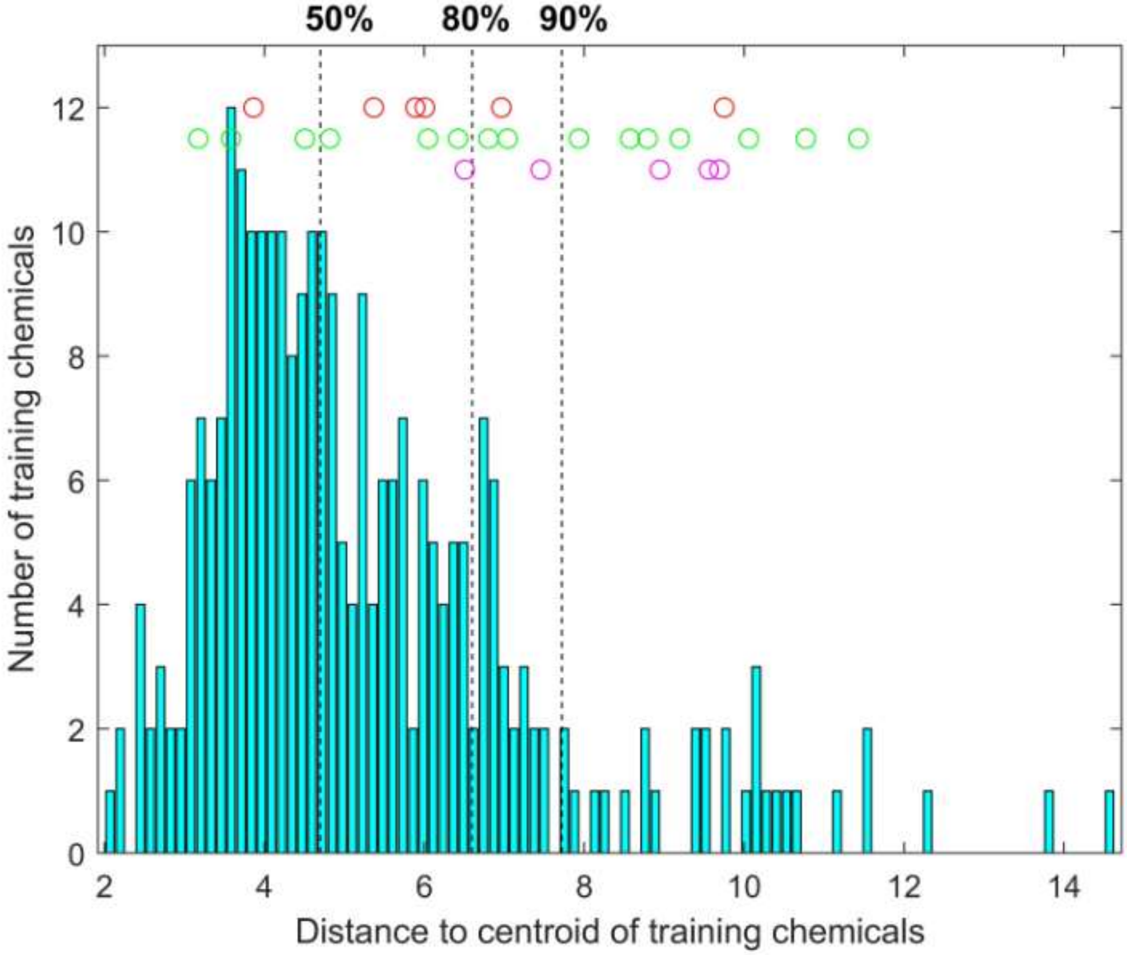

Figure S6. Applicability domain analysis. The x-axis indicates the distance to centroid of the training chemicals and the y-axis gives number of training chemicals represented by the cyan bars. The black dashed lines depict the distances of 50%, 80%, and 90% chemicals with shorter distances that the values. The drugs in the external validation set were plotted as red empty circles, the cosmetics as green empty circles, and food additives as magenta empty circles.

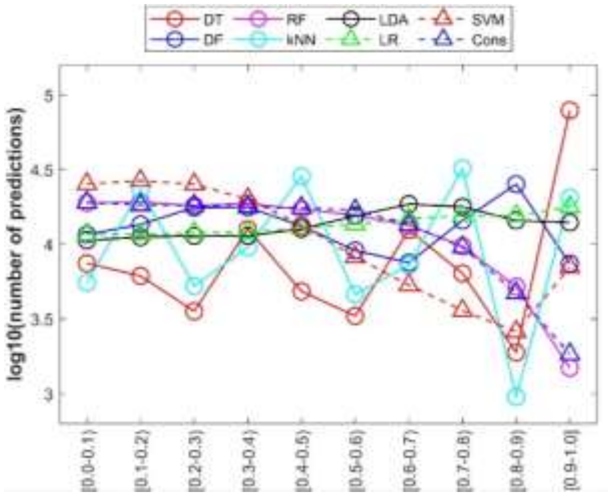

Prediction confidence

Figure S7. Distribution of predictions in prediction confidence analysis. The x-axis gave the prediction confidence values which were grouped into 10 bins with even interval of 0.1. Number of predictions in each bin of prediction confidence were counted and its log10 value were given as y-axis. Models from DT (decision tree) were plotted in red line with circle, DF (decision forest) in blue line with circle, RF (random forest) in pink line with circle, kNN (k-nearest neighbors) in cyan with circle, LDA (linear discriminant analysis) in black line with circle, LR (logistic regression) in green dash line with triangle, SVM (support vector machine) in red dash line with triangle, and Cons (consensus model) in blue dash line with triangle.

Python codes for Decision Tree (DT)

```
#!/usr/bin/env python
# coding: utf-8

from sklearn import model_selection
from sklearn.model_selection import cross_validate, validation_curve, check_cv, ParameterGrid
from sklearn.model_selection import cross_val_score, cross_val_predict
from sklearn.model_selection import StratifiedKFold, RepeatedStratifiedKFold , train_test_split
from sklearn.preprocessing import MinMaxScaler
from sklearn.ensemble import RandomForestClassifier
from sklearn import neighbors, datasets, metrics
from sklearn.datasets import make_classification
from sklearn.metrics import precision_score, recall_score, accuracy_score, classification_report, confusion_matrix
from sklearn.tree import DecisionTreeClassifier
from sklearn import tree
from sklearn.model_selection import RandomizedSearchCV
from scipy.stats import randint
from sklearn import svm
from sklearn.neighbors import KNeighborsClassifier
from sklearn.discriminant_analysis import LinearDiscriminantAnalysis
from sklearn.naive_bayes import GaussianNB
from sklearn.model_selection import GridSearchCV
from sklearn.metrics import balanced_accuracy_score, auc, roc_curve, matthews_corrcoef, make_scorer
from sklearn.neural_network import MLPClassifier
from statistics import mean, stdev
import statistics
import pandas as pd
import numpy as np
import pylab as pl
import xlrd
import os
import math
import re
import time
import random
from time import time
import matplotlib.pyplot as plt
import matplotlib.pylab as pylab

# Change these paths to a location under the home directory
HOME = 'C:/Users/Jie.Liu1/OneDrive - FDA/Desktop/toxrefdb-2021/model_development_052021/data_input/'

input1 = os.path.join(HOME, 'variable_selection/275chem_var_selection_34des.xlsx')
input2 = os.path.join(HOME, 'data input 05172021/Stratified-splitting-5fd.xlsx')

# data input1: toxrefdb data with 275 chemicals, 34 descriptors, and 1 toxicity label
DF1 = pd.read_excel(input1)
DF1.shape

# data input2: chemical indexes for 5-fold cross-validation, 500 times
DF2 = pd.read_excel(input2, header = None)
DF2.shape
```

```
##########################################################################
# cross-validation
##########################################################################
import time
start_time = time.time()
predicted = []
probability = []
important_features = []
chem_index = []

for i in range(0,500,1):
   for j in range(1,6,1):
       # select one fold of chemical index as the test data set
       test_index_0 = DF2.iloc[((j-1)*55+0):((j-1)*55+55), i]
       test_index = sorted(test_index_0)

       # subset test datasets
       test = DF1[DF1['chemical_id'].isin(test_index)]
       X_test = test.iloc[:, 4:38]
       Y_test = test.iloc[:, 3]

       #actual label
       index = test.iloc[:,0]
       chem_index.extend(index)

       # subset train datasets
       train = DF1[DF1['chemical_id'].isin(test_index) == False]
       X_train = train.iloc[:, 4:38]
       Y_train = train.iloc[:, 3]

       # classification
       clf =  DecisionTreeClassifier(min_samples_split=12, min_samples_leaf=6, random_state = 1)
       clf.fit(X_train, Y_train)

       # predicted label
       pred = clf.predict(X_test)
       predicted.extend(pred)

       # probability
       prob = clf.predict_proba(X_test)
       probability.extend(prob)

       # feature_importances
       feature_important = clf.feature_importances_
       important_features.append(feature_important)

print("The whole time needed is %.2f seconds" %(time.time()-start_time))


# prediction label
l1 = np.array(chem_index).reshape(500,275)
l2 = pd.DataFrame(l1).T
m1 = np.array(predicted).reshape(500,275)
m2 = pd.DataFrame(m1).T
```

```
combine = []
for t in range(0,500,1):
    m = pd.merge(l2.iloc[:,t], m2.iloc[:,t], how="outer", left_index = True, right_index = True)
    m.columns = ["chem", "predict_label_"]
    m3 = m.sort_values(by=["chem"])
    m4 = pd.DataFrame(m3).iloc[:,1]
    combine.append(m4)
m5 = np.array(combine).reshape(500,275)
m6 = pd.DataFrame(m5).T
# add original chemical id and toxicity label
chem = DF1.iloc[:, 0:4]
predict_label = pd.concat([chem, m6], axis = 1)
predict_label.to_excel('C:/Users/Jie.Liu1/OneDrive - FDA/Desktop/toxrefdb-2021/model_development_052021/model_output/decision tree/predict_label_dt.xlsx', index=False)

# performance
sensitivity = []
accuracy = []
specificity = []
balanced_ac = []
tppr = []
tnpr = []
mcc = []

for p in range(4,504,1):
    y_true = np.array(predict_label.iloc[:,3])
    y_pred = np.array(predict_label.iloc[:,p])
    TP = np.sum(predict_label[predict_label.iloc[:,3]==predict_label.iloc[:,p]].iloc[:,3]==1)
    TN = np.sum(predict_label[predict_label.iloc[:,3]==predict_label.iloc[:,p]].iloc[:,3]==0)
    FP = np.sum(predict_label[predict_label.iloc[:,3]!=predict_label.iloc[:,p]].iloc[:,3]==0)
    FN = np.sum(predict_label[predict_label.iloc[:,3]!=predict_label.iloc[:,p]].iloc[:,3]==1)

    recall = recall_score(y_true, y_pred)
    sensitivity.append(recall)
    acc = accuracy_score(y_true, y_pred)
    accuracy.append(acc)
    spec = TN/(TN+FP)
    specificity.append(spec)
    ba = balanced_accuracy_score(y_true, y_pred)
    balanced_ac.append(ba)
    precision = precision_score(y_true, y_pred)
    tppr.append(precision)
    tnprs = TN/(TN+FN)
    tnpr.append(tnprs)
    mccs = matthews_corrcoef(y_true, y_pred)
    mcc.append(mccs)

ac = pd.DataFrame(accuracy)
sen = pd.DataFrame(sensitivity)
sp = pd.DataFrame(specificity)
ba = pd.DataFrame(balanced_ac)
tppr_all = pd.DataFrame(tppr)
tnpr_all = pd.DataFrame(tnpr)
mcc_all = pd.DataFrame(mcc)
```

```
perf = pd.concat([ac, sen, sp, ba, tppr_all, tnpr_all, mcc_all], axis =1)
perf.columns = ["Accuracy","Sensitivity","Specificity", "Balanced accuracy", "PPR", "NPR", "MCC"]
perf.to_excel('C:/Users/Jie.Liu1/OneDrive - FDA/Desktop/toxrefdb-
2021/model_development_052021/model_output/decision tree/perf_dt.xlsx', index=False)

## prediction probability
## save predicted probability to active toxicity ("1") to a spreadsheet
prob1 = np.array(probability)[:,1].reshape(500,275)
prob2 = pd.DataFrame(prob1).T
combine_prob = []
for tt in range(0,500,1):
    prob = pd.merge(l2.iloc[:,tt], prob2.iloc[:,tt], how="outer", left_index = True, right_index = True)
    prob.columns = ["chem", "probability"]
    prob3 = prob.sort_values(by=["chem"])
    prob4 = pd.DataFrame(prob3).iloc[:,1]
    combine_prob.append(prob4)
prob5 = np.array(combine_prob).reshape(500,275)
prob6 = pd.DataFrame(prob5).T
# add original chemical id and toxicity label
chem = DF1.iloc[:, 0:4]
prob7 = pd.concat([chem, prob6], axis = 1)
prob7.to_excel('C:/Users/Jie.Liu1/OneDrive - FDA/Desktop/toxrefdb-
2021/model_development_052021/model_output/decision tree/probability_to_active_dt.xlsx', index=False)

# feature importances
feature = pd.DataFrame(important_features)
feature.columns = X_train.columns
feature.T.to_excel('C:/Users/Jie.Liu1/OneDrive - FDA/Desktop/toxrefdb-
2021/model_development_052021/model_output/decision tree/feature_im_values_2500_dt.xlsx')

##############################################################################
# external validation
##############################################################################
# import external data
input3 = os.path.join(HOME, 'external_data/external_data_34des.xlsx')
DF3 = pd.read_excel(input3)
DF3.shape            # 29  38
ex_test = DF3.iloc[:, 4:38]
X_train = DF1.iloc[:, 4:38]
Y_train = DF1.iloc[:, 3]
clf =  DecisionTreeClassifier(min_samples_split=12, min_samples_leaf=6, random_state = 1)
clf.fit(X_train, Y_train)

# predicted label
pred = clf.predict(ex_test)
# prediction probability
prob = clf.predict_proba(ex_test)
# save predicted labels to a spreadsheet
m1 = pd.DataFrame(pred)
chem = DF3.iloc[:, 0:4]
predict_label = pd.concat([chem, m1], axis = 1)
predict_label.to_excel('C:/Users/Jie.Liu1/OneDrive - FDA/Desktop/toxrefdb-
2021/model_development_052021/model_output/decision tree/predict_label_external_dt.xlsx', index=False)
```

```
# performance
y_true = np.array(predict_label.iloc[:,3])
y_pred = np.array(predict_label.iloc[:,4])
TP = np.sum(predict_label[predict_label.iloc[:,3]==predict_label.iloc[:,4]].iloc[:,3]==1)
TN = np.sum(predict_label[predict_label.iloc[:,3]==predict_label.iloc[:,4]].iloc[:,3]==0)
FP = np.sum(predict_label[predict_label.iloc[:,3]!=predict_label.iloc[:,4]].iloc[:,3]==0)
FN = np.sum(predict_label[predict_label.iloc[:,3]!=predict_label.iloc[:,4]].iloc[:,3]==1)
accuracy = accuracy_score(y_true, y_pred)
sensitivity = recall_score(y_true, y_pred)
specificity = TN/(TN+FP)
balanced_ac = balanced_accuracy_score(y_true, y_pred)
tppr = precision_score(y_true, y_pred)
tnpr = TN/(TN+FN)
mcc = matthews_corrcoef(y_true, y_pred)
perf_1 = [accuracy, sensitivity, specificity, balanced_ac, tppr, tnpr, mcc]
perf_dt = pd.DataFrame(perf_1).T
perf_dt.insert(0, "Algorithm", ["DT"], True)
perf_dt.columns = ["Algorithm", "Accuracy","Sensitivity","Specificity", "Balanced accuracy", "PPR", "NPR", "MCC"]
perf_dt.to_excel('C:/Users/Jie.Liu1/OneDrive - FDA/Desktop/toxrefdb-
2021/model_development_052021/model_output/decision tree/perf_external_dt.xlsx', index=False)

# prediction probability
prob1 = np.array(prob)[:,1].reshape(1,29)
prob2 = pd.DataFrame(prob1).T
# add original chemical id and toxicity label
chem = DF3.iloc[:, 0:4]
prob3 = pd.concat([chem, prob2], axis = 1)
prob3.to_excel('C:/Users/Jie.Liu1/OneDrive - FDA/Desktop/toxrefdb-
2021/model_development_052021/model_output/decision tree/probability_to_active_external_dt.xlsx', index=False)
```

Python codes for Random Forest (RF)

```
#!/usr/bin/env python
# coding: utf-8

# Change these paths to a location under the home directory
HOME = 'C:/Users/Jie.Liu1/OneDrive - FDA/Desktop/toxrefdb-2021/model_development_052021/data_input/'
input1 = os.path.join(HOME, 'variable_selection/275chem_var_selection_34des.xlsx')
input2 = os.path.join(HOME, 'data input 05172021/Stratified-splitting-5fd.xlsx')

# data input1: toxrefdb data with 275 chemicals, 34 descriptors, and 1 toxicity label
DF1 = pd.read_excel(input1)
DF1.shape

# data input2: chemical indexes for 5-fold cross-validation, 500 times
DF2 = pd.read_excel(input2, header = None)
DF2.shape

################################################################################
# cross-validation
################################################################################
import time
start_time = time.time()
predicted = []
probability = []
important_features = []
chem_index = []

for i in range(0,500,1):
    for j in range(1,6,1):
        # select one fold of chemical index as the test data set
        test_index_0 = DF2.iloc[((j-1)*55+0):((j-1)*55+55), i]
        test_index = sorted(test_index_0)

        # subset test datasets
        test = DF1[DF1['chemical_id'].isin(test_index)]
        X_test = test.iloc[:, 4:38]
        Y_test = test.iloc[:, 3]

        #actual label
        index = test.iloc[:,0]
        chem_index.extend(index)

        # subset train datasets
        train = DF1[DF1['chemical_id'].isin(test_index) == False]
        X_train = train.iloc[:, 4:38]
        Y_train = train.iloc[:, 3]

        # classification
        clf =  RandomForestClassifier(n_estimators = 500, max_features = 7, max_samples = 220, random_state = 1)
        clf.fit(X_train, Y_train)

        # predicted label
        pred = clf.predict(X_test)
```

```
        predicted.extend(pred)

        # probability
        prob = clf.predict_proba(X_test)
        probability.extend(prob)

        # feature_importances
        feature_important = clf.feature_importances_
        important_features.append(feature_important)

print("The whole time needed is %.2f seconds" %(time.time()-start_time))


# prediction label
l1 = np.array(chem_index).reshape(500,275)
l2 = pd.DataFrame(l1).T
m1 = np.array(predicted).reshape(500,275)
m2 = pd.DataFrame(m1).T
combine = []
for t in range(0,500,1):
    m = pd.merge(l2.iloc[:,t], m2.iloc[:,t], how="outer", left_index = True, right_index = True)
    m.columns = ["chem", "predict_label_"]
    m3 = m.sort_values(by=["chem"])
    m4 = pd.DataFrame(m3).iloc[:,1]
    combine.append(m4)
m5 = np.array(combine).reshape(500,275)
m6 = pd.DataFrame(m5).T
# add original chemical id and toxicity label
chem = DF1.iloc[:, 0:4]
predict_label = pd.concat([chem, m6], axis = 1)
predict_label.to_excel('C:/Users/Jie.Liu1/OneDrive - FDA/Desktop/toxrefdb-
2021/model_development_052021/model_output/random forest/predict_label_rf.xlsx', index=False)

# performance
sensitivity = []
accuracy = []
specificity = []
balanced_ac = []
tppr = []
tnpr = []
mcc = []

for p in range(4,504,1):
    y_true = np.array(predict_label.iloc[:,3])
    y_pred = np.array(predict_label.iloc[:,p])
    TP = np.sum(predict_label[predict_label.iloc[:,3]==predict_label.iloc[:,p]].iloc[:,3]==1)
    TN = np.sum(predict_label[predict_label.iloc[:,3]==predict_label.iloc[:,p]].iloc[:,3]==0)
    FP = np.sum(predict_label[predict_label.iloc[:,3]!=predict_label.iloc[:,p]].iloc[:,3]==0)
    FN = np.sum(predict_label[predict_label.iloc[:,3]!=predict_label.iloc[:,p]].iloc[:,3]==1)

    recall = recall_score(y_true, y_pred)
    sensitivity.append(recall)
    acc = accuracy_score(y_true, y_pred)
    accuracy.append(acc)
```

```
    spec = TN/(TN+FP)
    specificity.append(spec)
    ba = balanced_accuracy_score(y_true, y_pred)
    balanced_ac.append(ba)
    precision = precision_score(y_true, y_pred)
    tppr.append(precision)
    tnprs = TN/(TN+FN)
    tnpr.append(tnprs)
    mccs = matthews_corrcoef(y_true, y_pred)
    mcc.append(mccs)

ac = pd.DataFrame(accuracy)
sen = pd.DataFrame(sensitivity)
sp = pd.DataFrame(specificity)
ba = pd.DataFrame(balanced_ac)
tppr_all = pd.DataFrame(tppr)
tnpr_all = pd.DataFrame(tnpr)
mcc_all = pd.DataFrame(mcc)
perf = pd.concat([ac, sen, sp, ba, tppr_all, tnpr_all, mcc_all], axis =1)
perf.columns = ["Accuracy","Sensitivity","Specificity", "Balanced accuracy", "PPR", "NPR", "MCC"]
perf.to_excel('C:/Users/Jie.Liu1/OneDrive - FDA/Desktop/toxrefdb-
2021/model_development_052021/model_output/random forest/perf_rf.xlsx', index=False)

## prediction probability
## save predicted probability to active toxicity ("1") to a spreadsheet
prob1 = np.array(probability)[:,1].reshape(500,275)
prob2 = pd.DataFrame(prob1).T
combine_prob = []
for tt in range(0,500,1):
    prob = pd.merge(l2.iloc[:,tt], prob2.iloc[:,tt], how="outer", left_index = True, right_index = True)
    prob.columns = ["chem", "probability"]
    prob3 = prob.sort_values(by=["chem"])
    prob4 = pd.DataFrame(prob3).iloc[:,1]
    combine_prob.append(prob4)
prob5 = np.array(combine_prob).reshape(500,275)
prob6 = pd.DataFrame(prob5).T
# add original chemical id and toxicity label
chem = DF1.iloc[:, 0:4]
prob7 = pd.concat([chem, prob6], axis = 1)
prob7.to_excel('C:/Users/Jie.Liu1/OneDrive - FDA/Desktop/toxrefdb-
2021/model_development_052021/model_output/random forest/probability_to_active_rf.xlsx', index=False)

# feature importances
feature = pd.DataFrame(important_features)
feature.columns = X_train.columns
feature.T.to_excel('C:/Users/Jie.Liu1/OneDrive - FDA/Desktop/toxrefdb-
2021/model_development_052021/model_output/random forest/feature_im_values_2500_rf.xlsx')

##########################################################################
# external validation
##########################################################################
# import external data
input3 = os.path.join(HOME, 'external_data/external_data_34des.xlsx')
DF3 = pd.read_excel(input3)
```

```
DF3.shape            # 29  38
ex_test = DF3.iloc[:, 4:38]
X_train = DF1.iloc[:, 4:38]
Y_train = DF1.iloc[:, 3]
clf =  RandomForestClassifier(n_estimators = 500, max_features = 7, max_samples = 220, random_state = 1)
clf.fit(X_train,  Y_train)

# predicted label
pred = clf.predict(ex_test)
# prediction probability
prob = clf.predict_proba(ex_test)
# save predicted labels to a spreadsheet
m1 = pd.DataFrame(pred)
chem = DF3.iloc[:, 0:4]
predict_label = pd.concat([chem, m1], axis = 1)
predict_label.to_excel('C:/Users/Jie.Liu1/OneDrive - FDA/Desktop/toxrefdb-
2021/model_development_052021/model_output/random forest/predict_label_external_rf.xlsx', index=False)

# performance
y_true = np.array(predict_label.iloc[:,3])
y_pred = np.array(predict_label.iloc[:,4])
TP = np.sum(predict_label[predict_label.iloc[:,3]==predict_label.iloc[:,4]].iloc[:,3]==1)
TN = np.sum(predict_label[predict_label.iloc[:,3]==predict_label.iloc[:,4]].iloc[:,3]==0)
FP = np.sum(predict_label[predict_label.iloc[:,3]!=predict_label.iloc[:,4]].iloc[:,3]==0)
FN = np.sum(predict_label[predict_label.iloc[:,3]!=predict_label.iloc[:,4]].iloc[:,3]==1)
accuracy = accuracy_score(y_true, y_pred)
sensitivity = recall_score(y_true, y_pred)
specificity = TN/(TN+FP)
balanced_ac = balanced_accuracy_score(y_true, y_pred)
tppr = precision_score(y_true, y_pred)
tnpr = TN/(TN+FN)
mcc = matthews_corrcoef(y_true, y_pred)
perf_1 = [accuracy, sensitivity, specificity, balanced_ac, tppr, tnpr, mcc]
perf_dt = pd.DataFrame(perf_1).T
perf_dt.insert(0, "Algorithm", ["DT"], True)
perf_dt.columns = ["Algorithm", "Accuracy","Sensitivity","Specificity", "Balanced accuracy", "PPR", "NPR", "MCC"]
perf_dt.to_excel('C:/Users/Jie.Liu1/OneDrive - FDA/Desktop/toxrefdb-
2021/model_development_052021/model_output/random forest/perf_external_rf.xlsx', index=False)

# prediction probability
prob1 = np.array(prob)[:,1].reshape(1,29)
prob2 = pd.DataFrame(prob1).T
# add original chemical id and toxicity label
chem = DF3.iloc[:, 0:4]
prob3 = pd.concat([chem, prob2], axis = 1)
prob3.to_excel('C:/Users/Jie.Liu1/OneDrive - FDA/Desktop/toxrefdb-
2021/model_development_052021/model_output/random forest/probability_to_active_external_rf.xlsx', index=False)
```

Python codes for k-Nearest Neighbors (kNN)

```
#!/usr/bin/env python
# coding: utf-8

###############################################################################
# auto scale training and external data
# (1) autoscale trainging data:  transform value(ij) = (value(ij) - mean(j)(train)) / std(j)(train)
# (2) autoscale external data:  transform value (kj) = (value(kj) - mean(j)(train))/ std(j)(train)
#      i, k – row number; j – column number.
###############################################################################

# Change these paths to a location under the home directory
HOME = 'C:/Users/Jie.Liu1/OneDrive - FDA/Desktop/toxrefdb-2021/model_development_052021/data_input/'
input1 = os.path.join(HOME, 'variable_selection/275chem_var_selection_34des_autoscale.xlsx')
input2 = os.path.join(HOME, 'data input 05172021/Stratified-splitting-5fd.xlsx')

# data input1: toxrefdb data with 275 chemicals, 34 descriptors, and 1 toxicity label
DF1 = pd.read_excel(input1)
DF1.shape
# data input2: chemical indexes for 5-fold cross-validation, 500 times
DF2 = pd.read_excel(input2, header = None)
DF2.shape

#########################################################################
# cross-validation
#########################################################################
import time
start_time = time.time()
predicted = []
probability = []
chem_index = []

for i in range(0,500,1):
   for j in range(1,6,1):
      # select one fold of chemical index as the test data set
      test_index_0 = DF2.iloc[((j-1)*55+0):((j-1)*55+55), i]
      test_index = sorted(test_index_0)

      # subset test datasets
      test = DF1[DF1['chemical_id'].isin(test_index)]
      X_test = test.iloc[:, 4:38]
      Y_test = test.iloc[:, 3]

      #actual label
      index = test.iloc[:,0]
      chem_index.extend(index)

      # subset train datasets
      train = DF1[DF1['chemical_id'].isin(test_index) == False]
      X_train = train.iloc[:, 4:38]
      Y_train = train.iloc[:, 3]
```

```
        # classification
        clf = KNeighborsClassifier(n_neighbors=7, p=1, weights='distance', n_jobs=-1, random_state = 1)
        clf.fit(X_train, Y_train)

        # predicted label
        pred = clf.predict(X_test)
        predicted.extend(pred)

        # probability
        prob = clf.predict_proba(X_test)
        probability.extend(prob)

print("The whole time needed is %.2f seconds" %(time.time()-start_time))


# prediction label
l1 = np.array(chem_index).reshape(500,275)
l2 = pd.DataFrame(l1).T
m1 = np.array(predicted).reshape(500,275)
m2 = pd.DataFrame(m1).T
combine = []
for t in range(0,500,1):
    m = pd.merge(l2.iloc[:,t], m2.iloc[:,t], how="outer", left_index = True, right_index = True)
    m.columns = ["chem", "predict_label_"]
    m3 = m.sort_values(by=["chem"])
    m4 = pd.DataFrame(m3).iloc[:,1]
    combine.append(m4)
m5 = np.array(combine).reshape(500,275)
m6 = pd.DataFrame(m5).T
# add original chemical id and toxicity label
chem = DF1.iloc[:, 0:4]
predict_label = pd.concat([chem, m6], axis = 1)
predict_label.to_excel('C:/Users/Jie.Liu1/OneDrive - FDA/Desktop/toxrefdb-
2021/model_development_052021/model_output/knn/predict_label_knn.xlsx', index=False)

# performance
sensitivity = []
accuracy = []
specificity = []
balanced_ac = []
tppr = []
tnpr = []
mcc = []

for p in range(4,504,1):
    y_true = np.array(predict_label.iloc[:,3])
    y_pred = np.array(predict_label.iloc[:,p])
    TP = np.sum(predict_label[predict_label.iloc[:,3]==predict_label.iloc[:,p]].iloc[:,3]==1)
    TN = np.sum(predict_label[predict_label.iloc[:,3]==predict_label.iloc[:,p]].iloc[:,3]==0)
    FP = np.sum(predict_label[predict_label.iloc[:,3]!=predict_label.iloc[:,p]].iloc[:,3]==0)
    FN = np.sum(predict_label[predict_label.iloc[:,3]!=predict_label.iloc[:,p]].iloc[:,3]==1)

    recall = recall_score(y_true, y_pred)
    sensitivity.append(recall)
```

```
    acc = accuracy_score(y_true, y_pred)
    accuracy.append(acc)
    spec = TN/(TN+FP)
    specificity.append(spec)
    ba = balanced_accuracy_score(y_true, y_pred)
    balanced_ac.append(ba)
    precision = precision_score(y_true, y_pred)
    tppr.append(precision)
    tnprs = TN/(TN+FN)
    tnpr.append(tnprs)
    mccs = matthews_corrcoef(y_true, y_pred)
    mcc.append(mccs)

ac = pd.DataFrame(accuracy)
sen = pd.DataFrame(sensitivity)
sp = pd.DataFrame(specificity)
ba = pd.DataFrame(balanced_ac)
tppr_all = pd.DataFrame(tppr)
tnpr_all = pd.DataFrame(tnpr)
mcc_all = pd.DataFrame(mcc)
perf = pd.concat([ac, sen, sp, ba, tppr_all, tnpr_all, mcc_all], axis =1)
perf.columns = ["Accuracy","Sensitivity","Specificity", "Balanced accuracy", "PPR", "NPR", "MCC"]
perf.to_excel('C:/Users/Jie.Liu1/OneDrive - FDA/Desktop/toxrefdb-
2021/model_development_052021/model_output/knn/perf_knn.xlsx', index=False)

## prediction probability
## save predicted probability to active toxicity ("1") to a spreadsheet
prob1 = np.array(probability)[:,1].reshape(500,275)
prob2 = pd.DataFrame(prob1).T
combine_prob = []
for tt in range(0,500,1):
    prob = pd.merge(l2.iloc[:,tt], prob2.iloc[:,tt], how="outer", left_index = True, right_index = True)
    prob.columns = ["chem", "probability"]
    prob3 = prob.sort_values(by=["chem"])
    prob4 = pd.DataFrame(prob3).iloc[:,1]
    combine_prob.append(prob4)
prob5 = np.array(combine_prob).reshape(500,275)
prob6 = pd.DataFrame(prob5).T
# add original chemical id and toxicity label
chem = DF1.iloc[:, 0:4]
prob7 = pd.concat([chem, prob6], axis = 1)
prob7.to_excel('C:/Users/Jie.Liu1/OneDrive - FDA/Desktop/toxrefdb-
2021/model_development_052021/model_output/knn/probability_to_active_knn.xlsx', index=False)

##########################################################################
# external validation
##########################################################################

# import external data
input3 = os.path.join(HOME, 'external_data/external_data_34des_autoscale_new.xlsx')
DF3 = pd.read_excel(input3)
DF3.shape          # 29  38
ex_test = DF3.iloc[:, 4:38]
X_train = DF1.iloc[:, 4:38]
```

```
Y_train = DF1.iloc[:, 3]
clf = KNeighborsClassifier(n_neighbors=7, p=1, weights='distance', n_jobs=-1, random_state = 1)
clf.fit(X_train,  Y_train)

# predicted label
pred = clf.predict(ex_test)
# prediction probability
prob = clf.predict_proba(ex_test)
# save predicted labels to a spreadsheet
m1 = pd.DataFrame(pred)
chem = DF3.iloc[:, 0:4]
predict_label = pd.concat([chem, m1], axis = 1)
predict_label.to_excel('C:/Users/Jie.Liu1/OneDrive - FDA/Desktop/toxrefdb-
2021/model_development_052021/model_output/knn/predict_label_external_knn.xlsx', index=False)

# performance
y_true = np.array(predict_label.iloc[:,3])
y_pred = np.array(predict_label.iloc[:,4])
TP = np.sum(predict_label[predict_label.iloc[:,3]==predict_label.iloc[:,4]].iloc[:,3]==1)
TN = np.sum(predict_label[predict_label.iloc[:,3]==predict_label.iloc[:,4]].iloc[:,3]==0)
FP = np.sum(predict_label[predict_label.iloc[:,3]!=predict_label.iloc[:,4]].iloc[:,3]==0)
FN = np.sum(predict_label[predict_label.iloc[:,3]!=predict_label.iloc[:,4]].iloc[:,3]==1)
accuracy = accuracy_score(y_true, y_pred)
sensitivity = recall_score(y_true, y_pred)
specificity = TN/(TN+FP)
balanced_ac = balanced_accuracy_score(y_true, y_pred)
tppr = precision_score(y_true, y_pred)
tnpr = TN/(TN+FN)
mcc = matthews_corrcoef(y_true, y_pred)
perf_1 = [accuracy, sensitivity, specificity, balanced_ac, tppr, tnpr, mcc]
perf_dt = pd.DataFrame(perf_1).T
perf_dt.insert(0, "Algorithm", ["DT"], True)
perf_dt.columns = ["Algorithm", "Accuracy","Sensitivity","Specificity", "Balanced accuracy", "PPR", "NPR", "MCC"]
perf_dt.to_excel('C:/Users/Jie.Liu1/OneDrive - FDA/Desktop/toxrefdb-
2021/model_development_052021/model_output/knn /perf_external_knn.xlsx', index=False)

# prediction probability
prob1 = np.array(prob)[:,1].reshape(1,29)
prob2 = pd.DataFrame(prob1).T
# add original chemical id and toxicity label
chem = DF3.iloc[:, 0:4]
prob3 = pd.concat([chem, prob2], axis = 1)
prob3.to_excel('C:/Users/Jie.Liu1/OneDrive - FDA/Desktop/toxrefdb-
2021/model_development_052021/model_output/knn/probability_to_active_external_knn.xlsx', index=False)
```

Python codes for Support Vector Machine (SVM)

```
#!/usr/bin/env python
# coding: utf-8

##################################################################################
# auto scale training and external data
# (1) autoscale trainging data:  transform value(ij) = (value(ij) - mean(j)(train)) / std(j)(train)
# (2) autoscale external data:  transform value (kj) = (value(kj) - mean(j)(train))/ std(j)(train)
#       i, k – row number; j – column number.
##################################################################################
# Change these paths to a location under the home directory
HOME = 'C:/Users/Jie.Liu1/OneDrive - FDA/Desktop/toxrefdb-2021/model_development_052021/data_input/'
input1 = os.path.join(HOME, 'variable_selection/275chem_var_selection_34des_autoscale.xlsx')
input2 = os.path.join(HOME, 'data input 05172021/Stratified-splitting-5fd.xlsx')

# data input1: toxrefdb data with 275 chemicals, 34 descriptors, and 1 toxicity label
DF1 = pd.read_excel(input1)
DF1.shape
# data input2: chemical indexes for 5-fold cross-validation, 500 times
DF2 = pd.read_excel(input2, header = None)
DF2.shape
############################################################################
# cross-validation
############################################################################
import time
start_time = time.time()
predicted = []
probability = []
chem_index = []

for i in range(0,500,1):
    for j in range(1,6,1):
        # select one fold of chemical index as the test data set
        test_index_0 = DF2.iloc[((j-1)*55+0):((j-1)*55+55), i]
        test_index = sorted(test_index_0)

        # subset test datasets
        test = DF1[DF1['chemical_id'].isin(test_index)]
        X_test = test.iloc[:, 4:38]
        Y_test = test.iloc[:, 3]

        #actual label
        index = test.iloc[:,0]
        chem_index.extend(index)

        # subset train datasets
        train = DF1[DF1['chemical_id'].isin(test_index) == False]
        X_train = train.iloc[:, 4:38]
        Y_train = train.iloc[:, 3]

        # classification
        clf = svm.SVC(random_state = 1, probability = False, kernel = 'linear', C = 1.6)
        clf.fit(X_train, Y_train)
```

```
        # predicted label
        pred = clf.predict(X_test)
        predicted.extend(pred)

        # predict confidence score -- train
        prob_train = clf.decision_function(X_train)
        min_train = min(prob_train)
        max_train = max(prob_train)

        # predict confidence score --- test
        prob = clf.decision_function(X_test)

        prob_test[prob >= max_train] = 1
        prob_test[prob <= min_train] = 0

        prob_test = np.where((prob >= 0) & (prob <= max(prob)), 0.5 + 0.5*prob/max(prob), 0.5 + 0.5*prob/abs(min(prob)))
        probability.extend(prob_test)
print("The whole time needed is %.2f seconds" %(time.time()-start_time))


# prediction label
l1 = np.array(chem_index).reshape(500,275)
l2 = pd.DataFrame(l1).T
m1 = np.array(predicted).reshape(500,275)
m2 = pd.DataFrame(m1).T
combine = []
for t in range(0,500,1):
    m = pd.merge(l2.iloc[:,t], m2.iloc[:,t], how="outer", left_index = True, right_index = True)
    m.columns = ["chem", "predict_label_"]
    m3 = m.sort_values(by=["chem"])
    m4 = pd.DataFrame(m3).iloc[:,1]
    combine.append(m4)
m5 = np.array(combine).reshape(500,275)
m6 = pd.DataFrame(m5).T
# add original chemical id and toxicity label
chem = DF1.iloc[:, 0:4]
predict_label = pd.concat([chem, m6], axis = 1)
predict_label.to_excel('C:/Users/Jie.Liu1/OneDrive - FDA/Desktop/toxrefdb-
2021/model_development_052021/model_output/svm/predict_label_svm.xlsx', index=False)

# performance
sensitivity = []
accuracy = []
specificity = []
balanced_ac = []
tppr = []
tnpr = []
mcc = []
for p in range(4,504,1):
    y_true = np.array(predict_label.iloc[:,3])
    y_pred = np.array(predict_label.iloc[:,p])
    TP = np.sum(predict_label[predict_label.iloc[:,3]==predict_label.iloc[:,p]].iloc[:,3]==1)
    TN = np.sum(predict_label[predict_label.iloc[:,3]==predict_label.iloc[:,p]].iloc[:,3]==0)
```

```
    FP = np.sum(predict_label[predict_label.iloc[:,3]!=predict_label.iloc[:,p]].iloc[:,3]==0)
    FN = np.sum(predict_label[predict_label.iloc[:,3]!=predict_label.iloc[:,p]].iloc[:,3]==1)
    recall = recall_score(y_true, y_pred)
    sensitivity.append(recall)
    acc = accuracy_score(y_true, y_pred)
    accuracy.append(acc)
    spec = TN/(TN+FP)
    specificity.append(spec)
    ba = balanced_accuracy_score(y_true, y_pred)
    balanced_ac.append(ba)
    precision = precision_score(y_true, y_pred)
    tppr.append(precision)
    tnprs = TN/(TN+FN)
    tnpr.append(tnprs)
    mccs = matthews_corrcoef(y_true, y_pred)
    mcc.append(mccs)

ac = pd.DataFrame(accuracy)
sen = pd.DataFrame(sensitivity)
sp = pd.DataFrame(specificity)
ba = pd.DataFrame(balanced_ac)
tppr_all = pd.DataFrame(tppr)
tnpr_all = pd.DataFrame(tnpr)
mcc_all = pd.DataFrame(mcc)
perf = pd.concat([ac, sen, sp, ba, tppr_all, tnpr_all, mcc_all], axis =1)
perf.columns = ["Accuracy","Sensitivity","Specificity", "Balanced accuracy", "PPR", "NPR", "MCC"]
perf.to_excel('C:/Users/Jie.Liu1/OneDrive - FDA/Desktop/toxrefdb-
2021/model_development_052021/model_output/svm/perf_svm.xlsx', index=False)

## prediction probability
## save predicted probability to active toxicity ("1") to a spreadsheet
prob1 = np.array(probability)[:,1].reshape(500,275)
prob2 = pd.DataFrame(prob1).T
combine_prob = []
for tt in range(0,500,1):
    prob = pd.merge(l2.iloc[:,tt], prob2.iloc[:,tt], how="outer", left_index = True, right_index = True)
    prob.columns = ["chem", "probability"]
    prob3 = prob.sort_values(by=["chem"])
    prob4 = pd.DataFrame(prob3).iloc[:,1]
    combine_prob.append(prob4)
prob5 = np.array(combine_prob).reshape(500,275)
prob6 = pd.DataFrame(prob5).T
# add original chemical id and toxicity label
chem = DF1.iloc[:, 0:4]
prob7 = pd.concat([chem, prob6], axis = 1)
prob7.to_excel('C:/Users/Jie.Liu1/OneDrive - FDA/Desktop/toxrefdb-
2021/model_development_052021/model_output/svm/probability_to_active_svm.xlsx', index=False)

###########################################################################
# external validation
###########################################################################
# import external data
input3 = os.path.join(HOME, 'external_data/external_data_34des_autoscale_new.xlsx')
DF3 = pd.read_excel(input3)
```

```
DF3.shape             # 29  38
ex_test = DF3.iloc[:, 4:38]
X_train = DF1.iloc[:, 4:38]
Y_train = DF1.iloc[:, 3]
clf = svm.SVC(random_state = 1, probability = False, kernel = 'linear', C = 1.6)
clf.fit(X_train,  Y_train)

# predicted label
pred = clf.predict(ex_test)

#predict confidence score -- train
prob_train = clf.decision_function(X_train)
min_train = min(prob_train)
max_train = max(prob_train)
# predict confidence score --- test
prob = clf.decision_function(ex_test)
prob_test[prob >= max_train] = 1
prob_test[prob <= min_train] = 0
prob_test = np.where((prob >= 0) & (prob <= max(prob)), 0.5 + 0.5*prob/max(prob), 0.5 + 0.5*prob/abs(min(prob)))

# save predicted labels to a spreadsheet
m1 = pd.DataFrame(pred)
chem = DF3.iloc[:, 0:4]
predict_label = pd.concat([chem, m1], axis = 1)
predict_label.to_excel('C:/Users/Jie.Liu1/OneDrive - FDA/Desktop/toxrefdb-
2021/model_development_052021/model_output/svm/predict_label_external_svm.xlsx', index=False)

# performance
y_true = np.array(predict_label.iloc[:,3])
y_pred = np.array(predict_label.iloc[:,4])
TP = np.sum(predict_label[predict_label.iloc[:,3]==predict_label.iloc[:,4]].iloc[:,3]==1)
TN = np.sum(predict_label[predict_label.iloc[:,3]==predict_label.iloc[:,4]].iloc[:,3]==0)
FP = np.sum(predict_label[predict_label.iloc[:,3]!=predict_label.iloc[:,4]].iloc[:,3]==0)
FN = np.sum(predict_label[predict_label.iloc[:,3]!=predict_label.iloc[:,4]].iloc[:,3]==1)
accuracy = accuracy_score(y_true, y_pred)
sensitivity = recall_score(y_true, y_pred)
specificity = TN/(TN+FP)
balanced_ac = balanced_accuracy_score(y_true, y_pred)
tppr = precision_score(y_true, y_pred)
tnpr = TN/(TN+FN)
mcc = matthews_corrcoef(y_true, y_pred)
perf_1 = [accuracy, sensitivity, specificity, balanced_ac, tppr, tnpr, mcc]
perf_dt = pd.DataFrame(perf_1).T
perf_dt.insert(0, "Algorithm", ["DT"], True)
perf_dt.columns = ["Algorithm", "Accuracy","Sensitivity","Specificity", "Balanced accuracy", "PPR", "NPR", "MCC"]
perf_dt.to_excel('C:/Users/Jie.Liu1/OneDrive - FDA/Desktop/toxrefdb-
2021/model_development_052021/model_output/svm /perf_external_svm.xlsx', index=False)

# prediction probability
prob1 = pd.DataFrame(prob_test)
# add original chemical id and toxicity label
chem = DF3.iloc[:, 0:4]
prob2 = pd.concat([chem, prob1], axis = 1)
prob2.to_excel('C:/Users/Jie.Liu1/OneDrive - FDA/Desktop/toxrefdb-
```

```
2021/model_development_052021/model_output/svm/probability_to_active_external_svm.xlsx', index=False)
```

Python codes for Linear Discriminant Analysis (LDA)

```
#!/usr/bin/env python
# coding: utf-8

###############################################################################
# auto scale training and external data
# (1) autoscale trainging data:  transform value(ij) = (value(ij) - mean(j)(train)) / std(j)(train)
# (2) autoscale external data:  transform value (kj) = (value(kj) - mean(j)(train))/ std(j)(train)
#       i, k – row number; j – column number.
###############################################################################

# Change these paths to a location under the home directory
HOME = 'C:/Users/Jie.Liu1/OneDrive - FDA/Desktop/toxrefdb-2021/model_development_052021/data_input/'
input1 = os.path.join(HOME, 'variable_selection/275chem_var_selection_34des_autoscale.xlsx')
input2 = os.path.join(HOME, 'data input 05172021/Stratified-splitting-5fd.xlsx')

# data input1: toxrefdb data with 275 chemicals, 34 descriptors, and 1 toxicity label
DF1 = pd.read_excel(input1)
DF1.shape
# data input2: chemical indexes for 5-fold cross-validation, 500 times
DF2 = pd.read_excel(input2, header = None)
DF2.shape

#########################################################################
# cross-validation
#########################################################################
import time
start_time = time.time()
predicted = []
probability = []
chem_index = []

for i in range(0,500,1):
    for j in range(1,6,1):
        # select one fold of chemical index as the test data set
        test_index_0 = DF2.iloc[((j-1)*55+0):((j-1)*55+55), i]
        test_index = sorted(test_index_0)

        # subset test datasets
        test = DF1[DF1['chemical_id'].isin(test_index)]
        X_test = test.iloc[:, 4:38]
        Y_test = test.iloc[:, 3]

        #actual label
        index = test.iloc[:,0]
        chem_index.extend(index)

        # subset train datasets
        train = DF1[DF1['chemical_id'].isin(test_index) == False]
        X_train = train.iloc[:, 4:38]
        Y_train = train.iloc[:, 3]
```

```
        # classification
        clf = LinearDiscriminantAnalysis(solver='lsqr', shrinkage=0.6)
        clf.fit(X_train, Y_train)

        # predicted label
        pred = clf.predict(X_test)
        predicted.extend(pred)

        # probability
        prob = clf.predict_proba(X_test)
        probability.extend(prob)

print("The whole time needed is %.2f seconds" %(time.time()-start_time))


# prediction label
l1 = np.array(chem_index).reshape(500,275)
l2 = pd.DataFrame(l1).T
m1 = np.array(predicted).reshape(500,275)
m2 = pd.DataFrame(m1).T
combine = []
for t in range(0,500,1):
    m = pd.merge(l2.iloc[:,t], m2.iloc[:,t], how="outer", left_index = True, right_index = True)
    m.columns = ["chem", "predict_label_"]
    m3 = m.sort_values(by=["chem"])
    m4 = pd.DataFrame(m3).iloc[:,1]
    combine.append(m4)
m5 = np.array(combine).reshape(500,275)
m6 = pd.DataFrame(m5).T
# add original chemical id and toxicity label
chem = DF1.iloc[:, 0:4]
predict_label = pd.concat([chem, m6], axis = 1)
predict_label.to_excel('C:/Users/Jie.Liu1/OneDrive - FDA/Desktop/toxrefdb-
2021/model_development_052021/model_output/lda/predict_label_lda.xlsx', index=False)

# performance
sensitivity = []
accuracy = []
specificity = []
balanced_ac = []
tppr = []
tnpr = []
mcc = []

for p in range(4,504,1):
    y_true = np.array(predict_label.iloc[:,3])
    y_pred = np.array(predict_label.iloc[:,p])
    TP = np.sum(predict_label[predict_label.iloc[:,3]==predict_label.iloc[:,p]].iloc[:,3]==1)
    TN = np.sum(predict_label[predict_label.iloc[:,3]==predict_label.iloc[:,p]].iloc[:,3]==0)
    FP = np.sum(predict_label[predict_label.iloc[:,3]!=predict_label.iloc[:,p]].iloc[:,3]==0)
    FN = np.sum(predict_label[predict_label.iloc[:,3]!=predict_label.iloc[:,p]].iloc[:,3]==1)

    recall = recall_score(y_true, y_pred)
    sensitivity.append(recall)
```

```
    acc = accuracy_score(y_true, y_pred)
    accuracy.append(acc)
    spec = TN/(TN+FP)
    specificity.append(spec)
    ba = balanced_accuracy_score(y_true, y_pred)
    balanced_ac.append(ba)
    precision = precision_score(y_true, y_pred)
    tppr.append(precision)
    tnprs = TN/(TN+FN)
    tnpr.append(tnprs)
    mccs = matthews_corrcoef(y_true, y_pred)
    mcc.append(mccs)

ac = pd.DataFrame(accuracy)
sen = pd.DataFrame(sensitivity)
sp = pd.DataFrame(specificity)
ba = pd.DataFrame(balanced_ac)
tppr_all = pd.DataFrame(tppr)
tnpr_all = pd.DataFrame(tnpr)
mcc_all = pd.DataFrame(mcc)
perf = pd.concat([ac, sen, sp, ba, tppr_all, tnpr_all, mcc_all], axis =1)
perf.columns = ["Accuracy","Sensitivity","Specificity", "Balanced accuracy", "PPR", "NPR", "MCC"]
perf.to_excel('C:/Users/Jie.Liu1/OneDrive - FDA/Desktop/toxrefdb-
2021/model_development_052021/model_output/lda/perf_lda.xlsx', index=False)

## prediction probability
## save predicted probability to active toxicity ("1") to a spreadsheet
prob1 = np.array(probability)[:,1].reshape(500,275)
prob2 = pd.DataFrame(prob1).T
combine_prob = []
for tt in range(0,500,1):
    prob = pd.merge(l2.iloc[:,tt], prob2.iloc[:,tt], how="outer", left_index = True, right_index = True)
    prob.columns = ["chem", "probability"]
    prob3 = prob.sort_values(by=["chem"])
    prob4 = pd.DataFrame(prob3).iloc[:,1]
    combine_prob.append(prob4)
prob5 = np.array(combine_prob).reshape(500,275)
prob6 = pd.DataFrame(prob5).T
# add original chemical id and toxicity label
chem = DF1.iloc[:, 0:4]
prob7 = pd.concat([chem, prob6], axis = 1)
prob7.to_excel('C:/Users/Jie.Liu1/OneDrive - FDA/Desktop/toxrefdb-
2021/model_development_052021/model_output/lda/probability_to_active_lda.xlsx', index=False)

##########################################################################
# external validation
##########################################################################

# import external data
input3 = os.path.join(HOME, 'external_data/external_data_34des_autoscale_new.xlsx')
DF3 = pd.read_excel(input3)
DF3.shape              # 29  38
ex_test = DF3.iloc[:, 4:38]
X_train = DF1.iloc[:, 4:38]
```

```
Y_train = DF1.iloc[:, 3]
clf = LinearDiscriminantAnalysis(solver='lsqr', shrinkage=0.6)
clf.fit(X_train,  Y_train)

# predicted label
pred = clf.predict(ex_test)
# prediction probability
prob = clf.predict_proba(ex_test)
# save predicted labels to a spreadsheet
m1 = pd.DataFrame(pred)
chem = DF3.iloc[:, 0:4]
predict_label = pd.concat([chem, m1], axis = 1)
predict_label.to_excel('C:/Users/Jie.Liu1/OneDrive - FDA/Desktop/toxrefdb-
2021/model_development_052021/model_output/lda/predict_label_external_lda.xlsx', index=False)

# performance
y_true = np.array(predict_label.iloc[:,3])
y_pred = np.array(predict_label.iloc[:,4])
TP = np.sum(predict_label[predict_label.iloc[:,3]==predict_label.iloc[:,4]].iloc[:,3]==1)
TN = np.sum(predict_label[predict_label.iloc[:,3]==predict_label.iloc[:,4]].iloc[:,3]==0)
FP = np.sum(predict_label[predict_label.iloc[:,3]!=predict_label.iloc[:,4]].iloc[:,3]==0)
FN = np.sum(predict_label[predict_label.iloc[:,3]!=predict_label.iloc[:,4]].iloc[:,3]==1)
accuracy = accuracy_score(y_true, y_pred)
sensitivity = recall_score(y_true, y_pred)
specificity = TN/(TN+FP)
balanced_ac = balanced_accuracy_score(y_true, y_pred)
tppr = precision_score(y_true, y_pred)
tnpr = TN/(TN+FN)
mcc = matthews_corrcoef(y_true, y_pred)
perf_1 = [accuracy, sensitivity, specificity, balanced_ac, tppr, tnpr, mcc]
perf_dt = pd.DataFrame(perf_1).T
perf_dt.insert(0, "Algorithm", ["DT"], True)
perf_dt.columns = ["Algorithm", "Accuracy","Sensitivity","Specificity", "Balanced accuracy", "PPR", "NPR", "MCC"]
perf_dt.to_excel('C:/Users/Jie.Liu1/OneDrive - FDA/Desktop/toxrefdb-
2021/model_development_052021/model_output/lda /perf_external_lda.xlsx', index=False)

# prediction probability
prob1 = np.array(prob)[:,1].reshape(1,29)
prob2 = pd.DataFrame(prob1).T
# add original chemical id and toxicity label
chem = DF3.iloc[:, 0:4]
prob3 = pd.concat([chem, prob2], axis = 1)
prob3.to_excel('C:/Users/Jie.Liu1/OneDrive - FDA/Desktop/toxrefdb-
2021/model_development_052021/model_output/lda/probability_to_active_external_lda.xlsx', index=False)
```

Python codes for Logistic Regression (LR)

```
#!/usr/bin/env python
# coding: utf-8

##############################################################################
# auto scale training and external data
# (1) autoscale trainging data:  transform value(ij) = (value(ij) - mean(j)(train)) / std(j)(train)
# (2) autoscale external data:  transform value (kj) = (value(kj) - mean(j)(train))/ std(j)(train)
#      i, k – row number; j – column number.
##############################################################################

# Change these paths to a location under the home directory
HOME = 'C:/Users/Jie.Liu1/OneDrive - FDA/Desktop/toxrefdb-2021/model_development_052021/data_input/'
input1 = os.path.join(HOME, 'variable_selection/275chem_var_selection_34des_autoscale.xlsx')
input2 = os.path.join(HOME, 'data input 05172021/Stratified-splitting-5fd.xlsx')

# data input1: toxrefdb data with 275 chemicals, 34 descriptors, and 1 toxicity label
DF1 = pd.read_excel(input1)
DF1.shape
# data input2: chemical indexes for 5-fold cross-validation, 500 times
DF2 = pd.read_excel(input2, header = None)
DF2.shape

########################################################################
# cross-validation
########################################################################
import time
start_time = time.time()
predicted = []
probability = []
chem_index = []

for i in range(0,500,1):
   for j in range(1,6,1):
      # select one fold of chemical index as the test data set
      test_index_0 = DF2.iloc[((j-1)*55+0):((j-1)*55+55), i]
      test_index = sorted(test_index_0)

      # subset test datasets
      test = DF1[DF1['chemical_id'].isin(test_index)]
      X_test = test.iloc[:, 4:38]
      Y_test = test.iloc[:, 3]

      #actual label
      index = test.iloc[:,0]
      chem_index.extend(index)

      # subset train datasets
      train = DF1[DF1['chemical_id'].isin(test_index) == False]
      X_train = train.iloc[:, 4:38]
      Y_train = train.iloc[:, 3]
```

```
        # classification
        clf = LogisticRegression(solver = 'liblinear', C = 4.0, penalty = 'l2', random_state = 1)
        clf.fit(X_train, Y_train)

        # predicted label
        pred = clf.predict(X_test)
        predicted.extend(pred)

        # probability
        prob = clf.predict_proba(X_test)
        probability.extend(prob)

print("The whole time needed is %.2f seconds" %(time.time()-start_time))


# prediction label
l1 = np.array(chem_index).reshape(500,275)
l2 = pd.DataFrame(l1).T
m1 = np.array(predicted).reshape(500,275)
m2 = pd.DataFrame(m1).T
combine = []
for t in range(0,500,1):
    m = pd.merge(l2.iloc[:,t], m2.iloc[:,t], how="outer", left_index = True, right_index = True)
    m.columns = ["chem", "predict_label_"]
    m3 = m.sort_values(by=["chem"])
    m4 = pd.DataFrame(m3).iloc[:,1]
    combine.append(m4)
m5 = np.array(combine).reshape(500,275)
m6 = pd.DataFrame(m5).T
# add original chemical id and toxicity label
chem = DF1.iloc[:, 0:4]
predict_label = pd.concat([chem, m6], axis = 1)
predict_label.to_excel('C:/Users/Jie.Liu1/OneDrive - FDA/Desktop/toxrefdb-
2021/model_development_052021/model_output/lr/predict_label_lr.xlsx', index=False)

# performance
sensitivity = []
accuracy = []
specificity = []
balanced_ac = []
tppr = []
tnpr = []
mcc = []

for p in range(4,504,1):
    y_true = np.array(predict_label.iloc[:,3])
    y_pred = np.array(predict_label.iloc[:,p])
    TP = np.sum(predict_label[predict_label.iloc[:,3]==predict_label.iloc[:,p]].iloc[:,3]==1)
    TN = np.sum(predict_label[predict_label.iloc[:,3]==predict_label.iloc[:,p]].iloc[:,3]==0)
    FP = np.sum(predict_label[predict_label.iloc[:,3]!=predict_label.iloc[:,p]].iloc[:,3]==0)
    FN = np.sum(predict_label[predict_label.iloc[:,3]!=predict_label.iloc[:,p]].iloc[:,3]==1)
    recall = recall_score(y_true, y_pred)
    sensitivity.append(recall)
    acc = accuracy_score(y_true, y_pred)
```

```
    accuracy.append(acc)
    spec = TN/(TN+FP)
    specificity.append(spec)
    ba = balanced_accuracy_score(y_true, y_pred)
    balanced_ac.append(ba)
    precision = precision_score(y_true, y_pred)
    tppr.append(precision)
    tnprs = TN/(TN+FN)
    tnpr.append(tnprs)
    mccs = matthews_corrcoef(y_true, y_pred)
    mcc.append(mccs)

ac = pd.DataFrame(accuracy)
sen = pd.DataFrame(sensitivity)
sp = pd.DataFrame(specificity)
ba = pd.DataFrame(balanced_ac)
tppr_all = pd.DataFrame(tppr)
tnpr_all = pd.DataFrame(tnpr)
mcc_all = pd.DataFrame(mcc)
perf = pd.concat([ac, sen, sp, ba, tppr_all, tnpr_all, mcc_all], axis =1)
perf.columns = ["Accuracy","Sensitivity","Specificity", "Balanced accuracy", "PPR", "NPR", "MCC"]
perf.to_excel('C:/Users/Jie.Liu1/OneDrive - FDA/Desktop/toxrefdb-
2021/model_development_052021/model_output/lr/perf_lr.xlsx', index=False)

## prediction probability
## save predicted probability to active toxicity ("1") to a spreadsheet
prob1 = np.array(probability)[:,1].reshape(500,275)
prob2 = pd.DataFrame(prob1).T
combine_prob = []
for tt in range(0,500,1):
    prob = pd.merge(l2.iloc[:,tt], prob2.iloc[:,tt], how="outer", left_index = True, right_index = True)
    prob.columns = ["chem", "probability"]
    prob3 = prob.sort_values(by=["chem"])
    prob4 = pd.DataFrame(prob3).iloc[:,1]
    combine_prob.append(prob4)
prob5 = np.array(combine_prob).reshape(500,275)
prob6 = pd.DataFrame(prob5).T
# add original chemical id and toxicity label
chem = DF1.iloc[:, 0:4]
prob7 = pd.concat([chem, prob6], axis = 1)
prob7.to_excel('C:/Users/Jie.Liu1/OneDrive - FDA/Desktop/toxrefdb-
2021/model_development_052021/model_output/lr/probability_to_active_lr.xlsx', index=False)

##########################################################################
# external validation
##########################################################################
# import external data
input3 = os.path.join(HOME, 'external_data/external_data_34des_autoscale_new.xlsx')
DF3 = pd.read_excel(input3)
DF3.shape          # 29  38
ex_test = DF3.iloc[:, 4:38]
X_train = DF1.iloc[:, 4:38]
Y_train = DF1.iloc[:, 3]
```

```
clf = LogisticRegression(solver = 'liblinear', C = 4.0, penalty = 'l2', random_state = 1)
clf.fit(X_train,  Y_train)

# predicted label
pred = clf.predict(ex_test)
# prediction probability
prob = clf.predict_proba(ex_test)
# save predicted labels to a spreadsheet
m1 = pd.DataFrame(pred)
chem = DF3.iloc[:, 0:4]
predict_label = pd.concat([chem, m1], axis = 1)
predict_label.to_excel('C:/Users/Jie.Liu1/OneDrive - FDA/Desktop/toxrefdb-
2021/model_development_052021/model_output/lr/predict_label_external_lr.xlsx', index=False)

# performance
y_true = np.array(predict_label.iloc[:,3])
y_pred = np.array(predict_label.iloc[:,4])
TP = np.sum(predict_label[predict_label.iloc[:,3]==predict_label.iloc[:,4]].iloc[:,3]==1)
TN = np.sum(predict_label[predict_label.iloc[:,3]==predict_label.iloc[:,4]].iloc[:,3]==0)
FP = np.sum(predict_label[predict_label.iloc[:,3]!=predict_label.iloc[:,4]].iloc[:,3]==0)
FN = np.sum(predict_label[predict_label.iloc[:,3]!=predict_label.iloc[:,4]].iloc[:,3]==1)
accuracy = accuracy_score(y_true, y_pred)
sensitivity = recall_score(y_true, y_pred)
specificity = TN/(TN+FP)
balanced_ac = balanced_accuracy_score(y_true, y_pred)
tppr = precision_score(y_true, y_pred)
tnpr = TN/(TN+FN)
mcc = matthews_corrcoef(y_true, y_pred)
perf_1 = [accuracy, sensitivity, specificity, balanced_ac, tppr, tnpr, mcc]
perf_dt = pd.DataFrame(perf_1).T
perf_dt.insert(0, "Algorithm", ["DT"], True)
perf_dt.columns = ["Algorithm", "Accuracy","Sensitivity","Specificity", "Balanced accuracy", "PPR", "NPR", "MCC"]
perf_dt.to_excel('C:/Users/Jie.Liu1/OneDrive - FDA/Desktop/toxrefdb-
2021/model_development_052021/model_output/lr/perf_external_lr.xlsx', index=False)

# prediction probability
prob1 = np.array(prob)[:,1].reshape(1,29)
prob2 = pd.DataFrame(prob1).T
# add original chemical id and toxicity label
chem = DF3.iloc[:, 0:4]
prob3 = pd.concat([chem, prob2], axis = 1)
prob3.to_excel('C:/Users/Jie.Liu1/OneDrive - FDA/Desktop/toxrefdb-
2021/model_development_052021/model_output/lr/probability_to_active_external_lr.xlsx', index=False)
```

Matlab codes for Decision Forest (DF)

```
######## 5-f CV by Decision Forest modeling

pc=zeros(275,500);
pb=pc;
varU=zeros(34,1);
for i=1:500
  for j=1:5
    xte=x0(ind((j-1)*55+1:j*55,i),:);
    xtr=x0;
    xtr(ind((j-1)*55+1:j*55,i),:)=[];
    yte=y(ind((j-1)*55+1:j*55,i));
    ytr=y;
    ytr(ind((j-1)*55+1:j*55,i))=[];
    zzz=df4c2020Pred(ytr,xtr,xte,3, 2, 7, 14, 'gdi');
    pb(ind((j-1)*55+1:j*55,i),i)=zzz.prob;
    pc(ind((j-1)*55+1:j*55,i),i)=zzz.pred;
    varU=varU + zzz.varUsed;
  end
  fprintf('Done %d\n', i),
end

yy=repmat(y, 1,500);
d=(pc-yy).*(pc-yy);
acc=1-sum(d)/275;
fpp=sum(d(find(y==0),:));
fnp=sum(d(find(y==1),:));
tpp=94-fnp;
tnp=181-fpp;
spec=tnp/181;
sens=tpp/94;
mcc=(tpp.*tnp-fpp.*fnp)./sqrt((tpp+fpp).*(tpp+fnp).*(tnp+fnp).*(tnp+fpp));
tppr=tpp./(tpp+fpp);
tnpr=tnp./(tnp+fnp);
bacc=(spec+sens)/2;
plot(1:500, acc, 'ro');
hold on;
plot(1:500, spec, 'bo');
plot(1:500, sens, 'mo');
plot(1:500, tppr, 'co');
plot(1:500, tnpr, 'yo');
plot(1:500, bacc, 'go');
plot(1:500, mcc, 'ko');
hold off;
ylim([0 0.9]);
xlabel('5-fold cross validation run');
ylabel('Performance');
legend({'Accuracy','Specificity','Sensitivity','TPPR','TNPR',...
'B-Accuracy','MCC'}, 'Location','northoutside', 'NumColumns',4);

saveas(gcf,'C:\Manuscripts\Jie Liu\ToxRefDB\input data\data input 94 positive\DF-3\DF.fig');
fig = gcf;
```

```
fig.PaperPositionMode = 'auto';
print('C:\Manuscripts\Jie Liu\ToxRefDB\input data\data input 94 positive\DF-3\DF.jpg', '-djpeg','-r600');
ax = gca;
exportgraphics(ax,'C:\Manuscripts\Jie Liu\ToxRefDB\input data\data input 94 positive\DF-
3\DF.pdf','ContentType','vector');


*******************External validation*******************

zExtPred=df4c2020Pred(y,x0,xExt,3, 2, 7, 14, 'gdi');


save('C:\Manuscripts\Jie Liu\ToxRefDB\input data\data input 94 positive\DF-3\Results-DF.mat');


********************* df4c2020Pred

function [pred]=df4c2020Pred(y,x,xte,nt,ndd,mls,mps,sm)

% df4c2020Pred builds a DF classification model from y and x, then predict on xte
% for MatLab version 2020
%
% output:
% pred---prediction results
%
% input:
% y---vector of classes of samples
% x---variable matrix
% xte---independent variables of testing set
% sm---node splitting method: 'gdi' (default) for Gini's diversity index;
%                         'twoing' for the twoing rule;
%                         'deviance' for maximum deviance reduction.
% mls---minimum leaf size (default 5)
% mps---minimum parent size (default 10)
% nt---number of trees to make a forest (default 7)
% ndd---number of descriptors to delete for each tree (default 3)
%
%======================================================================
%   Copyright: NCTR/FDA
%   Written by Dr. Huixiao Hong May 21, 2021

if nargin<3,
   error('dtCV:TooFewInputs','Requires at least two input arguments.');
end

[nn nm]=size(xte);

% Process inputs

if nargin<4
   nt = 7;
end
if nargin<5
   ndd = 3;
```

```
end
if nargin<6
   mls = 5;
end
if nargin<7
   mps = 10;
end
if nargin<8
   sm = 'gdi';
end

% Construct output structure

pred.probt = [];
pred.predt = [];
pred.varUsed = zeros(nm,1);
pred.pred = zeros(nn,1);

% Begin to run cross validation
tstart = cputime;
      for tn = 1:nt
         ndd0=0;
%          tree = fitctree(x,y);
         tree = fitctree(x,y,'MinLeafSize', mls, 'MinParentSize', mps, 'SplitCriterion', sm);
         [pc, sc]=predict(tree, xte);

         for k=1:tree.NumNodes
            varU = cell2mat(tree.CutPredictor(k));
            nnk = length(varU);
            if nnk > 1
               varN = str2num(varU(2:nnk));
               pred.varUsed(varN) = pred.varUsed(varN) + 1;
               if ndd0 < (ndd+1)
                  x(:,varN)=0;
                  ndd0=ndd0+1;
               end
            end
         end
         pred.probt = [pred.probt, sc(:,2)];
         pred.predt = [pred.predt, pc];
      end
      pred.prob = mean(pred.probt,2);
      pred.pred(find(pred.prob>=0.5)) = tree.ClassNames(2);

   tend = cputime;
 %   fprintf('Start at %d; Finish at %d.   CPU time used = %d seconds\n', tstart, tend, tend-tstart);
```
